# A step-economic and one-pot access to chiral C^α^-tetrasubstituted α-amino acid derivatives *via* a bicyclic imidazole-catalyzed direct enantioselective *C*-acylation†

**DOI:** 10.1039/d0sc00808g

**Published:** 2020-04-24

**Authors:** Mo Wang, Muxing Zhou, Lu Zhang, Zhenfeng Zhang, Wanbin Zhang

**Affiliations:** Shanghai Key Laboratory for Molecular Engineering of Chiral Drugs, School of Chemistry and Chemical Engineering, Frontiers Science Center for Transformative Molecules, Shanghai Jiao Tong University Shanghai 200240 China wanbin@sjtu.edu.cn zhenfeng@sjtu.edu.cn; School of Pharmacy, Shanghai Jiao Tong University Shanghai 200240 China

## Abstract

C^α^-Tetrasubstituted α-amino acids are ubiquitous and unique structural units in bioactive natural products and pharmaceutical compounds. The asymmetric synthesis of these molecules has attracted a lot of attention, but a more efficient method is still greatly desired. Here we describe the first sequential four-step acylation reaction for the efficient synthesis of chiral C^α^-tetrasubstituted α-amino acid derivatives from simple *N*-acylated amino acids *via* an auto-tandem catalysis using a single nucleophilic catalyst. The synthetic efficiency is improved *via* a direct enantioselective *C*-acylation; the methodology affords the corresponding C^α^-tetrasubstituted α-amino acid derivatives with excellent enantioselectivities (up to 99% ee). This step-economic, one-pot, and auto-tandem strategy provides facile access to important chiral building blocks, such as peptides, serines, and oxazolines, which are often used in medicinal and synthetic chemistry.

## Abstract 2

The first four-step sequential reaction for the synthesis of C^α^-tetrasubstituted chiral α-amino acid derivatives *via* auto-tandem catalysis has been developed.

## Graphical abstract

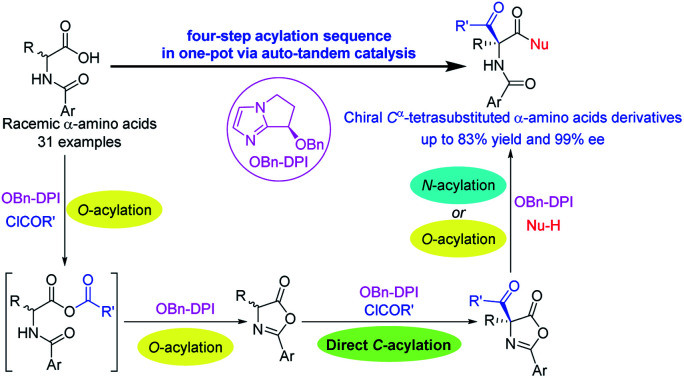

## Introduction

**Fig. 1:**
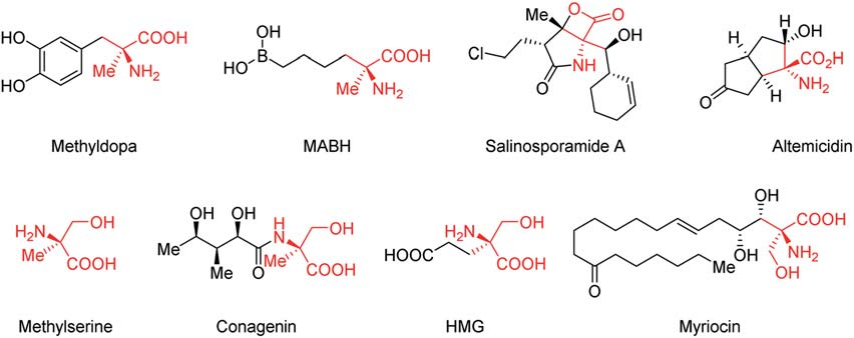
Examples of C^α^-tetrasubstituted α-amino acid derivatives with biological activities and physiological effects.

**Scheme 1:**
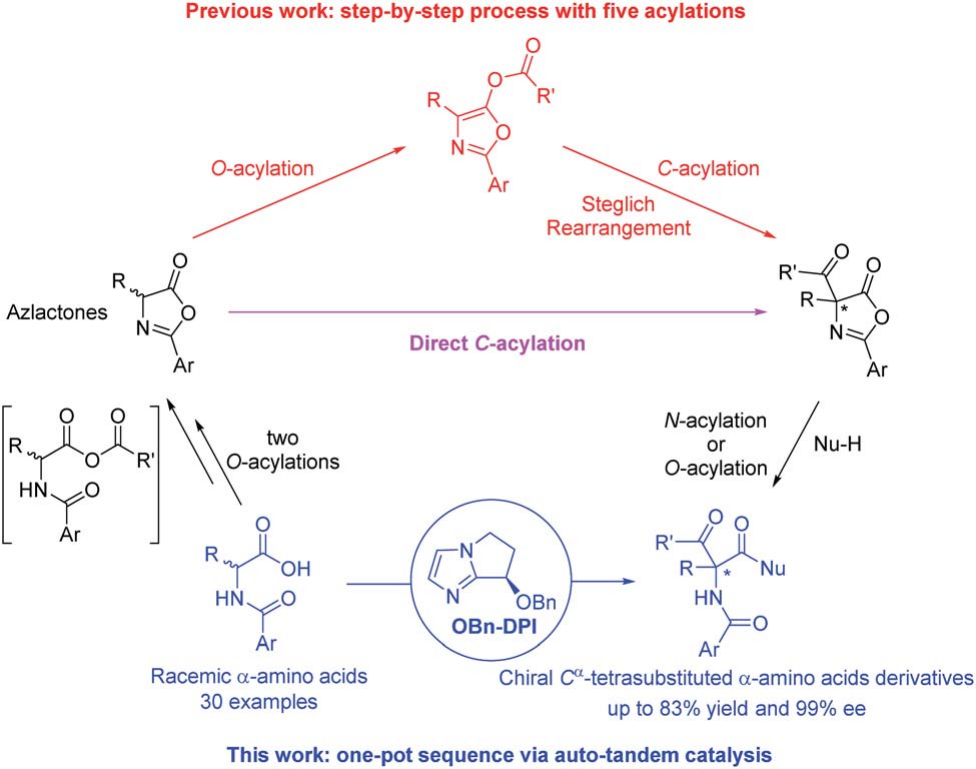
Our new approach to enantiopure C^α^-tetrasubstituted α-amino acid derivatives by a four-step acylation sequence using auto-tandem catalysis.

C^α^-Tetrasubstituted α-amino acids are ubiquitous and unique structural units in many natural products and synthetic compounds, many of which exhibit significant biological activities and physiological effects (Fig. 1).^1^ Several feasible methodologies have been developed for the synthesis of C^α^-tetrasubstituted α-amino acids, ranging from the use of chiral auxiliaries to the presently reported catalytic models.^2^ These include: (1) the enantioselective addition of alkyl, aryl, or even acyl precursors (Strecker reaction followed by hydrolysis) to ketimines,^3^ and; (2) the asymmetric α-alkylation, arylation, or acylation of α-substituted amino acid derivatives.^4,5^ However, due to the challenges imposed by C^α^-tetrasubstituted α-amino acids, efficient methods are still lacking, especially for the synthesis of α-acyl-substituted C^α^-tetrasubstituted α-amino acid derivatives.^5^ The most commonly used approach relies on the *O*-acylation of azlactones followed by a Lewis base-catalyzed asymmetric *O*- to *C*-acyl transfer (well-known as Steglich rearrangement, red arrows in Scheme 1). After the initial work developed by Fu *et al.*,^5*a*^ several improvements on the asymmetric Steglich rearrangement have been reported by many other research groups.^5*b*–*k*^ Our group has also developed an efficient bicyclic imidazole organocatalyst for this reaction, which can be easily synthesized from imidazole in only three steps.^5*f*^ However, the efficiency of this transformation is hindered by the prefabrication of unstable azlactones and relatively inert *O*-acylated azlactones. Such inefficiencies are present in many other transformations which lead to poor practical applicability.^5*b*,6^

In order to improve the synthetic efficiency, minimizing the number of steps of the synthetic sequence is important. This strategy, termed step-economy, has frequently been employed in advanced total syntheses by utilizing new reactions.^7^ Herein, we would like to put forward a more general approach in which a two-step rearrangement process (AB + C → AB–C → C–AB) is replaced by a direct reaction (AB + C → C–AB) by altering chemoselectivity. As the first example, we recently developed a bicyclic imidazole-catalyzed direct enantioselective *C*-acylation of benzofuran-2(3*H*)-ones and 2-oxindoles.^8^ For the most part, the starting material is directly converted to the *C*-acylated product but not *via* the relatively inert *O*-acylated intermediate. As a result, the efficiency for the synthesis of the desired products is dramatically improved compared to the corresponding Black rearrangement.^9^ Inspired by this, and in continuation of our interest in the construction of C^α^-tetrasubstituted α-amino acids,^10^ we propose a direct enantioselective *C*-acylation of azlactones with the purpose of improving the synthetic efficiency for the preparation of 4-carboxyazlactones (pink arrow in Scheme 1).^11^ To further improve the synthetic efficiency, simplifying the operations *via* a sequential process in one pot is an applicable method. This strategy, termed pot-economy, has been widely used for the construction of multiple bonds in one pot.^12^ Generally, different catalysts are used for different reaction steps (tandem catalysis). Obviously, carrying out all the reaction steps with a single catalyst would be more beneficial (auto-tandem catalysis).^13^ This strategy, termed catalyst-economy, has often been employed in two-step sequences but seldom in multi-step sequences.^13*c*^ During the initial research concerning the direct enantioselective *C*-acylation of azlactones, we found that the starting azlactones could be prepared *via* intramolecular *O*-acylation of *in situ* generated anhydrides (shown in the square brackets in Scheme 1); the produced 4-carboxyazlactones could be readily derivatized by another acylation to produce various C^α^-tetrasubstituted α-amino acid derivatives. Therefore, by combining the aforementioned step-, pot-, and catalyst-economy strategies, we aimed to design a four-step acylation sequence for the efficient synthesis of chiral C^α^-tetrasubstituted α-amino acid derivatives and biologically active dipeptides from simple *N*-acylated amino acids. The reaction would proceed *via* auto-tandem catalysis using a single bicyclic imidazole nucleophilic catalyst (blue part in Scheme 1). To the best of our knowledge, this type of sequential reaction that achieves excellent enantioselectivity has not been reported previously.

## Results and discussion

We initially focused on screening the reaction conditions for the first three steps in the sequence in one pot. (4-Methoxybenzoyl)alanine **1a** was selected as the starting material and benzylamine was used as the nucleophilic reagent to derivatize the produced 4-carboxyazlactone shown in the square brackets in Table 1. Over the past decade, we have developed a series of bicyclic imidazole nucleophilic catalysts which have been successfully applied in a number of different reactions by our group^5*f*,8,9,14^ and other groups.^15^ We envisaged that the bicyclic imidazole would be able to catalyze all the steps in the acylation sequence. Several bicyclic imidazole catalysts were screened for the sequential acylation of **1a** with benzyl chloroformate as the acylating agent and di(isopropyl)ethylamine (DIPEA) as a base to afford product **2a** (Table 1, entries 1–4). Catalysts bearing an alkoxy group gave better ee than those bearing an acyloxy group, and catalyst **OBn-DPI** was found to give the best result. Replacing the acylating agent, benzyl chloroformate with allyl chloroformate or phenyl chloroformate, afforded the desired products in lower yield and ee (entries 5 and 6, respectively). Use of ethyl chloroformate only afforded a trace amount of the product (entry 7). The base triethylamine (TEA) was also applied in this reaction but no product was observed (entry 8). The effect of solvents was studied and toluene provided the product with the highest ee and satisfactory conversion (entries 4, 9–14). After preliminary screening, the catalyst **OBn-DPI**, acylating agent benzyl chloroformate, base DIPEA and solvent toluene were chosen as the optimal conditions for further study, affording the desired product in 78% yield and with 91% ee (entry 4).

**Table 1:** The effect of catalyst, acylating reagent, base, and solvent

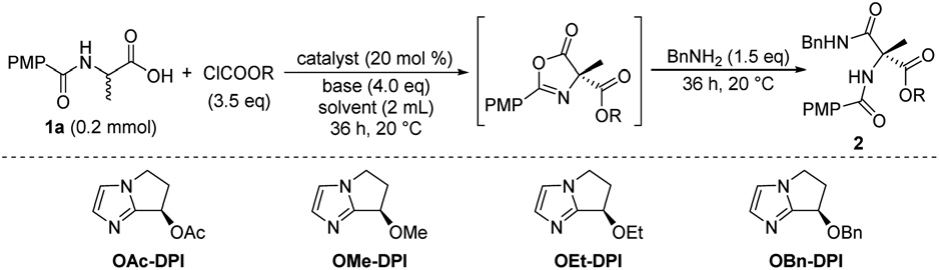					
Entry^a^	Catalyst	Acylating reagent	Solvent	Yield^b^ (%)	ee^c^ (%)
1	OAc-DPI	ClCOOBn	Toluene	77	84
2	OMe-DPI	ClCOOBn	Toluene	62	87
3	OEt-DPI	ClCOOBn	Toluene	65	88
4	OBn-DPI	ClCOOBn	Toluene	78	91
5	OBn-DPI	ClCOOallyl	Toluene	71	91
6	OBn-DPI	ClCOOPh	Toluene	81	60
7	OBn-DPI	ClCOOEt	Toluene	Trace	—
8^d^	OBn-DPI	ClCOOBn	Toluene	—	—
9	OBn-DPI	ClCOOBn	THF	80	90
10	OBn-DPI	ClCOOBn	Dioxane	77	90
11	OBn-DPI	ClCOOBn	Et2O	30	91
12	OBn-DPI	ClCOOBn	MTBE	66	91
13	OBn-DPI	ClCOOBn	DCM	45	88
14	OBn-DPI	ClCOOBn	*t*-AA	76	71

a Conditions: **1a** (0.1 M), ClCOOR (3.5 eq.), catalyst (20 mol%), DIPEA (4.0 eq.), solvent (2 mL), 20 °C, 36 h, unless otherwise noted.

b Yields were calculated from ^1^H NMR spectra.

c The ee values were calculated from HPLC spectra.

d TEA was used instead of DIPEA and only azlactone without COOBn substituent was obtained together with some NEt_2_COOBn.

Next, the effect of reaction temperature was researched (Table 2). At a lower reaction temperature of 0 °C for 36 h, product **2a** was obtained with same yield and better ee compared to that of 20 °C (entries 1 and 2). When the reaction temperature was reduced to −20 °C for 36 h, the yield of **2a** was reduced to 68% while the enantioselectivity increased to 95% (entry 3). Extending the reaction time to 48 hours gave the product in an increased yield (76%, entry 4). So the reaction was conducted for a longer time (72 h) to give better yield when reaction temperature was further decreased (entries 5–7). Finally, −55 °C was found to be the optimized temperature with 78% yield and 99% ee (entry 6). Then the equivalents of ClCOOBn were investigated and it was found that 3.0 equivalents of ClCOOBn afforded the best results (entries 6, 8 and 9). Therefore, the sequential reaction of substrate **1a** with 3.0 equivalents of benzyl chloroformate, 4.0 equivalents of DIPEA and 20 mol% **OBn-DPI** at −55 °C over 72 h was optimal for both reactivity and enantioselectivity, leading to the product **2a** in 78% yield and 99% ee (entry 8).

**Table 2:** The effect of reaction temperature

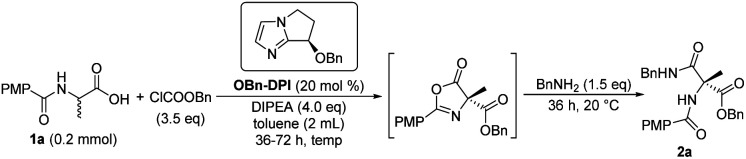				
Entry^a^	Temp. (°C)	Time (h)	Yield^b^ (%)	ee^c^ (%)
1	20	36	78	91
2	0	36	78	93
3	−20	36	68	95
4	−20	48	76	95
5	−50	72	78	98
6	−55	72	78	99
7	−60	72	77	98
8^d^	−55	72	78	99
9^e^	−55	72	75	99

a Conditions: **1a** (0.1 M), ClCOOBn (3.5 eq.), **OBn-DPI** (20 mol%), DIPEA (4.0 eq.), toluene (2 mL), unless otherwise noted.

b Yields were calculated from ^1^H NMR spectra.

c The ee values were calculated from HPLC spectra.

d ClCOOBn (3.0 eq.).

e ClCOOBn (2.5 eq.).

**Scheme 2:**
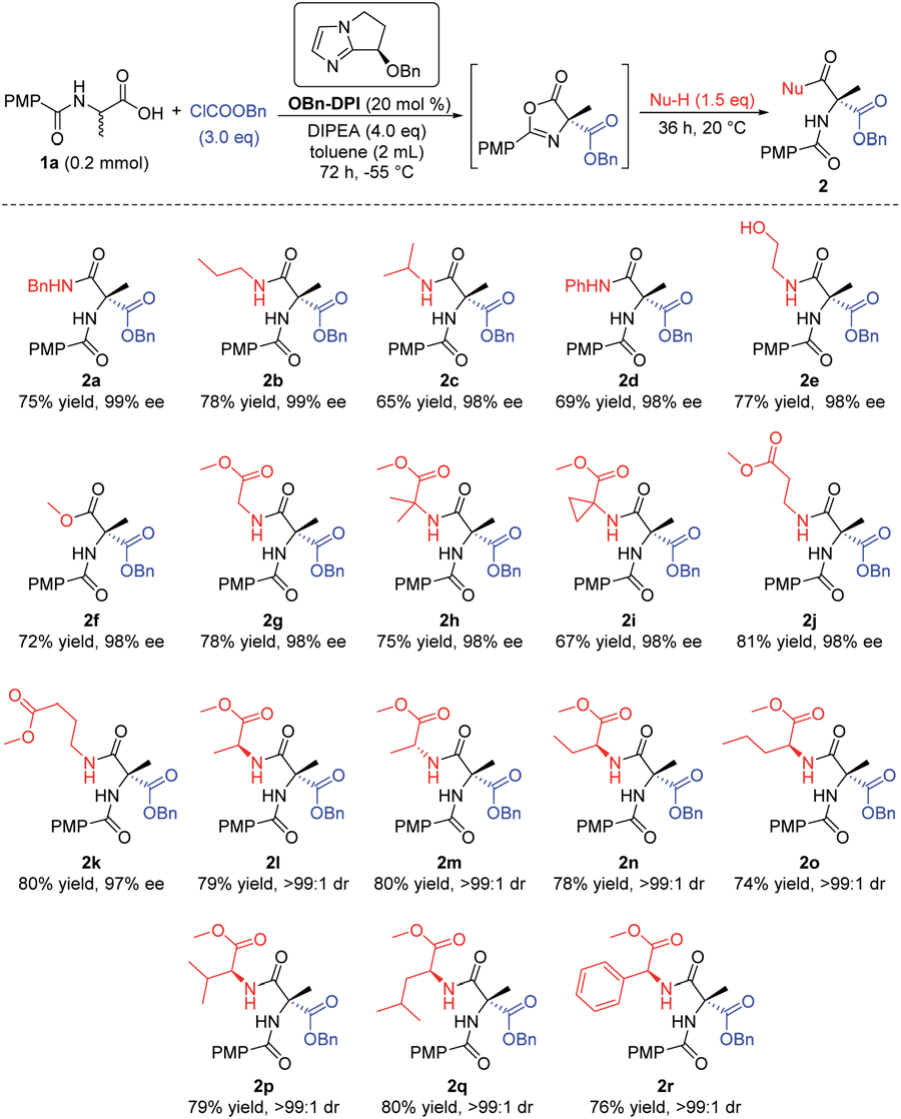
Expanding the nucleophiles. All the yields are isolated yields. ^1^H NMR spectroscopy of the crude reaction products was used to assess diastereoisomeric ratios (dr).

Having established the optimal reaction conditions, we investigated the nucleophile in the last step of the sequential process, employing (4-methoxybenzoyl)alanine **1a** as the initial substrate (Scheme 2). Firstly a number of different alkyl amines and a phenyl amine were tested as nucleophiles (**2a–d**). Amines with less steric hindrance showed higher yields (**2a**, **b***vs.***2c**, **d**). Cholamine, bearing both amino and hydroxyl groups, was also used as the nucleophile. Due to the wide difference in the activity of the alcohols and amines for this reaction, only the product **2e** was obtained, *via* attack of the amino group, with good yield and excellent enantioselectivity (98% ee). Secondly, excess methanol was employed as a nucleophile in this reaction to give the corresponding product **2f** with similar results. In addition, a variety of amino acid esters gave their corresponding enantiomerically pure dipeptides, which will be of particular use in the fields of biology and medicine (**2g–r**). When using an enantiomerically pure amino acid ester which contained a chiral center, the corresponding dipeptide products bearing two stereocenters were obtained with high dr (>99 : 1, **2l–r**).

**Scheme 3:**
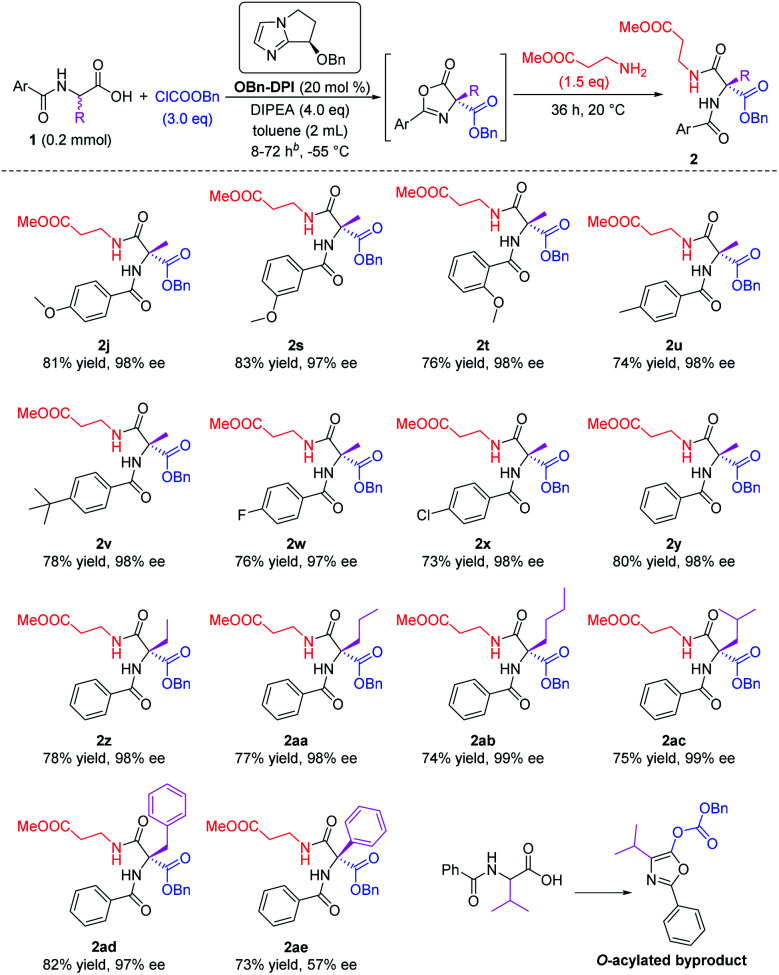
Expanding the racemic amino acid substrates. All the yields are isolated yields. Reaction time for **2s–v** is 72 h, reaction time for **2w–x** is 8 h, reaction time for **2y–ad** is 10 h.

Various *N*-protected α-amino acid substrates **1** were tested in the sequential process using methyl 3-aminopropanoate as the nucleophile (Scheme 3). Firstly, *N*-substituted amino acid substrates bearing a methoxy group at the *para*-, *meta*-, and *ortho*-positions of the phenyl ring were tested in the domino reaction (**2j**, **2s** and **2t**). All these products were obtained in good yields and with excellent enantioselectivities. The effect of different substituents at the *para*-position of the phenyl ring was studied (**2u–x**). Substrates bearing electron-donating groups such as Me or *t*Bu, and electron-withdrawing groups such as F or Cl, all afforded the corresponding products in good yields and with excellent enantioselectivities. The presence of an electron-withdrawing group enhances the acidity of the substrate and its corresponding intermediates in the two *O*-acylation steps and the *C*-acylation step (see proposed mechanism below), thus reducing the reaction time of these substrates to 8 h, much shorter than the reaction time for substrates bearing electron-donating groups (72 h). A substrate without a substituent group on the phenyl ring also gave the corresponding product **2y** in 80% yield and with 98% ee within 10 h. We then employed simple *N*-benzoyl amino acids to study the influence of the R group on the stereocenter (**2y–ae**). The substrate bearing a phenyl substituent gave the corresponding product **2ae** with only 57% ee, and the substrate bearing an isopropyl group only gave the *O*-acylated byproduct. All other tested substrates gave the corresponding products in good yields and with excellent enantioselectivities. It's worth mentioning that products **2ab** and **2ac** were both obtained with 99% ee.

**Scheme 4:**
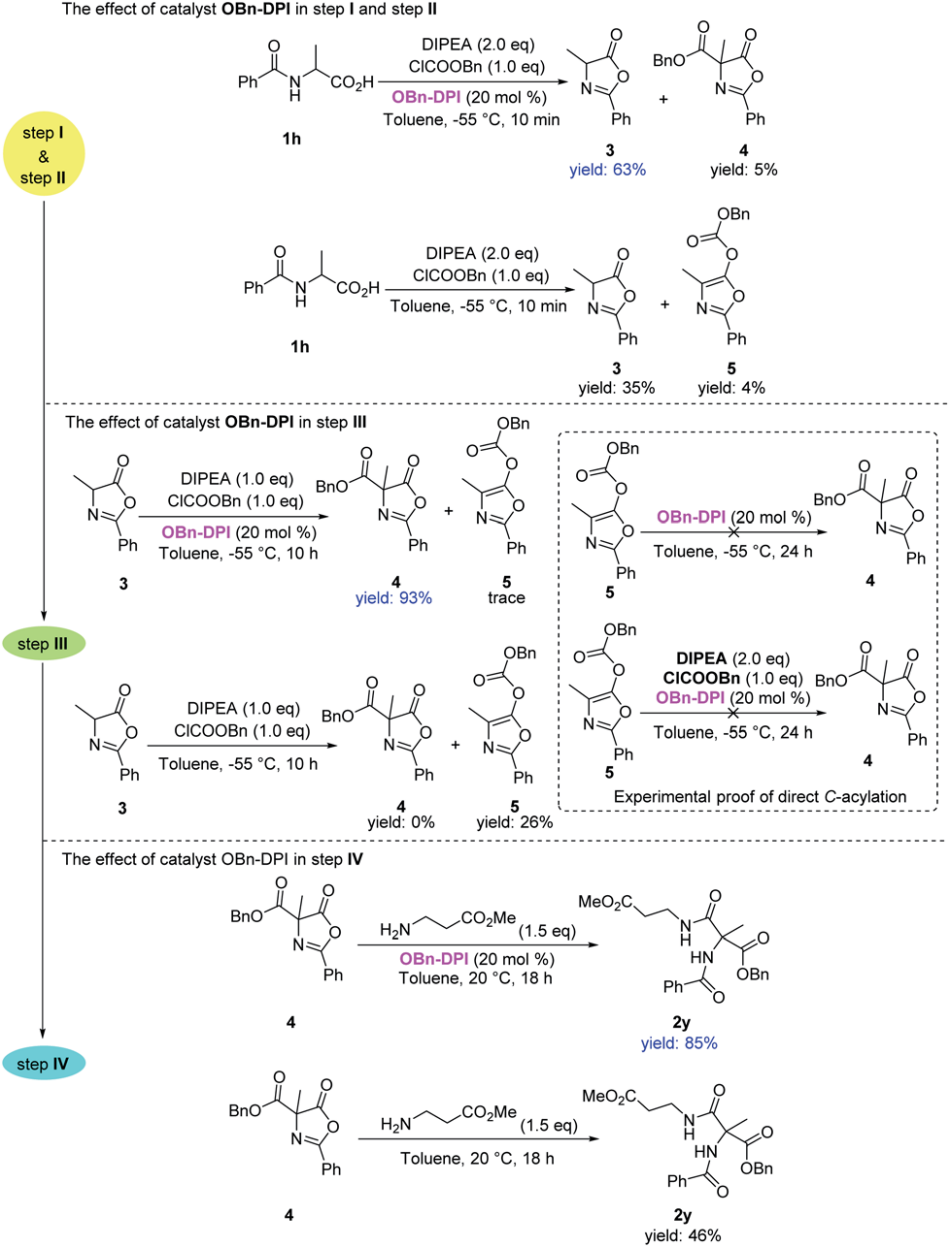
Experimental studies on the effect of catalyst **OBn-DPI** and direct *C*-acylation. Yields were calculated from ^1^H NMR spectra.

Having explored the substrate scope of both nucleophiles and amino acids, we focused on elucidating the effect of the catalyst **OBn-DPI** in each step of the acylation sequence (Scheme 4). Firstly, benzoylalanine **1h** was reacted with 1.0 equivalent of benzyl chloroformate in the presence or absence of catalyst **OBn-DPI** to test the effect of the catalyst in the first two *O*-acylation steps (step **I** and step **II**). In the presence of the catalyst, **1h** afforded the cyclized product **3** in 63% yield at −55 °C in 10 min; however in the absence of catalyst, **1h** only gave the desired product **3** in 35% yield. We next subjected **3** to reaction with benzyl chloroformate in the presence or absence of catalyst **OBn-DPI** to test effect of the catalyst in the third *C*-acylation step (step **III**). In the presence of the catalyst, **3** afforded *C*-acylated product **4** in 93% yield in 10 h, whereas in the absence of catalyst, **3** was unable to afford *C*-acylated product **4** giving *O*-acylated compound **5** in 26% yield. Compound **5** was then employed as a reactant at −55 °C in the presence of the catalyst **OBn-DPI**; no rearrangement product **4** was observed after 24 h. These experimental results strongly support that **3** undergoes a direct *C*-acylation to afford **4**. Finally, **4** was reacted with an amino acid ester in order to test the effect of the catalyst in the last step (step **IV**). In the presence of the catalyst, **4** afforded final product **2y** in 85% yield at 20 °C in 18 h, whereas **2y** was only obtained in 46% yield in the absence of catalyst.

**Scheme 5:**
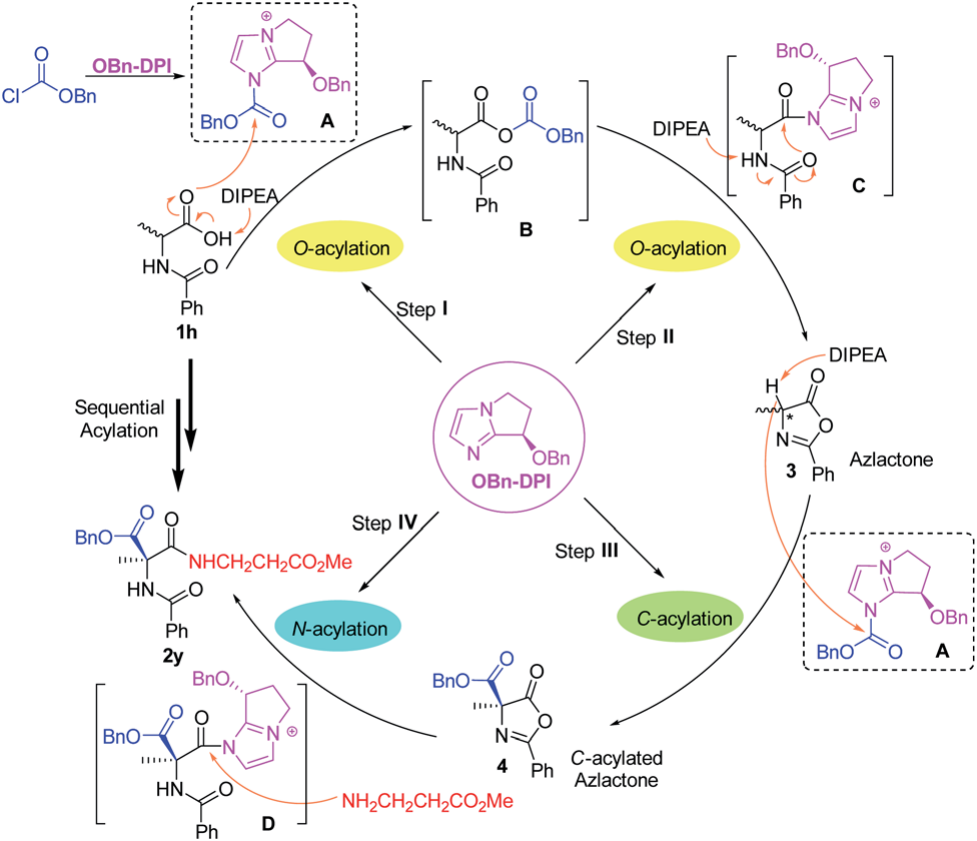
Proposed mechanism for the four-step acylation sequence *via* auto-tandem catalysis (the anion is omitted in **A**, **B**, **C**, and **D** for clarity).

Based on these results, a mechanism for the four-step acylation sequence has been proposed (Scheme 5). First, the catalyst **OBn-DPI** and benzyl chloroformate generate active species **A**. In the presence of DIPEA, *N*-substituted amino acid substrates **1**, such as **1h**, attack **A** to generate the intermediate mixed anhydride **B**. Reaction of **B** and **OBn-DPI** gives intermediate **C** which generates azlactone **3***via* an intramolecular cyclization. In step **III**, azlactone **3** attacks the active species **A** to form *C*-acylated azlactone **4***via* an enantioselective direct *C*-acylation. This chiral compound **4** and catalyst **OBn-DPI** form active intermediate **D**, which is attacked by the amino acid ester to further generate valuable dipeptide **2y***via N*-acylation. Experimental studies suggest that **OBn-DPI** acts as a nucleophilic catalyst in all the steps of the acylation sequence with compound **4** being formed from direct *C*-acylation.

**Scheme 6:**
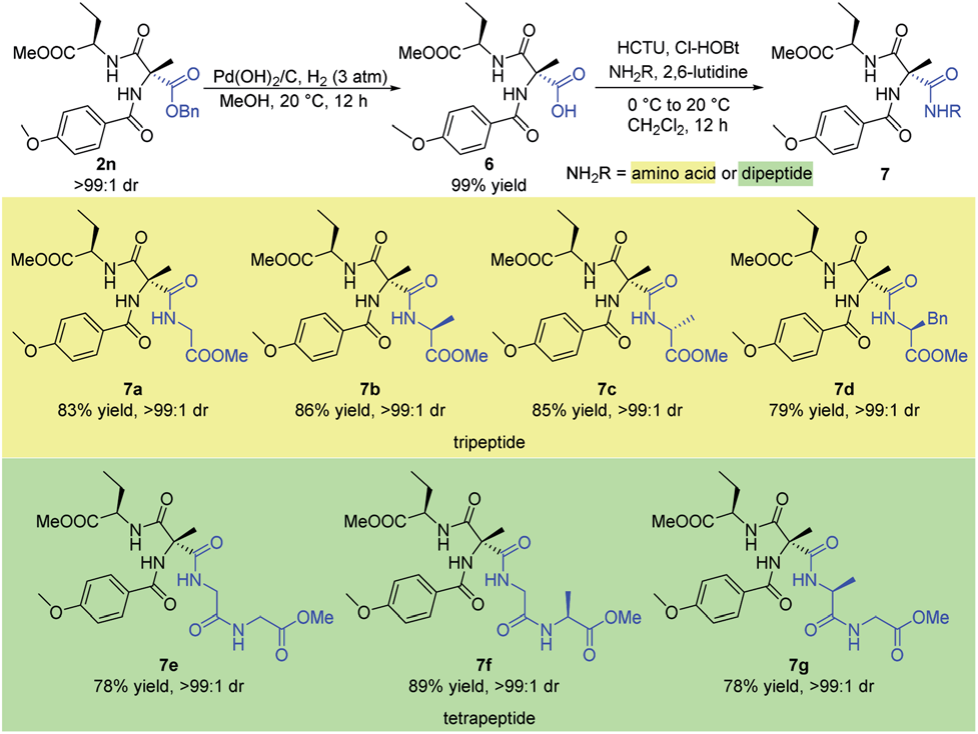
Synthesis of small peptide. All the yields are isolated yields. ^1^H NMR spectroscopy of the crude reaction products was used to assess diastereoisomeric ratios (dr).

Small peptides play a significant role in many biological, pharmaceutical, and environmental applications.^16^ Therefore the stereoselective synthesis of such biomolecules is of great importance. Here, we describe the concise synthesis of small optically active peptides from the sequentially acylated product **2n** (Scheme 6). Firstly, C^α^-tetrasubstituted α-amino acid **6** could be obtained from hydrogenation of **2n** under 3 atm hydrogen pressure with Pd(OH)_2_/C in quantitative yield. Then, coupling of **6** with several different amino acids or dipeptides afforded a variety of valuable small chiral peptides in good yields (**7a–g**). In particular, coupling of **6** with enantiomerically pure amino acids or dipeptides which contained a chiral center afforded products bearing three stereocenters with high dr (**7b–d**, **7f–g**).

**Scheme 7:**
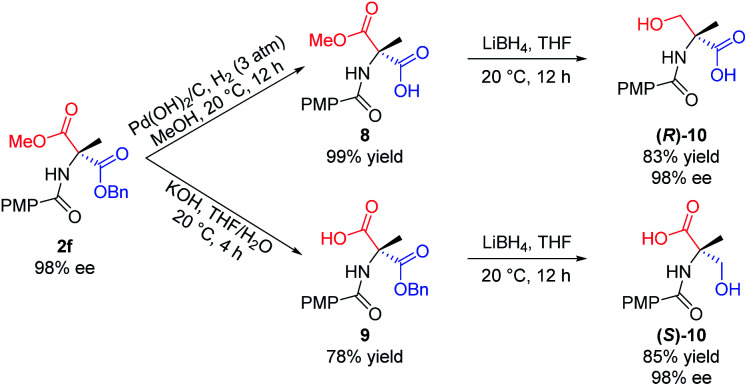
Enantiodivergent synthesis of α-methyl serine. All the yields are isolated yields.

Furthermore, the utility of this acylation sequence was demonstrated *via* the enantiodivergent synthesis of α-methyl serine from **2f** by manipulating the different reactivities of the two ester groups (Scheme 7). Hydrogenation of the benzyl ester group of **2f** with Pd(OH)_2_/C in MeOH at 20 °C for 12 h provided **8** in quantitative yield. Reduction of **8** with LiBH_4_ in THF at ambient temperature for 12 h afforded the (*R*)-isomer of *N*-protected α-methyl serine, **(R)-10**, in 83% yield. Hydrolysis of the methyl ester group of **2f** with KOH in THF/H_2_O at 20 °C provided **9** in 78% yield, which was reduced with LiBH_4_ to give the (*S*)-isomer of *N*-protected α-methyl serine, **(S)-10**, in 85% yield.

**Scheme 8:**
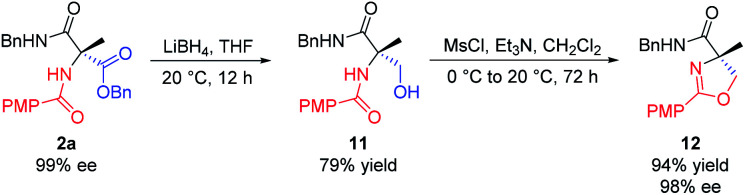
Synthesis of 4,4′-disubstituted oxazoline. All the yields are isolated yields.

In addition, we realized the synthesis of 4,4′-disubstituted oxazoline from **2a**, as shown in Scheme 8. Reduction of **2a** with LiBH_4_ in THF provided the corresponding alcohol **11**. Mesylation of **11** with methanesulfonyl chloride in DCM at 0 °C in the presence of triethylamine, followed by intramolecular cyclization at 20 °C in one pot, finally afforded oxazoline **12** in 94% yield (74% overall from **2a**). This method allows for the practical synthesis of chiral oxazolines, which can be used as chiral ligands for asymmetric catalysis.^17^

## Experimental

Under a N_2_ atmosphere, the substrate **1h** (0.2 mmol, 44.7 mg), the catalyst **OBn-DPI** (0.04 mmol, 8.6 mg) and DIPEA (0.8 mmol, 132.2 μL) were dissolved in anhydrous toluene (2 mL) and cooled to −55 °C in a dry two-necked flask. ClCOOallyl (0.6 mmol, 84.5 μL) was then added and the vial was sealed with a septum. The reaction mixture was stirred at −55 °C for 10 h and then the temperature was gradually raised to 20 °C. Methyl 3-aminopropionate hydrochloride (0.3 mmol, 41.9 mg) and DIPEA (0.3 mmol, 49.6 μL) were dissolved in anhydrous toluene (1 mL) in another dry flask and stirred for 10 min. Then the mixture was transferred into the former reaction flask and the reaction mixture was stirred at 20 °C for 36 h. The reaction mixture was quenched with 0.2 M HCl (5 mL) and extracted with DCM (5 mL × 3). The combined organic phases were dried over Na_2_SO_4_. After filtration, the residue was purified by column chromatography (petroleum ether/ethyl acetate) to give the corresponding product **2y**. The ee value was determined by chiral HPLC analysis after purification by column chromatography (petroleum ether/ethyl acetate).

## Conclusions

In summary, the first four-step sequential acylation reaction for the concise asymmetric synthesis of C^α^-tetrasubstituted α-amino acid derivatives *via* auto-tandem catalysis has been successfully developed. This step-economic, one-pot, and auto-tandem strategy is promoted by a direct enantioselective *C*-acylation. Through four acylations catalyzed by a single chiral bicyclic imidazole, the simple *N*-acylated amino acids could be smoothly converted to the corresponding C^α^-tetrasubstituted α-amino acid derivatives with excellent enantioselectivities (up to 99% ee). Significantly, the obtained products, particularly dipeptides, are potentially biologically active compounds. These products can be further transformed to other biomolecules and important chiral building blocks such as small peptides, α-substituted serines and 4,4′-disubstituted oxazolines.

## Conflicts of interest

The authors declare that they have no conflict of interest.

## Supplementary Material

SC-011-D0SC00808G-s001
