# Supplementary material for: A step-economic and one-pot access to chiral Cα-tetrasubstituted α-amino acid derivatives via a bicyclic imidazole-catalyzed direct enantioselective C-acylation
Source: Chem Sci. 2020 Apr 24;11(18):4801–7. doi: 10.1039/d0sc00808g (PMC8159231; doi:10.1039/d0sc00808g)
Supplement: SC-011-D0SC00808G-s001 [file SC-011-D0SC00808G-s001.pdf]

Electronic Supplementary Material (ESI) for Chemical Science.
This journal is © The Royal Society of Chemistry 2020

Supporting Information

A step-economic and one-pot access to chiral C^{α}-tetrasubstituted α-amino acid derivatives via a bicyclic imidazole-catalyzed direct enantioselective C-acylation

Mo Wang,[1,2] Muxing Zhou,[2] Lu Zhang,[2] Zhenfeng Zhang,[2*] and Wanbin Zhang[1,2*]

[1]*Shanghai Key Laboratory for Molecular Engineering of Chiral Drugs, School of Chemistry and Chemical Engineering, Frontiers Science Center for Transformative Molecules, Shanghai Jiao Tong University, Shanghai 200240, China.*

[2]*School of Pharmacy, Shanghai Jiao Tong University, Shanghai 200240, China.*

[*]Email: wanbin@sjtu.edu.cn zhenfeng@sjtu.edu.cn

Table of Contents

1. General Details S2
2. Preparation of Catalysts S3
3. Preparation of Substrates S4
4. Optimization of Conditions S8
5. Asymmetric Reactions S9
6. Effect of N-Acylated Catalyst S27
7. Synthetic Applications S28
8. Reference S36
9. NMR Spectra S37
10. HPLC Charts S81

1. General Details

All air- and moisture-sensitive manipulations were carried out under a nitrogen atmosphere. The solvents used in the reactions were distilled under nitrogen after dehydration. All other chemicals and solvents were purchased from commercial company and used as received. The NMR spectra were recorded on a Bruker Avance III HD (400 MHz, ^{1}H; 100 MHz, ^{13}C) spectrometer with chemical shifts reported in ppm relative to the residual deuterated solvents or the internal standard tetramethylsilane. Mass spectrometry analysis was carried out using a Waters Micromass Q-TOF Premier Mass Spectrometer at the Instrumental Analysis Center of Shanghai Jiao Tong University. Melting points were measured with SGW X-4 micro melting point apparatus. Optical rotations were measured on a Rudolph Research Analytical Autopol VI automatic polarimeter using a 50 mm path-length cell at 589 nm. Chiral HPLC analyses were performed using a Shimadzu LC-10Avp system using isopropanol-hexane mobile phase and UV detection.

2. Preparation of Catalysts

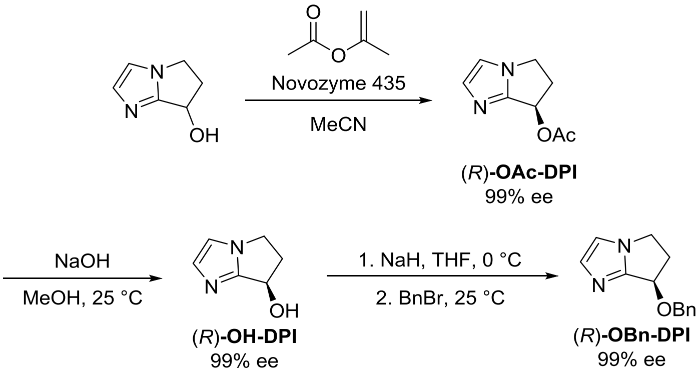

(*R*)-7-Benzyloxy-6,7-dihydro-5*H*-pyrrolo[1,2-a]imidazole ((*R*)-OBn-DPI)[1-4]

A solution of racemic 6,7-dihydro-5*H*-pyrrolo[1,2-*a*]imidazol-7-ol (620.7 mg, 5 mmol,), isopropenyl acetate (0.65mL, 6 mmol) and NOV435 (620.7 mg) in 3.0 mL acetonitrile was stirred gently at 35 ℃ for 3 h in a 10 mL two-necked flask. The reaction mixture was filtrated and MeCN was removed. The residue was purified by column chromatography (EtOAc/MeOH = 30/1, Rf = 0.38) to give (*R*)-**OAc-DPI** (340.7 mg, 41% yield) as a colorless oil. **HPLC analysis:** 99.9% ee [Daicel CHIRALPAK OJ column; solvent system: 10% isopropanol/90% hexane; 0.5 mL/min; retention times: 39.0 min (major), 41.6 min (minor)].

A solution of (*R*)-**OAc-DPI** (830.9 mg, 5 mmol) and NaOH (300.0 mg, 7.5 mmol) in 30 mL MeOH was stirred gently at 25 ℃ for 3 h in a 100 mL two-necked flask. The reaction mixture was filtrated and MeOH was removed. The residue was purified by column chromatography (EtOAc/MeOH = 4/1, Rf = 0.30) to give (*R*)-**OH-DPI** (505.0 mg, 81% yield) as a white solid. **HPLC analysis:** 99.9% ee [Daicel CHIRALPAK OJ column; solvent system: 10% isopropanol/90% hexane; 0.5 mL/min; retention times: 39.0 min (major), 41.6 min (minor)].

In a dry two-necked flask, a solution of (*R*)-**OH-DPI** (100.0 mg, 0.8 mmol) in THF (15 mL) was treated with NaH (48.0 mg, 1.2 mmol) for 2 h at 0 ℃, then BnBr (0.15 mL, 1.3 mmol) was added dropwise and stirred for 16 h at 25 ℃. The solvent was acidized with 1 M HCl (10 mL) then basified with 2 M NaOH and extracted with EtOAc (30 mL × 3). The crude product was purified by flash chromatography on silica gel (EtOAc/MeOH = 20/1, Rf = 0.38) to give 125.3 mg of (*R*)-**OBn-DPI** as yellow oil (73% yield). **HPLC analysis:** 99.9% ee [Daicel CHIRALPAK IE column; solvent system: 30% isopropanol/70% hexane; 0.5 mL/min; retention times: 18.7 min (major)]. **$[\alpha]_D^{14}$** = 55.0 (*c* 0.13, MeOH). **^{1}H NMR ($CDCl_3$, 400 MHz):** *δ* 7.41-7.27 (m, 5 H), 7.16 (d, *J* = 1.2 Hz, 1 H), 6.93 (d, *J* = 1.2 Hz, 1 H), 4.83 (dd, *J* = 7.2 Hz, 2.0 Hz, 1 H), 4.81 (dd, *J* = 67.6 Hz, 11.6 Hz, 2 H), 4.21-4.13 (m, 1 H), 3.96-3.89 (m, 1 H), 2.92-2.82 (m, 1 H), 2.67-2.59 (m, 1 H). **^{13}C NMR ($CDCl_3$, 100 MHz):** *δ* 153.5, 137.9, 133.8, 128.4, 128.1, 127.7, 115.0, 71.1, 70.8, 43.1, 35.3. **HRMS (ESI):** calcd. for $C_{13}H_{15}N_2O$ $(M+H)^+$ 215.1179, found 215.1184.

3. Preparation of Substrates

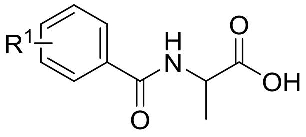

1a~1h

1a: R^1 = 4-MeO
1b: R^1 = 3-MeO
1c: R^1 = 2-MeO
1d: R^1 = 4-Me
1e: R^1 = 4-*t*Bu
1f: R^1 = 4-F
1g: R^1 = 4-Cl
1h: R^1 = None

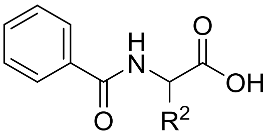

1i~1n

1i: R^2 = Et
1j: R^2 = *n*Pr
1k: R^2 = *n*Bu
1l: R^2 = *i*Bu
1m: R^2 = Bn
1n: R^2 = Ph

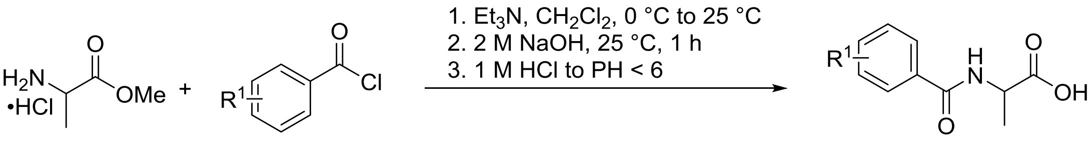

1

General procedure A:[5]

(DL)-Alanine methyl ester hydrochloride (10 mmol) were dissolved in CH_2Cl_2 (50 mL) in a flask and Et_3N (25 mmol) were added. The resulting slurry was cooled to 0 ℃, and the corresponding benzoyl chloride (10 mmol) in CH_2Cl_2 (10 mL) was added by cannula over 15 minutes. After 75 minutes, the ice bath was removed, and the mixture was stirred at room temperature for 6 hours. The mixture was then washed with 1 M HCI (30 mL × 2), saturated $NaHCO_3$ (30 mL × 2), and saturated NaCl (30 mL). The CH_2Cl_2 layer was dried over $MgSO_4$, and the solvent was removed by rotary evaporation, providing the *N*-acyl-(DL)-alanine methyl ester as a white solid. This solid was dissolved in methanol (30 mL), and 2 M aqueous NaOH (6 mL) was added. The resulting mixture was stirred for 20 minutes, and then the methanol was removed by rotary evaporation. Water was added until the aqueous solution was homogeneous, and then the aqueous solution was washed with CH_2Cl_2 (20 mL × 2). The aqueous layer was made acidic with 1M HC1, giving a white precipitate, which was filtered, washed with several portions of water, and dried with a flow of air through a filter.

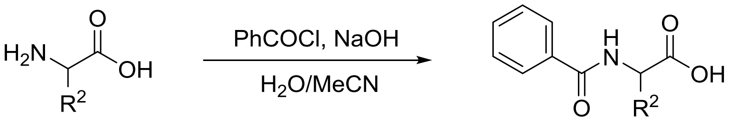

1

General procedure B:[6]

The corresponding racemic amino acid (10 mmol) and NaOH (40 mmol) were dissolved in H_2O/CH_3CN (75/25, 50 mL). After cooling to 0 ℃, benzoyl chloride (11 mmol) was added dropwise at this temperature. After the addition was complete, the mixture was stirred for additional 2 h at 0 ℃. Subsequently, the mixture was allowed to warm to room temperature and was stirred for one additional hour. All volatiles were then removed under reduced pressure before conc. HCl was added to cause

precipitation. The mixture was filtered and the filter cake was washed with ice-cold diethyl ether and dried with a flow of air through a filter.

Substrates **1a-1g** were prepared via general procedure A, while substrates **1i-1m** were prepared via general procedure B according to the literature's methods. Substrates **1b**, **1h**, **1l** and **1n** were commercially available. The following substrates **1a-1m** are known compounds, and their characterization data were in agreement with reported values.[5-9]

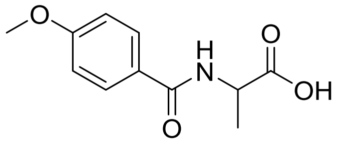

1a

(4-Methoxybenzoyl)alanine (1a)[5]

^{1}H NMR (DMSO, 400 MHz): δ 12.46 (br s, 1H), 8.49 (d, *J* = 7.0 Hz, 1H), 7.87 (d, *J* = 8.8 Hz, 2H), 7.00 (d, *J* = 8.8 Hz, 2H), 4.43 – 4.34 (m, 1H), 3.81 (s, 3H), 1.38 (d, *J* = 7.2 Hz, 3H).

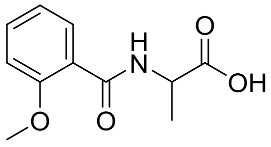

1c

(2-Methoxybenzoyl)alanine (1c)[7]

^{1}H NMR ($CDCl_3$, 400 MHz): δ 10.67 (s, 1H), 8.81 (d, *J* = 6.6 Hz, 1H), 8.17 (dd, *J* = 7.8, 1.8 Hz, 1H), 7.50 – 7.39 (m, 1H), 7.11 – 6.90 (m, 2H), 4.87 – 4.78 (m, 1H), 3.95 (s, 3H), 1.58 (d, *J* = 7.2 Hz, 3H).

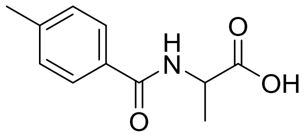

1d

(4-Methylbenzoyl)alanine (1d)[8]

^{1}H NMR (DMSO, 400 MHz): δ 8.58 (d, *J* = 7.2 Hz, 1H), 7.77 (d, *J* = 7.8 Hz, 2H), 7.26 (d, *J* = 7.8 Hz, 2H), 4.39 – 4.36 (m, 1H), 2.34 (s, 3H), 1.37 (d, *J* = 7.2 Hz, 3H).

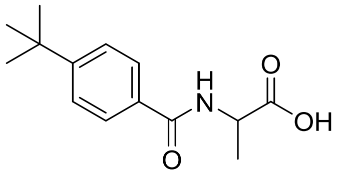

1e

(4-(*tert*-Butyl)benzoyl)alanine (1e)[8]

^{1}H NMR (DMSO, 400 MHz): δ 12.59 (br s, 1H), 8.57 (d, *J* = 7.2 Hz, 1H), 7.81 (d, *J* = 8.2 Hz, 2H), 7.48 (d, *J* = 8.2 Hz, 2H), 4.46 – 4.35 (m, 1H), 1.38 (d, *J* = 7.4 Hz, 3H), 1.30 (s, 9H).

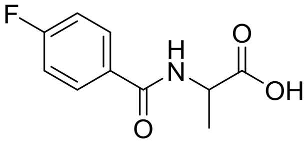

1f

(4-Fluorobenzoyl)alanine (1f)[8]

^{1}H NMR ($CDCl_3$, 400 MHz): δ 7.84 – 7.81 (m, 2H), 7.16 – 7.12 (m, 2H), 6.61 (d, *J* = 4.0 Hz, 1H), 4.82 – 4.77 (m, 1H), 1.59 (d, *J* = 4.8 Hz, 3H).

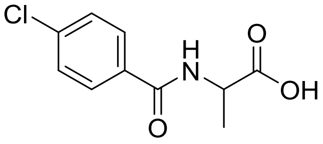

1g

(4-Chlorobenzoyl)alanine (1g)[8]

^{1}H NMR (DMSO, 400 MHz): δ 8.74 (s, 1H), 7.88 (d, *J* = 8.2 Hz, 2H), 7.54 (d, *J* = 8.2 Hz, 2H), 4.39 – 4.33 (m, 1H), 1.37 (d, *J* = 7.2 Hz, 3H).

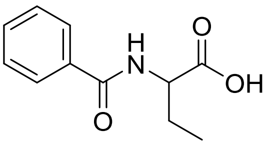

1i

2-Benzamidobutanoic acid (1i)[9]

^{1}H NMR (DMSO, 400 MHz): δ 12.59 (br s, 1H), 8.54 (d, *J* = 7.2 Hz, 1H), 7.89 (d, *J* = 7.2 Hz, 2H), 7.57 – 7.43 (m, 3H), 4.33 – 4.25 (m, 1H), 1.92 – 1.71 (m, 2H), 0.95 (t, *J* = 7.4 Hz, 3H).

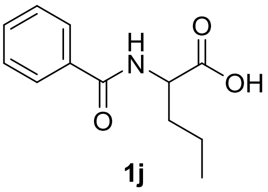

1j

2-Benzamidopentanoic acid (1j)[9]

^{1}H NMR (DMSO, 400 MHz): δ 12.53 (br s, 1H), 8.56 (d, *J* = 7.6 Hz, 1H), 7.88 (d, *J* = 7.4 Hz, 2H), 7.57 – 7.42 (m, 3H), 4.42 – 4.33 (m, 1H), 1.82 – 1.72 (m, 2H), 1.49 – 1.30 (m, 2H), 0.90 (t, *J* = 7.2 Hz, 3H).

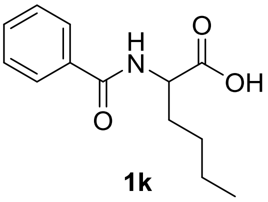

1k

2-Benzamidohexanoic acid (1k)[9]

^{1}H NMR (DMSO, 400 MHz): δ 12.56 (br s, 1H), 8.57 (d, *J* = 7.6 Hz, 1H), 7.93 – 7.85 (m, 2H), 7.58 – 7.43 (m, 3H), 4.39 – 4.31 (m, 1H), 1.87 – 1.70 (m, 2H), 1.46 – 1.24 (m, 4H), 0.87 (t, *J* = 7.0 Hz, 3H).

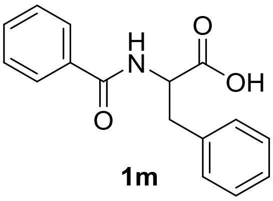

1m

Benzoylphenylalanine (1m)[6]

^{1}H NMR (DMSO, 400 MHz): δ 8.69 (d, *J* = 6.2 Hz, 1H), 7.83 – 7.72 (m, 2H), 7.56 – 7.39 (m, 3H), 7.35 – 7.10 (m, 5H), 4.65 – 4.57 (m, 1H), 3.21 – 3.03 (m, 2H).
The other analytical data are in accordance with the literature.

4. Optimization of Conditions

Table S1. The Effect of the Usage of Each Components

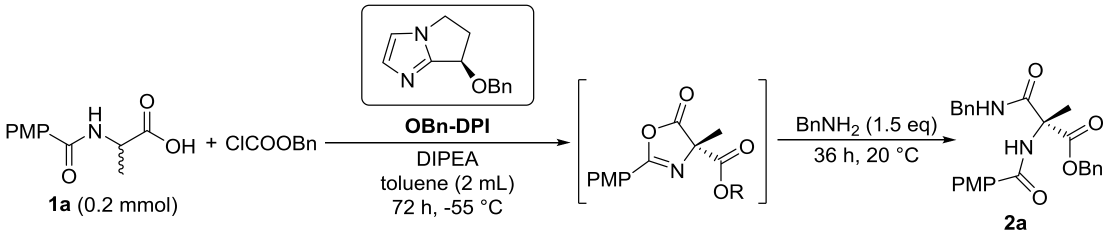

entry [a)]	loading of ClCOOBn (eq)	loading of DIPEA (eq)	yield (%) [b)]	ee (%) [c)]
1	3.5	4.0	78	99
2	2.5	4.0	75	99
3	3.0	4.0	78	99
4	4.0	4.0	76	98
5	3.0	3.5	76	99
6	3.0	4.5	73	98
7 [d)]	3.0	4.0	56	99

a) **1a** (0.1 M), **OBn-DPI** (20 mol %), toluene (2 mL), −55 ℃, 72 h, unless otherwise noted. b) Yields were calculated from ^{1}H NMR spectra. c) The ee values were calculated from HPLC spectra. d) **OBn-DPI** (10 mol %).

The effect of reagent equivalents was investigated (Table S1). Changing the usage of benzyl chloroformate has almost no effect on results (entries 1-4). Only when the usage was increased to 4.0 equivalents, the ee value of product was slightly decreased to 98% (entry 4). Decreasing the usage of DIPEA to 3.5 equivalents will slightly reduce the yield (entry 5), while increasing the usage to 4.5 equivalents decreased the ee value to 98% (entry 6). Decreasing the loading of catalyst to 10 mol % dramatically reduced the yield, though the enantioselectivity was remained at 99% ee (entry 7).

5. Asymmetric Reactions

Different Nucleophiles:

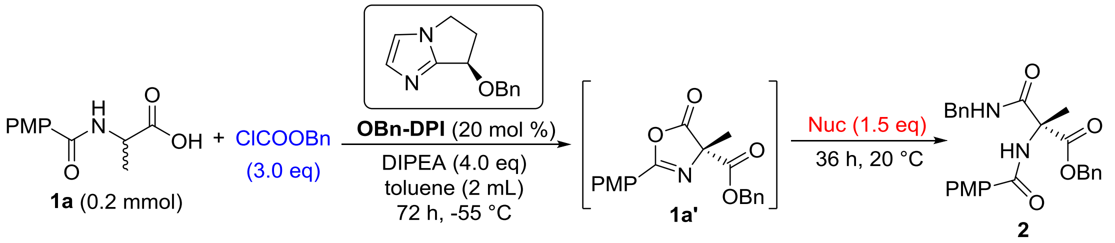

The steps to 1a': Under a N_2 atmosphere, the substrate **1a** (0.2 mmol, 44.7 mg), the catalyst **OBn-DPI** (0.04 mmol, 8.6 mg) and DIPEA (0.8 mmol, 132.2 μL) were dissolved in anhydrous toluene (2 mL) and cooled to −55 ℃ in a dry two-necked flask. ClCOOallyl (0.6 mmol, 84.5 μL) was then added and the vial was sealed with a septum. The reaction mixture was stirred at −55 ℃ for 72 h and then the temperature was gradually raised to 20 ℃.

The step to 2a~2e: Following the steps to **1a'**, the corresponding amines (0.3 mmol) was added to the vial and the reaction mixture was was stirred at 20 ℃ for 36 h.

The step to 2f: Following the steps to **1a'**, 1 mL methanol was added to the vial and the reaction mixture was stirred at 20 ℃ for 36 h.

The step to 2g~2r: Following the steps to **1a'**, the corresponding amino acid methyl ester hydrochloride (0.3 mmol) and DIPEA (0.3 mmol, 49.6 μL) were dissolved in anhydrous toluene (1 mL) in another dry flask and stirred for 10 min. Then the mixture was transferred into the former reaction flask and the reaction mixture was stirred at 20 ℃ for 36 h.

The reaction mixture was quenched with 0.2 M HCl (5 mL) and extracted with DCM (5 mL × 3). The combined organic phases were dried over Na_2SO_4. After filtration, the residue was purified by column chromatography (petroleum ether/ethyl acetate) to give the corresponding product **2**.

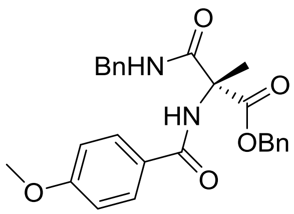

2a

Benzyl (*R*)-3-(benzylamino)-2-(4-methoxybenzamido)-2-methyl-3-oxopropanoate (2a)

Purification by flash chromatography (petroleum ether/ethyl acetate = 3/1) afforded the product as a white solid (66.9 mg, 75% yield). **M.p.:** 130.6-131.6 ℃. **HPLC analysis:** 99% ee [Daicel CHIRALPAK OD-H column; solvent system: 20% isopropanol/80% hexane; 0.5 mL/min; retention times: 28.8 min (minor), 33.2 min (major)]. **$[\alpha]^{25}_D$** = 4.9 (*c* 0.55, CH_2Cl_2). **^{1}H NMR ($CDCl_3$, 400 MHz):** δ 7.82 – 7.77 (m, 2H), 7.74 (s, 1H), 7.33 – 7.22 (m, 8H), 7.18 – 7.12 (m, 2H), 6.96 – 6.90 (m, 2H), 6.55 (t, *J* = 5.2 Hz, 1H), 5.19 (s, 2H), 4.45 (dd, *J* = 14.8, 5.6 Hz, 1H), 4.36 (dd, *J* =

14.8, 5.6 Hz, 1H), 3.86 (s, 3H), 1.92 (s, 3H). **^{13}C NMR ($CDCl_3$, 100 MHz):** δ 170.7, 167.9, 165.8, 162.5, 137.1, 135.0, 129.0, 128.8, 128.5, 128.3, 128.1, 127.7, 127.4, 125.8, 113.7, 68.1, 63.1, 55.4, 44.1, 22.3. **IR (thin film):** ν 3715, 3647, 3482, 2923, 2848, 1743, 1646, 1486, 1384, 1258, 1179, 1118, 1029, 845, 697 cm^{-1}. **HRMS (ESI):** calcd. for $C_{26}H_{26}N_2O_5$ $(M+H)^+$ 447.1914, found 447.1913.

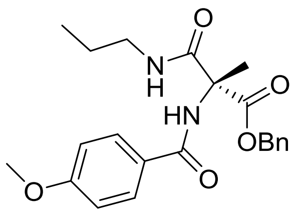

2b

Benzyl (*R*)-2-(4-methoxybenzamido)-2-methyl-3-oxo-3-(propylamino)propanoate (2b)

Purification by flash chromatography (petroleum ether/ethyl acetate = 2/1) afforded the product as a colorless oil (62.0 mg, 78% yield). **HPLC analysis:** 99% ee [Daicel CHIRALPAK OD-H column; solvent system: 35% isopropanol/65% hexane; 0.3 mL/min; retention times: 19.0 min (major), 21.2 min (minor)]. **$[\alpha]^{25}_D$** = 12.8 (*c* 0.55, CH_2Cl_2). **^{1}H NMR ($CDCl_3$, 400 MHz):** δ 7.83 – 7.76 (m, 3H), 7.28 (s, 5H), 6.95 – 6.90 (m, 2H), 6.17 (t, *J* = 5.2 Hz, 1H), 5.22 (d, *J* = 12.0 Hz, 1H), 5.18 (d, *J* = 12.0 Hz, 1H), 3.85 (s, 3H), 3.25 – 3.10 (m, 2H), 1.90 (s, 3H), 1.47 – 1.37 (m, 2H), 0.84 (t, *J* = 7.4 Hz, 3H). **^{13}C NMR ($CDCl_3$, 100 MHz):** δ 170.8, 167.8, 165.7, 162.4, 135.1, 129.0, 128.4, 128.3, 128.2, 126.0, 113.7, 68.0, 63.0, 55.4, 41.8, 22.4, 22.4, 11.1. **IR (thin film):** ν 3736, 3481, 3420, 2963, 2923, 2850, 1740, 1646, 1607, 1536, 1483, 1384, 1257, 1178, 1118, 1030, 845, 769, 602 cm^{-1}. **HRMS (ESI):** calcd. for $C_{22}H_{26}N_2O_5$ $(M+H)^+$ 399.1914, found 399.1914.

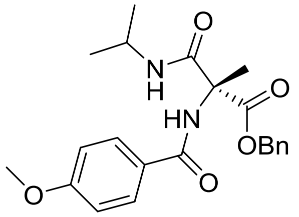

2c

Benzyl (*R*)-3-(isopropylamino)-2-(4-methoxybenzamido)-2-methyl-3-oxopropanoate (2c)

Purification by flash chromatography (petroleum ether/ethyl acetate = 3/1) afforded the product as a light yellow oil (51.5 mg, 65% yield). **HPLC analysis:** 98% ee [Daicel CHIRALPAK OD-H column; solvent system: 20% isopropanol/80% hexane; 0.1 mL/min; retention times: 92.7 min (minor), 99.6 min (major)]. **$[\alpha]^{25}_D$** = -10.4 (*c* 0.10, CH_2Cl_2). **^{1}H NMR ($CDCl_3$, 400 MHz):** δ 7.84 – 7.77 (m, 3H), 7.32 – 7.28 (m, 5H), 6.95 – 6.90 (m, 2H), 5.81 (d, *J* = 7.6 Hz, 1H), 5.21 (d, *J* = 12.0 Hz, 1H), 5.16 (d, *J* = 12.0 Hz, 1H), 4.03 – 3.92 (m, 1H), 3.86 (s, 3H), 1.88 (s, 3H), 1.11 (d, *J* = 6.4 Hz,

3H), 0.95 (d, *J* = 6.8 Hz, 3H). **^{13}C NMR ($CDCl_3$, 100 MHz):** δ 170.8, 167.0, 165.7, 162.5, 135.3, 129.0, 128.5, 128.4, 128.4, 126.0, 113.7, 67.9, 63.0, 55.4, 42.5, 22.4, 22.3, 22.0. **IR (thin film):** ν 3714, 3648, 3481, 2920, 2854, 1748, 1647, 1558, 1384, 1261, 596 cm^{-1}. **HRMS (ESI):** calcd. for $C_{22}H_{26}N_2O_5$ (M+H)$^+$ 399.1914, found 399.1914.

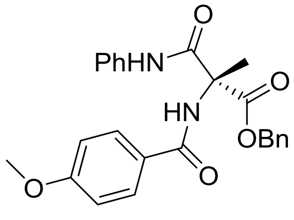

2d

Benzyl (*R*)-2-(4-methoxybenzamido)-2-methyl-3-oxo-3-(phenylamino)propanoate (2d)

Purification by flash chromatography (petroleum ether/ethyl acetate = 3/1) afforded the product as a yellow oil (59.4 mg, 69% yield). **HPLC analysis:** 98% ee [Daicel CHIRALPAK OD-H column; solvent system: 10% isopropanol/90% hexane; 0.5 mL/min; retention times: 44.8 min (minor), 49.4 min (major)]. **[α]^{25}$_D$** = -2.0 (*c* 0.40, CH_2Cl_2). **^{1}H NMR ($CDCl_3$, 400 MHz):** δ 8.42 (s, 1H), 7.83 – 7.79 (m, 2H), 7.60 (s, 1H), 7.44 – 7.22 (m, 9H), 7.16 – 7.11 (m, 1H), 6.96 – 6.93 (m, 2H), 5.26 (s, 2H), 3.86 (s, 3H), 2.00 (s, 3H). **^{13}C NMR ($CDCl_3$, 100 MHz):** δ 170.8, 166.3, 166.1, 162.7, 137.0, 134.9, 129.1, 129.0 128.5, 128.4, 128.1, 125.6, 124.9, 120.2, 113.8, 68.3, 63.9, 55.4, 22.2. **IR (thin film):** ν 3363, 3063, 3034, 2961, 2921, 2851, 1738, 1689, 1635, 1605, 1543, 1500, 1444, 1380, 1316, 1259, 1178, 1123, 1029, 845, 756, 696, 601 cm^{-1}. **HRMS (ESI):** calcd. for $C_{25}H_{25}N_2O_5$ (M+H)$^+$ 433.1758, found 433.1760.

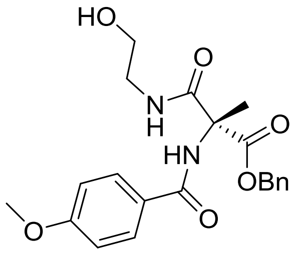

2e

Benzyl (*R*)-3-((2-hydroxyethyl)amino)-2-(4-methoxybenzamido)-2-methyl-3-oxopropanoate (2e)

Purification by flash chromatography (petroleum ether/ethyl acetate = 1/2) afforded the product as a colorless oil (61.5 mg, 77% yield). **HPLC analysis:** 98% ee [Daicel CHIRALPAK OD-H column; solvent system: 20% isopropanol/80% hexane; 0.7 mL/min; retention times: 19.0 min (major), 25.0 min (minor)]. **[α]^{25}$_D$** = 20.7 (*c* 0.45, CH_2Cl_2). **^{1}H NMR ($CDCl_3$, 400 MHz):** δ 7.83 – 7.76 (m, 2H), 7.65 (s, 1H), 7.41 – 7.30 (m, 5H), 6.98 – 6.88 (m, 2H), 6.72 (t, *J* = 5.2 Hz, 1H), 5.26 (d, *J* = 12.4 Hz, 1H), 5.19 (d, *J* = 12.4 Hz, 1H), 3.86 (s, 3H), 3.61 (t, *J* = 5.2 Hz, 2H), 3.46 – 3.27 (m, 2H),

1.89 (s, 3H). **^{13}C NMR (CDCl$_3$, 100 MHz):** δ 170.8, 168.6, 166.2, 162.7, 135.1, 129.1, 128.5, 128.5, 128.3, 125.6, 113.8, 68.1, 63.2, 61.2, 55.5, 42.7, 21.9. **IR (thin film):** ν 3481, 2960, 2923, 2851, 1744, 1643, 1607, 1537, 1485, 1384, 1259, 1177, 1121, 1029, 800 cm^{-1}. **HRMS (ESI):** calcd. for $C_{21}H_{25}N_2O_6$ $(M+H)^+$ 401.1707, found 401.1707.

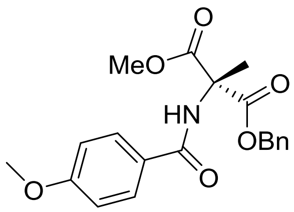

2f

1-Benzyl 3-methyl (*R*)-2-(4-methoxybenzamido)-2-methylmalonate (2f)

Purification by flash chromatography (petroleum ether/ethyl acetate = 2/1) afforded the product as a colorless oil (53.6 mg, 72% yield). **HPLC analysis:** 98% ee [Daicel CHIRALPAK OD-H column; solvent system: 20% isopropanol/80% hexane; 0.3 mL/min; retention times: 31.9 min (major), 36.9 min (minor)]. **$[\alpha]^{25}_D$** = 18.1 (*c* 0.30, CH_2Cl_2). **^{1}H NMR (CDCl$_3$, 400 MHz):** δ 7.83 – 7.73 (m, 2H), 7.45 (s, 1H), 7.37 – 7.28 (m, 5H), 6.98 – 6.89 (m, 2H), 5.27 (d, *J* = 12.4 Hz, 1H), 5.23 (d, *J* = 12.4 Hz, 1H), 3.86 (s, 3H), 3.73 (s, 3H), 1.88 (s, 3H). **^{13}C NMR (CDCl$_3$, 100 MHz):** δ 169.3, 168.7, 165.5, 162.5, 135.0, 129.0, 128.5, 128.4, 128.0, 125.6, 113.8, 68.0, 63.2, 55.4, 53.4, 21.1. **IR (thin film):** ν 3486, 3419, 2918, 2847, 1743, 1658, 1488, 1384, 1276, 1259, 1221, 1126, 1113, 1029, 843, 765, 750 cm^{-1}. **HRMS (ESI):** calcd. for $C_{20}H_{22}NO_6$ $(M+H)^+$ 372.1442, found 372.1443.

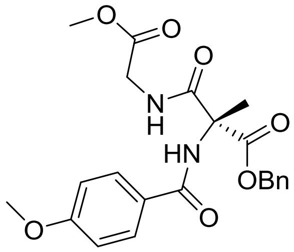

2g

Benzyl (*R*)-3-((2-methoxy-2-oxoethyl)amino)-2-(4-methoxybenzamido)-2-methyl-3-oxopropanoate (2g)

Purification by flash chromatography (petroleum ether/ethyl acetate = 1/1) afforded the product as a colorless oil (67.1 mg, 78% yield). **HPLC analysis:** 98% ee [Daicel CHIRALPAK OD-H column; solvent system: 20% isopropanol/80% hexane; 0.5 mL/min; retention times: 31.9 min (minor), 39.1 min (major)]. **$[\alpha]^{25}_D$** = 8.4 (*c* 0.30, CH_2Cl_2). **^{1}H NMR (CDCl$_3$, 400 MHz):** δ 7.82 – 7.75 (m, 2H), 7.60 (s, 1H), 7.32 – 7.27 (m, 5H), 6.95 – 6.86 (m, 3H), 5.23 (s, 2H), 4.08 (dd, *J* = 18.0, 5.6 Hz, 1H), 3.91 (dd, *J* = 18.0, 5.2 Hz, 1H), 3.85 (s, 3H), 3.74 (s, 3H), 1.94 (s, 3H). **^{13}C NMR (CDCl$_3$, 100 MHz):** δ 170.4, 169.3, 168.4, 165.9, 162.5, 135.0, 129.0, 128.4, 128.3, 128.1,

125.7, 113.7, 68.2, 63.1, 55.4, 52.5, 41.6, 22.1. **IR (thin film):** ν 3482, 2960, 2920, 2850, 1746, 1640, 1529, 1485, 1384, 1259, 1215, 1121, 1023, 768, 747, 601 cm^{-1}. **HRMS (ESI):** calcd. for $C_{22}H_{25}N_2O_7$ $(M+H)^+$ 429.1656, found 429.1651.

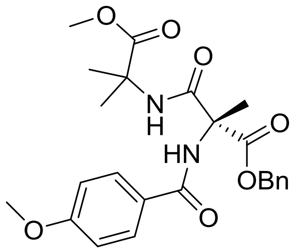

2h

Benzyl (*R*)-3-((1-methoxy-2-methyl-1-oxopropan-2-yl)amino)-2-(4-methoxybenzamido)-2-methyl-3-oxopropanoate (2h)

Purification by flash chromatography (petroleum ether/ethyl acetate = 2/1) afforded the product as a colorless oil (68.5 mg, 75% yield). **HPLC analysis:** 98% ee [Daicel CHIRALPAK OD-H column; solvent system: 20% isopropanol/80% hexane; 0.5 mL/min; retention times: 21.7 min (minor), 25.2 min (major)]. $[\alpha]^{25}_{D}$ = 13.3 (*c* 0.25, CH_2Cl_2). **^{1}H NMR ($CDCl_3$, 400 MHz):** δ 7.82 – 7.77 (m, 2H), 7.67 (s, 1H), 7.36 – 7.27 (m, 5H), 6.95 – 6.89 (m, 2H), 6.69 (s, 1H), 5.28 (d, *J* = 12.4 Hz, 1H), 5.19 (d, *J* = 12.4 Hz, 1H), 3.85 (s, 3H), 3.69 (s, 3H), 1.91 (s, 3H), 1.45 (s, 3H), 1.41 (s, 3H). **^{13}C NMR ($CDCl_3$, 100 MHz):** δ 174.1, 170.4, 167.1, 165.6, 162.4, 135.2, 129.0, 128.5, 128.4, 128.3, 125.9, 113.7, 68.0, 63.0, 57.0, 55.4, 52.8, 24.5, 24.1, 22.2. **IR (thin film):** ν 3481, 2920, 2847, 1743, 1639, 1484, 1385, 1260, 1123, 1030, 704, 625, 601 cm^{-1}. **HRMS (ESI):** calcd. for $C_{24}H_{29}N_2O_7$ $(M+H)^+$ 457.1969, found 457.1972.

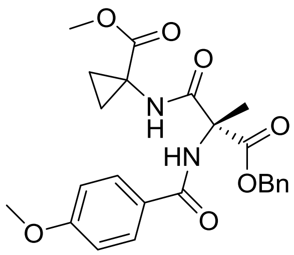

2i

Methyl (*R*)-1-(3-(benzyloxy)-2-(4-methoxybenzamido)-2-methyl-3-oxopropanamido)cyclopropane-1-carboxylate (2i)

Purification by flash chromatography (petroleum ether/ethyl acetate = 2/1) afforded the product as a colorless oil (60.7 mg, 67% yield). **HPLC analysis:** 98% ee [Daicel CHIRALPAK OD-H column; solvent system: 20% isopropanol/80% hexane; 0.5 mL/min; retention times: 23.5 min (minor), 28.6 min (major)]. $[\alpha]^{25}_{D}$ = 8.8 (*c* 0.30, CH_2Cl_2). **^{1}H NMR ($CDCl_3$, 400 MHz):** δ 7.83 – 7.76 (m, 2H), 7.68 (s, 1H), 7.29 (s, 5H), 6.97 – 6.89 (m, 2H), 6.73 (s, 1H), 5.25 (d, *J* = 12.4 Hz, 1H), 5.16 (d, *J* = 12.0 Hz, 1H), 3.86 (s, 3H), 3.63 (s, 3H), 1.93 (s, 3H), 1.60 – 1.54 (m, 1H), 1.49 – 1.42 (m, 1H), 1.06 – 0.99 (m, 1H), 0.91 – 0.85 (m, 1H). **^{13}C NMR ($CDCl_3$, 100 MHz):** δ 172.0,

170.4, 169.2, 165.8, 162.5, 135.2, 129.0, 128.5, 128.4, 128.3, 125.8, 113.8, 68.0, 63.1, 55.4, 52.6, 33.8, 22.1, 17.6, 17.3. **IR (thin film):** ν 3848, 3715, 3648, 3481, 2950, 2918, 2845, 1738, 1639, 1488, 1384, 1340, 1259, 1125, 1029, 750, 602 cm^{-1}. **HRMS (ESI):** calcd. for $C_{24}H_{27}N_2O_7$ $(M+H)^+$ 455.1813, found 455.1812.

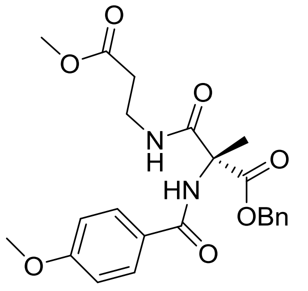

2j

Benzyl (*R*)-3-((3-methoxy-3-oxopropyl)amino)-2-(4-methoxybenzamido)-2-methyl-3-oxopropanoate (2j)

Purification by flash chromatography (petroleum ether/ethyl acetate = 1/1) afforded the product as a colorless oil (71.9 mg, 81% yield). **HPLC analysis:** 98% ee [Daicel CHIRALPAK OD-H column; solvent system: 10% isopropanol/90% hexane; 0.5 mL/min; retention times: 32.4 min (major), 45.9 min (minor)]. $[\alpha]^{25}_D$ = -0.8 (*c* 0.25, CH_2Cl_2). **^{1}H NMR ($CDCl_3$, 400 MHz):** δ 7.83 – 7.75 (m, 2H), 7.68 (s, 1H), 7.29 (s, 5H), 6.98 – 6.88 (m, 2H), 6.77 (t, *J* = 5.6 Hz, 1H), 5.23 (d, *J* = 12.4 Hz, 1H), 5.18 (d, *J* = 12.0 Hz, 1H), 3.85 (s, 3H), 3.64 (s, 3H), 3.51 – 3.45 (m, 2H), 2.53 – 2.39 (m, 2H), 1.88 (s, 3H). **^{13}C NMR ($CDCl_3$, 100 MHz):** δ 172.3, 170.4, 167.9, 165.7, 162.5, 135.1, 129.0, 128.4, 128.3, 128.1, 125.9, 113.7, 68.0, 63.1, 55.4, 51.8, 35.6, 33.2, 22.0. **IR (thin film):** ν 3482, 3419, 2918, 2850, 1738, 1640, 1533, 1488, 1384, 1258, 1177, 1121, 1029, 845, 700, 588 cm^{-1}. **HRMS (ESI):** calcd. for $C_{23}H_{27}N_2O_7$ $(M+H)^+$ 443.1813, found 443.1814.

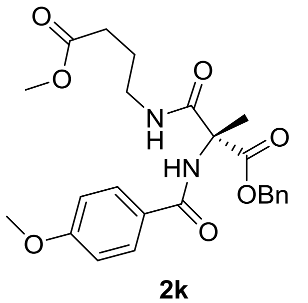

2k

Methyl (*R*)-4-(3-(benzyloxy)-2-(4-methoxybenzamido)-2-methyl-3-oxopropanamido)butanoate (2k)

Purification by flash chromatography (petroleum ether/ethyl acetate = 1/1) afforded the product as a colorless oil (72.7 mg, 80% yield). **HPLC analysis:** 97% ee [Daicel CHIRALPAK OD-H column; solvent system: 10% isopropanol/90% hexane; 0.5 mL/min; retention times: 33.9 min (major), 39.8 min (minor)]. $[\alpha]^{25}_D$ = 7.8 (*c* 0.35, CH_2Cl_2). **^{1}H NMR ($CDCl_3$, 400 MHz):** δ 7.82 – 7.76 (m, 2H), 7.71 (s, 1H), 7.29 (s, 5H), 6.96 – 6.90 (m, 2H), 6.46 (t, *J* = 5.2 Hz, 1H), 5.23 (d, *J* = 12.4 Hz, 1H), 5.19 (d,

J = 12.4 Hz, 1H), 3.86 (s, 3H), 3.67 (s, 3H), 3.34 – 3.18 (m, 2H), 2.27 (t, J = 7.2 Hz, 2H), 1.89 (s, 3H), 1.78 – 1.70 (m, 2H). **^{13}C NMR ($CDCl_3$, 100 MHz):** δ 173.4, 170.6, 168.1, 165.8, 162.5, 135.1, 129.0, 128.4, 128.3, 128.2, 125.9, 113.7, 68.0, 63.1, 55.4, 51.7, 39.5, 31.1, 24.2, 22.2. **IR (thin film):** ν 3707, 3646, 3482, 2922, 2850, 1738, 1647, 1558, 1489, 1384, 1259, 1175, 1119, 1023, 851, 806, 602 cm^{-1}. **HRMS (ESI):** calcd. for $C_{24}H_{29}N_2O_7$ $(M+H)^+$ 457.1969, found 457.1970.

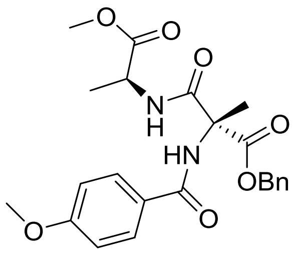

2l

Benzyl (*R*)-3-(((*S*)-1-methoxy-1-oxopropan-2-yl)amino)-2-(4-methoxybenzamido)-2-methyl-3-oxopropanoate (2l)

1H NMR analysis of the crude mixture showed a dr of >99:1. Purification by flash chromatography (petroleum ether/ethyl acetate = 2/1) afforded the product as a colorless oil (69.8 mg, 79% yield). **$[\alpha]^{25}_D$** = 3.2 (*c* 0.25, CH_2Cl_2). **^{1}H NMR ($CDCl_3$, 400 MHz):** δ 7.82 – 7.75 (m, 2H), 7.63 (s, 1H), 7.34 – 7.27 (m, 5H), 6.95 – 6.90 (m, 2H), 6.87 (d, J = 7.2 Hz, 1H), 5.26 (d, J = 12.4 Hz, 1H), 5.22 (d, J = 12.4 Hz, 1H), 4.55 – 4.45 (m, 1H), 3.85 (s, 3H), 3.70 (s, 3H), 1.93 (s, 3H), 1.40 (d, J = 7.2 Hz, 3H). **^{13}C NMR ($CDCl_3$, 100 MHz):** δ 172.3, 170.3, 167.6, 165.9, 162.5, 135.1, 129.0, 128.4, 128.2, 128.1, 125.9, 113.7, 68.1, 63.2, 55.4, 52.5, 48.7, 22.1, 17.9. **IR (thin film):** ν 3481, 3416, 2953, 2921, 2847, 1743, 1639, 1529, 1484, 1384, 1258, 1210, 1173, 1123, 603, 570 cm^{-1}. **HRMS (ESI):** calcd. for $C_{23}H_{27}N_2O_7$ $(M+H)^+$ 443.1813, found 443.1815.

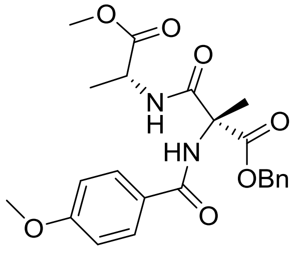

2m

Benzyl (*R*)-3-(((*R*)-1-methoxy-1-oxopropan-2-yl)amino)-2-(4-methoxybenzamido)-2-methyl-3-oxopropanoate (2m)

1H NMR analysis of the crude mixture showed a dr of >99:1. Purification by flash chromatography (petroleum ether/ethyl acetate = 2/1) afforded the product as a colorless oil (71.0 mg, 80% yield). **$[\alpha]^{25}_D$** = 6.0 (*c* 0.20, CH_2Cl_2). **^{1}H NMR ($CDCl_3$, 400 MHz):** δ 7.82 – 7.77 (m, 2H), 7.66 (s, 1H), 7.34 – 7.27 (m, 5H), 6.96 – 6.90 (m, 2H), 6.67 (d, J = 7.2 Hz, 1H), 5.27 (d, J = 12.0 Hz, 1H), 5.18 (d, J = 12.4 Hz, 1H),

4.53 – 4.43 (m, 1H), 3.85 (s, 3H), 3.74 (s, 3H), 1.93 (s, 3H), 1.22 (d, *J* = 7.2 Hz, 3H). **^{13}C NMR ($CDCl_3$, 100 MHz):** δ 172.4, 170.3, 167.6, 165.7, 162.5, 135.1, 129.0, 128.8, 128.5, 128.4, 125.8, 113.7, 68.0, 63.0, 55.4, 52.6, 48.6, 22.1, 17.6. **IR (thin film):** ν 3382, 3065, 3034, 2958, 2922, 2851, 1747, 1647, 1608, 1526, 1488, 1455, 1380, 1299, 1260, 1215, 1178, 1125, 1029, 846, 800, 770, 751, 699, 601 cm^{-1}. **HRMS (ESI):** calcd. for $C_{23}H_{27}N_2O_7$ $(M+H)^+$ 443.1813, found 443.1813.

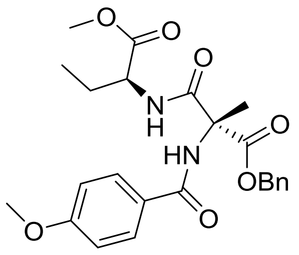

2n

Methyl (*S*)-2-((*R*)-3-(benzyloxy)-2-(4-methoxybenzamido)-2-methyl-3-oxopropanamido)butanoate (2n)

1H NMR analysis of the crude mixture showed a dr of >99:1. Purification by flash chromatography (petroleum ether/ethyl acetate = 2/1) afforded the product as a colorless oil (71.0 mg, 78% yield). **$[\alpha]^{25}_D$** = 2.8 (*c* 0.50, CH_2Cl_2). **^{1}H NMR ($CDCl_3$, 400 MHz):** δ 7.82 – 7.75 (m, 2H), 7.64 (s, 1H), 7.33 – 7.27 (m, 5H), 6.95 – 6.90 (m, 2H), 6.87 (d, *J* = 7.6 Hz, 1H), 5.24 (s, 2H), 4.50 (td, *J* = 7.2, 5.2 Hz, 1H), 3.85 (s, 3H), 3.70 (s, 3H), 1.94 (s, 3H), 1.93 – 1.86 (m, 1H), 1.78 – 1.67 (m, 1H), 0.88 (t, *J* = 7.6 Hz, 3H). **^{13}C NMR ($CDCl_3$, 100 MHz):** δ 171.7, 170.4, 167.9, 165.9, 162.5, 135.1, 129.0, 128.5, 128.3, 128.1, 125.9, 113.8, 68.2, 63.4, 55.4, 53.9, 52.5, 25.3, 22.3, 9.4. **IR (thin film):** ν 3363, 3065, 3034, 2961, 2923, 2851, 1744, 1659, 1647, 1607, 1577, 1532, 1500, 1488, 1381, 1299, 1259, 1211, 1179, 1125, 1029, 846, 802, 770, 699, 596 cm^{-1}. **HRMS (ESI):** calcd. for $C_{24}H_{29}N_2O_7$ $(M+H)^+$ 457.1969, found 457.1971.

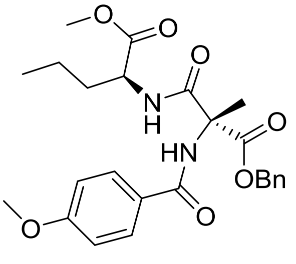

2o

Methyl (*S*)-2-((*R*)-3-(benzyloxy)-2-(4-methoxybenzamido)-2-methyl-3-oxopropanamido)pentanoate (2o)

1H NMR analysis of the crude mixture showed a dr of >99:1. Purification by flash chromatography (petroleum ether/ethyl acetate = 5/2) afforded the product as a colorless oil (69.5 mg, 74% yield). **$[\alpha]^{25}_D$** = 4.0 (*c* 0.15, CH_2Cl_2). **^{1}H NMR ($CDCl_3$, 400 MHz):** δ 7.82 – 7.75 (m, 2H), 7.63 (s, 1H), 7.33 – 7.27 (m, 5H), 6.95 – 6.90 (m, 2H), 6.81 (d, *J* = 7.6 Hz, 1H), 5.25 (d, *J* = 12.4 Hz, 1H), 5.22 (d, *J* = 12.4 Hz, 1H),

4.53 (td, *J* = 7.6, 5.2 Hz, 1H), 3.85 (s, 3H), 3.69 (s, 3H), 1.94 (s, 3H), 1.88 – 1.77 (m, 1H), 1.70 – 1.60 (m, 1H), 1.38 – 1.25 (m, 2H), 0.91 (t, *J* = 7.6 Hz, 3H). **^{13}C NMR ($CDCl_3$, 100 MHz):** δ 171.9, 170.3, 167.9, 165.9, 162.5, 135.0, 129.0, 128.4, 128.2, 128.1, 125.9, 113.7, 68.2, 63.3, 55.4, 52.7, 52.4, 34.1, 22.2, 18.5, 13.6. **IR (thin film):** ν 3408, 3005, 2959, 2921, 2850, 1744, 1659, 1647, 1607, 1532, 1488, 1471, 1379, 1301, 1259, 1209, 1181, 1114, 1030, 801, 769, 739, 699 cm^{-1}. **HRMS (ESI):** calcd. for $C_{25}H_{31}N_2O_7$ $(M+H)^+$ 471.2126, found 471.2123.

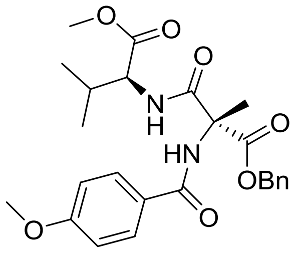

2p

Methyl ((*R*)-3-(benzyloxy)-2-(4-methoxybenzamido)-2-methyl-3-oxopropanoyl)-*L*-valinate (2p)

1H NMR analysis of the crude mixture showed a dr of >99:1. Purification by flash chromatography (petroleum ether/ethyl acetate = 2/1) afforded the product as a colorless oil (74.0 mg, 79% yield). **$[\alpha]^{25}_D$** = 4.6 (*c* 1.0, CH_2Cl_2). **^{1}H NMR ($CDCl_3$, 400 MHz):** δ 7.84 – 7.73 (m, 2H), 7.64 (s, 1H), 7.34 – 7.27 (m, 5H), 6.97 – 6.87 (m, 3H), 5.24 (s, 2H), 4.50 (dd, *J* = 8.8, 4.8 Hz, 1H), 3.85 (s, 3H), 3.68 (s, 3H), 2.24 – 2.13 (m, 1H), 1.95 (s, 3H), 0.93 (d, *J* = 6.8 Hz, 3H), 0.88 (d, *J* = 6.8 Hz, 3H). **^{13}C NMR ($CDCl_3$, 100 MHz):** δ 171.3, 170.4, 168.1, 166.0, 162.5, 135.0, 129.0, 128.4, 128.2, 128.1, 125.9, 113.7, 68.2, 63.4, 57.7, 55.4, 52.2, 31.2, 22.3, 18.9, 17.5. **IR (thin film):** ν 3345, 3064, 3034, 2963, 2935, 2875, 2850, 1743, 1682, 1607, 1532, 1504, 1373, 1302, 1259, 1210, 1180, 1124, 1029, 846, 770, 737, 699, 595 cm^{-1}. **HRMS (ESI):** calcd. for $C_{25}H_{31}N_2O_7$ $(M+H)^+$ 471.2126, found 471.2137.

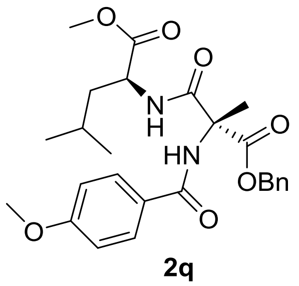

2q

Methyl ((*R*)-3-(benzyloxy)-2-(4-methoxybenzamido)-2-methyl-3-oxopropanoyl)-*L*-leucinate (2q)

1H NMR analysis of the crude mixture showed a dr of >99:1. Purification by flash chromatography (petroleum ether/ethyl acetate = 3/1) afforded the product as a colorless oil (77.8 mg, 80% yield). **$[\alpha]^{25}_D$** = 0.9 (*c* 0.45, CH_2Cl_2). **^{1}H NMR ($CDCl_3$, 400 MHz):** δ 7.82 – 7.76 (m, 2H), 7.63 (s, 1H), 7.33 – 7.27 (m, 5H), 6.96 – 6.88 (m, 2H), 6.73 (d, *J* = 8.0 Hz, 1H), 5.26 (d, *J* = 12.4 Hz, 1H), 5.21 (d, *J* = 12.4 Hz, 1H),

4.57 (td, J = 8.4, 5.2 Hz, 1H), 3.85 (s, 3H), 3.67 (s, 3H), 1.93 (s, 3H), 1.71 – 1.50 (m, 3H), 0.93 (d, J = 6.0 Hz, 6H). **^{13}C NMR ($CDCl_3$, 100 MHz):** δ 172.3, 170.4, 168.0, 166.0, 162.5, 135.1, 129.0, 128.5, 128.3, 128.1, 125.9, 113.8, 68.2, 63.3, 55.4, 52.4, 51.4, 41.1, 24.9, 22.8, 22.2, 21.8. **IR (thin film):** ν 3714, 3647, 3360, 3065, 3034, 2958, 2925, 2871, 2853, 1747, 1688, 1660, 1607, 1577, 1532, 1488, 1441, 1372, 1258, 1208, 1122, 1029, 770, 698 cm^{-1}. **HRMS (ESI):** calcd. for $C_{26}H_{33}N_2O_7$ $(M+H)^+$ 485.2282, found 485.2285.

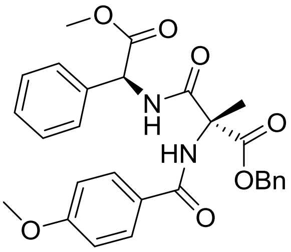

2r

Benzyl (*R*)-3-(((*S*)-2-methoxy-2-oxo-1-phenylethyl)amino)-2-(4-methoxybenzamido)-2-methyl-3-oxopropanoate (2r)

1H NMR analysis of the crude mixture showed a dr of >99:1. Purification by flash chromatography (petroleum ether/ethyl acetate = 5/2) afforded the product as a colorless oil (76.3 mg, 76% yield). **$[\alpha]^{25}_{D}$** = 42.9 (c 0.90, CH_2Cl_2). **^{1}H NMR ($CDCl_3$, 400 MHz):** δ 7.81 – 7.74 (m, 2H), 7.60 (s, 1H), 7.46 (d, J = 6.4 Hz, 1H), 7.38 – 7.27 (m, 10H), 6.94 – 6.86 (m, 2H), 5.44 (d, J = 6.8 Hz, 1H), 5.27 (d, J = 12.4 Hz, 1H), 5.22 (d, J = 12.4 Hz, 1H), 3.84 (s, 3H), 3.69 (s, 3H), 1.90 (s, 3H). **^{13}C NMR ($CDCl_3$, 100 MHz):** δ 170.3, 170.3, 167.5, 166.0, 162.5, 135.8, 135.1, 129.1, 129.0, 128.8, 128.5, 128.3, 128.2, 127.0, 125.9, 113.7, 68.3, 63.2, 57.1, 55.4, 53.0, 22.1. **IR (thin film):** ν 3390, 3064, 3033, 3007, 2955, 2921, 2849, 1743, 1680, 1648, 1607, 1522, 1499, 1456, 1439, 1380, 1320, 1300, 1259, 1214, 1176, 1124, 1029, 769, 736, 698 cm^{-1}. **HRMS (ESI):** calcd. for $C_{28}H_{29}N_2O_7$ $(M+H)^+$ 505.1969, found 505.1962.

Different Substrates:

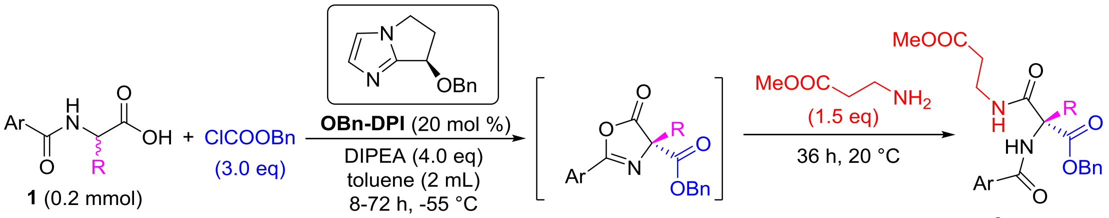

Under a N_2 atmosphere, the substrate **1** (0.2 mmol, 44.7 mg), the catalyst **OBn-DPI** (0.04 mmol, 8.6 mg) and DIPEA (0.8 mmol, 132.2 μL) were dissolved in anhydrous toluene (2 mL) and cooled to −55 ℃ in a dry two-necked flask. ClCOOBn (0.6 mmol, 84.5 μL) was then added and the vial was sealed with a septum. The reaction mixture was stirred at −55 ℃ for 8-72 h (Reaction time for **2s-v** was 72 h, reaction time for

2w-x was 8 h, reaction time for **2y-ad** was 10 h) and then the temperature was gradually raised to 20 ℃. Methyl 3-aminopropionate hydrochloride (0.3 mmol, 41.9 mg) and DIPEA (0.3 mmol, 49.6 μL) were dissolved in anhydrous toluene (1 mL) in another dry flask and stirred for 10 min. Then the mixture was transferred into the former reaction flask and the reaction mixture was stirred at 20 ℃ for 36 h. The reaction mixture was quenched with 0.2 M HCl (5 mL) and extracted with DCM (5 mL × 3). The combined organic phases were dried over Na_2SO_4. After filtration, the residue was purified by column chromatography (petroleum ether/ethyl acetate) to give the corresponding product **2**. The ee value was determined by chiral HPLC analysis after purification by column chromatography (petroleum ether/ethyl acetate).

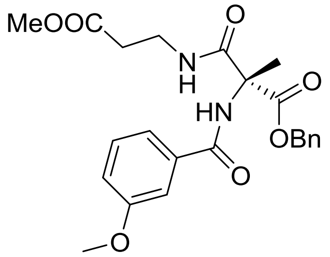

2s

Benzyl (*R*)-3-((3-methoxy-3-oxopropyl)amino)-2-(3-methoxybenzamido)-2-methyl-3-oxopropanoate (2s)

Purification by flash chromatography (petroleum ether/ethyl acetate = 3/2) afforded the product as a colorless oil (73.3 mg, 83% yield). **HPLC analysis:** 97% ee [Daicel CHIRALPAK AS-H column; solvent system: 20% isopropanol/80% hexane; 1.0 mL/min; retention times: 13.8 min (minor), 36.9 min (major)]. $[\alpha]^{25}_{D}$ = 2.9 (*c* 0.70, CH_2Cl_2). **^{1}H NMR ($CDCl_3$, 400 MHz):** δ 7.79 (s, 1H), 7.41 – 7.27 (m, 8H), 7.09 – 7.02 (m, 1H), 6.76 (t, *J* = 5.6 Hz, 1H), 5.24 (d, *J* = 12.0 Hz, 1H), 5.19 (d, *J* = 12.0 Hz, 1H), 3.84 (s, 3H), 3.65 (s, 3H), 3.51 – 3.45 (m, 2H), 2.55 – 2.38 (m, 2H), 1.89 (s, 3H). **^{13}C NMR ($CDCl_3$, 100 MHz):** δ 172.4, 170.3, 167.8, 166.1, 159.8, 135.1, 135.1, 129.6, 128.5, 128.4, 128.2, 119.0, 118.4, 112.2, 68.1, 63.2, 55.5, 51.9, 35.7, 33.3, 21.9. **IR (thin film):** ν 3372, 3066, 3034, 3002, 2955, 2922, 2851, 1744, 1731, 1659, 1583, 1506, 1481, 1373, 1262, 1225, 1122, 1044, 800, 758, 698 cm^{-1}. **HRMS (ESI):** calcd. for $C_{23}H_{27}N_2O_7$ $(M+H)^+$ 443.1813, found 443.1811.

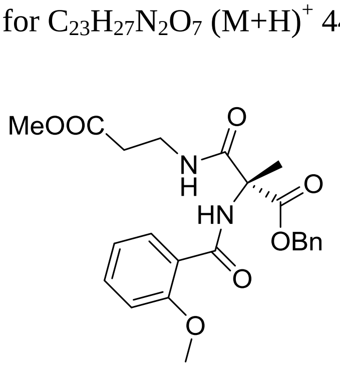

2t

Benzyl (*R*)-3-((3-methoxy-3-oxopropyl)amino)-2-(2-methoxybenzamido)-2-methyl-3-oxopropanoate (2t)

Purification by flash chromatography (petroleum ether/ethyl acetate = 3/2) afforded the product as a colorless oil (67.1 mg, 76% yield). **HPLC analysis:** 98% ee [Daicel CHIRALPAK AS-H column; solvent system: 20% isopropanol/80% hexane; 0.5 mL/min; retention times: 26.1 min (minor), 30.4 min (major)]. **$[\alpha]^{25}_{D}$** = 0.9 (*c* 0.70, CH_2Cl_2). **^{1}H NMR ($CDCl_3$, 400 MHz):** δ 9.45 (s, 1H), 8.15 (dd, *J* = 8.0, 5.6 Hz, 1H), 7.52 – 7.43 (m, 1H), 7.35 – 7.27 (m, 5H), 7.11 – 7.04 (m, 1H), 6.99 (d, *J* = 8.0 Hz, 1H), 6.84 (t, *J* = 5.6 Hz, 1H), 5.25 (d, *J* = 12.4 Hz, 1H), 5.20 (d, *J* = 12.0 Hz, 1H), 4.01 (s, 3H), 3.61 (s, 3H), 3.54 – 3.41 (m, 2H), 2.54 – 2.40 (m, 2H), 1.88 (s, 3H). **^{13}C NMR ($CDCl_3$, 100 MHz):** δ 172.5, 170.6, 168.2, 164.2, 158.0, 135.3, 133.3, 132.1, 128.5, 128.3, 128.1, 121.1, 120.8, 111.4, 67.9, 63.8, 56.0, 51.8, 35.5, 33.4, 21.9. **IR (thin film):** ν 3360, 3067, 3033, 2922, 2850, 1738, 1688, 1647, 1601, 1516, 1483, 1440, 1373, 1301, 1262, 1241, 1178, 1103, 1048, 1021, 801, 758, 699, 608 cm^{-1}. **HRMS (ESI):** calcd. for $C_{23}H_{27}N_2O_7$ $(M+H)^+$ 443.1813, found 443.1815.

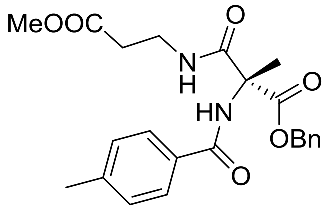

2u

Benzyl (*R*)-3-((3-methoxy-3-oxopropyl)amino)-2-methyl-2-(4-methylbenzamido)-3-oxopropanoate (2u)

Purification by flash chromatography (petroleum ether/ethyl acetate = 3/2) afforded the product as a colorless oil (63.2 mg, 74% yield). **HPLC analysis:** 98% ee [Daicel CHIRALPAK AS-H column; solvent system: 20% isopropanol/80% hexane; 1.0 mL/min; retention times: 11.2 min (minor), 21.3 min (major)]. **$[\alpha]^{25}_{D}$** = -0.8 (*c* 0.25, CH_2Cl_2). **^{1}H NMR ($CDCl_3$, 400 MHz):** δ 7.81 – 7.67 (m, 3H), 7.29 (s, 5H), 7.23 (d, *J* = 8.0 Hz, 2H), 6.76 (t, *J* = 5.6 Hz, 1H), 5.23 (d, *J* = 12.4 Hz, 1H), 5.18 (d, *J* = 12.4 Hz, 1H), 3.64 (s, 3H), 3.51 – 3.45 (m, 2H), 2.53 – 2.42 (m, 2H), 2.40 (s, 3H), 1.88 (s, 3H). **^{13}C NMR ($CDCl_3$, 100 MHz):** δ 172.4, 170.4, 167.9, 166.1, 142.3, 135.1, 130.7, 129.2, 128.4, 128.3, 128.1, 127.1, 68.0, 63.1, 51.8, 35.6, 33.2, 21.9, 21.5. **IR (thin film):** ν 3475, 3414, 2960, 2922, 2851, 1738, 1644, 1524, 1487, 1384, 1262, 1122, 799, 753, 698, 602 cm^{-1}. **HRMS (ESI):** calcd. for $C_{23}H_{27}N_2O_6$ $(M+H)^+$ 427.1864, found 427.1860.

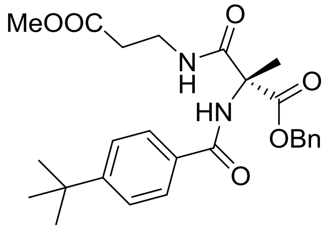

2v

Benzyl (*R*)-2-(4-(*tert*-butyl)benzamido)-3-((3-methoxy-3-oxopropyl)amino)-2-

methyl-3-oxopropanoate (2v)

Purification by flash chromatography (petroleum ether/ethyl acetate = 2/1) afforded the product as a colorless oil (73.2 mg, 78% yield). **HPLC analysis:** 98% ee [Daicel CHIRALPAK AS-H column; solvent system: 20% isopropanol/80% hexane; 1.0 mL/min; retention times: 7.6 min (minor), 10.8 min (major)]. $[\alpha]^{25}_D$ = 2.7 (*c* 0.15, CH_2Cl_2). **^{1}H NMR ($CDCl_3$, 400 MHz):** δ 7.81 – 7.72 (m, 3H), 7.45 (d, *J* = 8.4 Hz, 2H), 7.29 (s, 5H), 6.75 (t, *J* = 5.6 Hz, 1H), 5.24 (d, *J* = 12.0 Hz, 1H), 5.17 (d, *J* = 12.0 Hz, 1H), 3.64 (s, 3H), 3.51 – 3.45 (m, 2H), 2.55 – 2.38 (m, 2H), 1.88 (s, 3H), 1.34 (s, 9H). **^{13}C NMR ($CDCl_3$, 100 MHz):** δ 172.3, 170.4, 167.9, 166.1, 155.4, 135.1, 130.7, 128.4, 128.3, 128.1, 127.0, 125.4, 68.0, 63.1, 51.8, 35.6, 34.9, 33.3, 31.1, 21.9. **IR (thin film):** ν 3481, 3416, 2962, 2922, 2852, 1738, 1647, 1526, 1488, 1384, 1261, 1118, 1018, 801, 697, 602 cm^{-1}. **HRMS (ESI):** calcd. for $C_{26}H_{33}N_2O_6$ $(M+H)^+$ 469.2333, found 469.2332.

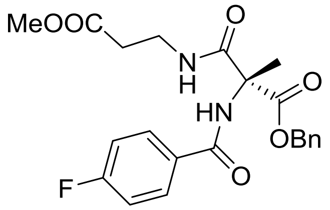

2w

Benzyl (*R*)-2-(4-fluorobenzamido)-3-((3-methoxy-3-oxopropyl)amino)-2-methyl-3-oxopropanoate (2w)

Purification by flash chromatography (petroleum ether/ethyl acetate = 3/2) afforded the product as a colorless oil (65.8 mg, 76% yield). **HPLC analysis:** 97% ee [Daicel CHIRALPAK AS-H column; solvent system: 20% isopropanol/80% hexane; 1.0 mL/min; retention times: 10.6 min (minor), 36.7 min (major)]. $[\alpha]^{25}_D$ = 4.4 (*c* 0.5, CH_2Cl_2). **^{1}H NMR ($CDCl_3$, 400 MHz):** δ 7.85 – 7.79 (m, 2H), 7.77 (s, 1H), 7.29 (s, 5H), 7.14 – 7.07 (m, 2H), 6.78 (t, *J* = 5.7 Hz, 1H), 5.23 (d, *J* = 12.3 Hz, 1H), 5.19 (d, *J* = 12.3 Hz, 1H), 3.65 (s, 3H), 3.52 – 3.45 (m, 2H), 2.55 – 2.38 (m, 2H), 1.88 (s, 3H). **^{13}C NMR ($CDCl_3$, 100 MHz):** δ 172.4, 170.2, 167.8, 165.2, 165.0 (d, *J* = 250.9 Hz), 135.0, 129.8 (d, *J* = 3.0 Hz), 129.5 (d, *J* = 9.0 Hz), 128.5, 128.4, 128.2, 115.6 (d, *J* = 21.8 Hz), 68.1, 63.2, 51.9, 35.7, 33.2, 21.9. **IR (thin film):** ν 3648, 3481, 2960, 2920, 2850, 1738, 1659, 1605, 1533, 1488, 1471, 1384, 1262, 1127, 852, 802, 700 cm^{-1}. **HRMS (ESI):** calcd. for $C_{22}H_{24}FN_2O_6$ $(M+H)^+$ 431.1613, found 431.1614.

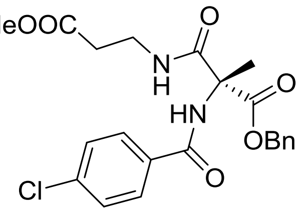

2x

Benzyl (*R*)-2-(4-chlorobenzamido)-3-((3-methoxy-3-oxopropyl)amino)-2-methyl-

3-oxopropanoate (2x)

Purification by flash chromatography (petroleum ether/ethyl acetate = 3/2) afforded the product as a light yellow oil (65.1 mg, 73% yield). **HPLC analysis:** 98% ee [Daicel CHIRALPAK AS-H column; solvent system: 20% isopropanol/80% hexane; 1.0 mL/min; retention times: 11.7 min (minor), 35.7 min (major)]. **$[\alpha]^{25}_D$** = -0.8 (*c* 0.25, CH_2Cl_2). **^1H NMR ($CDCl_3$, 400 MHz):** δ 7.82 – 7.72 (m, 3H), 7.44 – 7.38 (m, 2H), 7.32 – 7.27 (m, 5H), 6.74 (t, *J* = 5.6 Hz, 1H), 5.22 (d, *J* = 12.0 Hz, 1H), 5.18 (d, *J* = 12.4 Hz, 1H), 3.66 (s, 3H), 3.52 – 3.44 (m, 2H), 2.54 – 2.38 (m, 2H), 1.88 (s, 3H). **^{13}C NMR ($CDCl_3$, 100 MHz):** δ 172.3, 170.1, 167.6, 165.1, 138.1, 135.0, 132.0, 128.8, 128.5, 128.5, 128.4, 128.2, 68.1, 63.2, 51.9, 35.7, 33.2, 21.9. **IR (thin film):** ν 3481, 2960, 2920, 2850, 1738, 1659, 1647, 1541, 1472, 1384, 1263, 1123, 1093, 1014, 800, 701 cm^{-1}. **HRMS (ESI):** calcd. for $C_{22}H_{24}ClN_2O_6$ $(M+H)^+$ 447.1317, found 447.1318.

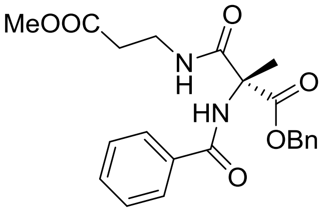

2y

Benzyl (*R*)-2-benzamido-3-((3-methoxy-3-oxopropyl)amino)-2-methyl-3-oxopropanoate (2y)

Purification by flash chromatography (petroleum ether/ethyl acetate = 2/1) afforded the product as a colorless oil (66.3 mg, 80% yield). **HPLC analysis:** 98% ee [Daicel CHIRALPAK AS-H column; solvent system: 20% isopropanol/80% hexane; 1.0 mL/min; retention times: 11.6 min (minor), 32.3 min (major)]. **$[\alpha]^{25}_D$** = 0.7 (*c* 0.55, CH_2Cl_2). **^1H NMR ($CDCl_3$, 400 MHz):** δ 7.89 – 7.78 (m, 3H), 7.55 – 7.49 (m, 1H), 7.48 – 7.40 (m, 2H), 7.29 (s, 5H), 6.77 (t, *J* = 5.6 Hz, 1H), 5.24 (d, *J* = 12.0 Hz, 1H), 5.19 (d, *J* = 12.4 Hz, 1H), 3.65 (s, 3H), 3.52 – 3.45 (m, 2H), 2.54 – 2.38 (m, 2H), 1.89 (s, 3H). **^{13}C NMR ($CDCl_3$, 100 MHz):** δ 172.3, 170.3, 167.8, 166.2, 135.0, 133.6, 131.8, 128.5, 128.4, 128.3, 128.1, 127.1, 68.1, 63.2, 51.8, 35.6, 33.2, 21.9. **IR (thin film):** ν 3481, 3420, 2962, 2925, 2852, 1731, 1647, 1580, 1539, 1472, 1384, 1261, 1110, 1025, 801, 697, 602 cm^{-1}. **HRMS (ESI):** calcd. for $C_{22}H_{25}N_2O_6$ $(M+H)^+$ 413.1707, found 413.1703.

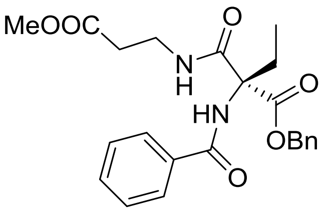

2z

Benzyl (*R*)-2-benzamido-2-((3-methoxy-3-oxopropyl)carbamoyl)butanoate (2z)

Purification by flash chromatography (petroleum ether/ethyl acetate = 2/1) afforded the product as a colorless oil (66.1 mg, 78% yield). **HPLC analysis:** 98% ee [Daicel CHIRALPAK AS-H column; solvent system: 20% isopropanol/80% hexane; 1.0 mL/min; retention times: 10.2 min (minor), 14.1 min (major)]. $[\alpha]^{25}_{D}$ = -1.8 (*c* 0.55, CH_2Cl_2). **^{1}H NMR ($CDCl_3$, 400 MHz):** δ 7.88 – 7.80 (m, 2H), 7.75 (s, 1H), 7.57 – 7.49 (m, 1H), 7.48 – 7.41 (m, 2H), 7.29 (s, 5H), 6.78 (t, *J* = 5.6 Hz, 1H), 5.24 (d, *J* = 12.0 Hz, 1H), 5.18 (d, *J* = 12.4 Hz, 1H), 3.65 (s, 3H), 3.54 – 3.43 (m, 2H), 2.78 – 2.66 (m, 1H), 2.56 – 2.39 (m, 2H), 2.34 – 2.23 (m, 1H), 0.79 (t, *J* = 7.6 Hz, 3H). **^{13}C NMR ($CDCl_3$, 100 MHz):** δ 172.4, 170.1, 166.8, 166.1, 135.1, 133.7, 131.8, 128.6, 128.5, 128.4, 128.2, 127.1, 68.0, 67.3, 51.9, 35.6, 33.3, 26.6, 7.7. **IR (thin film):** ν 3400, 3063, 3032, 2960, 2922, 2851, 1738, 1659, 1648, 1580, 1507, 1475, 1384, 1259, 1220, 1089, 1028, 802, 698 cm^{-1}. **HRMS (ESI):** calcd. for $C_{23}H_{27}N_2O_6$ $(M+H)^+$ 427.1864, found 427.1861.

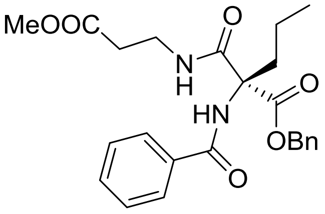

2aa

Benzyl (*R*)-2-benzamido-2-((3-methoxy-3-oxopropyl)carbamoyl)pentanoate (2aa)

Purification by flash chromatography (petroleum ether/ethyl acetate = 5/2) afforded the product as a colorless oil (67.7 mg, 77% yield). **HPLC analysis:** 98% ee [Daicel CHIRALPAK AS-H column; solvent system: 20% isopropanol/80% hexane; 1.0 mL/min; retention times: 9.2 min (minor), 12.3 min (major)]. $[\alpha]^{25}_{D}$ = -0.8 (*c* 0.50, CH_2Cl_2). **^{1}H NMR ($CDCl_3$, 400 MHz):** δ 7.85 – 7.80 (m, 2H), 7.76 (s, 1H), 7.55 – 7.49 (m, 1H), 7.48 – 7.41 (m, 2H), 7.29 (s, 5H), 6.78 (t, *J* = 5.6 Hz, 1H), 5.24 (d, *J* = 12.0 Hz, 1H), 5.18 (d, *J* = 12.0 Hz, 1H), 3.65 (s, 3H), 3.56 – 3.41 (m, 2H), 2.70 – 2.58 (m, 1H), 2.57 – 2.37 (m, 2H), 2.26 – 2.14 (m, 1H), 1.30 – 1.17 (m, 1H), 1.16 – 1.02 (m, 1H), 0.88 (t, *J* = 7.2 Hz, 3H). **^{13}C NMR ($CDCl_3$, 100 MHz):** δ 172.4, 170.1, 166.9, 166.1, 135.1, 133.7, 131.8, 128.6, 128.5, 128.4, 128.2, 127.1, 68.0, 66.9, 51.9, 35.6, 35.4, 33.3, 17.0, 13.8. **IR (thin film):** ν 3714, 3648, 3377, 3065, 3033, 2960, 2923, 2874, 2851, 1738, 1659, 1506, 1476, 1368, 1281, 1218, 1178, 1028, 803, 698, 601 cm^{-1}. **HRMS (ESI):** calcd. for $C_{24}H_{29}N_2O_6$ $(M+H)^+$ 441.2020, found 441.2016.

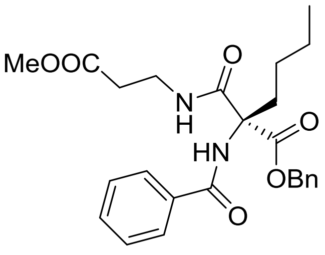

2ab

Benzyl (*R*)-2-benzamido-2-((3-methoxy-3-oxopropyl)carbamoyl)hexanoate (2ab)

Purification by flash chromatography (petroleum ether/ethyl acetate = 3/1) afforded the product as a colorless oil (67.0 mg, 74% yield). **HPLC analysis:** 99% ee [Daicel CHIRALPAK OD-H column; solvent system: 20% isopropanol/80% hexane; 1.0 mL/min; retention times: 7.8 min (minor), 11.1 min (major)]. $[\alpha]^{25}_D$ = -1.2 (*c* 0.50, CH_2Cl_2). **^{1}H NMR ($CDCl_3$, 400 MHz):** δ 7.86 – 7.80 (m, 2H), 7.75 (s, 1H), 7.56 – 7.49 (m, 1H), 7.48 – 7.41 (m, 2H), 7.29 (s, 5H), 6.79 (t, *J* = 5.6 Hz, 1H), 5.24 (d, *J* = 12.4 Hz, 1H), 5.17 (d, *J* = 12.4 Hz, 1H), 3.65 (s, 3H), 3.56 – 3.41 (m, 2H), 2.70 – 2.60 (m, 1H), 2.57 – 2.39 (m, 2H), 2.28 – 2.16 (m, 1H), 1.34 – 1.22 (m, 2H), 1.22 – 1.10 (m, 1H), 1.09 – 0.97 (m, 1H), 0.82 (t, *J* = 7.2 Hz, 3H). **^{13}C NMR ($CDCl_3$, 100 MHz):** δ 172.4, 170.1, 167.0, 166.0, 135.1, 133.7, 131.8, 128.6, 128.5, 128.4, 128.2, 127.2, 68.0, 66.8, 51.8, 35.6, 33.3, 33.1, 25.7, 22.4, 13.8. **IR (thin film):** ν 3390, 3063, 2959, 2924, 2853, 1738, 1659, 1580, 1507, 1472, 1376, 1264, 1214, 1111, 800, 696 cm^{-1}. **HRMS (ESI):** calcd. for $C_{25}H_{31}N_2O_6$ $(M+H)^+$ 455.2177, found 455.2172.

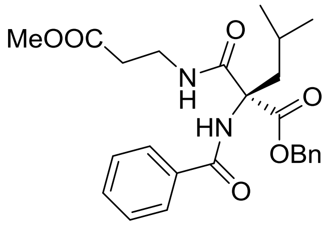

2ac

Benzyl (*R*)-2-benzamido-2-((3-methoxy-3-oxopropyl)carbamoyl)-4-methylpentanoate (2ac)

Purification by flash chromatography (petroleum ether/ethyl acetate = 3/1) afforded the product as a colorless oil (67.9 mg, 75% yield). **HPLC analysis:** 99% ee [Daicel CHIRALPAK AS-H column; solvent system: 20% isopropanol/80% hexane; 1.0 mL/min; retention times: 7.6 min (minor), 9.0 min (major)]. $[\alpha]^{25}_D$ = -0.9 (*c* 0.45, CH_2Cl_2). **^{1}H NMR ($CDCl_3$, 400 MHz):** δ 7.92 – 7.78 (m, 3H), 7.55 – 7.49 (m, 1H), 7.48 – 7.41 (m, 2H), 7.29 (s, 5H), 6.75 (t, *J* = 5.2 Hz, 1H), 5.21 (d, *J* = 12.4 Hz, 1H), 5.17 (d, *J* = 12.4 Hz, 1H), 3.65 (s, 3H), 3.53 – 3.40 (m, 2H), 2.67 (dd, *J* = 14.8, 6.4 Hz, 1H), 2.56 – 2.38 (m, 2H), 2.19 (dd, *J* = 14.8, 6.4 Hz, 1H), 1.60 – 1.52 (m, 1H), 0.87 (d, *J* = 7.2 Hz, 3H), 0.85 (d, *J* = 7.2 Hz, 3H). **^{13}C NMR ($CDCl_3$, 100 MHz):** δ 172.4, 170.4, 167.3, 166.1, 135.0, 133.8, 131.8, 128.6, 128.5, 128.4, 128.3, 127.1, 68.1, 66.5, 51.9, 41.1, 35.6, 33.1, 24.5, 23.5, 23.3. **IR (thin film):** ν 3378, 3065, 3033, 2957, 2925, 2871, 2852, 1738, 1659, 1580, 1506, 1476, 1443, 1368, 1261, 1216, 1178, 1076, 1028, 803, 697 cm^{-1}. **HRMS (ESI):** calcd. for $C_{25}H_{31}N_2O_6$ $(M+H)^+$ 455.2177, found 455.2175.

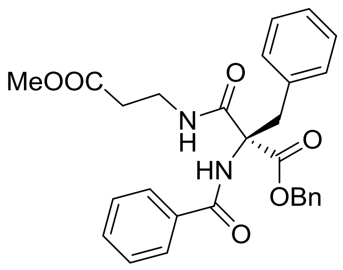

2ad

Benzyl (*R*)-2-benzamido-2-benzyl-3-((3-methoxy-3-oxopropyl)amino)-3-oxopropanoate (2ad)

Purification by flash chromatography (petroleum ether/ethyl acetate = 2/1) afforded the product as a colorless oil (80.3 mg, 82% yield). **HPLC analysis:** 97% ee [Daicel CHIRALPAK OD-H column; solvent system: 10% isopropanol/90% hexane; 1.0 mL/min; retention times: 23.5 min (minor), 35.2 min (major)]. $[\alpha]^{25}_{D}$ = -5.5 (*c* 0.80, CH_2Cl_2). **^{1}H NMR ($CDCl_3$, 400 MHz):** δ 7.76 – 7.67 (m, 2H), 7.55 – 7.38 (m, 4H), 7.38 – 7.30 (m, 5H), 7.23 – 7.10 (m, 3H), 6.97 – 6.92 (m, 2H), 6.83 (t, *J* = 5.6 Hz, 1H), 5.29 (d, *J* = 12.0 Hz, 1H), 5.22 (d, *J* = 12.0 Hz, 1H), 3.93 (d, *J* = 14.0 Hz, 1H), 3.65 (s, 3H), 3.62 (d, *J* = 14.4 Hz, 1H), 3.57 – 3.39 (m, 2H), 2.47 (t, *J* = 6.0 Hz, 2H). **^{13}C NMR ($CDCl_3$, 100 MHz):** δ 172.7, 169.6, 166.4, 166.0, 134.9, 134.9, 133.7, 131.9, 129.9, 128.6, 128.6, 128.5, 128.3, 127.2, 127.1, 68.3, 67.5, 51.9, 38.3, 35.6, 33.2. **IR (thin film):** ν 3648, 3390, 3064, 3032, 2954, 2924, 2853, 1738, 1683, 1660, 1580, 1506, 1476, 1445, 1374, 1281, 1202, 1117, 1084, 1049, 748, 701 cm^{-1}. **HRMS (ESI):** calcd. for $C_{28}H_{29}N_2O_6$ $(M+H)^+$ 489.2020, found 489.2026.

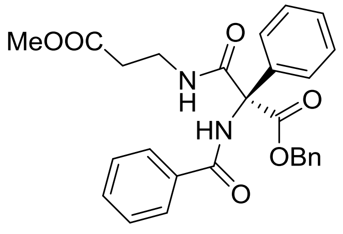

2ae

Benzyl (*R*)-2-benzamido-3-((3-methoxy-3-oxopropyl)amino)-3-oxo-2-phenylpropanoate (2ae)

Purification by flash chromatography (petroleum ether/ethyl acetate = 2/1) afforded the product as a white oil (69.5 mg, 73% yield). **HPLC analysis:** 57% ee [Daicel CHIRALPAK OD-H column; solvent system: 20% isopropanol/80% hexane; 1.0 mL/min; retention times: 10.7 min (major), 31.5 min (minor)]. $[\alpha]^{25}_{D}$ = -22.0 (*c* 0.10, CH_2Cl_2). **^{1}H NMR ($CDCl_3$, 400 MHz):** δ 7.96 (s, 1H), 7.85 – 7.80 (m, 2H), 7.62 – 7.49 (m, 4H), 7.46 – 7.40 (m, 2H), 7.37 – 7.31 (m, 3H), 7.29 – 7.26 (m, 3H), 7.24 – 7.20 (m, 2H), 5.28 (d, *J* = 12.4 Hz, 1H), 5.24 (d, *J* = 12.4 Hz, 1H), 3.61 (s, 3H), 3.59 – 3.52 (m, 2H), 2.58 – 2.44 (m, 2H). **^{13}C NMR ($CDCl_3$, 100 MHz):** δ 172.1, 169.4, 167.3, 166.5, 136.1, 134.9, 133.5, 132.0, 128.7, 128.6, 128.4, 128.3, 128.0, 127.4, 127.3, 69.6, 68.4, 51.8, 35.9, 33.4. **IR (thin film):** ν 3365, 3195, 3062, 3030, 2961, 2921, 2851, 1729, 1660, 1602, 1517, 1471, 1370, 1261, 1200, 1149, 1089, 1026, 939,

801, 697, 561 cm^{-1}. **HRMS (ESI):** calcd. for $C_{27}H_{26}N_2NaO_6$ $(M+Na)^+$ 497.1683, found 497.1698.

6. Effect of N-Acylated Catalyst

Under a N_2 atmosphere, azlactone **3** (0.2 mmol) and DIPEA (0.24 mmol) were dissolved in anhydrous toluene (1 mL) and cooled to −55 °C in a dry flask. The catalyst **OBn-DPI** (0.2 mmol) and ClCOOBn (0.24 mmol) were dissolved *in anhydrous toluene (1 mL)* in another dry flask and stirred for 10 min to form active species **A**. Then the mixture of the catalyst and ClCOOBn was transferred into the former reaction flask and the reaction mixture was stirred at −55 °C for 6 h. *C*-acylated product **4** was obtained in 82% yield (calculated from ^{1}H NMR spectra).

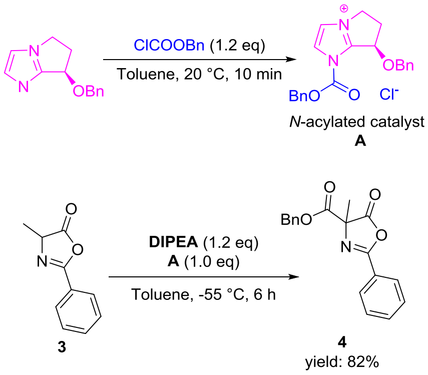

Effect of *N*-acylated catalyst

Because active species **A** can quickly turn into BnOH in the air, we made ^{1}H NMR spectra of **A** by taking catalyst **OBn-DPI** (0.1 mmol) and ClCOOBn (0.12 mmol) with *solvent* $CDCl_3$ in a NMR tube and tested this sample immediately. Then the sample was transferred into a reaction flask, which contains azlactone **3** (0.1 mmol), DIPEA (0.12 mmol) and anhydrous toluene (0.5 mL) at −55 °C. This reaction mixture was stirred at −55 °C for 6 h and the *C*-acylated product **4** was obtained in 78% yield (calculated from ^{1}H NMR spectra).

Here is the ^{1}H NMR spectra of active species **A**. **^{1}H NMR ($CDCl_3$, 400 MHz):** δ 8.10 (d, *J* = 2.1 Hz, 1H), 7.74 (d, *J* = 2.1 Hz, 1H), 7.53 – 7.48 (m, 2H), 7.44 – 7.36 (m, 4H), 7.32 – 7.29 (m, 2H), 7.19 – 7.11 (m, 2H), 5.66 (d, *J* = 6.7 Hz, 1H), 5.57 (d, *J* = 11.5 Hz, 1H), 5.49 (d, *J* = 11.5 Hz, 1H), 4.83 – 4.75 (m, 1H), 4.48 (d, *J* = 11.0 Hz, 1H), 4.43 (d, *J* = 11.0 Hz, 1H), 4.44 – 4.39 (m, 1H), 3.43 – 3.32 (m, 1H), 2.62 – 2.54 (m, 1H).

7. Synthetic Applications

Synthesis of small peptides

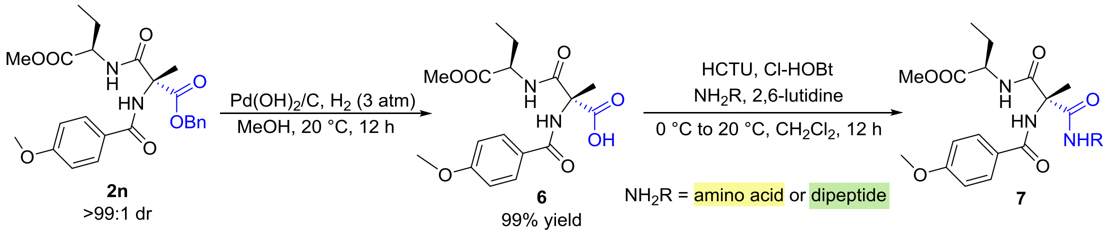

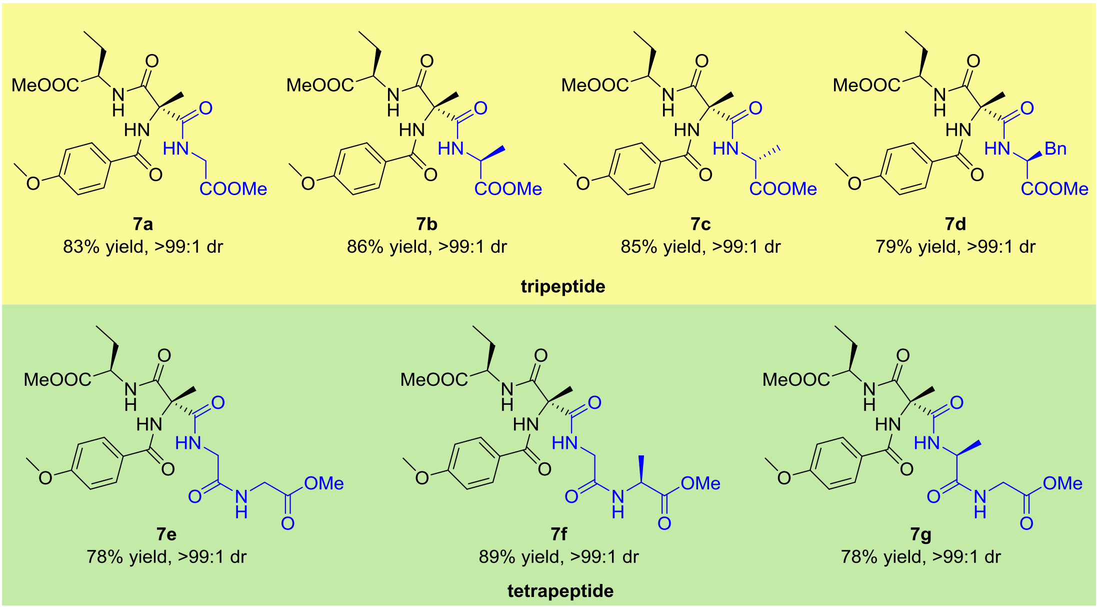

Substrate **2n** (0.78 mmol, 356 mg) and $Pd(OH)_2/C$ (274 mg) were charged in an autoclave. The system was evacuated and filled with hydrogen. Then anhydrous MeOH (10 mL) was added and the hydrogen pressure was adjusted to 3 atm. After vigorous stirring at 20 ℃ for 12 h, the reaction mixture was filtered and evaporated under reduced pressure to give **6** (285 mg, 99% yield).

Compound **6** (0.2 mmol, 73.3 mg), *O*-(6-chloro-1*H*-benzotriazol-1-yl)-1,1,3,3-tetramethyluronium hexafluorophosphate (HCTU, 83.0 mg, 0.2 mmol), 6-chloro-1-hydroxybenzotriazole (Cl-HOBt, 85.0 mg, 0.5 mmol) and 2,6-lutidine (70 μL, 0.6 mmol) were dissolved in dry dichloromethane (10 mL). The solution was cooled in an ice bath and treated with a solution of amino acid methyl ester hydrochloride (0.2 mmol) and 2,6-lutidine (24 μL, 0.2 mmol) in dichloromethane (3 mL). The reaction mixture was stirred at 0 ℃ for 2 h and gradually raised to 20 ℃ for another 12 h. Subsequently, the solution was washed thrice with 1M HCl and the aqueous phase was extracted with dichloromethane. After drying over Mg_2SO_4, the combined organic phases were filtered and evaporated under reduced pressure. The crude product was purified by flash chromatography on silica gel to give the corresponding small peptide product **7**.

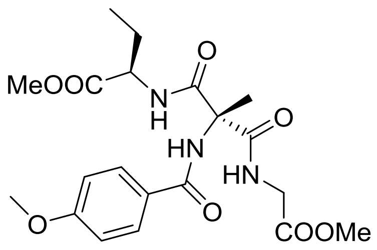

7a

Methyl (*R*)-2-((*S*)-3-((2-methoxy-2-oxoethyl)amino)-2-(4-methoxybenzamido)-2-methyl-3-oxopropanamido)butanoate (7a)

Purification by flash chromatography (petroleum ether/ethyl acetate = 1/2) afforded the product as a colorless oil (72.6 mg, 83% yield). **[α]$^{25}_{D}$** = 3.3 (*c* 1.2, CH_2Cl_2). **^{1}H NMR ($CDCl_3$, 400 MHz):** δ 7.87 – 7.81 (m, 3H), 7.69 – 7.62 (m, 1H), 7.57 (d, *J* = 7.5 Hz, 1H), 6.97 – 6.91 (m, 2H), 4.51 (td, *J* = 7.2, 5.5 Hz, 1H), 4.12 (dd, *J* = 18.1, 5.6 Hz, 1H), 4.02 (dd, *J* = 18.1, 5.2 Hz, 1H), 3.86 (s, 3H), 3.74 (s, 3H), 3.73 (s, 3H), 1.94 (s, 3H), 1.99 – 1.87 (m, 1H), 1.81 – 1.69 (m, 1H), 0.90 (t, *J* = 7.5 Hz, 3H). **^{13}C NMR ($CDCl_3$, 100 MHz):** δ 171.9, 170.6, 170.2, 169.5, 166.6, 162.8, 129.2, 125.7, 113.9, 63.3, 55.5, 54.1, 52.4, 52.4, 41.8, 25.2, 23.0, 9.5. **IR (thin film):** ν 3353, 3061, 2957, 2927, 2850, 1747, 1682, 1647, 1607, 1505, 1441, 1373, 1295, 1259, 1212, 1179, 1110, 1026, 983, 848, 795, 595 cm^{-1}. **HRMS (ESI):** calcd. for $C_{20}H_{27}N_3NaO_8$ $(M+Na)^+$ 460.1690, found 460.1695.

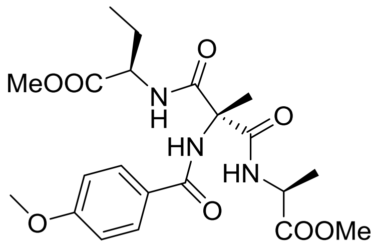

7b

Methyl (*R*)-2-((*S*)-3-(((*S*)-1-methoxy-1-oxopropan-2-yl)amino)-2-(4-methoxybenzamido)-2-methyl-3-oxopropanamido)butanoate (7b)

Purification by flash chromatography (petroleum ether/ethyl acetate = 1/2) afforded the product as a colorless oil (77.8 mg, 86% yield). **[α]$^{25}_{D}$** = -1.0 (*c* 0.6, CH_2Cl_2). **^{1}H NMR ($CDCl_3$, 400 MHz):** δ 7.87 – 7.83 (m, 2H), 7.82 (s, 1H), 7.53 (d, *J* = 7.1 Hz, 1H), 7.45 (d, *J* = 7.6 Hz, 1H), 6.97 – 6.92 (m, 2H), 4.62 – 4.47 (m, 2H), 3.86 (s, 3H), 3.74 (s, 3H), 3.72 (s, 3H), 1.98 – 1.89 (m, 1H), 1.93 (s, 3H), 1.79 – 1.68 (m, 1H), 1.43 (d, *J* = 7.2 Hz, 3H), 0.88 (t, *J* = 7.5 Hz, 3H). **^{13}C NMR ($CDCl_3$, 100 MHz):** δ 172.6, 171.9, 170.2, 169.6, 166.4, 162.7, 129.2, 125.8, 113.8, 63.2, 55.5, 53.9, 52.5, 52.4, 48.8, 25.2, 22.7, 17.7, 9.5. **IR (thin film):** ν 3379, 2955, 2878, 2847, 1744, 1661, 1607, 1506, 1376, 1296, 1257, 1211, 1178, 1029, 847, 772, 602 cm^{-1}. **HRMS (ESI):** calcd. for $C_{21}H_{29}N_3NaO_8$ $(M+Na)^+$ 474.1847, found 474.1850.

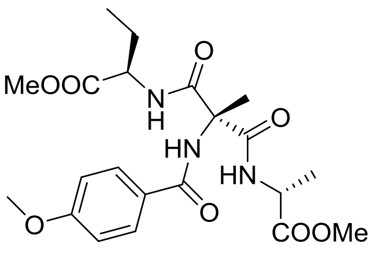

7c

Methyl (*R*)-2-((*S*)-3-(((*R*)-1-methoxy-1-oxopropan-2-yl)amino)-2-(4-methoxybenzamido)-2-methyl-3-oxopropanamido)butanoate (7c)

Purification by flash chromatography (petroleum ether/ethyl acetate = 1/2) afforded the product as a white solid (76.5 mg, 85% yield). **M.p.:** 135.2-136.0 ℃. **[α]$^{25}_{D}$** = -10.3 (*c* 0.7, CH_2Cl_2). **^{1}H NMR ($CDCl_3$, 400 MHz):** δ 7.90 (s, 1H), 7.87 – 7.81 (m, 2H), 7.59 (d, *J* = 7.0 Hz, 1H), 7.54 (d, *J* = 7.7 Hz, 1H), 6.96 – 6.91 (m, 2H), 4.60 – 4.48 (m, 2H), 3.86 (s, 3H), 3.72 (s, 3H), 3.72 (s, 3H), 1.99 – 1.88 (m, 1H), 1.93 (s, 3H), 1.81 – 1.69 (m, 1H), 1.43 (d, *J* = 7.2 Hz, 3H), 0.90 (t, *J* = 7.5 Hz, 3H). **^{13}C NMR ($CDCl_3$, 100 MHz):** δ 172.5, 171.8, 170.0, 169.8, 166.3, 162.7, 129.1, 125.8, 113.8, 63.3, 55.4, 54.0, 52.5, 52.4, 48.8, 25.2, 22.7, 17.9, 9.5. **IR (thin film):** ν 3481, 2962, 2925, 2854, 1747, 1681, 1647, 1608, 1507, 1455, 1376, 1263, 1209, 1030, 798, 601 cm^{-1}. **HRMS (ESI):** calcd. for $C_{21}H_{29}N_3NaO_8$ $(M+Na)^+$ 474.1847, found 474.1848.

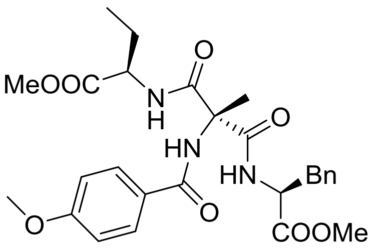

7d

Methyl (*R*)-2-((*R*)-3-(((*S*)-1-methoxy-1-oxo-3-phenylpropan-2-yl)amino)-2-(4-methoxybenzamido)-2-methyl-3-oxopropanamido)butanoate (7d)

Purification by flash chromatography (petroleum ether/ethyl acetate = 1/1) afforded the product as a colorless oil (83.3 mg, 79% yield). **[α]$^{25}_{D}$** = 29.5 (*c* 1.2, CH_2Cl_2). **^{1}H NMR ($CDCl_3$, 400 MHz):** δ 7.86 – 7.80 (m, 2H), 7.78 – 7.75 (m, 1H), 7.40 (d, *J* = 7.1 Hz, 2H), 7.18 – 7.09 (m, 5H), 6.97 – 6.92 (m, 2H), 4.84 (dt, *J* = 7.5, 6.0 Hz, 1H), 4.47 (td, *J* = 7.1, 5.6 Hz, 1H), 3.87 (s, 3H), 3.71 (s, 3H), 3.67 (s, 3H), 3.14 (dd, *J* = 13.7, 6.1 Hz, 1H), 3.09 (dd, *J* = 13.7, 5.6 Hz, 1H), 1.90 (s, 3H), 1.88 – 1.80 (m, 1H), 1.77 – 1.65 (m, 1H), 0.86 (t, *J* = 7.5 Hz, 3H). **^{13}C NMR ($CDCl_3$, 100 MHz):** δ 171.8, 171.2, 169.8, 169.8, 166.2, 162.8, 135.5, 129.2, 129.2, 128.6, 127.1, 125.7, 113.8, 63.3, 55.5, 54.0, 53.8, 52.4, 52.3, 37.7, 25.2, 22.7, 9.5. **IR (thin film):** ν 3374, 3062, 3030, 2954, 2879, 2842, 1747, 1689, 1608, 1516, 1362, 1295, 1258, 1212, 1029, 847, 772, 736, 702, 595 cm^{-1}. **HRMS (ESI):** calcd. for $C_{27}H_{33}N_3NaO_8$ $(M+Na)^+$ 550.2160, found 550.2163.

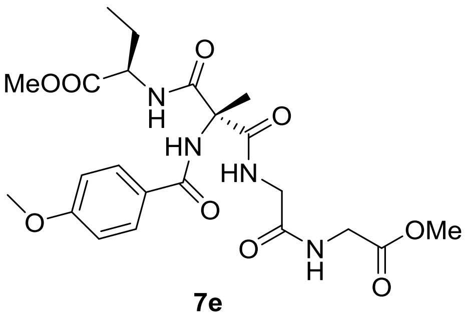

7e

Methyl (*R*)-2-((*S*)-3-((2-((2-methoxy-2-oxoethyl)amino)-2-oxoethyl)amino)-2-(4-methoxybenzamido)-2-methyl-3-oxopropanamido)butanoate (7e)

Purification by flash chromatography (petroleum ether/ethyl acetate = 1/7) afforded the product as a white solid (77.0 mg, 78% yield). **M.p.:** 179.0-179.8 ℃. **[α]$^{25}_{D}$** = -6.6 (*c* 0.7, CH_2Cl_2). **^{1}H NMR ($CDCl_3$, 400 MHz):** δ 7.98 (s, 1H), 7.85 – 7.79 (m, 2H), 7.66 – 7.59 (m, 1H), 7.59 – 7.52 (m, 1H), 7.34 (d, *J* = 7.7 Hz, 1H), 6.97 – 6.90 (m, 2H), 4.46 (td, *J* = 7.5, 5.2 Hz, 1H), 4.27 (dd, *J* = 17.2, 7.5 Hz, 1H), 4.17 (dd, *J* = 17.7, 6.4 Hz, 1H), 3.93 (dd, *J* = 17.7, 5.4 Hz, 1H), 3.86 (s, 3H), 3.80 (dd, *J* = 17.2, 5.2 Hz, 1H), 3.73 (s, 3H), 3.69 (s, 3H), 1.99 – 1.92 (m, 1H), 1.92 (s, 3H), 1.81 – 1.71 (m, 1H), 0.93 (t, *J* = 7.5 Hz, 3H). **^{13}C NMR ($CDCl_3$, 100 MHz):** δ 171.8, 170.6, 170.2, 169.6, 169.2, 167.2, 163.1, 129.3, 125.0, 113.9, 63.3, 55.5, 54.1, 52.5, 52.1, 43.4, 41.0, 25.0, 22.8, 9.6. **IR (thin film):** ν 3648, 3481, 2955, 2921, 2850, 1747, 1660, 1647, 1607, 1506, 1296, 1259, 1211, 1184, 1082, 1026, 970, 844, 601 cm^{-1}. **HRMS (ESI):** calcd. for $C_{22}H_{30}N_4NaO_9$ $(M+Na)^+$ 517.1905, found 517.1904.

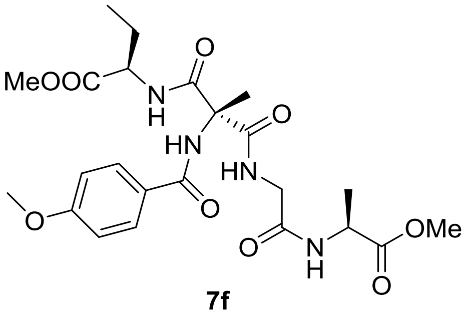

7f

Methyl (*R*)-2-((*S*)-3-((2-(((*S*)-1-methoxy-1-oxopropan-2-yl)amino)-2-oxoethyl)amino)-2-(4-methoxybenzamido)-2-methyl-3-oxopropanamido)butanoate (7f)

Purification by flash chromatography (ethyl acetate) afforded the product as a white solid (90.2 mg, 89% yield). **M.p.:** 152.5-153.5 ℃. **[α]$^{25}_{D}$** = 20.7 (*c* 0.8, CH_2Cl_2). **^{1}H NMR ($CDCl_3$, 400 MHz):** δ 7.98 (s, 1H), 7.84 (d, *J* = 8.7 Hz, 2H), 7.56 (d, *J* = 7.1 Hz, 1H), 7.54 – 7.48 (m, 1H), 7.28 (s, 1H), 6.95 (d, *J* = 8.7 Hz, 2H), 4.59 – 4.50 (m, 1H), 4.47 (td, *J* = 7.5, 5.4 Hz, 1H), 4.39 (dd, *J* = 17.2, 8.0 Hz, 1H), 3.87 (s, 3H), 3.74 (s, 3H), 3.68 (s, 3H), 3.64 (dd, *J* = 17.4, 4.8 Hz, 1H), 2.00 – 1.93 (m, 1H), 1.92 (s, 3H), 1.83 – 1.71 (m, 1H), 1.52 (d, *J* = 7.3 Hz, 3H), 0.93 (t, *J* = 7.4 Hz, 3H). **^{13}C NMR ($CDCl_3$, 100 MHz):** δ 173.5, 171.7, 170.3, 169.6, 168.6, 167.0, 163.1, 129.4, 125.1, 114.0, 63.3, 55.5, 54.1, 52.5, 52.3, 48.2, 43.3, 25.0, 22.8, 17.2, 9.6. **IR (thin film):** ν 3314, 3062, 2955, 2924, 2851, 1743, 1646, 1607, 1538, 1505, 1376, 1259, 1211, 1161,

1109, 1025, 849, 798, 736, 602 cm^{-1}. **HRMS (ESI):** calcd. for $C_{23}H_{32}N_4NaO_9$ $(M+Na)^+$ 531.2061, found 531.2068.

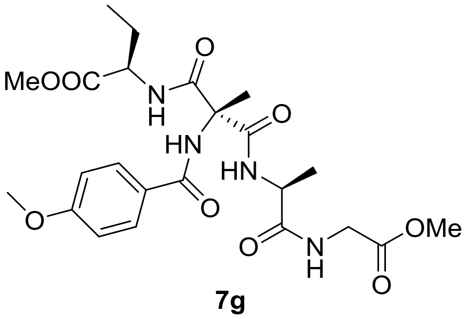

7g

Methyl (*R*)-2-((*S*)-3-(((*S*)-1-((2-methoxy-2-oxoethyl)amino)-1-oxopropan-2-yl)amino)-2-(4-methoxybenzamido)-2-methyl-3-oxopropanamido)butanoate (7g)

Purification by flash chromatography (petroleum ether/ethyl acetate = 1/20) afforded the product as a colorless oil (79.6 mg, 78% yield). $[\alpha]^{25}_D$ = -5.3 (*c* 0.3, CH_2Cl_2). **^{1}H NMR ($CDCl_3$, 400 MHz):** δ 7.87 – 7.81 (m, 2H), 7.76 (s, 1H), 7.41 (d, *J* = 7.5 Hz, 1H), 7.09 (d, *J* = 7.3 Hz, 1H), 7.07 – 7.02 (m, 1H), 6.98 – 6.92 (m, 2H), 4.57 – 4.49 (m, 1H), 4.47 (td, *J* = 7.6, 5.0 Hz, 1H), 4.07 (dd, *J* = 17.9, 5.8 Hz, 1H), 3.96 (dd, *J* = 17.9, 5.5 Hz, 1H), 3.87 (s, 3H), 3.71 (s, 3H), 3.70 (s, 3H), 1.98 – 1.89 (m, 1H), 1.93 (s, 3H), 1.80 – 1.72 (m, 1H), 1.41 (d, *J* = 7.1 Hz, 3H), 0.94 (t, *J* = 7.5 Hz, 3H). **^{13}C NMR ($CDCl_3$, 100 MHz):** δ 172.4, 171.8, 170.5, 170.4, 169.8, 166.4, 162.9, 129.2, 125.4, 114.0, 63.4, 55.5, 54.1, 52.4, 52.2, 49.7, 41.2, 24.8, 22.7, 17.1, 9.8. **IR (thin film):** ν 3840, 3740, 3352, 2954, 2925, 2854, 1744, 1659, 1607, 1506, 1458, 1376, 1259, 1209, 1181, 1098, 1026, 848, 795, 701, 594 cm^{-1}. **HRMS (ESI):** calcd. for $C_{23}H_{32}N_4NaO_9$ $(M+Na)^+$ 531.2061, found 531.2061.

Synthesis of α-Alkyl Serine:

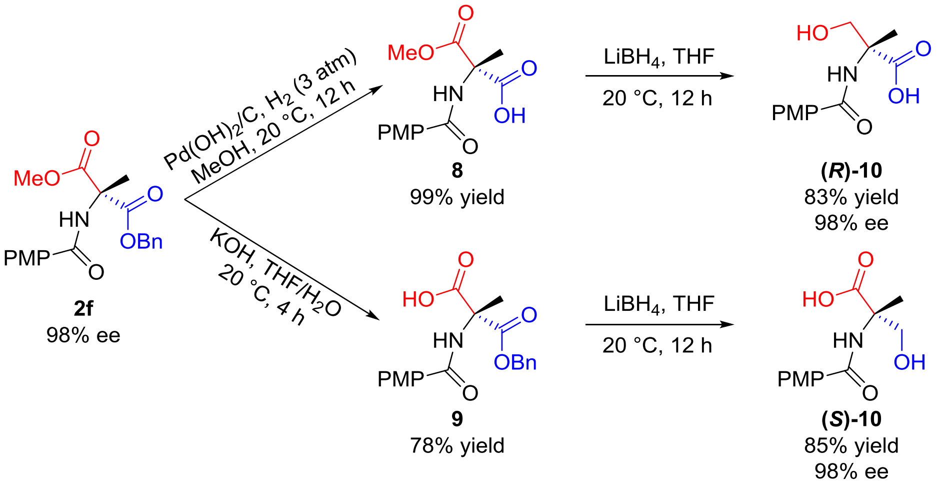

Substrate **2f** (0.41 mmol, 151 mg) and $Pd(OH)_2/C$ (100 mg) were charged in an autoclave. The system was evacuated and filled with hydrogen. Then anhydrous MeOH (2 mL) was added and the hydrogen pressure was adjusted to 3 atm. After vigorous stirring at 20 ℃ for 12 h, the reaction mixture was filtered and evaporated

under reduced pressure to give **8** (113 mg, 99% yield). **^{1}H NMR ($CDCl_3$, 400 MHz):** δ 7.77 (d, *J* = 8.7 Hz, 2H), 7.62 (s, 1H), 6.90 (d, *J* = 8.7 Hz, 2H), 3.83 (s, 3H), 3.80 (s, 3H), 1.84 (s, 3H). **^{13}C NMR ($CDCl_3$, 100 MHz):** δ 171.1, 170.1, 166.8, 162.8, 129.2, 124.9, 113.8, 63.2, 55.4, 53.6, 21.1.

8 (0.4 mmol, 113 mg) was dissolved in anhydrous THF (5 mL) in a dry two-necked flask at 20 ℃, $LiBH_4$ (2 M solution in THF, 0.6 mmol, 0.3 mL) was then added dropwise. The reaction mixture was stirred at 20 ℃ for 12 h. The solvent was acidized with 0.1 M HCl (8 mL) and extracted with EtOAc (10 mL × 4). The crude product was purified by flash chromatography on silica gel to give **(*R*)-10**.

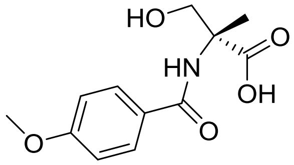

(*R*)-10

(*R*)-3-Hydroxy-2-(4-methoxybenzamido)-2-methylpropanoic acid ((*R*)-10)

Purification by flash chromatography (ethyl acetate/methanol = 5/1, added 0.5% AcOH) afforded the product as a white solid (71.2 mg, 83% yield). **M.p.:** 49.5-50.5 ℃. **HPLC analysis:** 98% ee [Daicel CHIRALPAK AS-H column; solvent system: 5% ethanol/95% hexane, added 0.5% AcOH; 0.5 mL/min; retention times: 46.7 min (minor), 52.1 min (major)]. **$[\alpha]^{25}_D$** = -0.6 (*c* 0.3, MeOH). **^{1}H NMR ($CDCl_3$, 400 MHz):** δ 7.74 (d, *J* = 8.7 Hz, 2H), 7.28 (s, 1H), 6.87 (d, *J* = 8.8 Hz, 2H), 6.77 (brs, 1H), 4.05 – 3.95 (m, 2H), 3.81 (s, 3H), 1.60 (s, 3H). **^{13}C NMR ($CDCl_3$, 100 MHz):** δ 175.4, 168.7, 162.7, 129.2, 125.5, 113.8, 66.2, 62.0, 55.4, 19.7. **IR (thin film):** ν 3411, 2955, 2917, 2849, 1715, 1640, 1608, 1540, 1504, 1463, 1305, 1259, 1180, 1120, 1052, 1030, 845, 769, 599 cm^{-1}. **HRMS (ESI):** calcd. for $C_{12}H_{15}NNaO_5$ $(M+Na)^+$ 276.0842, found 276.0847.

Substrate **2f** (0.43 mmol, 160 mg) was dissolved in THF (5 mL) in two-necked flask at 20 ℃. KOH (0.43 mmol, 24.1 mg) was dissolved in H_2O (5 mL) and added dropwise into the flask. The reaction mixture was stirred at 20 ℃ for 4 h. The solvent was acidized with 0.1 M HCl to PH < 6 and extracted with EtOAc (10 mL × 4). The crude product was purified by flash chromatography on silica gel (ethyl acetate/methanol = 10/1, added 0.5% AcOH) to give **9** (120.5 mg, 78% yield). **^{1}H NMR ($CDCl_3$, 400 MHz):** δ 7.75 (d, *J* = 8.7 Hz, 2H), 7.47 (s, 1H), 7.29 (s, 5H), 6.89 (d, *J* = 8.7 Hz, 2H), 5.24 (s, 2H), 3.83 (s, 3H), 1.86 (s, 3H). **^{13}C NMR ($CDCl_3$, 100 MHz):** δ 170.9, 169.5, 167.0, 162.8, 134.8, 129.2, 128.5, 128.4, 128.0, 124.8, 113.9, 68.3, 63.4, 55.4, 21.2.

9 (0.2 mmol, 71.5 mg) was dissolved in anhydrous THF (3 mL) in a dry two-necked flask at 20 ℃, $LiBH_4$ (2M solution in THF, 0.3 mmol, 0.15 mL) was then added dropwise. The reaction mixture was stirred at 20 ℃ for 12 h. The solvent was acidized with 0.1 M HCl (5 mL) and extracted with EtOAc (6 mL × 4). The crude product was purified by flash chromatography on silica gel to give **(*S*)-10**.

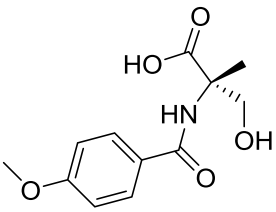

(*S*)-10

(*S*)-3-Hydroxy-2-(4-methoxybenzamido)-2-methylpropanoic acid ((*S*)-10)

Purification by flash chromatography (ethyl acetate/methanol = 5/1, added 0.5% AcOH) afforded the product as a white solid (43.1 mg, 85% yield). **M.p.:** 49.5-50.5 ℃. **HPLC analysis:** 98% ee [Daicel CHIRALPAK AS-H column; solvent system: 5% ethanol/95% hexane, added 0.5% AcOH; 0.5 mL/min; retention times: 46.1 min (major), 53.9 min (minor)]. $[\alpha]^{25}_{D}$ = 0.7 (*c* 0.3, MeOH). **^{1}H NMR ($CDCl_3$, 400 MHz):** δ 7.74 (d, *J* = 8.7 Hz, 2H), 7.28 (s, 1H), 6.87 (d, *J* = 8.8 Hz, 2H), 6.77 (brs, 1H), 4.05 – 3.95 (m, 2H), 3.81 (s, 3H), 1.60 (s, 3H). **^{13}C NMR ($CDCl_3$, 100 MHz):** δ 175.4, 168.7, 162.7, 129.2, 125.5, 113.8, 66.2, 62.0, 55.4, 19.7. **HRMS (ESI):** calcd. for $C_{12}H_{15}NNaO_5$ $(M+Na)^+$ 276.0842, found 276.0847.

Synthesis of Substituted 2-Oxazolines:

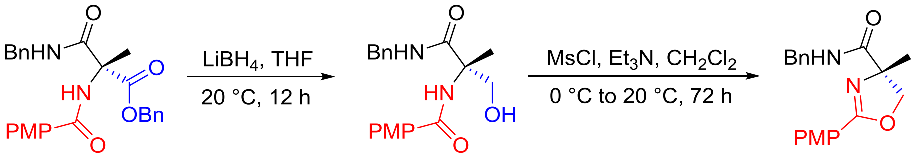

2a
99% ee

11
79% yield

12
94% yield
98% ee

Under a N_2 atmosphere, the compound **2a** (0.35 mmol, 156.3 mg) was dissolved in anhydrous THF (6 mL) in a dry two-necked flask at 20 ℃. $LiBH_4$ (2 M solution in THF, 0.53 mmol, 0.26 mL) was then added dropwise. The reaction mixture was stirred at 20 ℃ for 12 h. The solvent was acidized with 0.1 M HCl (6 mL) and extracted with EtOAc (10 mL × 4). The crude product was purified by flash chromatography on silica gel (petroleum ether/ethyl acetate = 1/2, added 0.5% AcOH) to give **11** (94.6 mg, 79% yield). **^{1}H NMR ($CDCl_3$, 400 MHz):** δ 8.11 – 8.04 (m, 1H), 7.78 – 7.72 (m, 2H), 7.35 – 7.28 (m, 3H), 7.28 – 7.23 (m, 2H), 6.96 – 6.89 (m, 2H), 4.53 – 4.42 (m, 2H), 4.28 – 4.20 (m, 1H), 3.86 (s, 3H), 3.68 – 3.61 (m, 1H), 1.69 (s, 3H). **^{13}C NMR ($CDCl_3$, 100 MHz):** δ 173.9, 168.2, 162.6, 138.0, 129.0, 128.7, 127.4, 127.3, 126.3, 113.9, 67.7, 61.1, 55.5, 43.5, 20.2. **HRMS (ESI):** calcd. for $C_{19}H_{23}N_2O_4$ $(M+H)^+$ 343.1652, found 343.1649.

Under a N_2 atmosphere, the compound **11** (0.16 mmol, 55.0 mg) and Et_3N (0.4 mmol, 56 μL) was dissolved in DCM (10 mL) in a dry two-necked flask and cooled to 0 ℃. MsCl (0.4 mmol, 31 μL) was added dropwise at 0 ℃. Then the ice bath was removed, and the mixture was stirred at 20 ℃ for 72 hours. The solvent was acidized with 0.1 M HCl (5 mL) and extracted with EtOAc (8 mL × 4). The combined organic phases were dried over Na_2SO_4. After filtration, the residue was purified by column chromatography to give **12**.

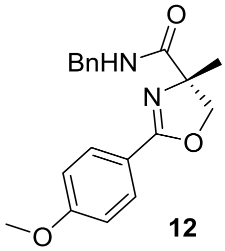

12

(*S*)-*N*-Benzyl-2-(4-methoxyphenyl)-4-methyl-4,5-dihydrooxazole-4-carboxamide (12)

Purification by flash chromatography (petroleum ether/ethyl acetate = 2/1) afforded the product as a white oil (49.2 mg, 94% yield). **HPLC analysis:** 98% ee [Daicel CHIRALPAK AS-H column; solvent system: 20% isopropanol/80% hexane; 0.5 mL/min; retention times: 15.9 min (minor), 20.2 min (major)]. **$[\alpha]^{25}_{D}$** = -15.8 (*c* 1.0, CH_2Cl_2). **^1H NMR ($CDCl_3$, 400 MHz):** δ 7.88 (d, *J* = 8.9 Hz, 2H), 7.36 – 7.30 (m, 2H), 7.30 – 7.24 (m, 3H), 7.18 – 7.09 (m, 1H), 6.91 (d, *J* = 8.9 Hz, 2H), 4.67 (d, *J* = 8.9 Hz, 1H), 4.57 (dd, *J* = 14.9, 6.5 Hz, 1H), 4.37 (dd, *J* = 15.0, 5.5 Hz, 1H), 4.27 (d, *J* = 8.9 Hz, 1H), 3.85 (s, 3H), 1.61 (s, 3H). **^{13}C NMR ($CDCl_3$, 100 MHz):** δ 175.1, 164.4, 162.5, 138.1, 130.2, 128.7, 127.6, 127.4, 119.6, 113.8, 76.5, 74.6, 55.4, 43.0, 26.7. **IR (thin film):** ν 3840, 3739, 3648, 3393, 3064, 3031, 2960, 2928, 2867, 1724, 1668, 1641, 1610, 1579, 1512, 1455, 1423, 1354, 1307, 1258, 1171, 1076, 1029, 841, 745, 699, 685 cm^{-1}. **HRMS (ESI):** calcd. for $C_{19}H_{21}N_2O_3$ $(M+H)^+$ 325.1547, found 325.1543.

8. Reference

1. Z. Zhang, F. Xie, J. Jia and W. Zhang, *J. Am. Chem. Soc.*, 2010, **132**, 15939-15941.
2. A. S. De Miranda, J. C. Gomes, M. T. Rodrigues, I. C. R. Costa, W. P. Almeida, R. d. O. Lopes, L. S. M. Miranda, F. Coelho and R. O. M. A. de Souza, *J. Mol. Catal. B: Enzym.*, 2013, **91**, 77-80.
3. M. Wang, Z. Zhang, S. Liu, F. Xie and W. Zhang, *Chem. Commun.*, 2014, **50**, 1227-1230.
4. M. Wang, X. Zhang, Z. Ling, Z. Zhang and W. Zhang, *Chem. Commun.*, 2017, **53**, 1381-1384.
5. J. C. Ruble and G. C. Fu, *J. Am. Chem. Soc.*, 1998, **120**, 11532-11533.
6. E. Badiola, B. Fiser, E. Gómez-Bengoa, A. Mielgo, I. Olaizola, I. Urruzuno, J. M. García, J. M. Odriozola, J. Razkin, M. Oiarbide and C. Palomo, *J. Am. Chem. Soc.*, 2014, **136**, 17869-17881.
7. S. Li, W. Zhu, F. Gao, C. Li, J. Wang and H. Liu, *J. Org. Chem.*, 2017, **82**, 126-134.
8. M. Weber, S. Jautze, W. Frey and R. Peters, *Chem. - Eur. J.*, 2012, **18**, 14792-14804.
9. M. Weber, W. Frey and R. Peters, *Angew. Chem. Int. Ed.*, 2013, **52**, 13223-13227.

9. NMR Spectra

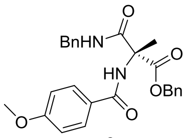

2a

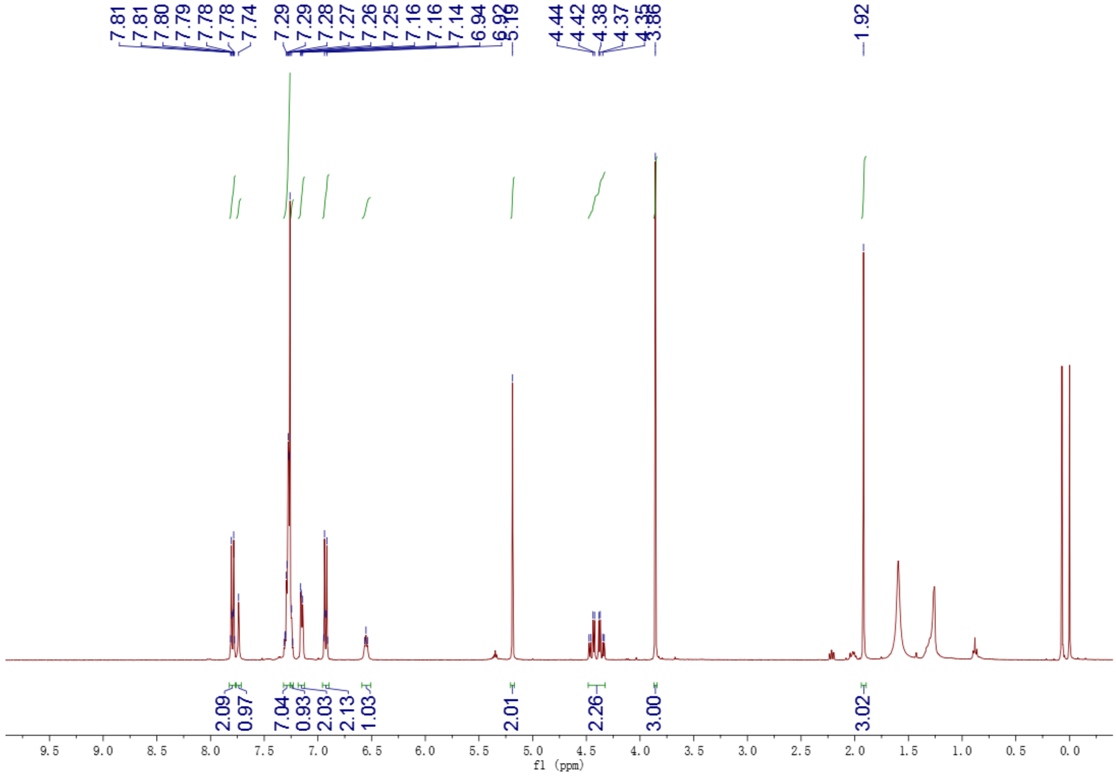

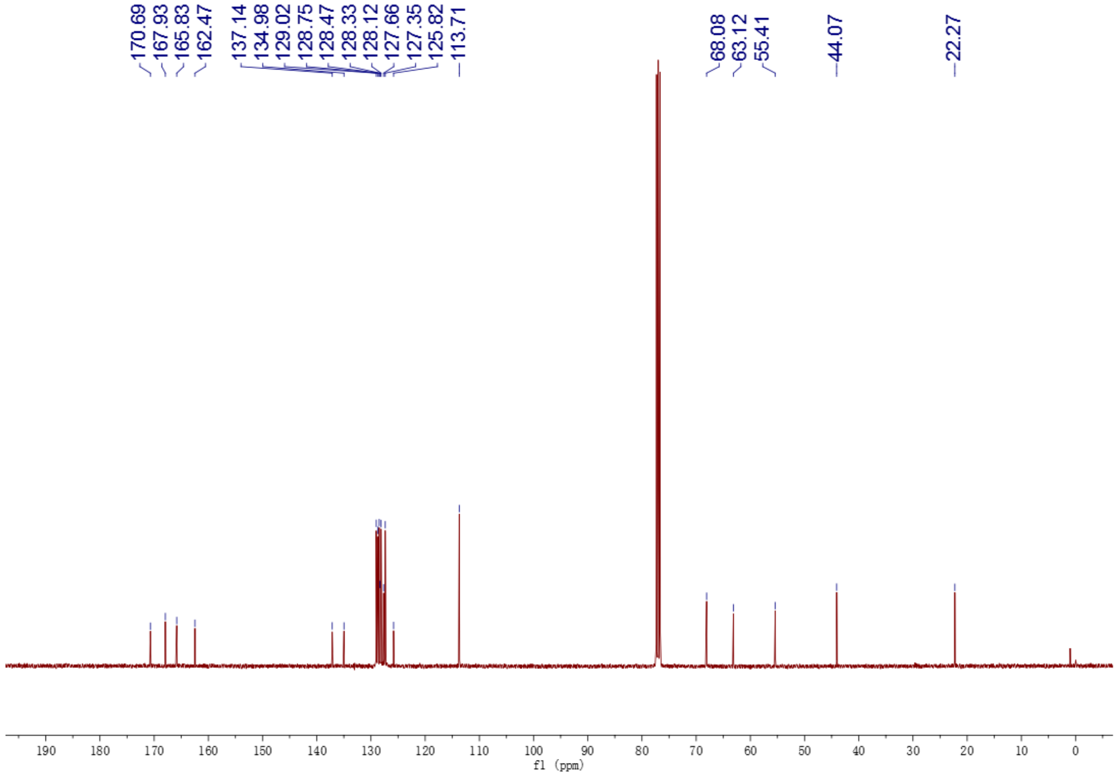

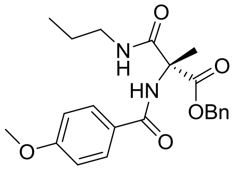
O
N
H
O
HN
OBn
O
O

2b

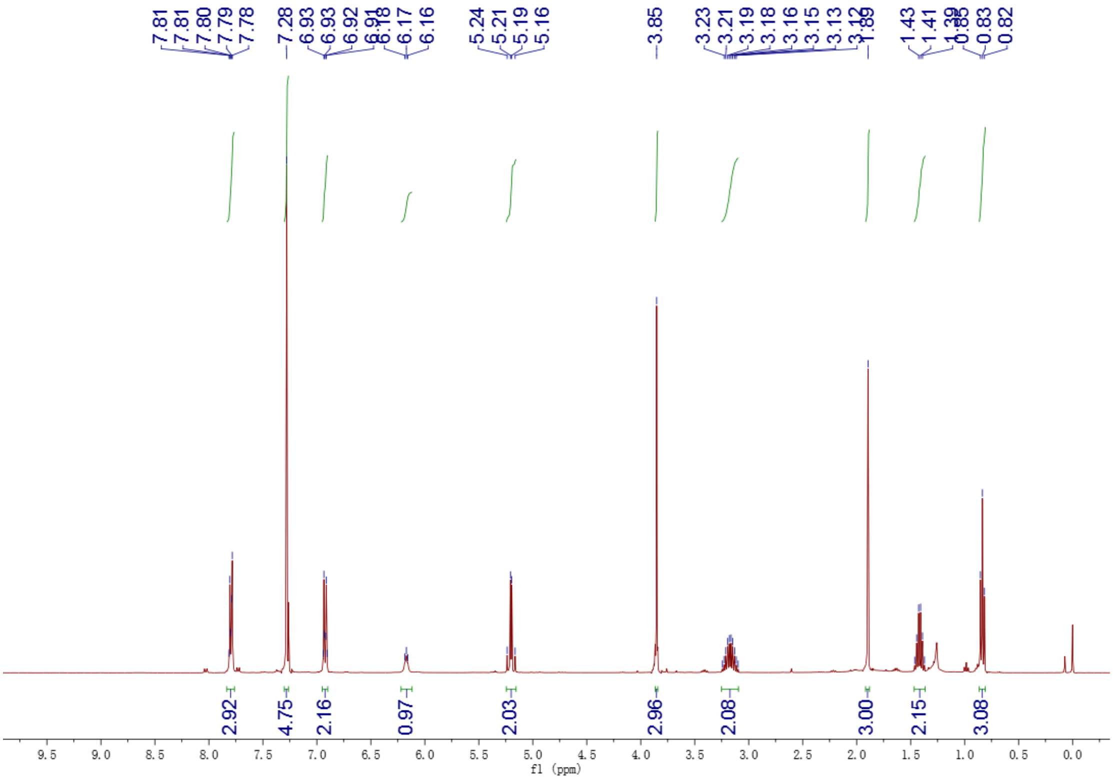
7.81
7.81
7.80
7.79
7.78
7.28
6.93
6.93
6.92
6.91
6.18
6.17
6.16
5.24
5.21
5.19
5.16
3.85
3.23
3.21
3.19
3.18
3.16
3.15
3.13
3.12
1.89
1.43
1.41
1.39
0.85
0.83
0.82
2.92
4.75
2.16
0.97
2.03
2.96
2.08
3.00
2.15
3.08
9.5 9.0 8.5 8.0 7.5 7.0 6.5 6.0 5.5 5.0 4.5 4.0 3.5 3.0 2.5 2.0 1.5 1.0 0.5 0.0
f1 (ppm)

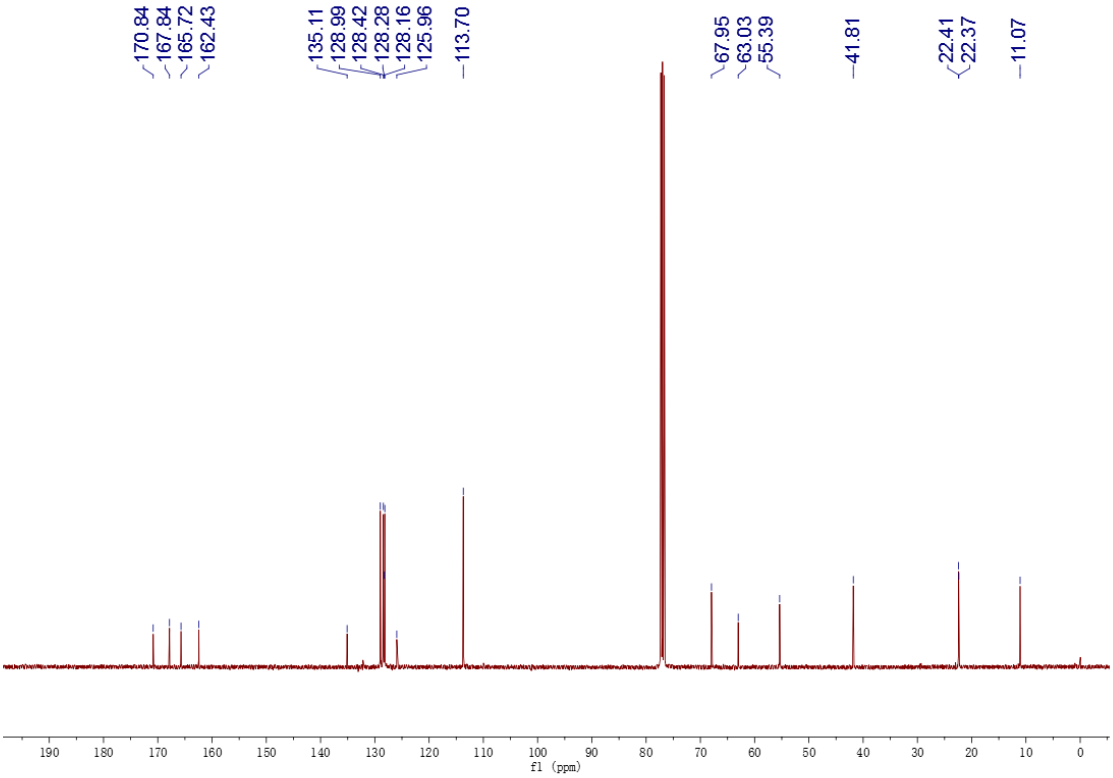
170.84
167.84
165.72
162.43
135.11
128.99
128.42
128.28
128.16
125.96
113.70
67.95
63.03
55.39
41.81
22.41
22.37
11.07
190 180 170 160 150 140 130 120 110 100 90 80 70 60 50 40 30 20 10 0
f1 (ppm)

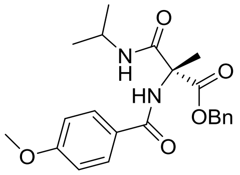
O
N
H
HN
O
OBn
O
O

2c

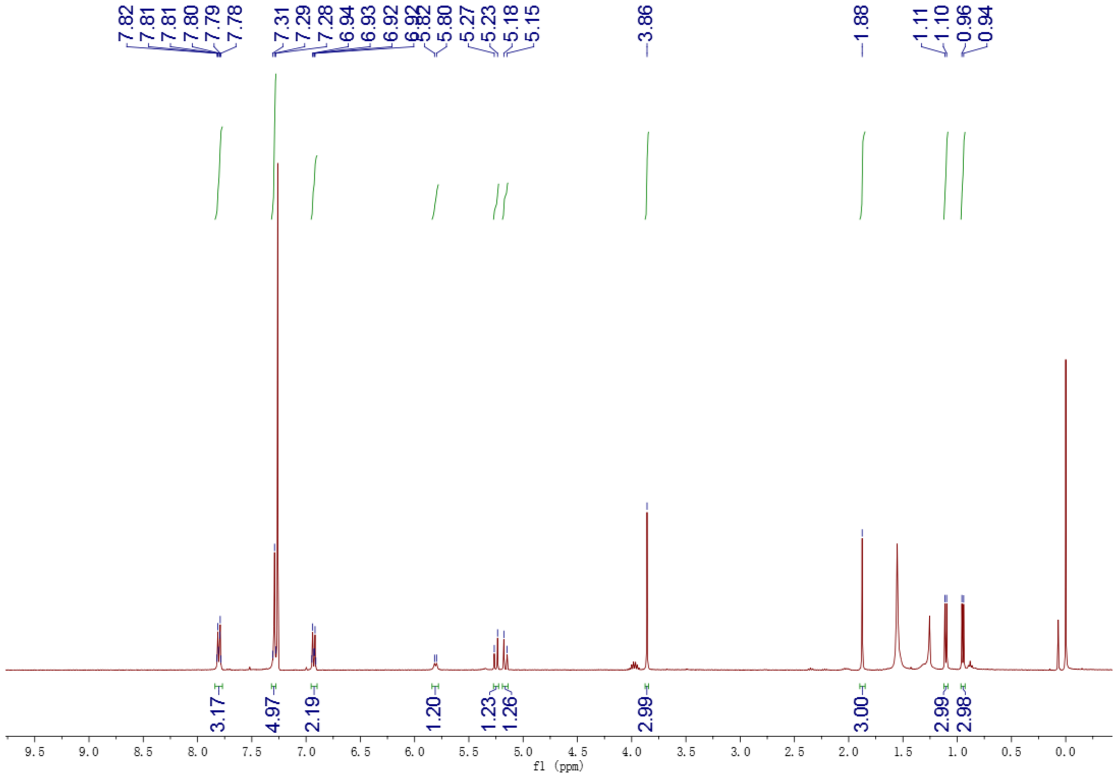
7.82
7.81
7.81
7.80
7.79
7.78
7.31
7.29
7.28
6.94
6.93
6.92
6.92
5.82
5.80
5.27
5.23
5.18
5.15
3.86
1.88
1.11
1.10
0.96
0.94
3.17
4.97
2.19
1.20
1.23
1.26
2.99
3.00
2.99
2.98
f1 (ppm)

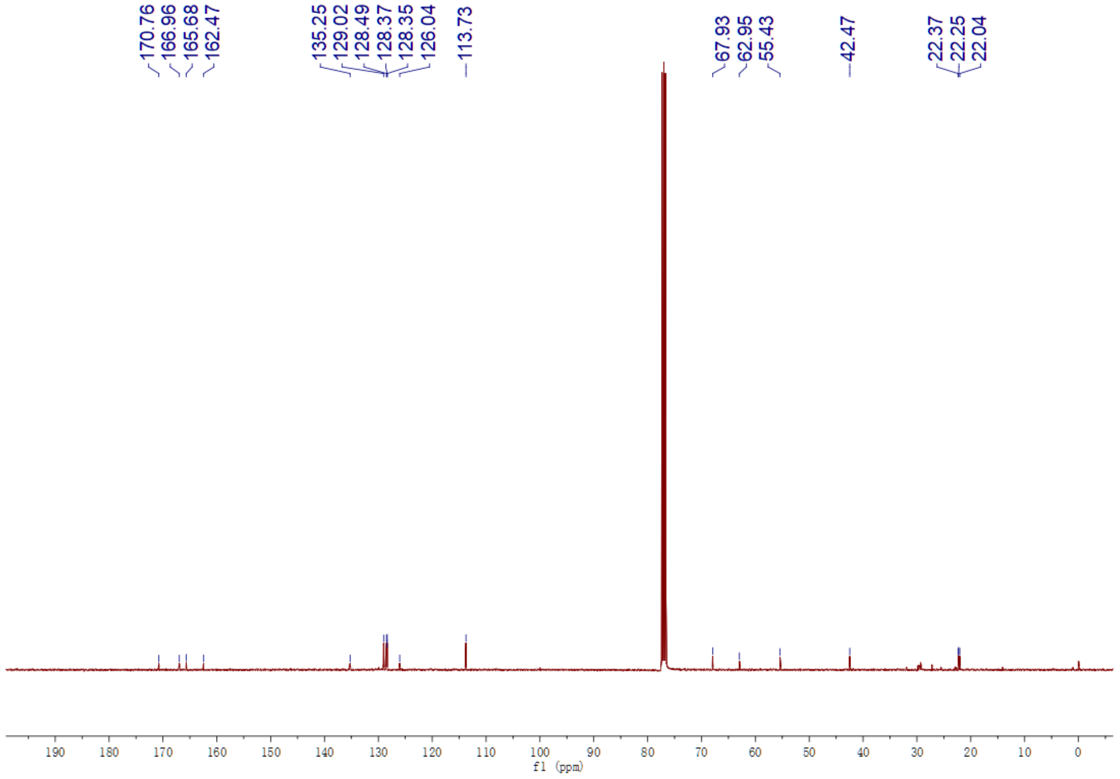
170.76
166.96
165.68
162.47
135.25
129.02
128.49
128.37
128.35
126.04
113.73
67.93
62.95
55.43
42.47
22.37
22.25
22.04
f1 (ppm)

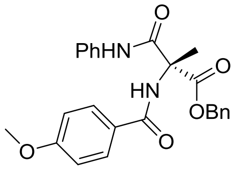
PhHN
O
O
HN
OBn
O
O

2d

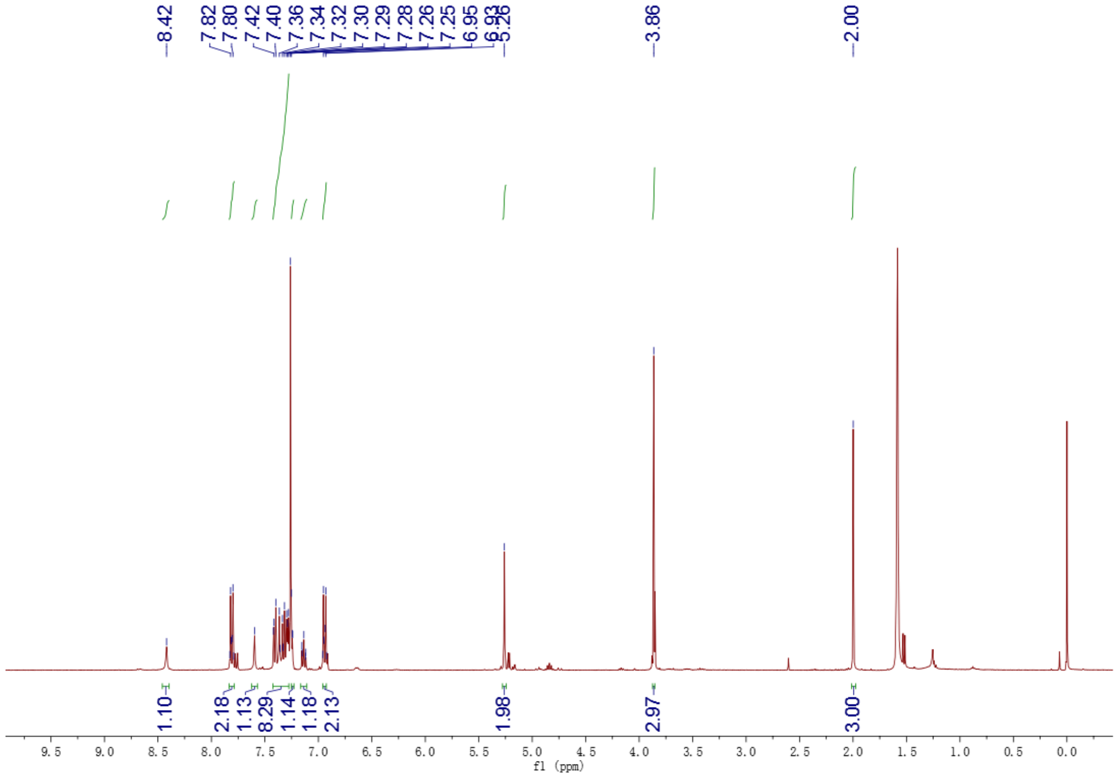
8.42
7.82
7.80
7.42
7.40
7.36
7.34
7.32
7.30
7.29
7.28
7.26
7.25
6.95
6.93
5.26
3.86
2.00
1.10
2.18
1.13
8.29
1.14
1.18
2.13
1.98
2.97
3.00
f1 (ppm)

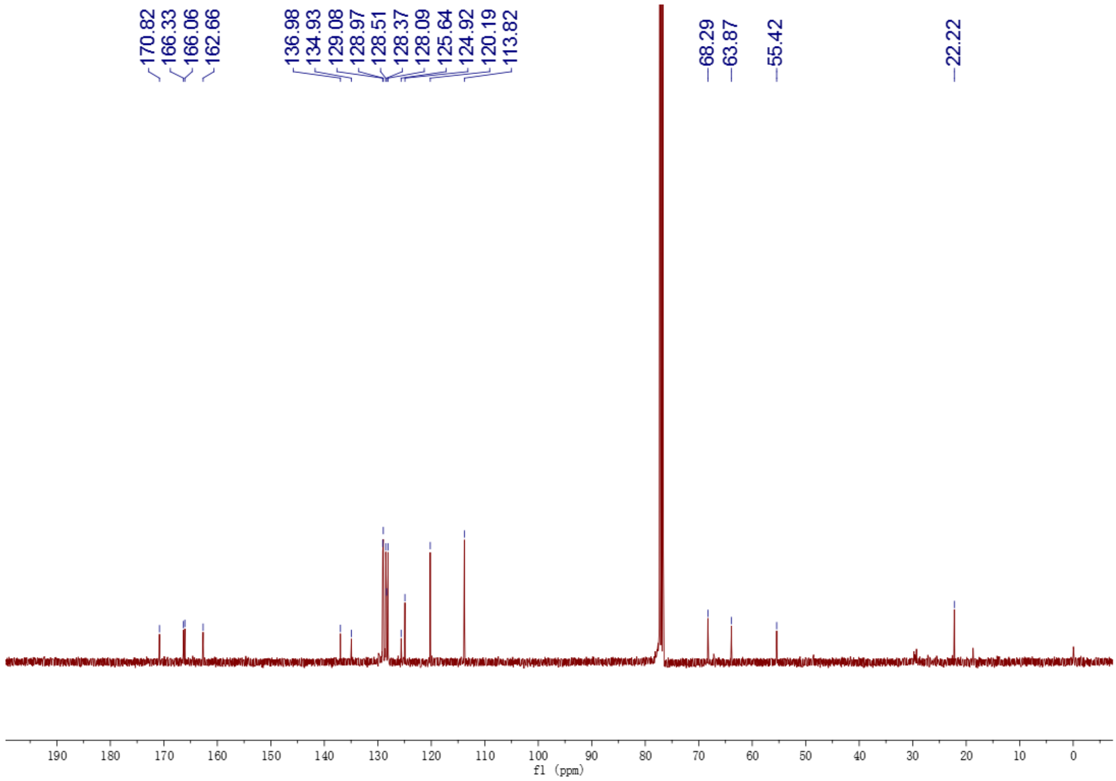
170.82
166.33
166.06
162.66
136.98
134.93
129.08
128.97
128.51
128.37
128.09
125.64
124.92
120.19
113.82
68.29
63.87
55.42
22.22
f1 (ppm)

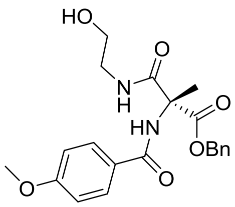
HO
O
N
H
O
HN
OBn
O
O

2e

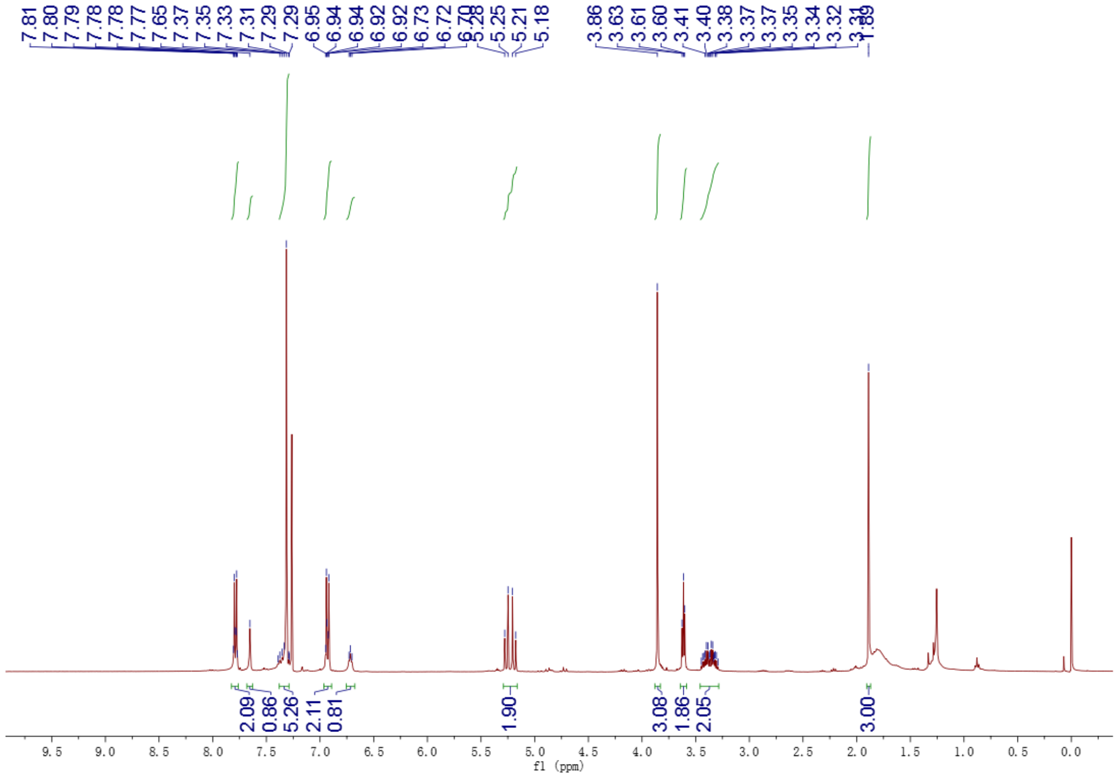
7.81 7.80 7.79 7.78 7.78 7.77 7.65 7.37 7.35 7.33 7.31 7.29 7.29 6.95 6.94 6.94 6.92 6.92 6.73 6.72 6.70 5.28 5.25 5.21 5.18 3.86 3.63 3.61 3.60 3.41 3.40 3.38 3.37 3.37 3.35 3.34 3.32 3.31 1.89
2.09 0.86 5.26 2.11 0.81 1.90 3.08 1.86 2.05 3.00
9.5 9.0 8.5 8.0 7.5 7.0 6.5 6.0 5.5 5.0 4.5 4.0 3.5 3.0 2.5 2.0 1.5 1.0 0.5 0.0
f1 (ppm)

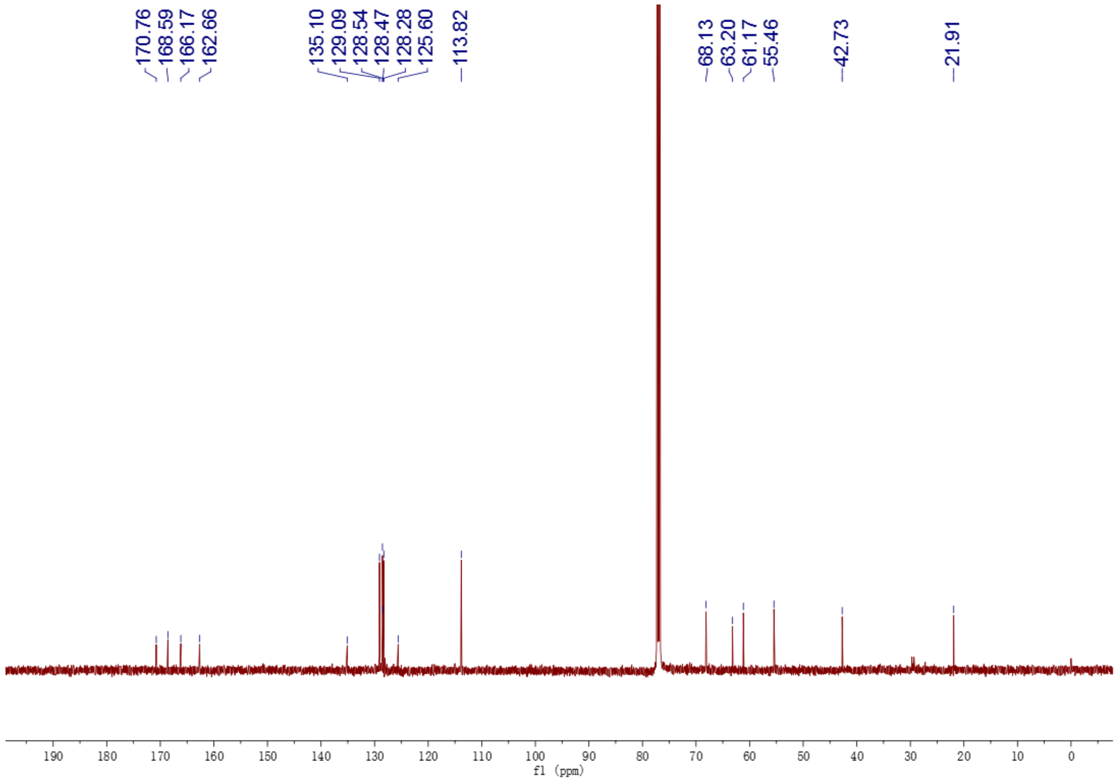
170.76 168.59 166.17 162.66 135.10 129.09 128.54 128.47 128.28 125.60 113.82 68.13 63.20 61.17 55.46 42.73 21.91
190 180 170 160 150 140 130 120 110 100 90 80 70 60 50 40 30 20 10 0
f1 (ppm)

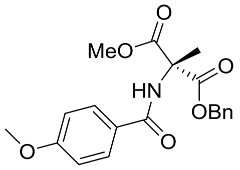
MeO
O
HN
O
OBn
O
O

2f

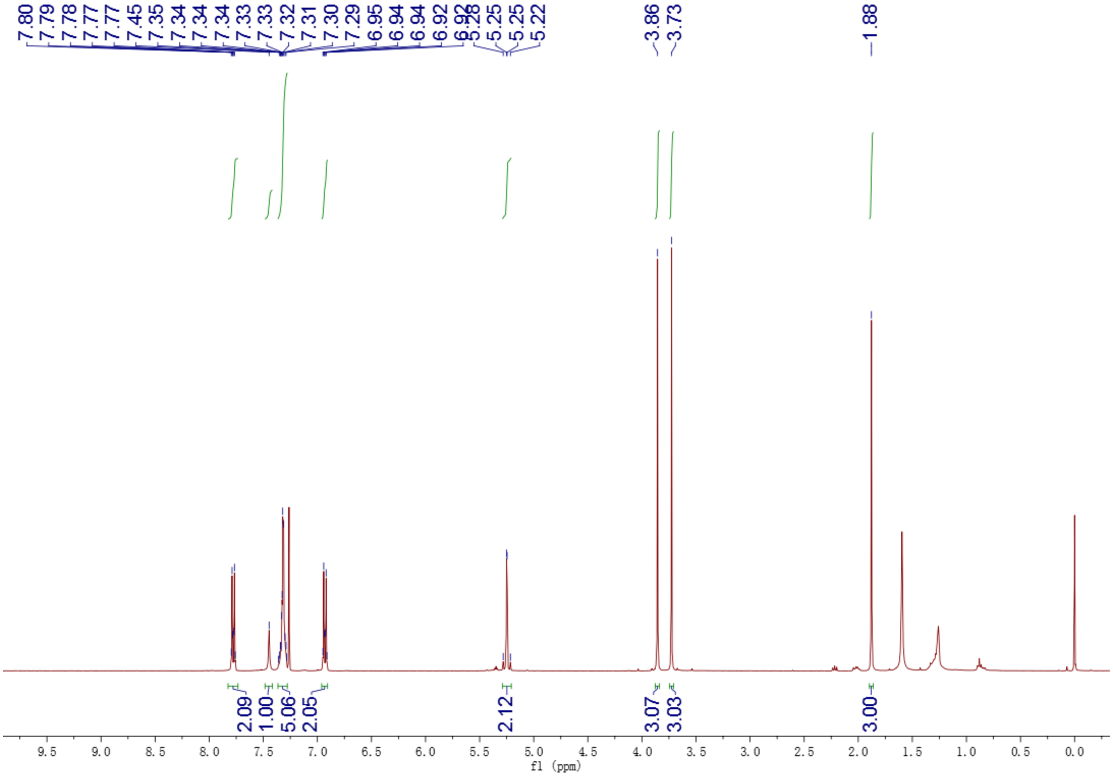
7.80
7.79
7.78
7.77
7.77
7.45
7.35
7.34
7.34
7.33
7.33
7.32
7.31
7.30
7.29
6.95
6.94
6.94
6.92
6.92
5.28
5.25
5.25
5.22
3.86
3.73
1.88
2.09
1.00
5.06
2.05
2.12
3.07
3.03
3.00
9.5 9.0 8.5 8.0 7.5 7.0 6.5 6.0 5.5 5.0 4.5 4.0 3.5 3.0 2.5 2.0 1.5 1.0 0.5 0.0
f1 (ppm)

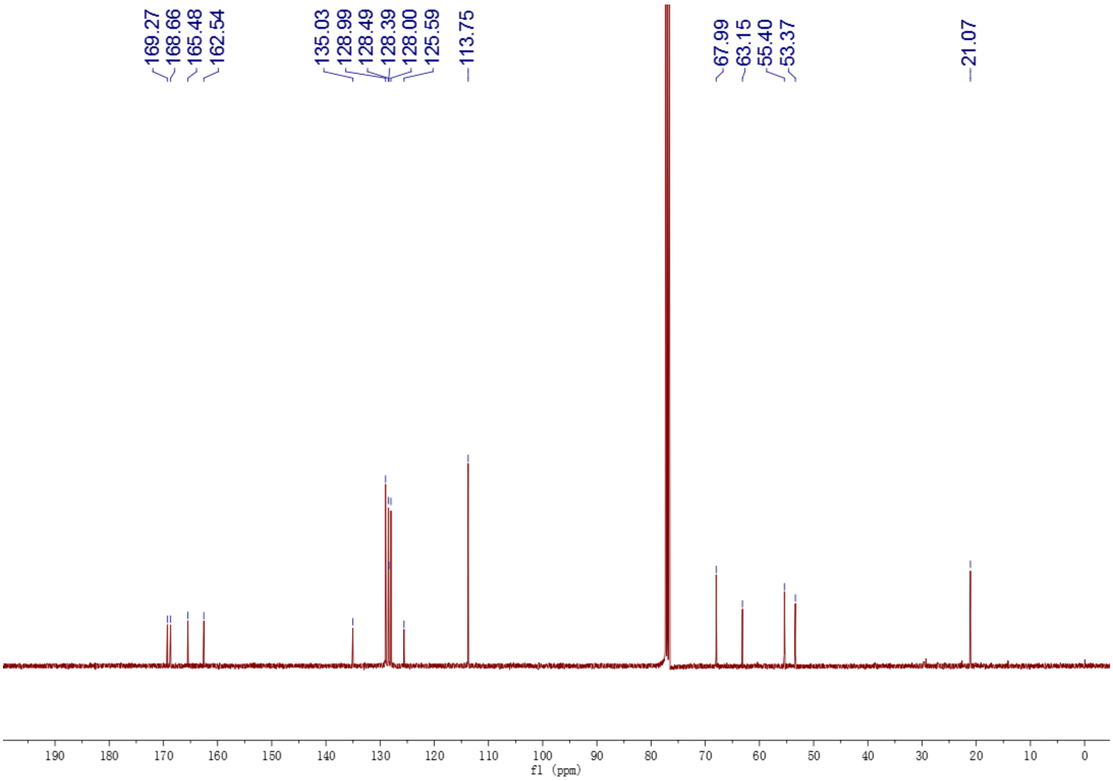
169.27
168.66
165.48
162.54
135.03
128.99
128.49
128.39
128.00
125.59
113.75
67.99
63.15
55.40
53.37
21.07
190 180 170 160 150 140 130 120 110 100 90 80 70 60 50 40 30 20 10 0
f1 (ppm)

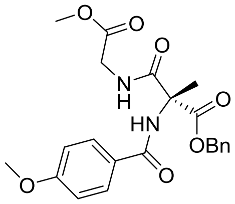
O
N
H
O
HN
O
OBn
O
O

2g

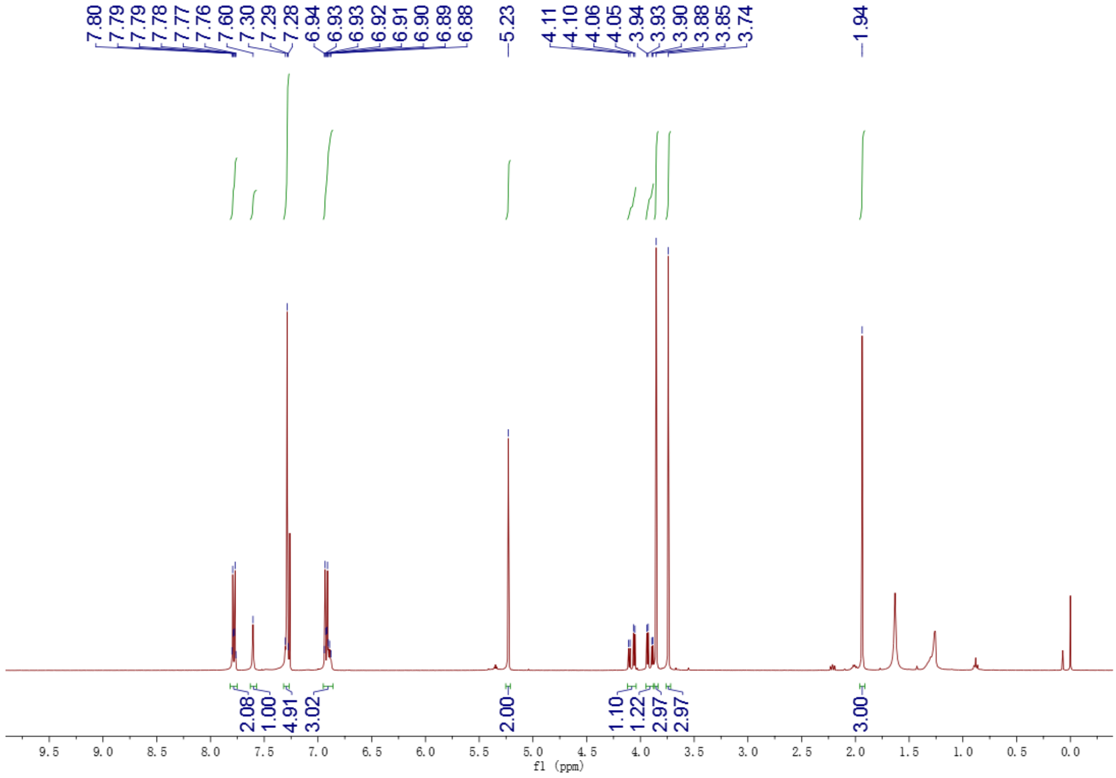
7.80
7.79
7.79
7.78
7.77
7.76
7.60
7.30
7.29
7.28
6.94
6.93
6.92
6.91
6.90
6.89
6.88
5.23
4.11
4.10
4.06
4.05
3.94
3.93
3.90
3.88
3.85
3.74
1.94
2.08
1.00
4.91
3.02
2.00
1.10
1.22
2.97
2.97
3.00
f1 (ppm)

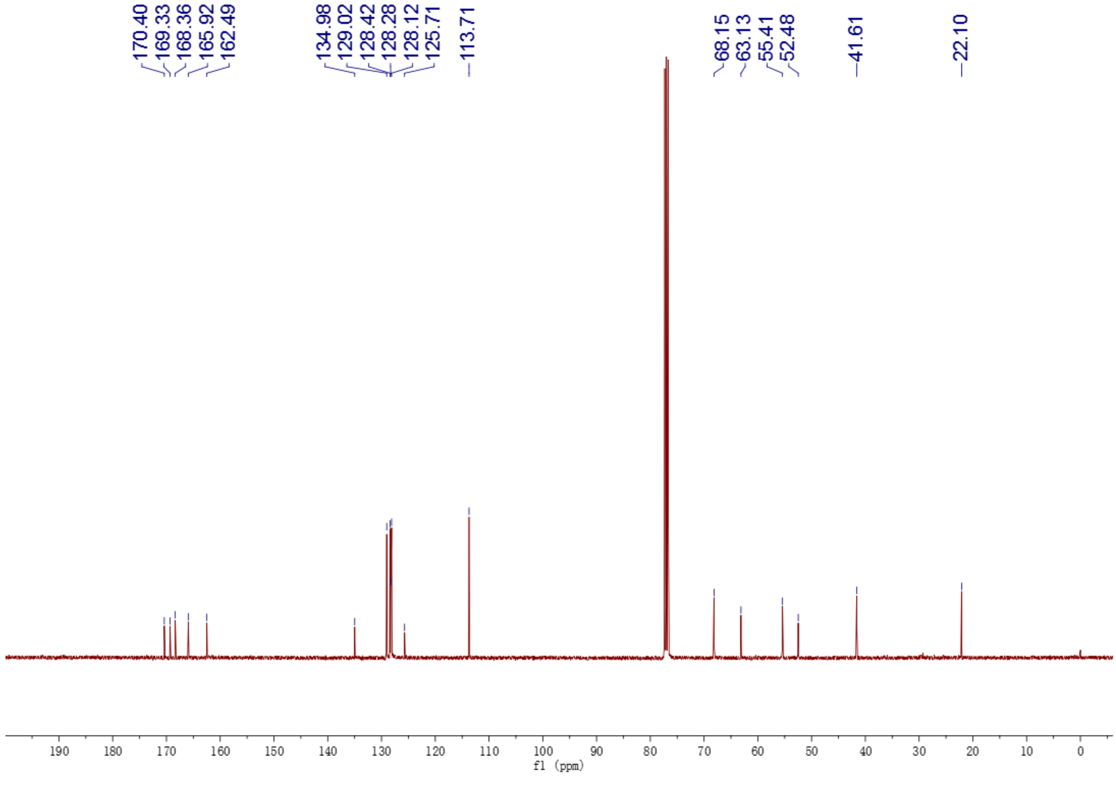
170.40
169.33
168.36
165.92
162.49
134.98
129.02
128.42
128.28
128.12
125.71
113.71
68.15
63.13
55.41
52.48
41.61
22.10
f1 (ppm)

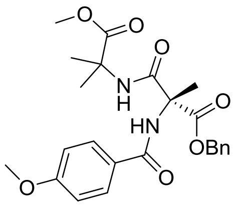
O
O
O
N
H
HN
O
OBn
O
O

2h

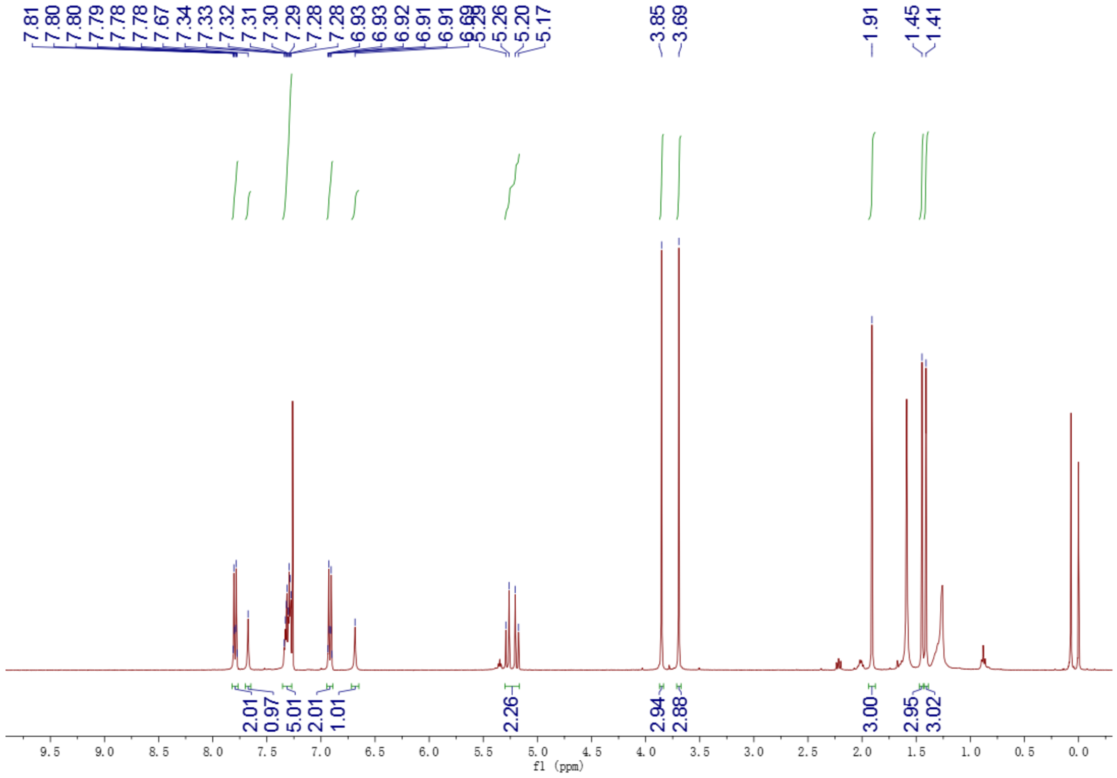
7.81
7.80
7.80
7.79
7.78
7.78
7.67
7.34
7.33
7.32
7.31
7.30
7.29
7.28
7.28
6.93
6.93
6.92
6.91
6.91
6.69
5.29
5.26
5.20
5.17
3.85
3.69
1.91
1.45
1.41
2.01
0.97
5.01
2.01
1.01
2.26
2.94
2.88
3.00
2.95
3.02
9.5 9.0 8.5 8.0 7.5 7.0 6.5 6.0 5.5 5.0 4.5 4.0 3.5 3.0 2.5 2.0 1.5 1.0 0.5 0.0
f1 (ppm)

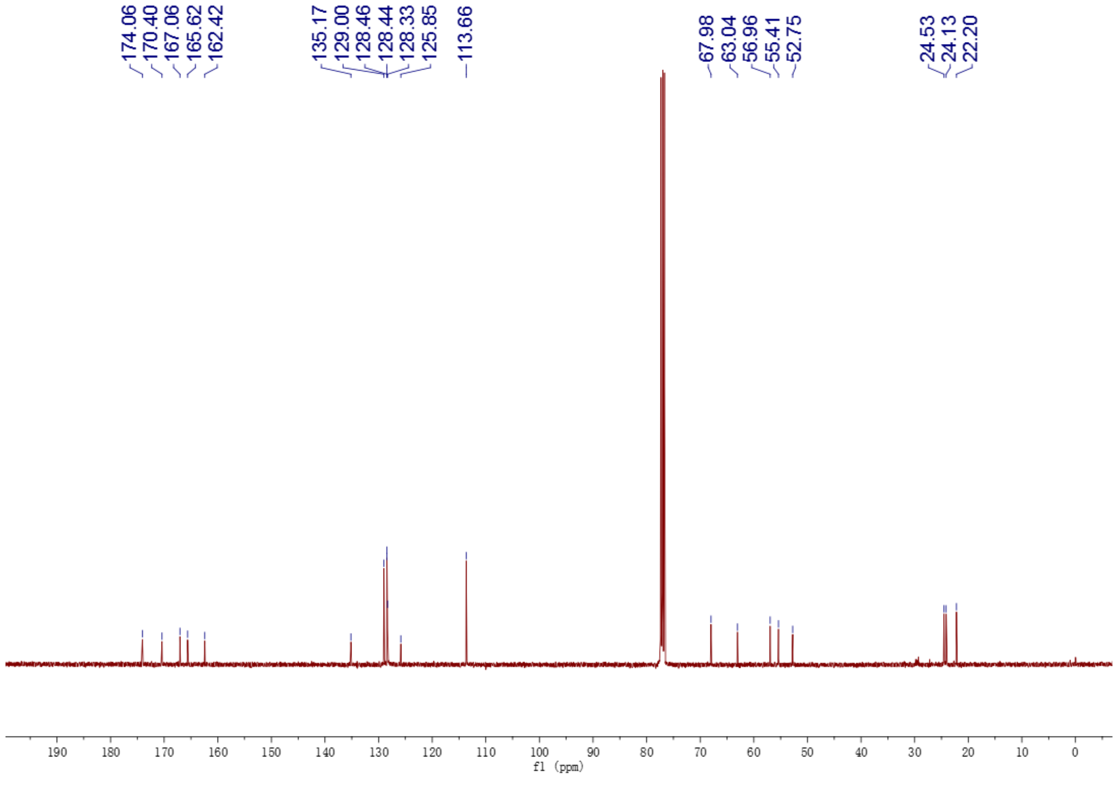
174.06
170.40
167.06
165.62
162.42
135.17
129.00
128.46
128.44
128.33
125.85
113.66
67.98
63.04
56.96
55.41
52.75
24.53
24.13
22.20
190 180 170 160 150 140 130 120 110 100 90 80 70 60 50 40 30 20 10 0
f1 (ppm)

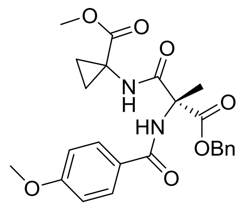
O
O
N
H
O
HN
O
OBn
O
O

2i

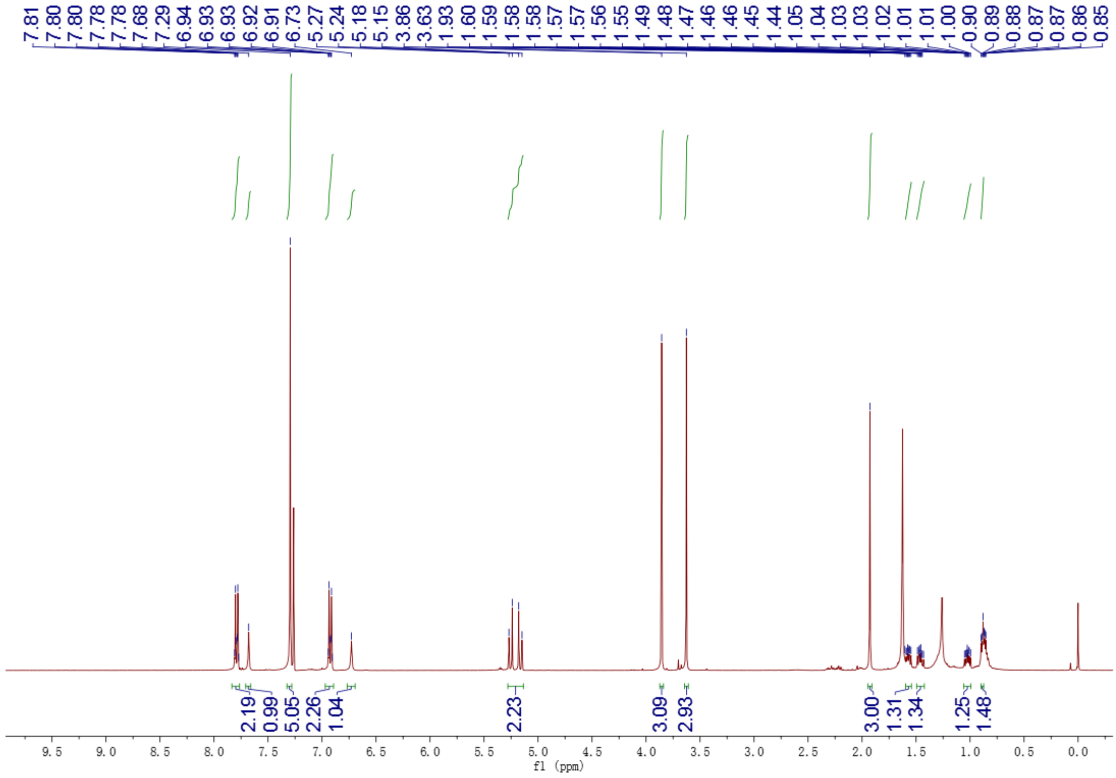
7.81
7.80
7.80
7.78
7.78
7.68
7.29
6.94
6.93
6.93
6.92
6.91
6.73
5.27
5.24
5.18
5.15
3.86
3.63
1.93
1.60
1.59
1.58
1.58
1.57
1.56
1.55
1.49
1.48
1.47
1.46
1.46
1.45
1.44
1.05
1.04
1.03
1.03
1.02
1.01
1.01
1.00
0.90
0.89
0.88
0.87
0.87
0.86
0.85
2.19
0.99
5.05
2.26
1.04
2.23
3.09
2.93
3.00
1.31
1.34
1.25
1.48
9.5
9.0
8.5
8.0
7.5
7.0
6.5
6.0
5.5
5.0
4.5
4.0
3.5
3.0
2.5
2.0
1.5
1.0
0.5
0.0
f1 (ppm)

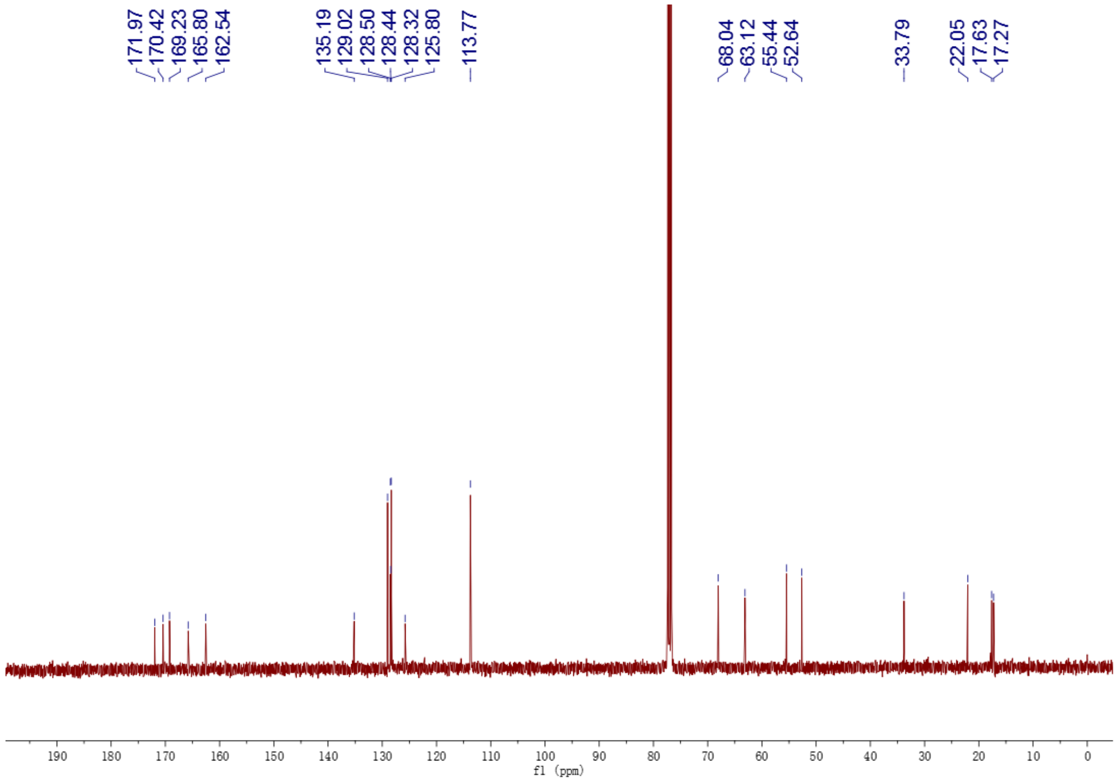
171.97
170.42
169.23
165.80
162.54
135.19
129.02
128.50
128.44
128.32
125.80
113.77
68.04
63.12
55.44
52.64
33.79
22.05
17.63
17.27
190
180
170
160
150
140
130
120
110
100
90
80
70
60
50
40
30
20
10
0
f1 (ppm)

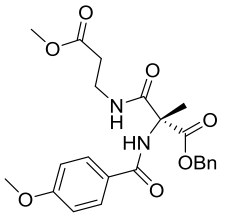
O
O
O
N
H
HN
O
OBn
O
O

2j

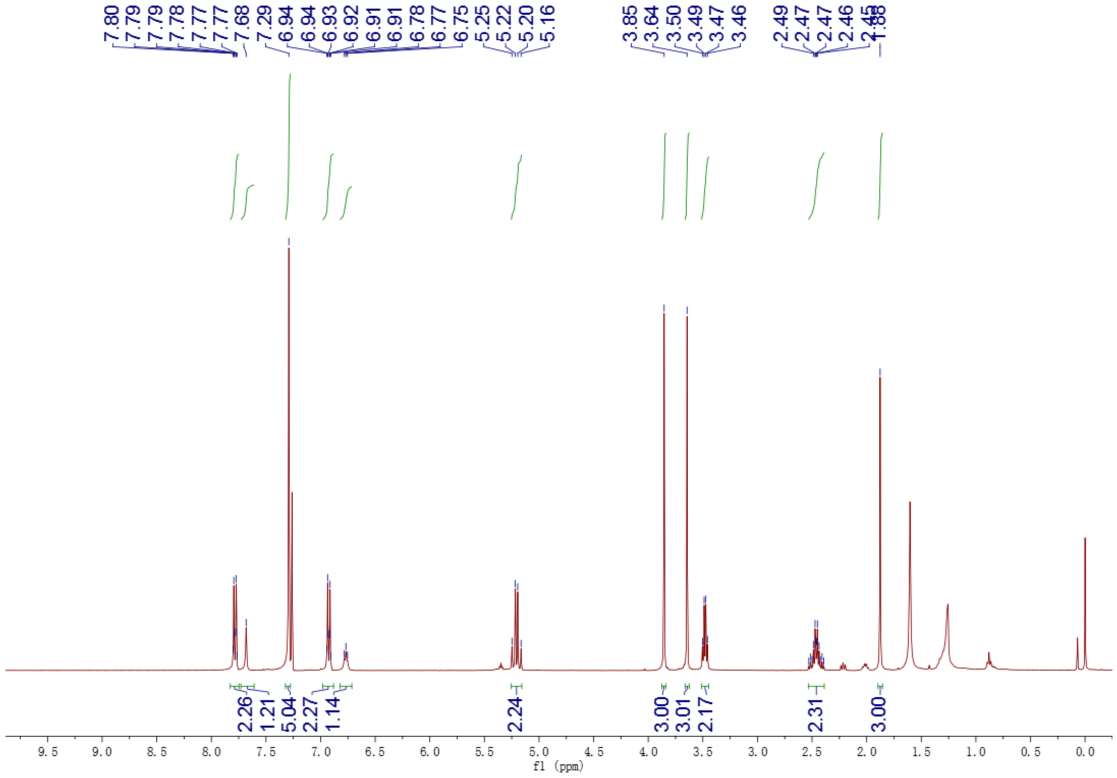
7.80 7.79 7.79 7.78 7.77 7.77 7.68 7.29 6.94 6.94 6.93 6.92 6.91 6.91 6.78 6.77 6.75 5.25 5.22 5.20 5.16
3.85 3.64 3.50 3.49 3.47 3.46
2.49 2.47 2.47 2.46 2.45 1.88
2.26 1.21 5.04 2.27 1.14 2.24 3.00 3.01 2.17 2.31 3.00
f1 (ppm)

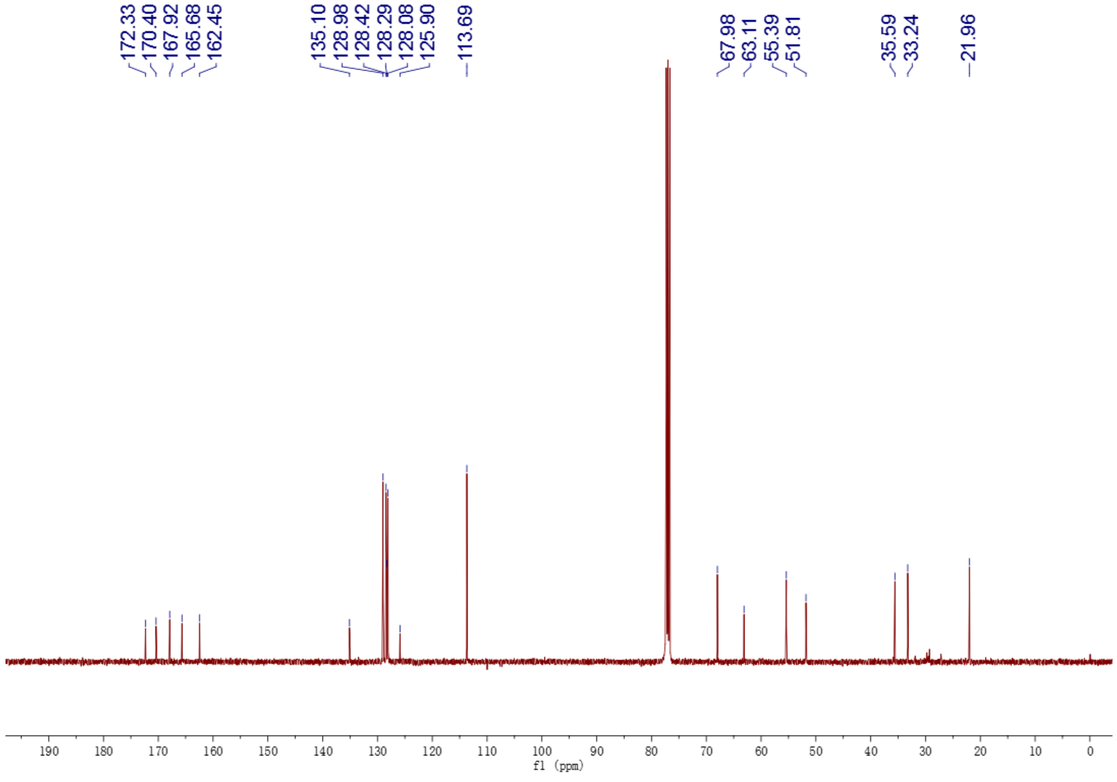
172.33 170.40 167.92 165.68 162.45
135.10 128.98 128.42 128.29 128.08 125.90 113.69
67.98 63.11 55.39 51.81
35.59 33.24 21.96
f1 (ppm)

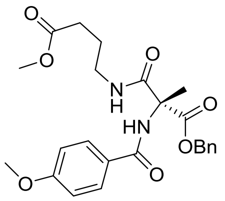
O
O
O
N
H
HN
O
OBn
O
O

2k

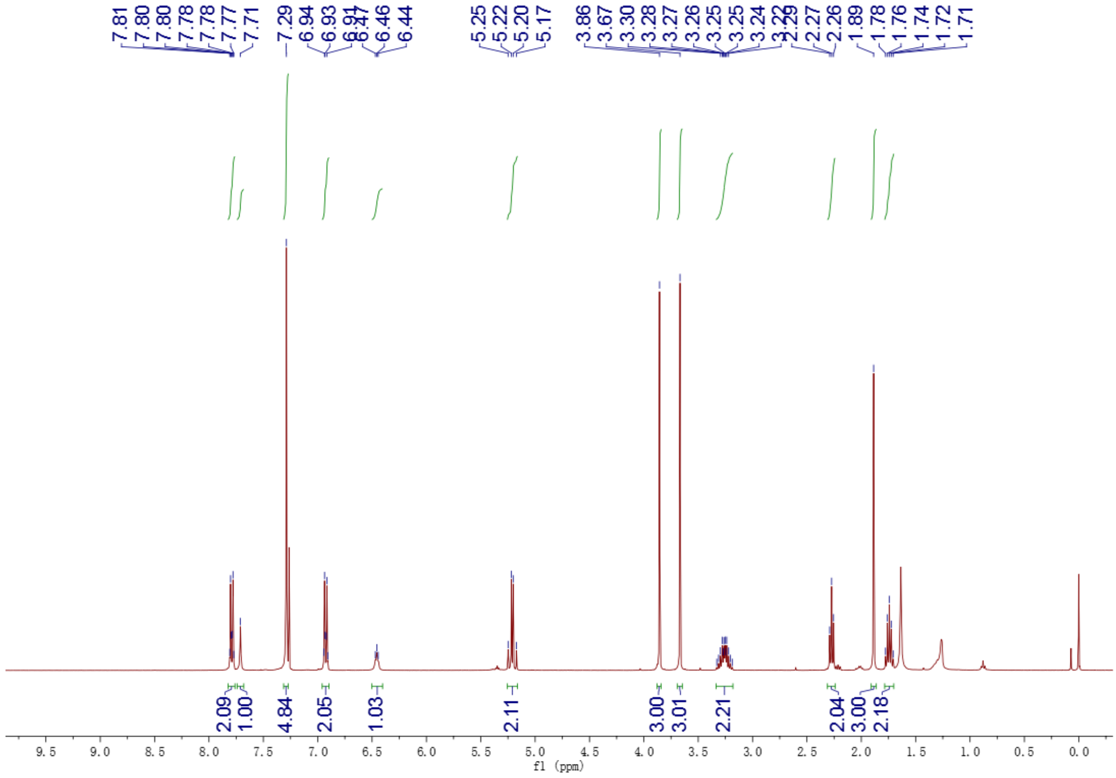
7.81
7.80
7.80
7.78
7.78
7.77
7.71
7.29
6.94
6.93
6.46
6.44
5.25
5.22
5.20
5.17
3.86
3.67
3.30
3.28
3.27
3.26
3.25
3.25
3.24
2.27
2.26
1.89
1.78
1.76
1.74
1.72
1.71
2.09
1.00
4.84
2.05
1.03
2.11
3.00
3.01
2.21
2.04
3.00
2.18
f1 (ppm)

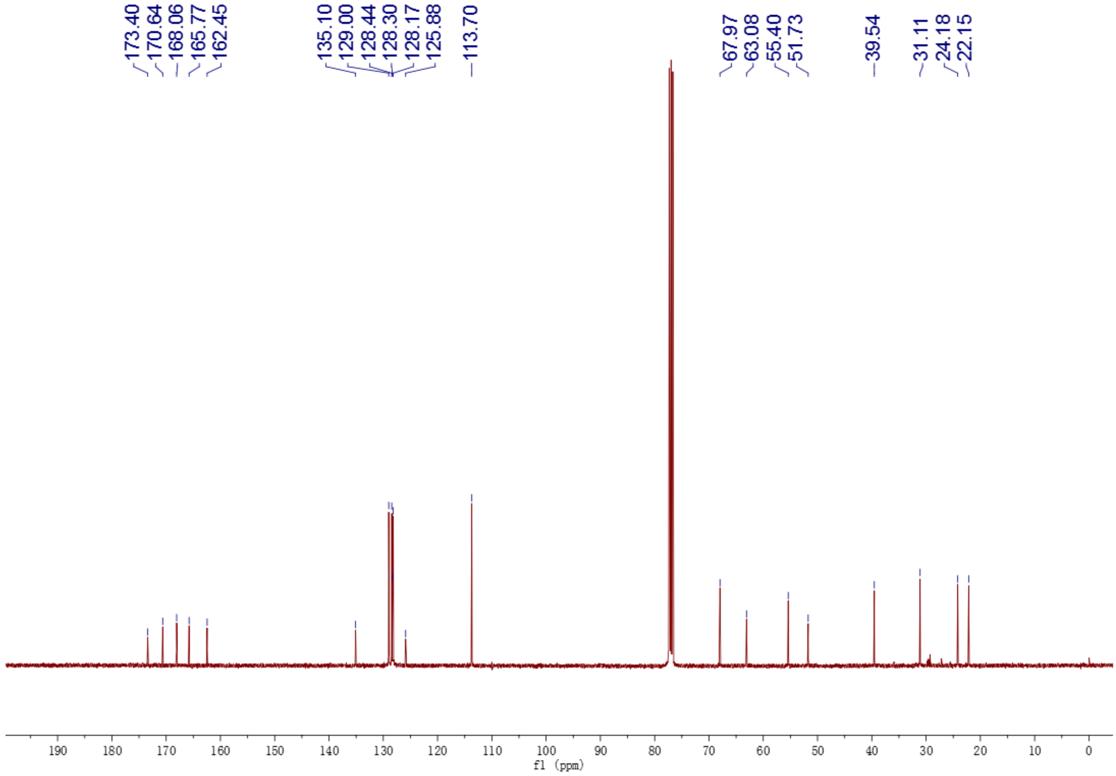
173.40
170.64
168.06
165.77
162.45
135.10
129.00
128.44
128.30
128.17
125.88
113.70
67.97
63.08
55.40
51.73
39.54
31.11
24.18
22.15
f1 (ppm)

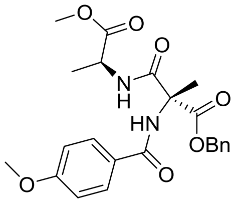
O
O
O
N
H
HN
O
OBn
O
O

2l

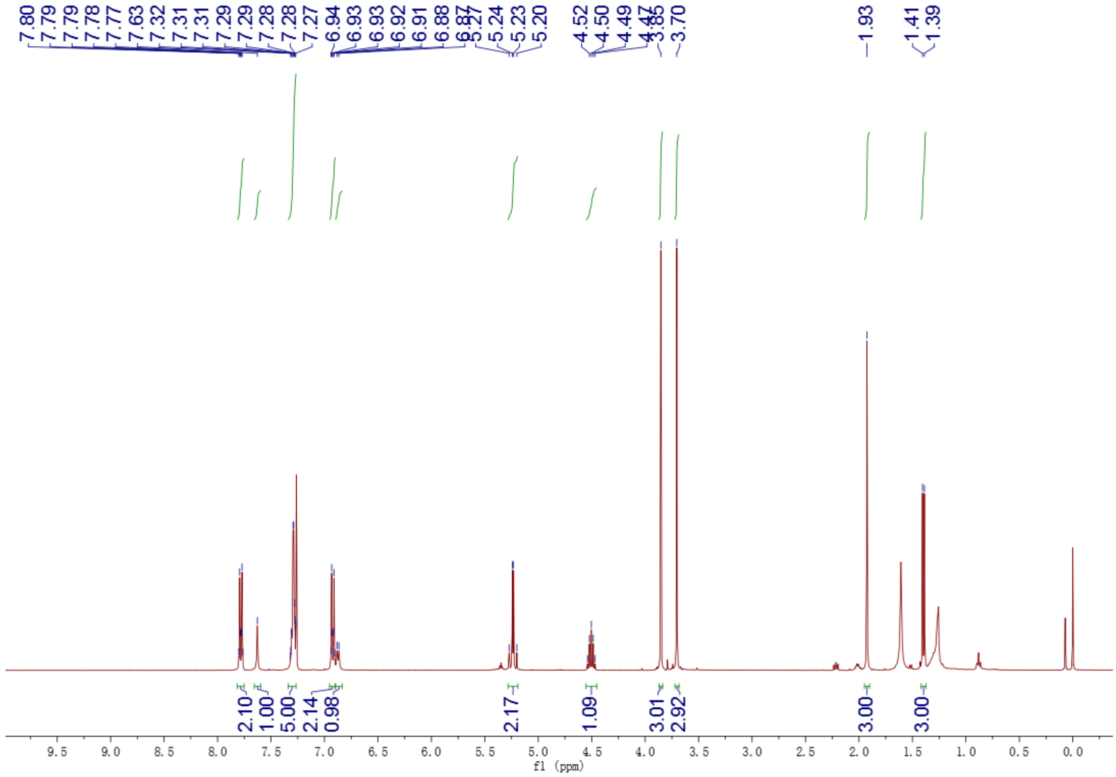
7.80
7.79
7.79
7.78
7.77
7.63
7.32
7.31
7.31
7.29
7.29
7.28
7.28
7.27
6.94
6.93
6.93
6.92
6.91
6.88
6.87
5.27
5.24
5.23
5.20
4.52
4.50
4.49
4.47
3.85
3.70
1.93
1.41
1.39
2.10
1.00
5.00
2.14
0.98
2.17
1.09
3.01
2.92
3.00
3.00
9.5 9.0 8.5 8.0 7.5 7.0 6.5 6.0 5.5 5.0 4.5 4.0 3.5 3.0 2.5 2.0 1.5 1.0 0.5 0.0
f1 (ppm)

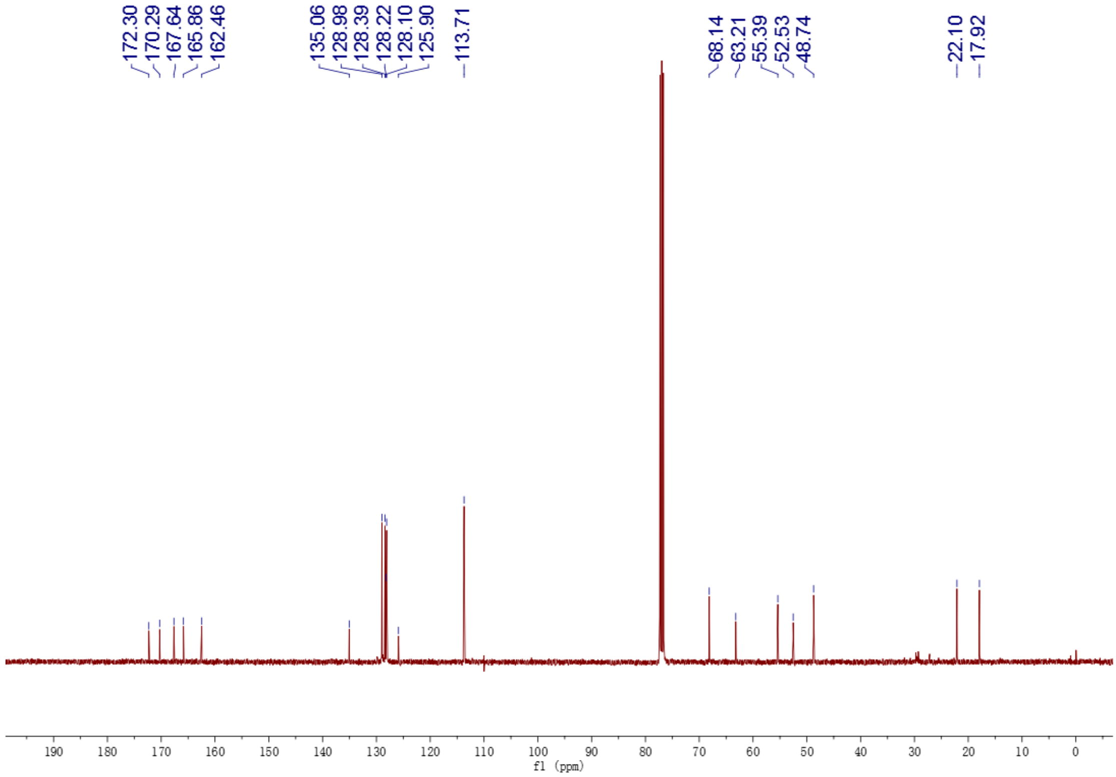
172.30
170.29
167.64
165.86
162.46
135.06
128.98
128.39
128.22
128.10
125.90
113.71
68.14
63.21
55.39
52.53
48.74
22.10
17.92
190 180 170 160 150 140 130 120 110 100 90 80 70 60 50 40 30 20 10 0
f1 (ppm)

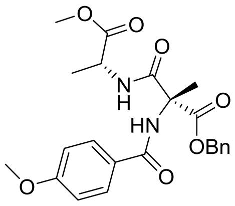
O
O
N
H
O
HN
O
OBn
O
O

2m

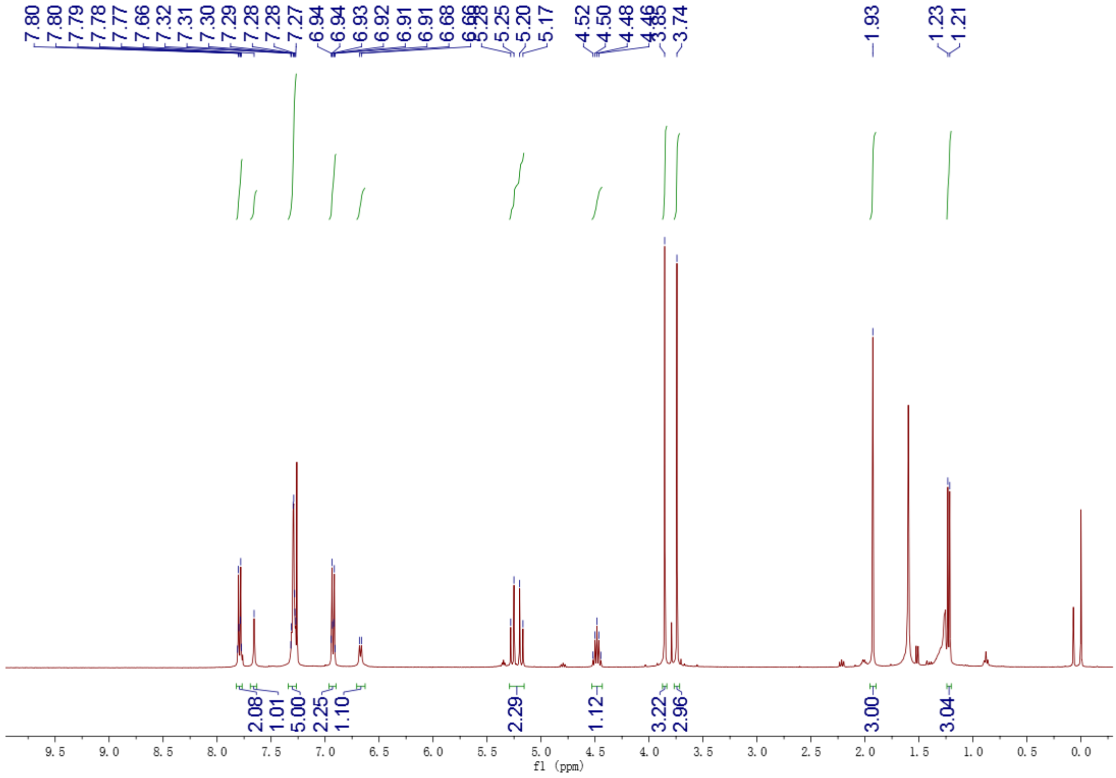
7.80
7.80
7.79
7.78
7.77
7.66
7.32
7.31
7.30
7.29
7.28
7.28
7.27
6.94
6.94
6.93
6.92
6.91
6.91
6.68
5.28
5.25
5.20
5.17
4.52
4.50
4.48
4.46
3.85
3.74
1.93
1.23
1.21
2.08
1.01
5.00
2.25
1.10
2.29
1.12
3.22
2.96
3.00
3.04
f1 (ppm)

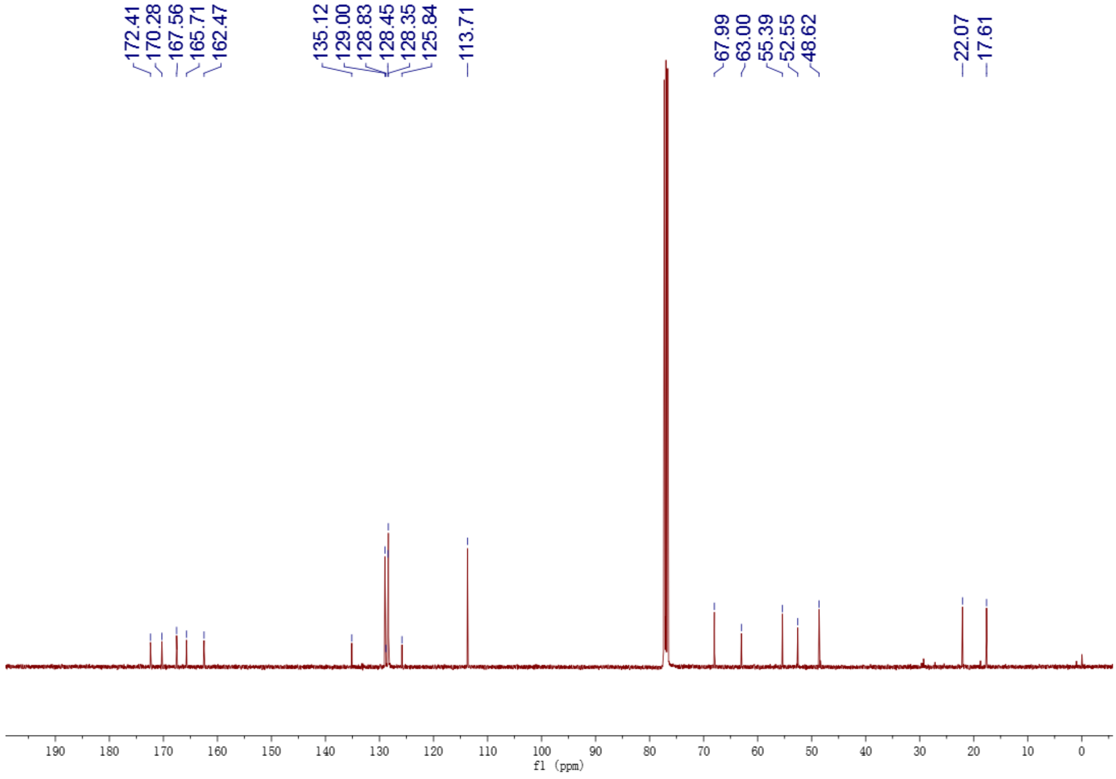
172.41
170.28
167.56
165.71
162.47
135.12
129.00
128.83
128.45
128.35
125.84
113.71
67.99
63.00
55.39
52.55
48.62
22.07
17.61
f1 (ppm)

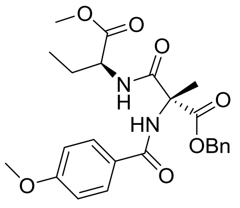
O
O
N
H
O
HN
O
OBn
O
O

2n

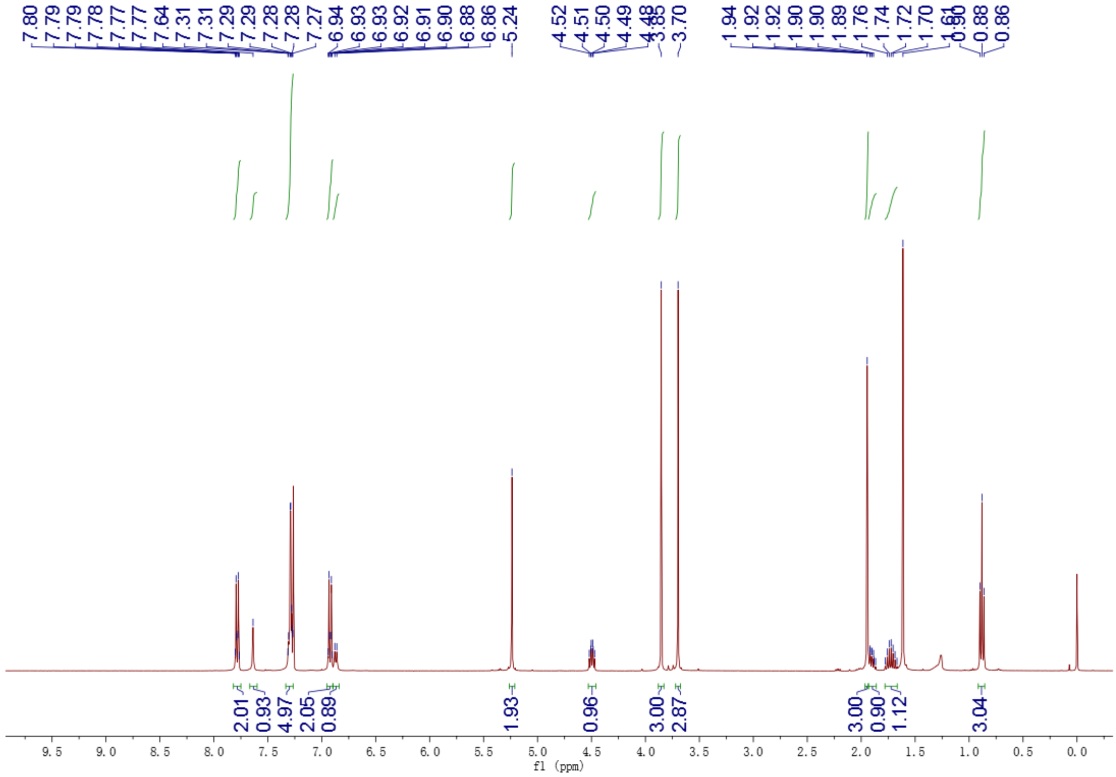
7.80
7.79
7.79
7.78
7.77
7.77
7.64
7.31
7.31
7.29
7.29
7.28
7.28
7.27
6.94
6.93
6.93
6.92
6.91
6.90
6.88
6.86
5.24
4.52
4.51
4.50
4.49
4.48
3.85
3.70
1.94
1.92
1.92
1.90
1.90
1.89
1.76
1.74
1.72
1.70
1.61
0.90
0.88
0.86
2.01
0.93
4.97
2.05
0.89
1.93
0.96
3.00
2.87
3.00
0.90
1.12
3.04
fl (ppm)

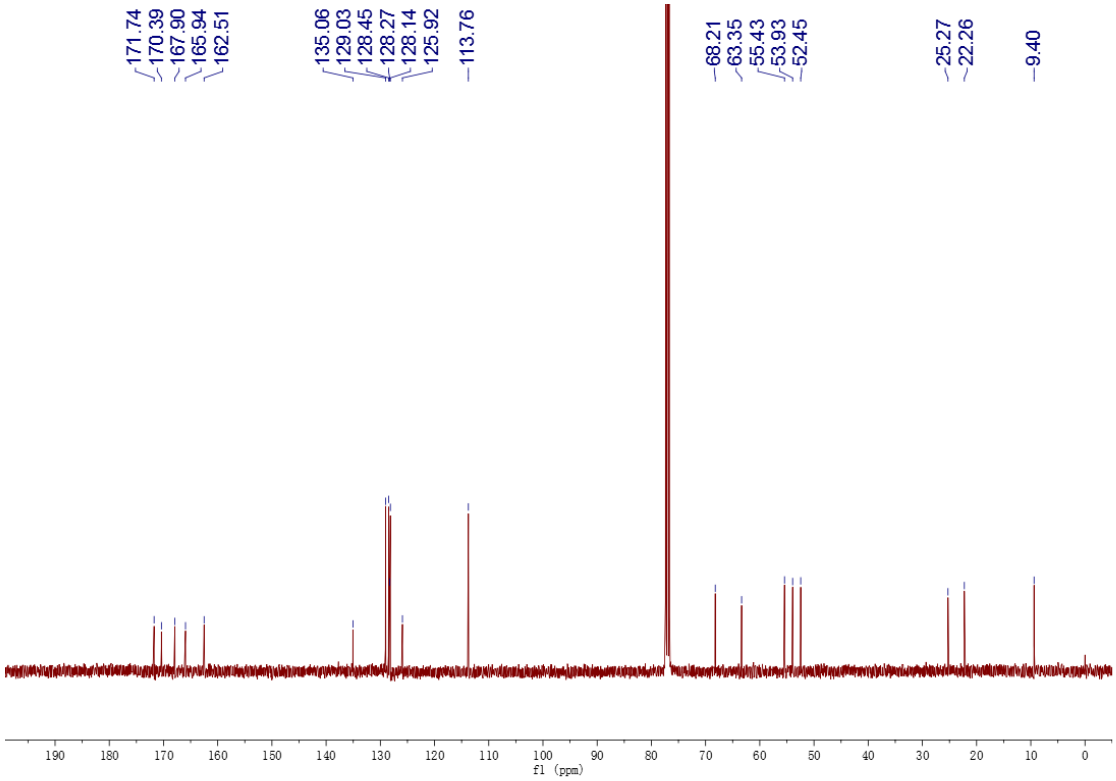
171.74
170.39
167.90
165.94
162.51
135.06
129.03
128.45
128.27
128.14
125.92
113.76
68.21
63.35
55.43
53.93
52.45
25.27
22.26
9.40
fl (ppm)

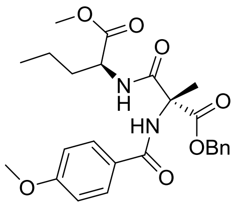
O
O
N
H
O
HN
O
OBn
O
O

2o

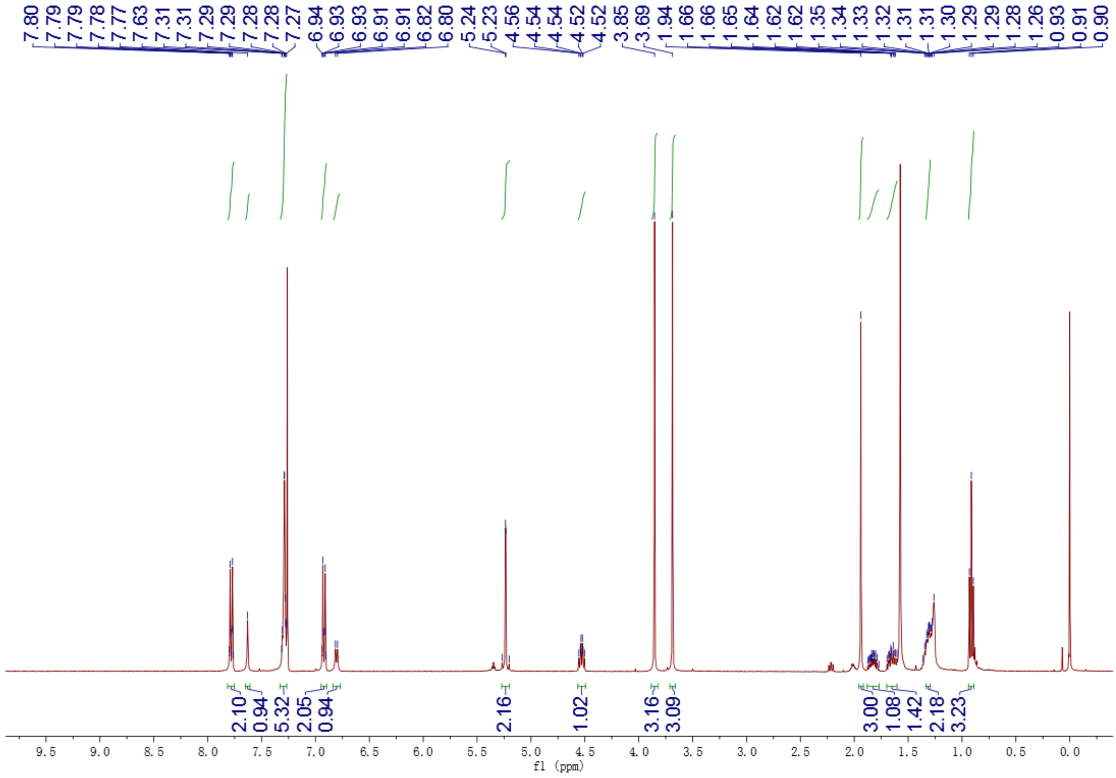
7.80 7.79 7.79 7.78 7.77 7.63 7.31 7.31 7.29 7.29 7.28 7.28 7.27 6.94 6.93 6.93 6.91 6.91 6.82 6.80 5.24 5.23 4.56 4.54 4.54 4.52 4.52 3.85 3.69 1.94 1.66 1.66 1.65 1.64 1.62 1.62 1.35 1.34 1.33 1.32 1.31 1.31 1.30 1.29 1.29 1.28 1.26 0.93 0.91 0.90
2.10 0.94 5.32 2.05 0.94 2.16 1.02 3.16 3.09 3.00 1.08 1.42 2.18 3.23
9.5 9.0 8.5 8.0 7.5 7.0 6.5 6.0 5.5 5.0 4.5 4.0 3.5 3.0 2.5 2.0 1.5 1.0 0.5 0.0
f1 (ppm)

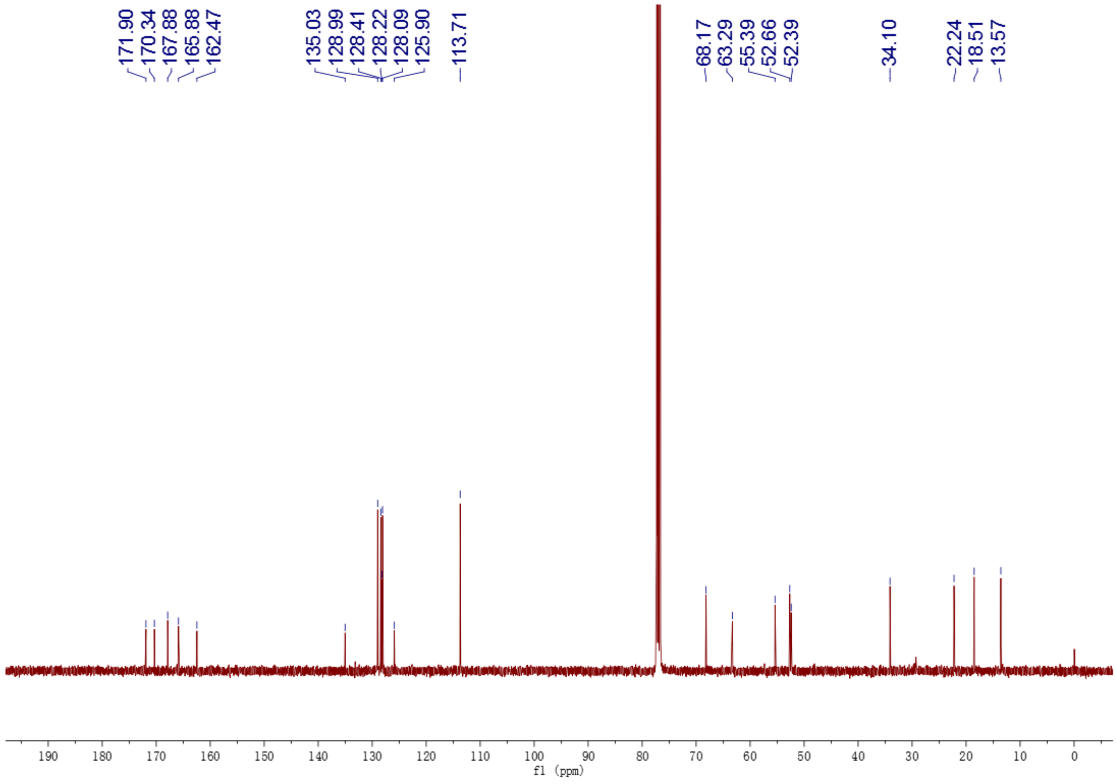
171.90 170.34 167.88 165.88 162.47
135.03 128.99 128.41 128.22 128.09 125.90
113.71
68.17 63.29 55.39 52.66 52.39
34.10
22.24 18.51 13.57
190 180 170 160 150 140 130 120 110 100 90 80 70 60 50 40 30 20 10 0
f1 (ppm)

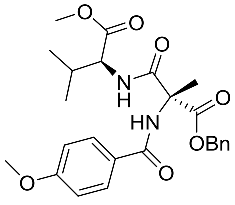
O
O
O
N
H
O
HN
OBn
O
O

2p

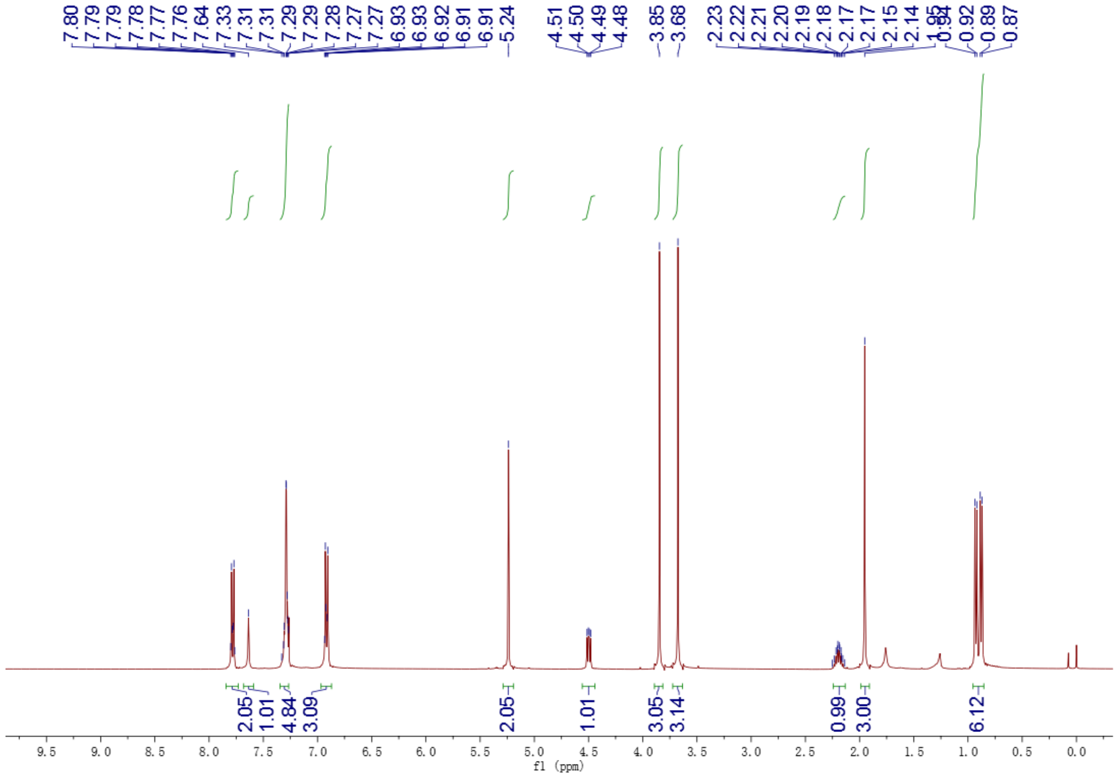
7.80 7.79 7.79 7.78 7.77 7.76 7.64 7.33 7.31 7.31 7.29 7.29 7.28 7.27 7.27 6.93 6.93 6.92 6.91 6.91 5.24 4.51 4.50 4.49 4.48 3.85 3.68 2.23 2.22 2.21 2.20 2.19 2.18 2.17 2.17 2.15 2.14 1.95 1.94 0.92 0.89 0.87
2.05 1.01 4.84 3.09 2.05 1.01 3.05 3.14 0.99 3.00 6.12
f1 (ppm)

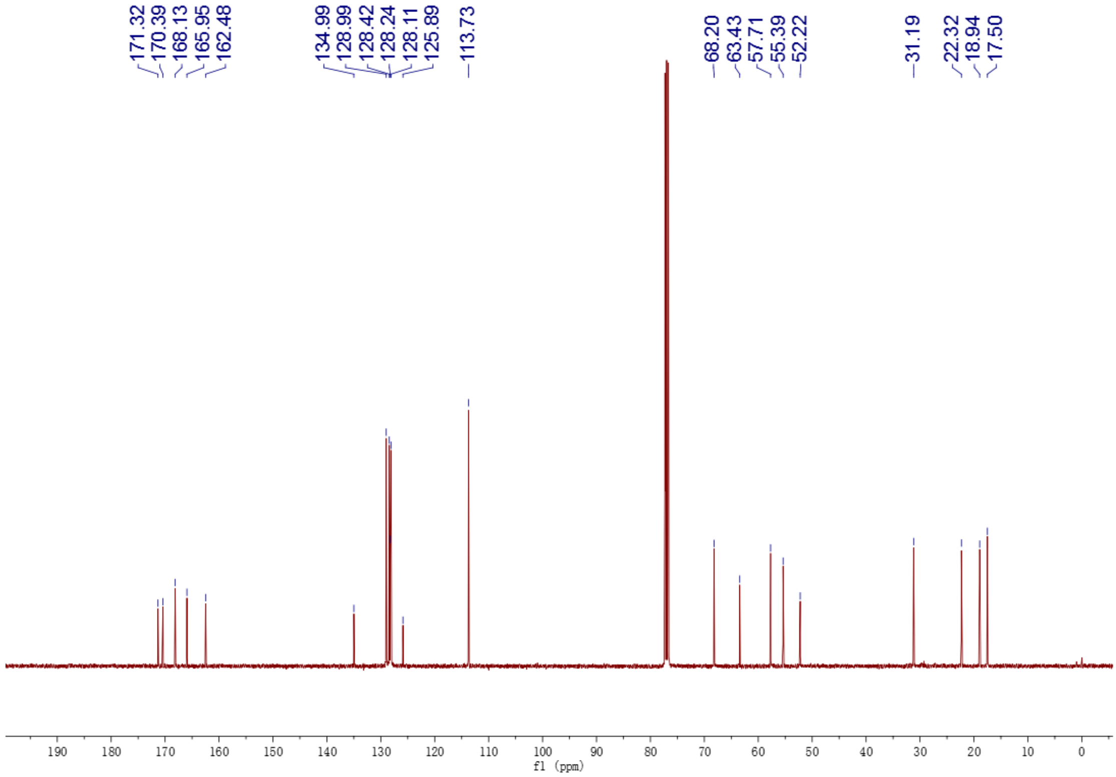
171.32 170.39 168.13 165.95 162.48 134.99 128.99 128.42 128.24 128.11 125.89 113.73 68.20 63.43 57.71 55.39 52.22 31.19 22.32 18.94 17.50
f1 (ppm)

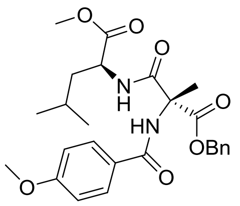

O
O
N
H
O
HN
O
OBn
O
O

2q

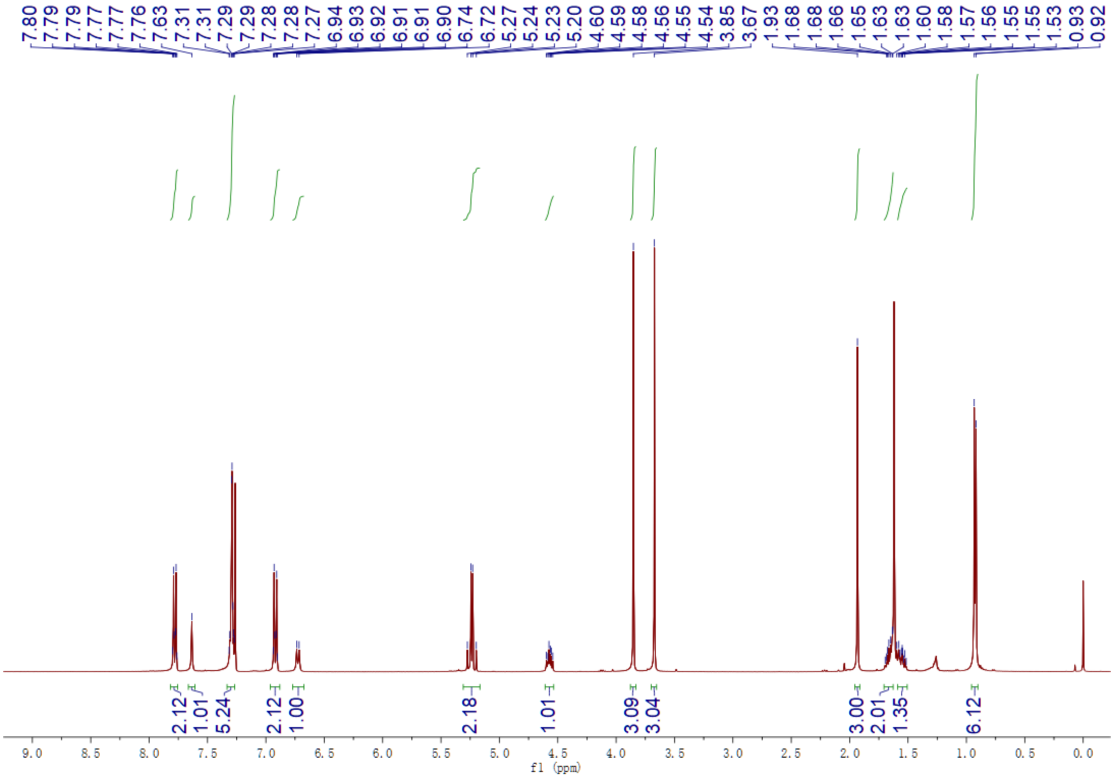

7.80 7.79 7.79 7.77 7.77 7.76 7.63 7.31 7.31 7.29 7.29 7.28 7.28 7.27 6.94 6.93 6.92 6.91 6.91 6.90 6.74 6.72 5.27 5.24 5.23 5.20 4.60 4.59 4.58 4.56 4.55 4.54 3.85 3.67 1.93 1.68 1.68 1.66 1.65 1.63 1.63 1.60 1.58 1.57 1.56 1.55 1.55 1.53 0.93 0.92
2.12 1.01 5.24 2.12 1.00 2.18 1.01 3.09 3.04 3.00 2.01 1.35 6.12
9.0 8.5 8.0 7.5 7.0 6.5 6.0 5.5 5.0 4.5 4.0 3.5 3.0 2.5 2.0 1.5 1.0 0.5 0.0
f1 (ppm)

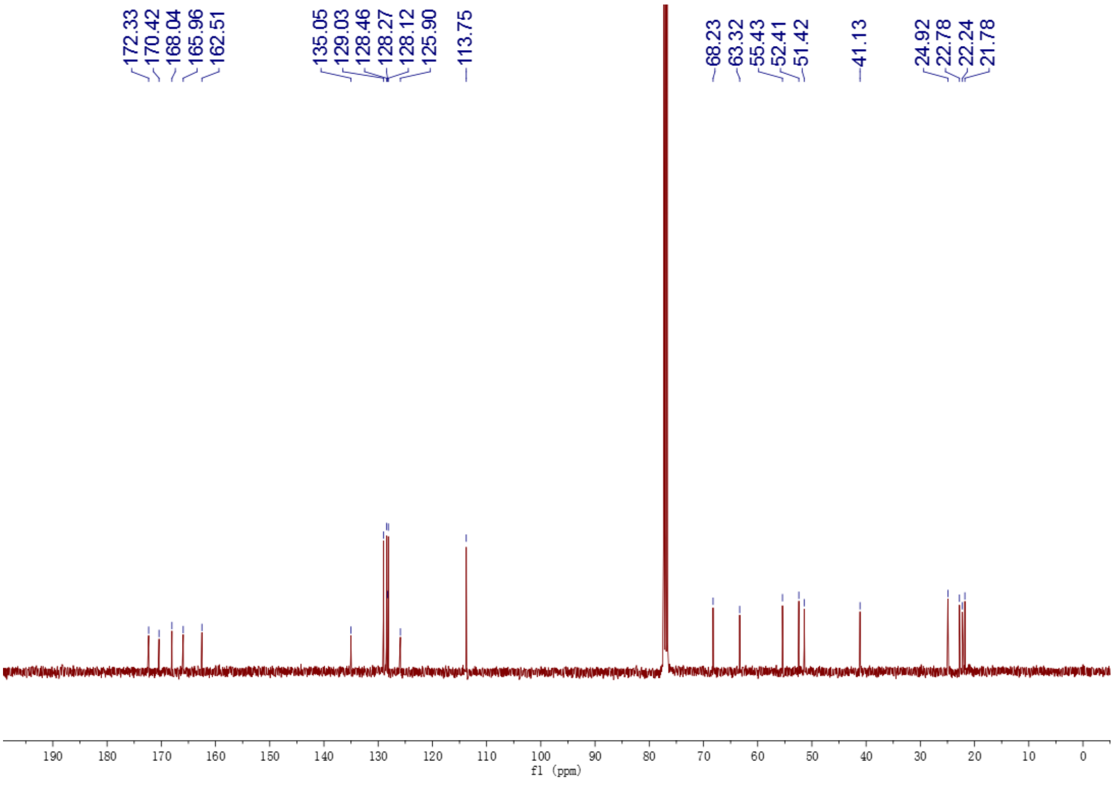

172.33 170.42 168.04 165.96 162.51 135.05 129.03 128.46 128.27 128.12 125.90 113.75 68.23 63.32 55.43 52.41 51.42 41.13 24.92 22.78 22.24 21.78
190 180 170 160 150 140 130 120 110 100 90 80 70 60 50 40 30 20 10 0
f1 (ppm)

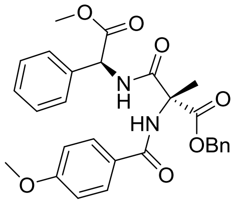
O
O
N
H
O
HN
O
OBn
O
O

2r

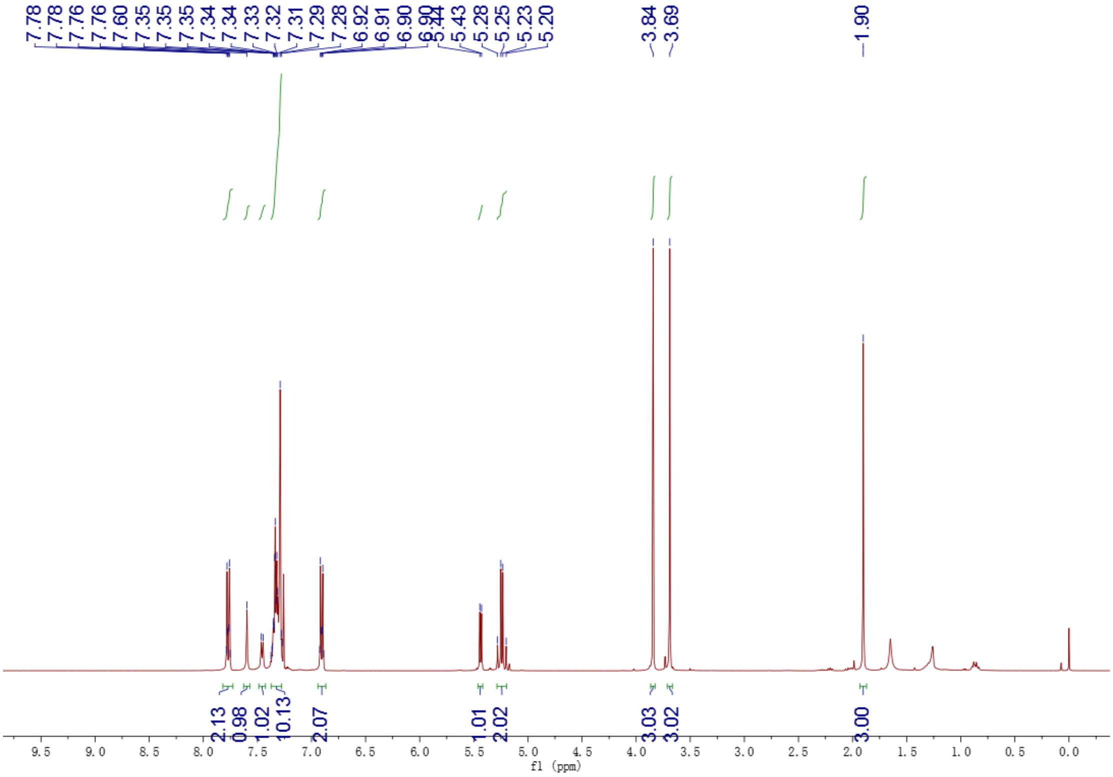
7.78 7.78 7.76 7.76 7.60 7.35 7.35 7.35 7.34 7.34 7.33 7.32 7.31 7.29 7.28 6.92 6.91 6.90 6.90 5.44 5.43 5.28 5.25 5.23 5.20
3.84 3.69
1.90
2.13 0.98 1.02 10.13 2.07 1.01 2.02 3.03 3.02 3.00
9.5 9.0 8.5 8.0 7.5 7.0 6.5 6.0 5.5 5.0 4.5 4.0 3.5 3.0 2.5 2.0 1.5 1.0 0.5 0.0
f1 (ppm)

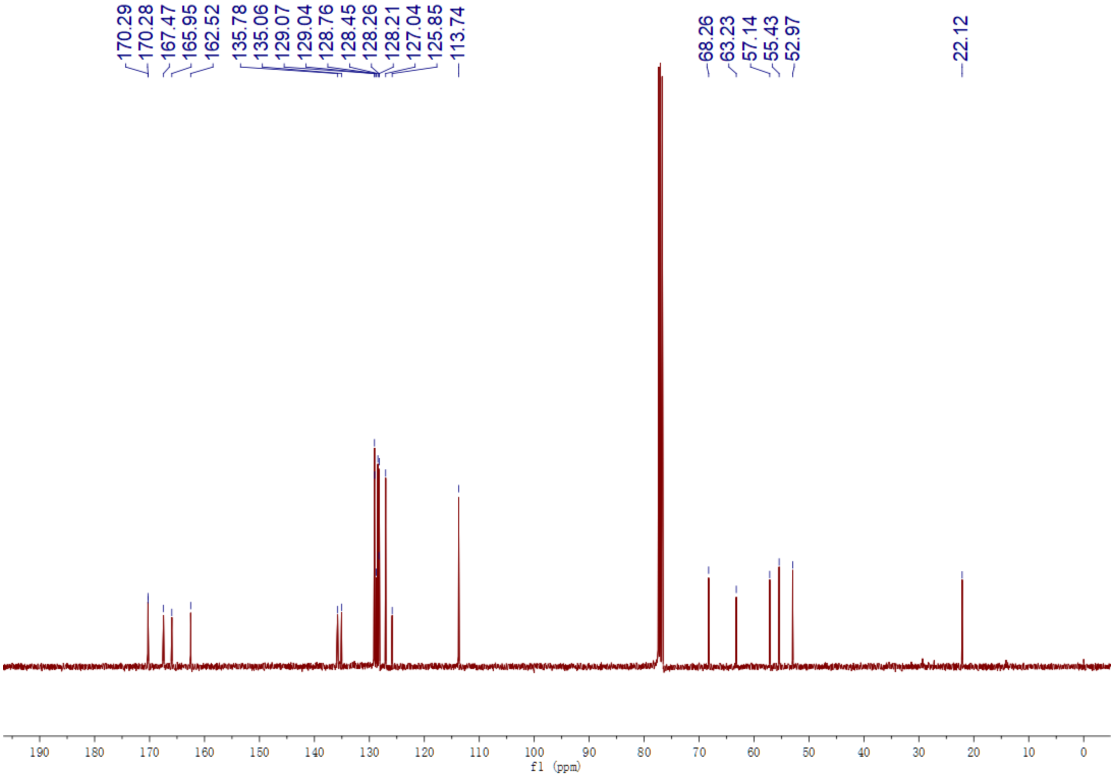
170.29 170.28 167.47 165.95 162.52 135.78 135.06 129.07 129.04 128.76 128.45 128.26 128.21 127.04 125.85 113.74
68.26 63.23 57.14 55.43 52.97
22.12
190 180 170 160 150 140 130 120 110 100 90 80 70 60 50 40 30 20 10 0
f1 (ppm)

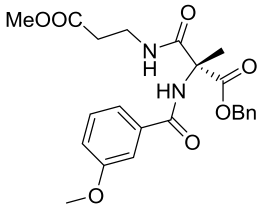
MeOOC
N
H
O
HN
O
OBn
O
O

2s

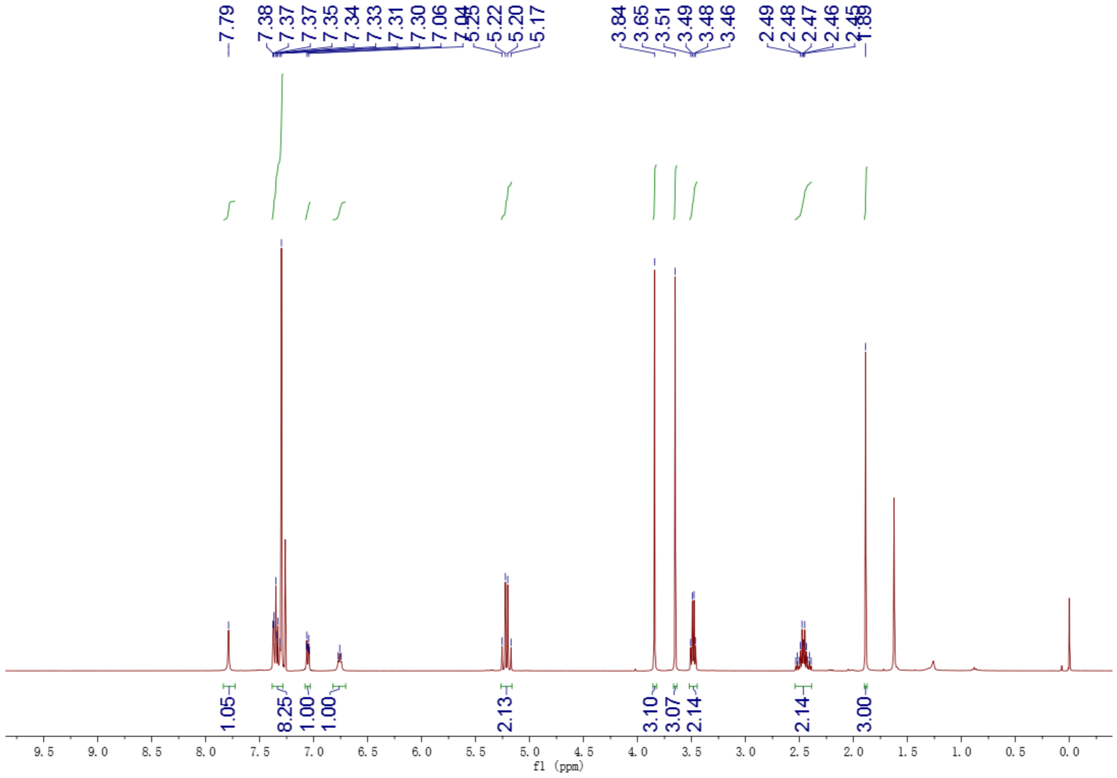
7.79
7.38
7.37
7.37
7.35
7.34
7.33
7.31
7.30
7.06
5.22
5.20
5.17
3.84
3.65
3.51
3.49
3.48
3.46
2.49
2.48
2.47
2.46
1.89
1.05
8.25
1.00
1.00
2.13
3.10
3.07
2.14
2.14
3.00
f1 (ppm)

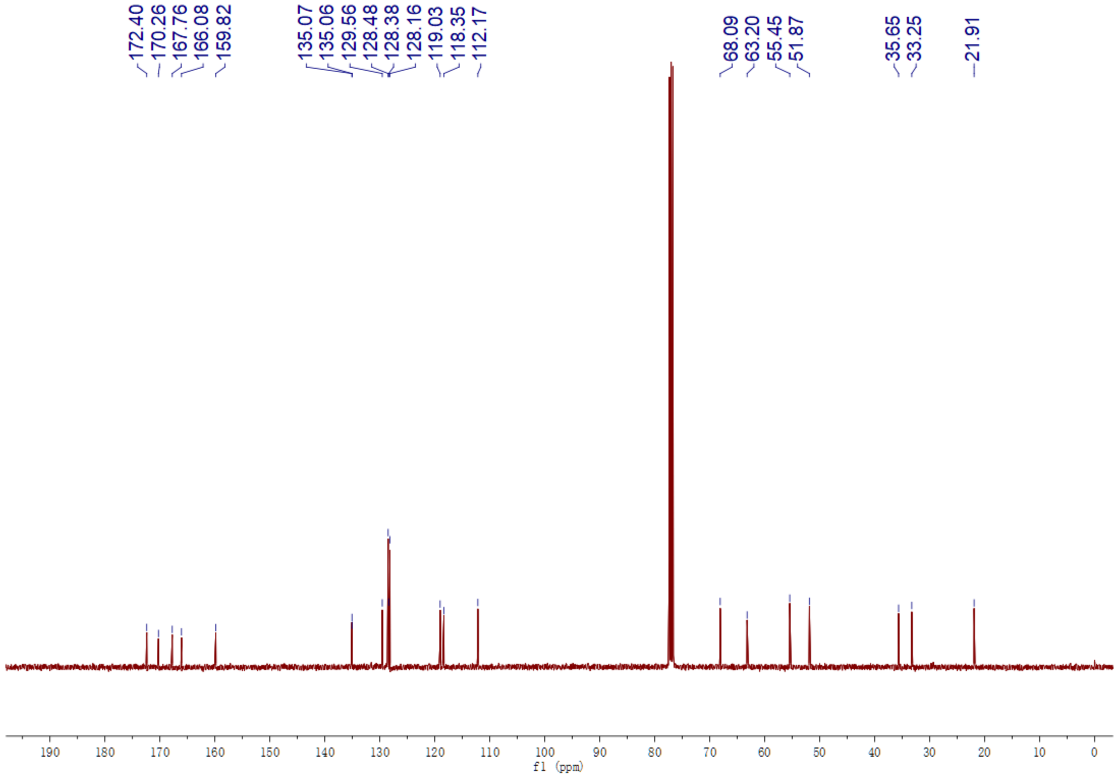
172.40
170.26
167.76
166.08
159.82
135.07
135.06
129.56
128.48
128.38
128.16
119.03
118.35
112.17
68.09
63.20
55.45
51.87
35.65
33.25
21.91
f1 (ppm)

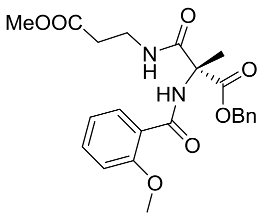

MeOOC
N
H
O
O
HN
OBn
O
O

2t

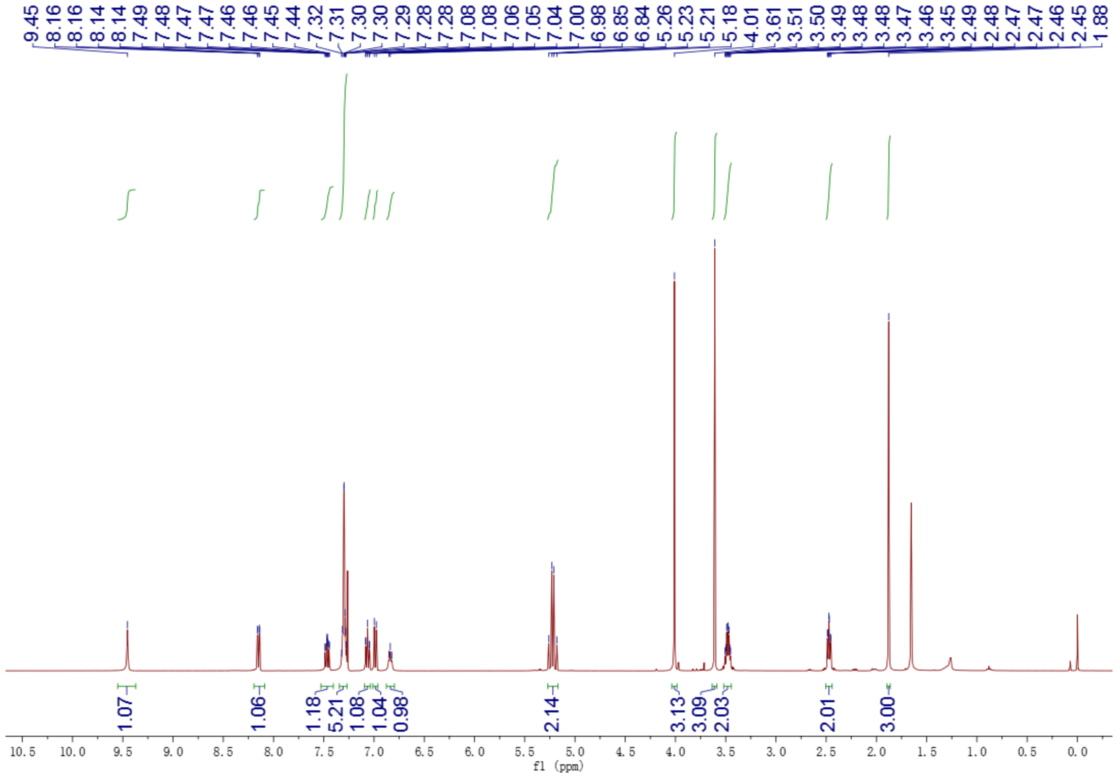

9.45 8.16 8.16 8.14 8.14 7.49 7.48 7.47 7.47 7.46 7.46 7.45 7.44 7.32 7.31 7.30 7.30 7.29 7.28 7.28 7.08 7.08 7.06 7.05 7.04 7.00 6.98 6.85 6.84 5.26 5.23 5.21 5.18 4.01 3.61 3.51 3.50 3.49 3.48 3.48 3.47 3.46 3.45 2.49 2.48 2.47 2.47 2.46 2.45 1.88
1.07 1.06 1.18 5.21 1.08 1.04 0.98 2.14 3.13 3.09 2.03 2.01 3.00
f1 (ppm)

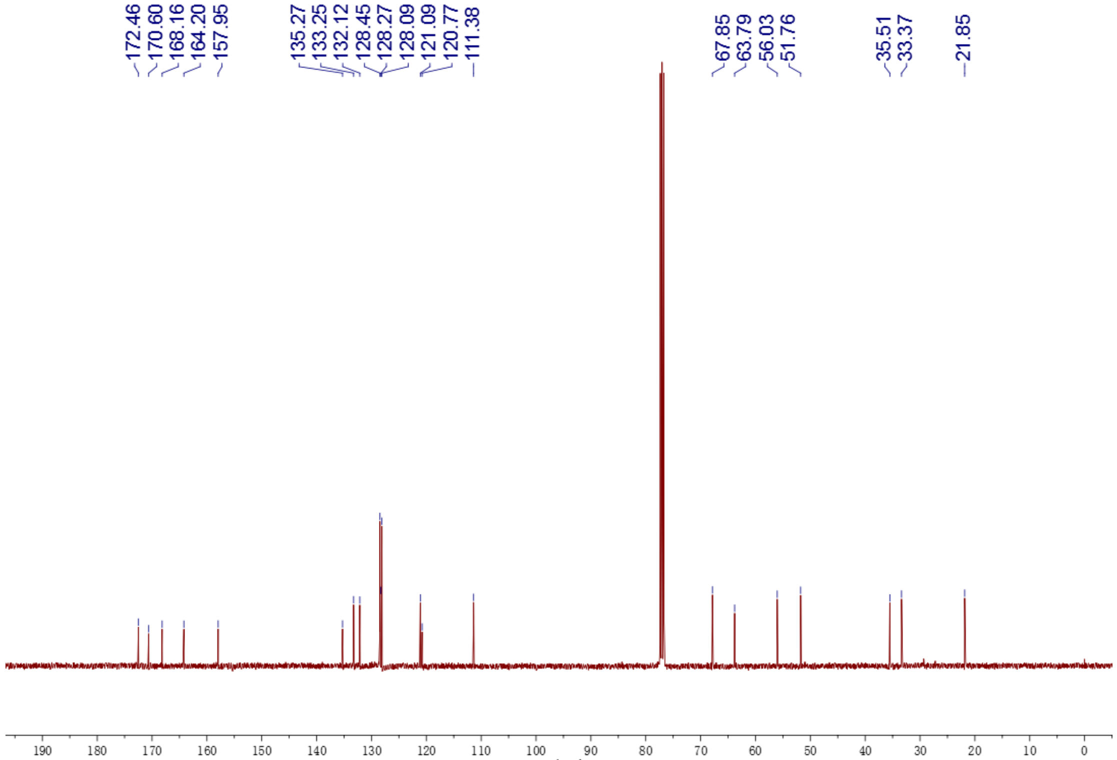

172.46 170.60 168.16 164.20 157.95 135.27 133.25 132.12 128.45 128.27 128.09 121.09 120.77 111.38 67.85 63.79 56.03 51.76 35.51 33.37 21.85
f1 (ppm)

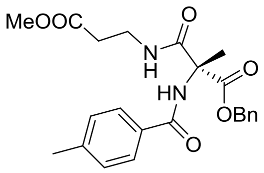
MeOOC
N
H
O
HN
O
OBn
O

2u

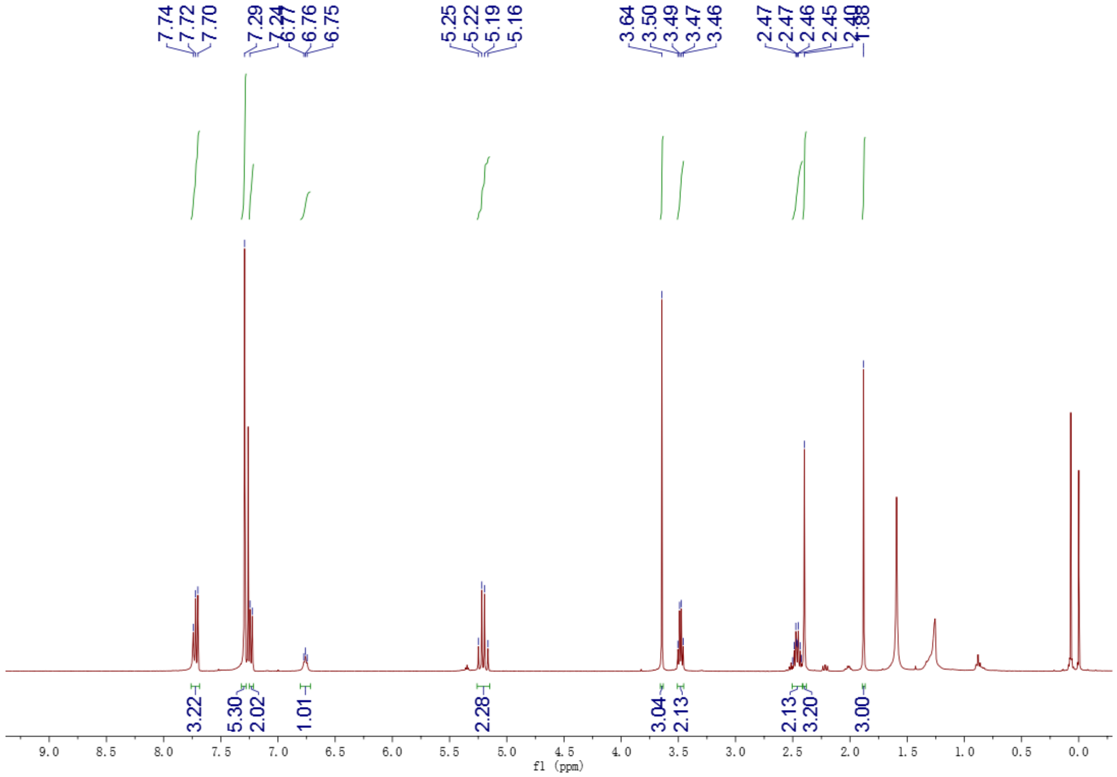
7.74
7.72
7.70
7.29
7.24
6.77
6.76
6.75
5.25
5.22
5.19
5.16
3.64
3.50
3.49
3.47
3.46
2.47
2.47
2.46
2.45
2.40
1.88
3.22
5.30
2.02
1.01
2.28
3.04
2.13
2.13
3.20
3.00
9.0 8.5 8.0 7.5 7.0 6.5 6.0 5.5 5.0 4.5 4.0 3.5 3.0 2.5 2.0 1.5 1.0 0.5 0.0
f1 (ppm)

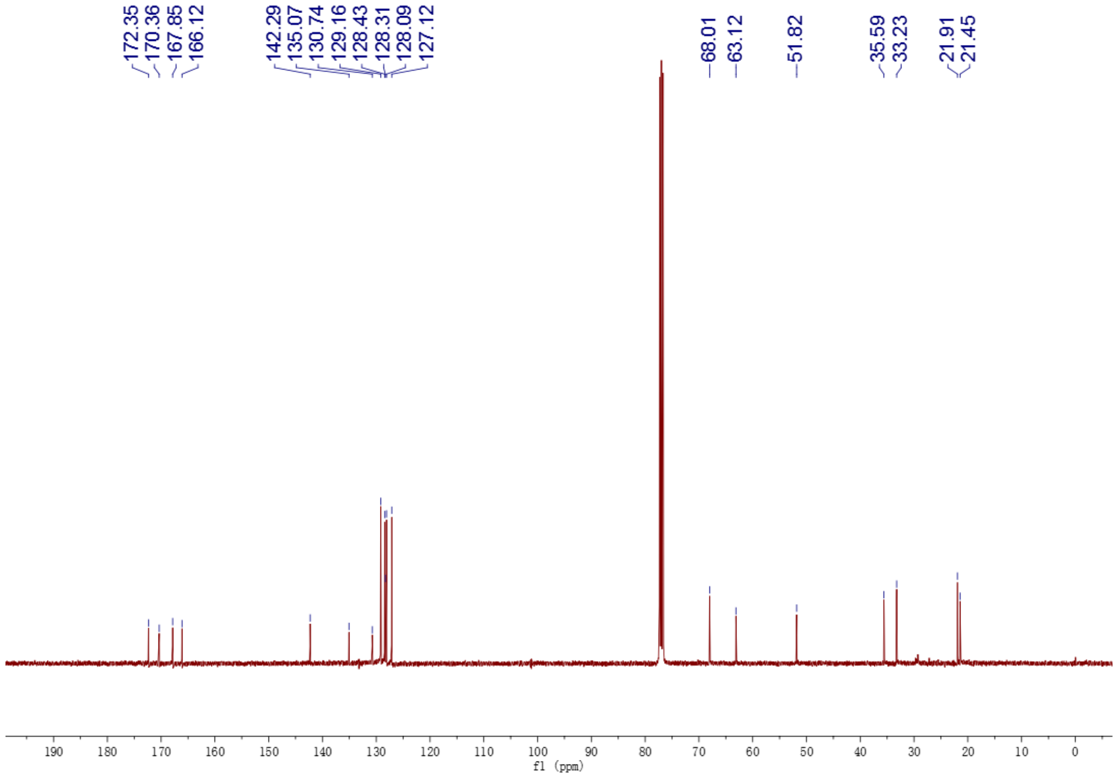
172.35
170.36
167.85
166.12
142.29
135.07
130.74
129.16
128.43
128.31
128.09
127.12
68.01
63.12
51.82
35.59
33.23
21.91
21.45
190 180 170 160 150 140 130 120 110 100 90 80 70 60 50 40 30 20 10 0
f1 (ppm)

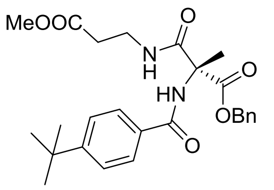
MeOOC
N
H
O
O
HN
OBn
O

2v

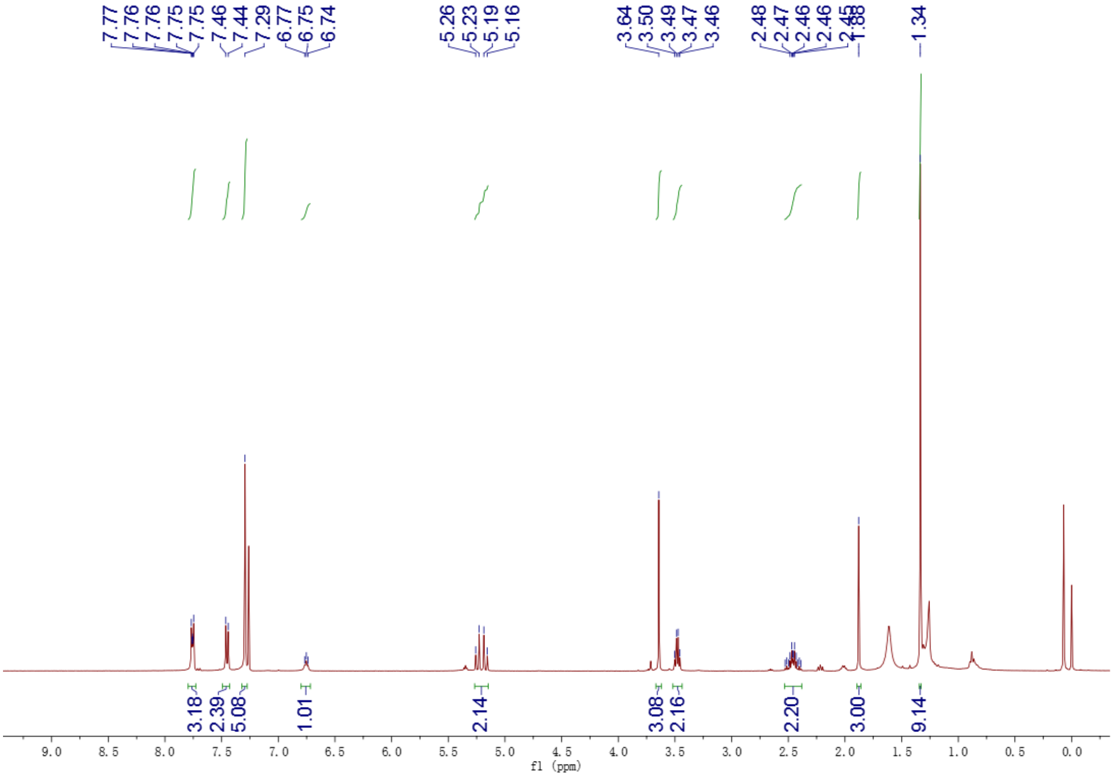
7.77
7.76
7.76
7.75
7.75
7.46
7.44
7.29
6.77
6.75
6.74
5.26
5.23
5.19
5.16
3.64
3.50
3.49
3.47
3.46
2.48
2.47
2.46
2.46
1.88
1.34
3.18
2.39
5.08
1.01
2.14
3.08
2.16
2.20
3.00
9.14
f1 (ppm)

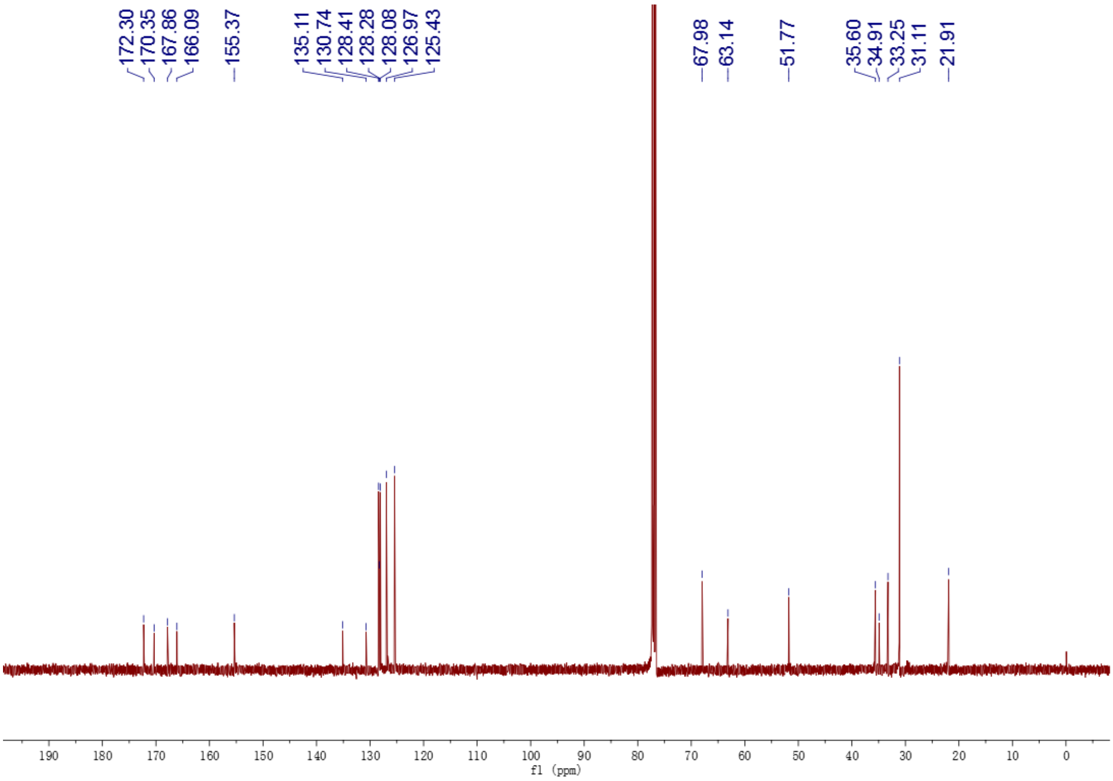
172.30
170.35
167.86
166.09
155.37
135.11
130.74
128.41
128.28
128.08
126.97
125.43
67.98
63.14
51.77
35.60
34.91
33.25
31.11
21.91
f1 (ppm)

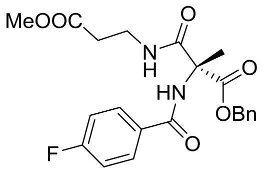
MeOOC
N
H
O
HN
O
OBn
F
O

2w

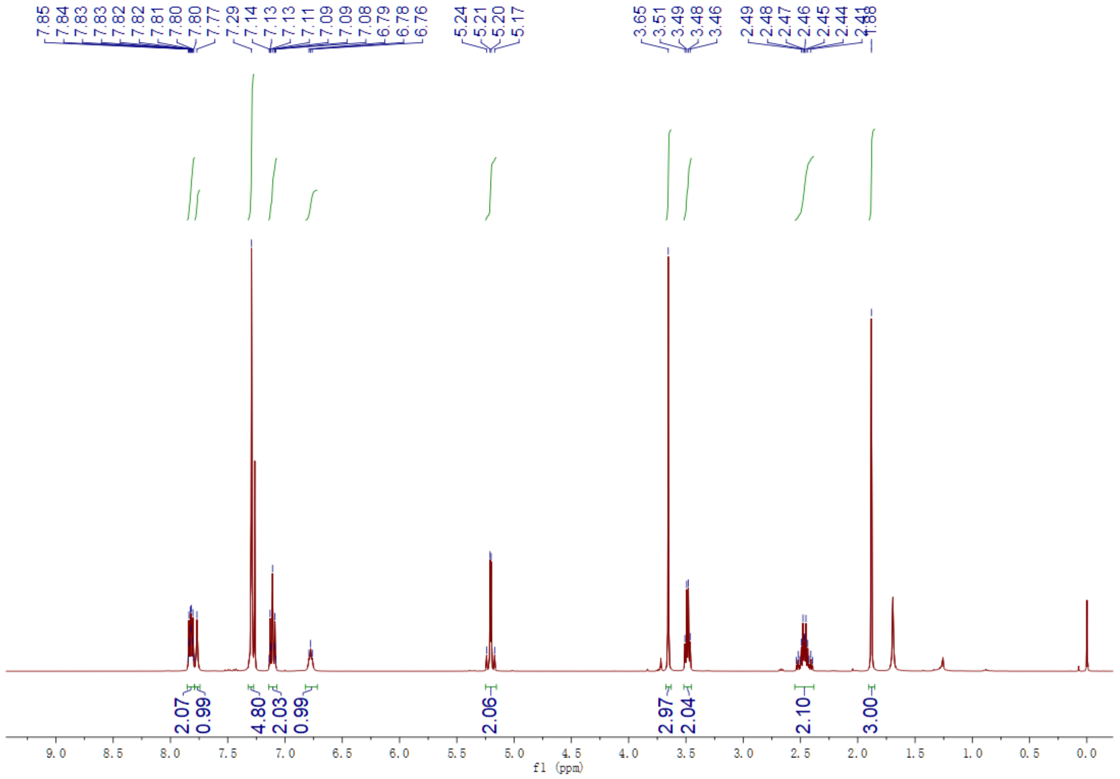
7.85
7.84
7.83
7.83
7.82
7.82
7.81
7.80
7.77
7.29
7.14
7.13
7.13
7.11
7.09
7.09
7.08
6.79
6.78
6.76
5.24
5.21
5.20
5.17
3.65
3.51
3.49
3.48
3.46
2.49
2.48
2.47
2.46
2.45
2.44
2.41
1.88
2.07
0.99
4.80
2.03
0.99
2.06
2.97
2.04
2.10
3.00
f1 (ppm)

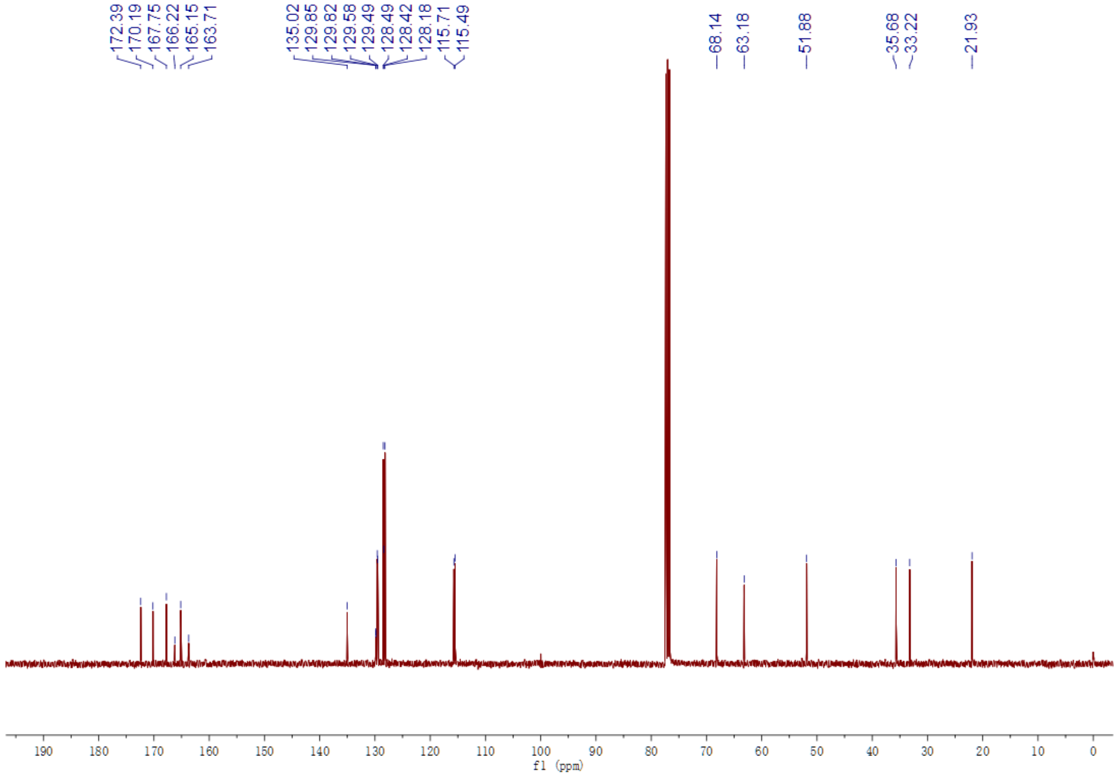
172.39
170.19
167.75
166.22
165.15
163.71
135.02
129.85
129.82
129.58
129.49
128.49
128.42
128.18
115.71
115.49
68.14
63.18
51.88
35.68
33.22
21.93
f1 (ppm)

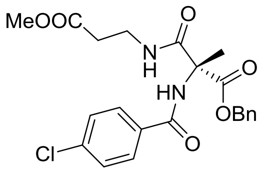
MeOOC
N
H
O
O
HN
OBn
Cl
O

2x

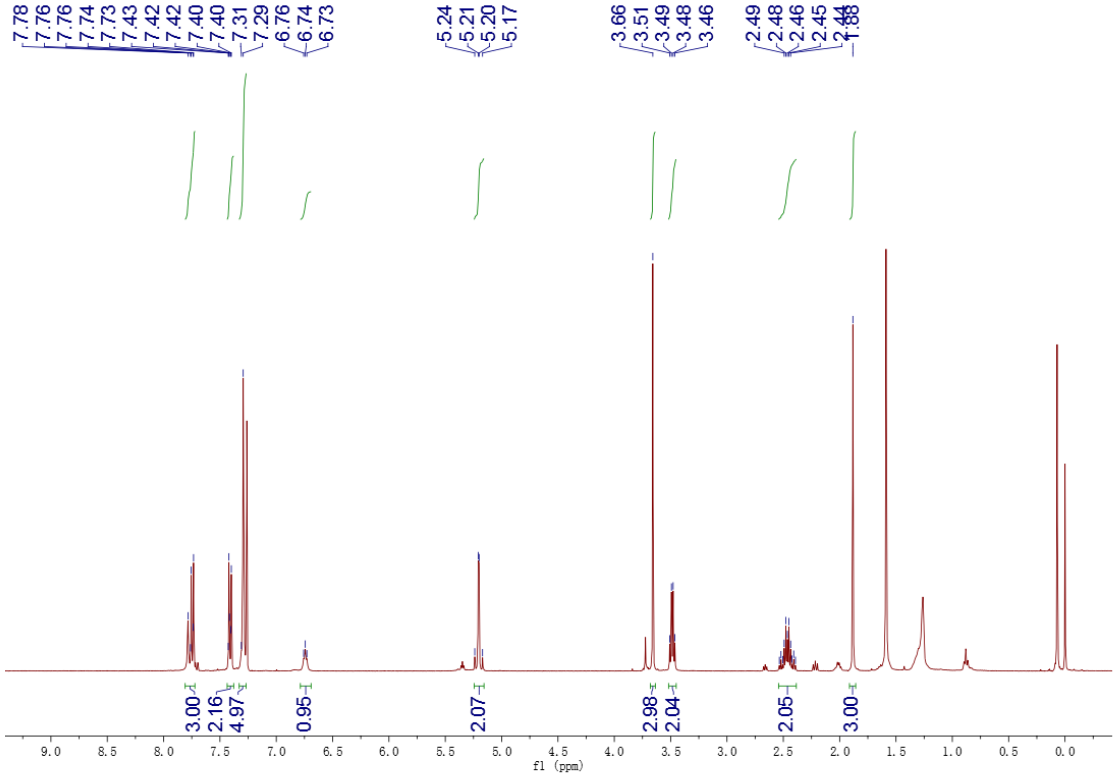
7.78
7.76
7.76
7.74
7.73
7.43
7.42
7.42
7.40
7.40
7.31
7.29
6.76
6.74
6.73
5.24
5.21
5.20
5.17
3.66
3.51
3.49
3.48
3.46
2.49
2.48
2.46
2.45
2.44
1.88
3.00
2.16
4.97
0.95
2.07
2.98
2.04
2.05
3.00
f1 (ppm)

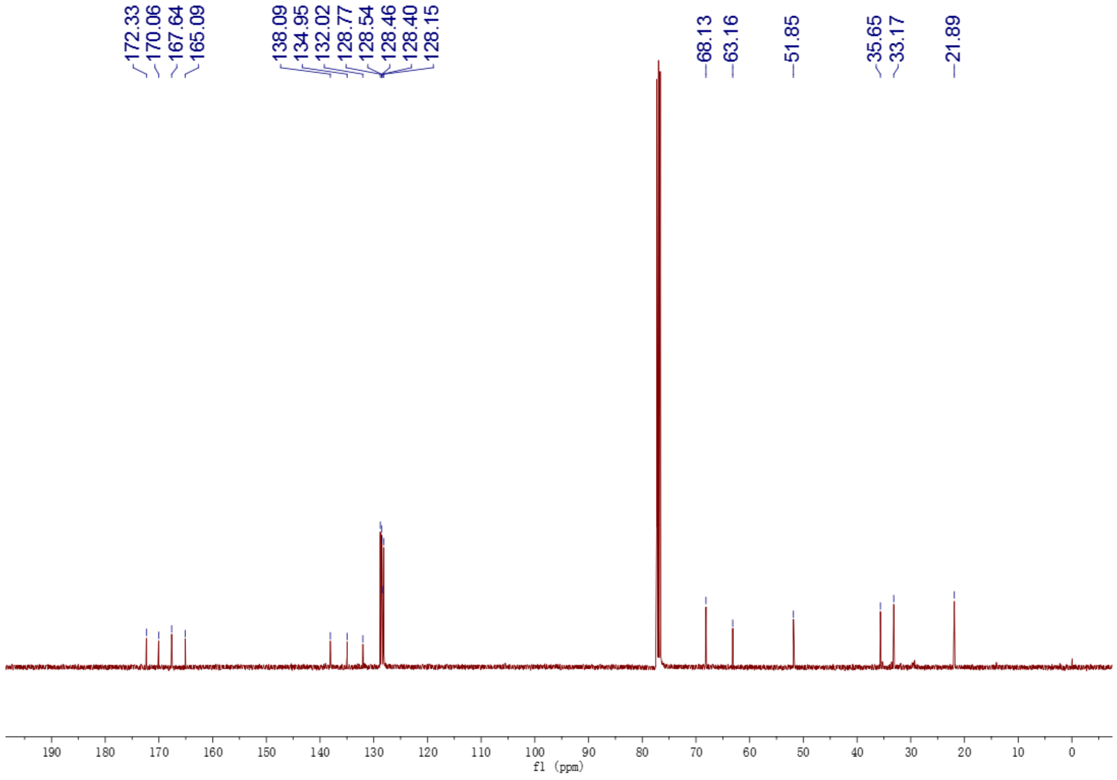
172.33
170.06
167.64
165.09
138.09
134.95
132.02
128.77
128.54
128.46
128.40
128.15
68.13
63.16
51.85
35.65
33.17
21.89
f1 (ppm)

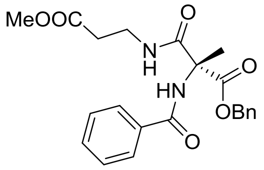
MeOOC
N
H
O
HN
O
OBn
O

2y

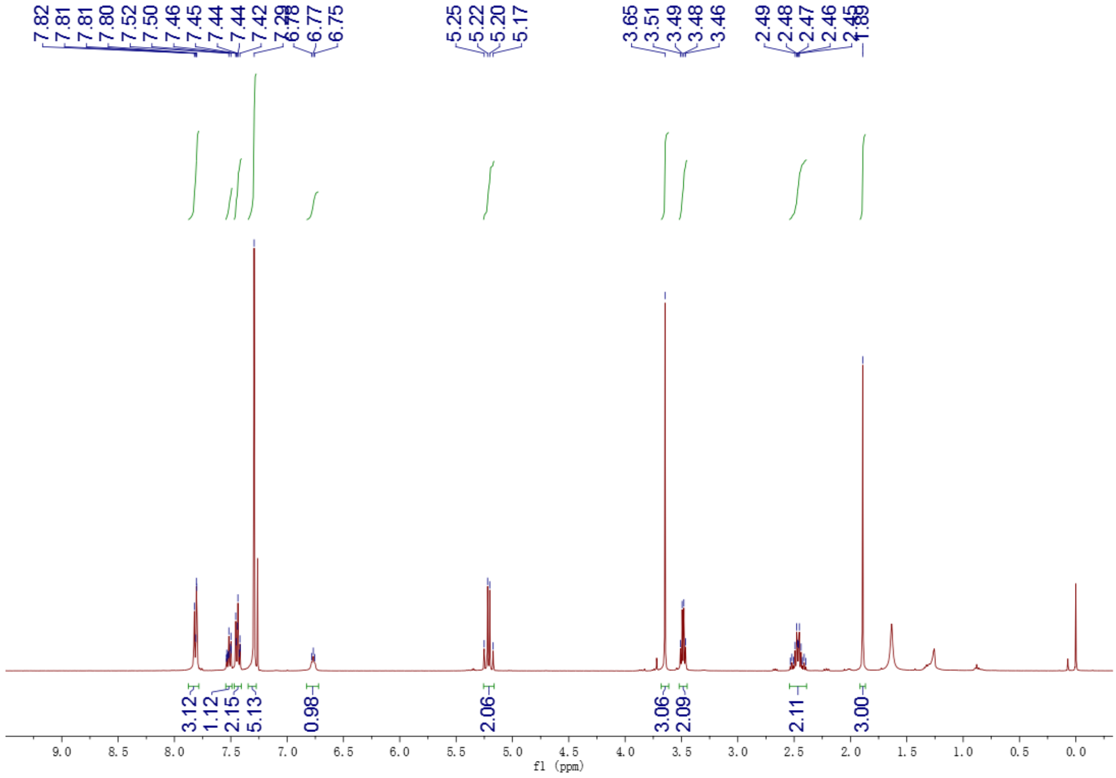
7.82 7.81 7.81 7.80 7.52 7.50 7.46 7.45 7.44 7.44 7.42 7.29 6.78 6.77 6.75
5.25 5.22 5.20 5.17
3.65 3.51 3.49 3.48 3.46
2.49 2.48 2.47 2.46 2.45 1.89
3.12 1.12 2.15 5.13 0.98 2.06 3.06 2.09 2.11 3.00
f1 (ppm)

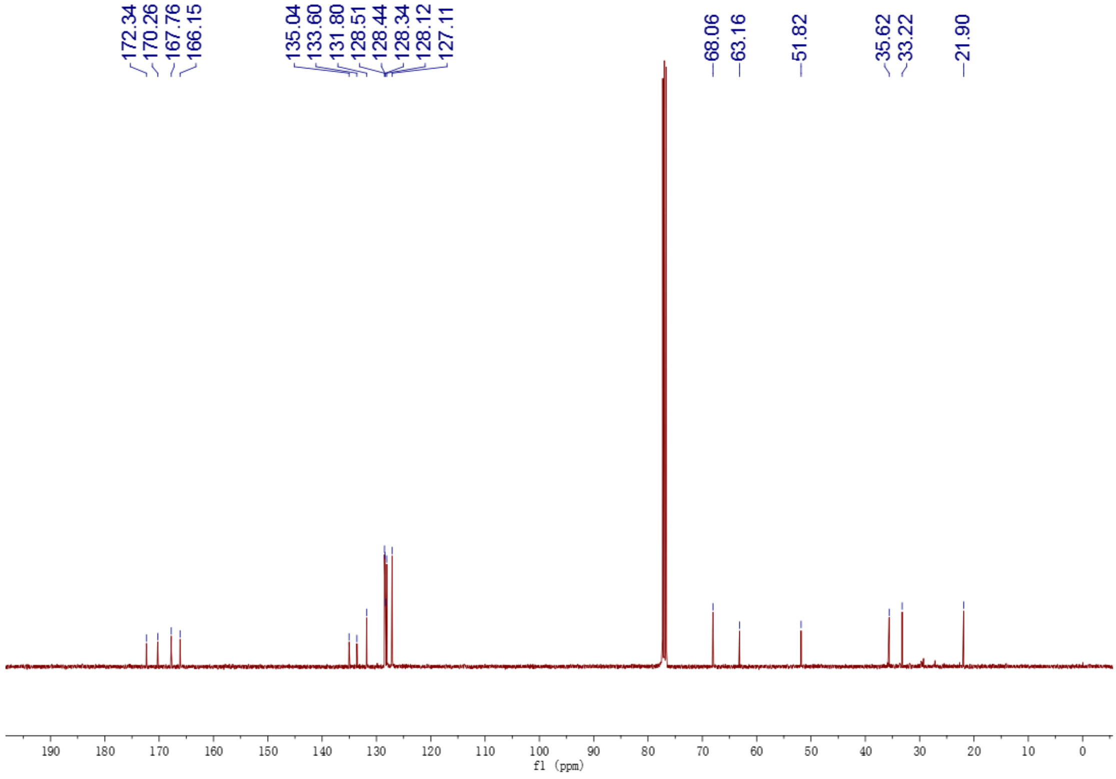
172.34 170.26 167.76 166.15
135.04 133.60 131.80 128.51 128.44 128.34 128.12 127.11
68.06 63.16 51.82 35.62 33.22 21.90
f1 (ppm)

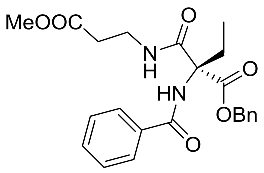
MeOOC
N
H
O
HN
O
OBn
O

2z

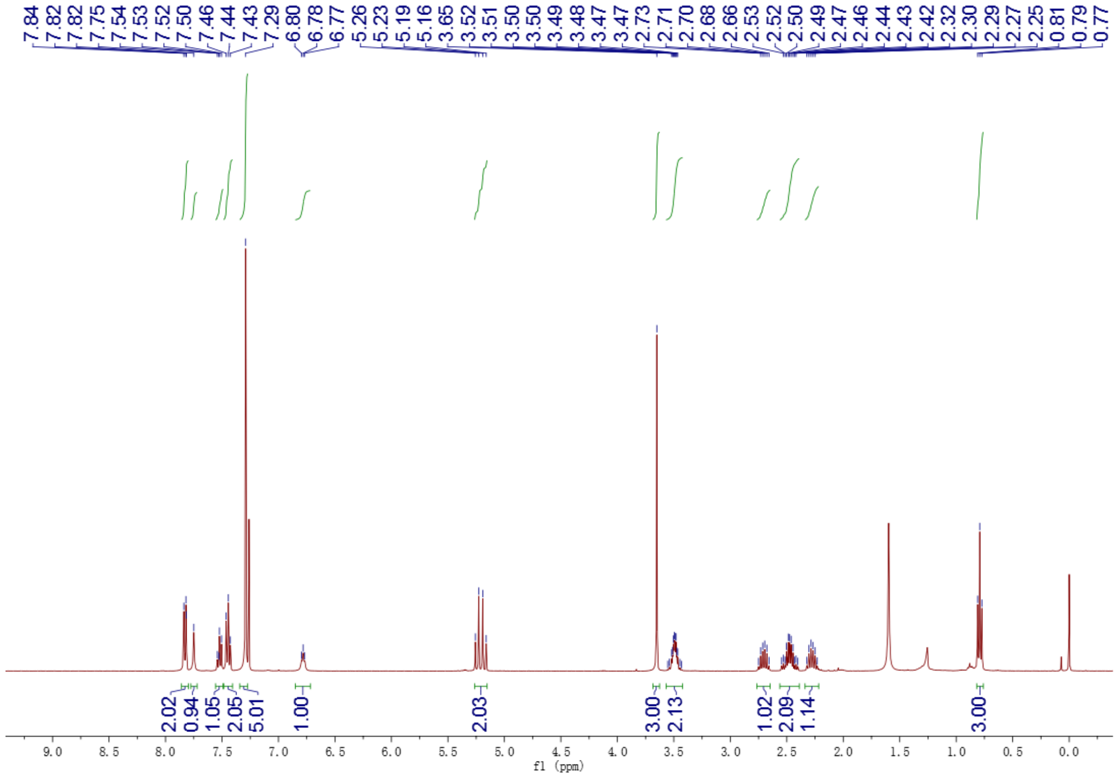
7.84
7.82
7.82
7.75
7.54
7.53
7.52
7.50
7.46
7.44
7.43
7.29
6.80
6.78
6.77
5.26
5.23
5.19
5.16
3.65
3.52
3.51
3.50
3.50
3.49
3.48
3.47
3.47
2.73
2.71
2.70
2.68
2.66
2.53
2.52
2.50
2.49
2.47
2.46
2.44
2.43
2.42
2.32
2.30
2.29
2.27
2.25
0.81
0.79
0.77
2.02
0.94
1.05
2.05
5.01
1.00
2.03
3.00
2.13
1.02
2.09
1.14
3.00
9.0
8.5
8.0
7.5
7.0
6.5
6.0
5.5
5.0
4.5
4.0
3.5
3.0
2.5
2.0
1.5
1.0
0.5
0.0
f1 (ppm)

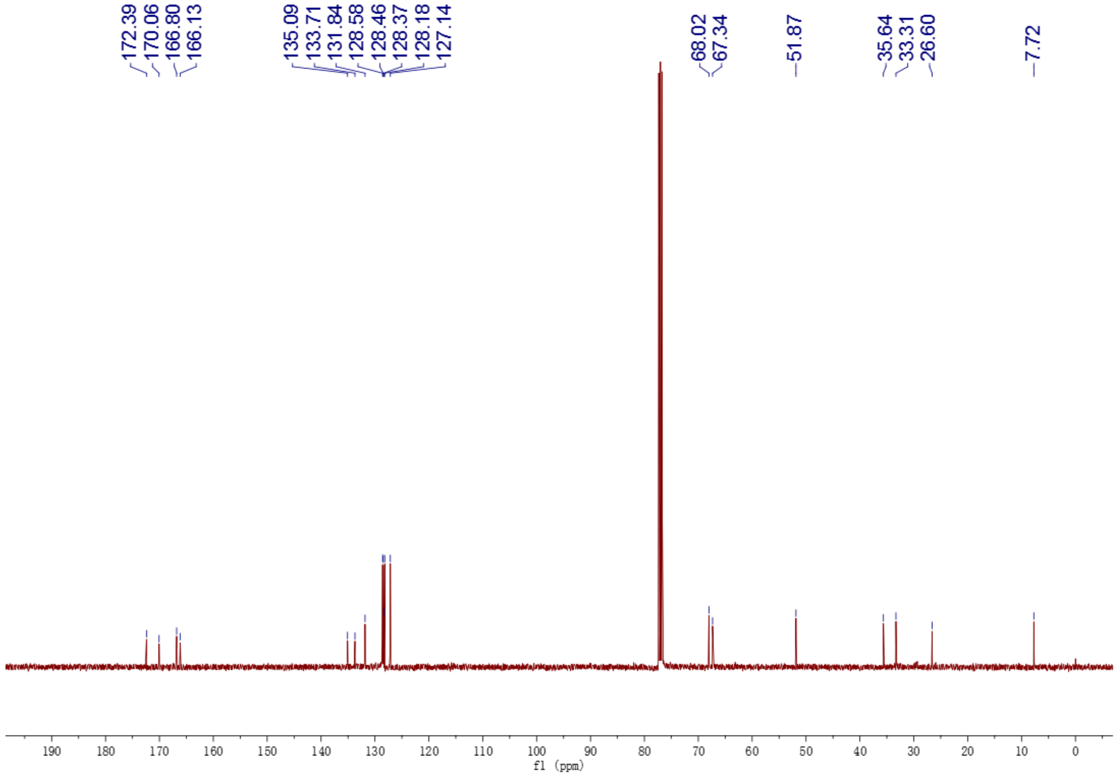
172.39
170.06
166.80
166.13
135.09
133.71
131.84
128.58
128.46
128.37
128.18
127.14
68.02
67.34
51.87
35.64
33.31
26.60
7.72
190
180
170
160
150
140
130
120
110
100
90
80
70
60
50
40
30
20
10
0
f1 (ppm)

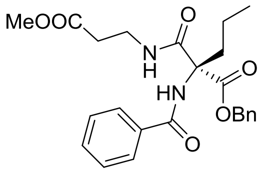
MeOOC
N
H
O
HN
O
OBn
O

2aa

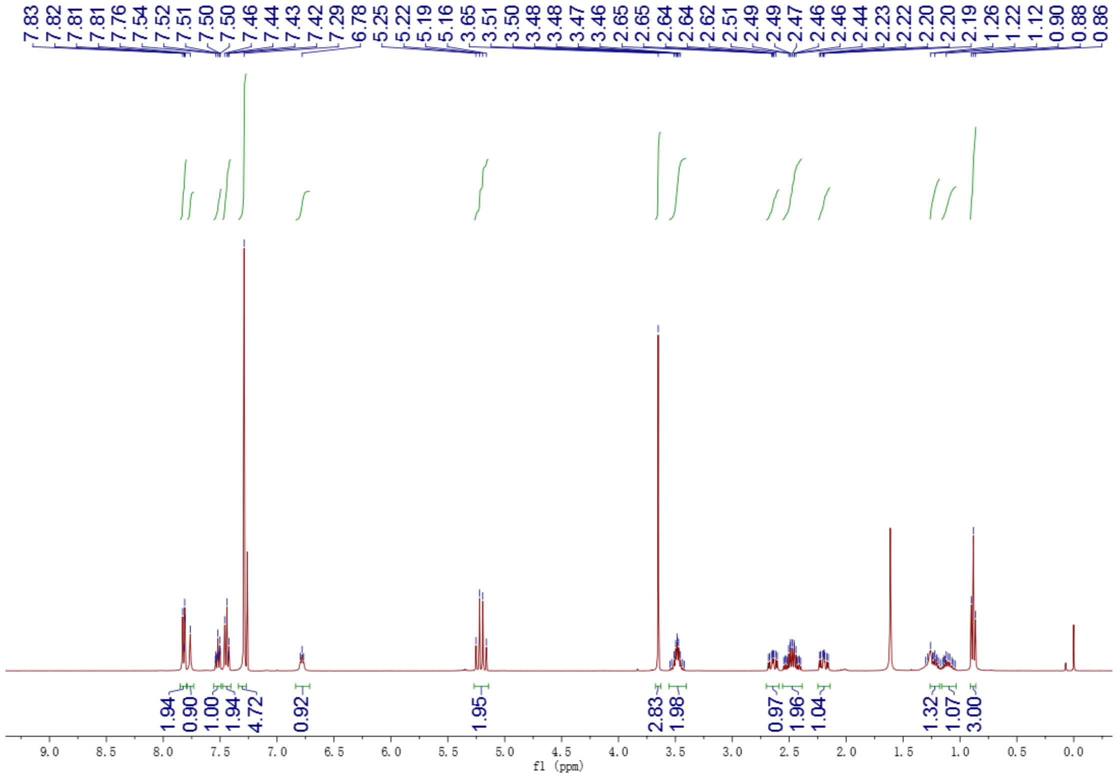
7.83 7.82 7.81 7.81 7.76 7.54 7.52 7.51 7.50 7.50 7.46 7.44 7.43 7.42 7.29 6.78 5.25 5.22 5.19 5.16 3.65 3.51 3.50 3.48 3.48 3.47 3.46 2.65 2.65 2.64 2.64 2.62 2.51 2.49 2.49 2.47 2.46 2.46 2.44 2.23 2.22 2.20 2.20 2.19 1.26 1.22 1.12 0.90 0.88 0.86
1.94 0.90 1.00 1.94 4.72 0.92 1.95 2.83 1.98 0.97 1.96 1.04 1.32 1.07 3.00
9.0 8.5 8.0 7.5 7.0 6.5 6.0 5.5 5.0 4.5 4.0 3.5 3.0 2.5 2.0 1.5 1.0 0.5 0.0
f1 (ppm)

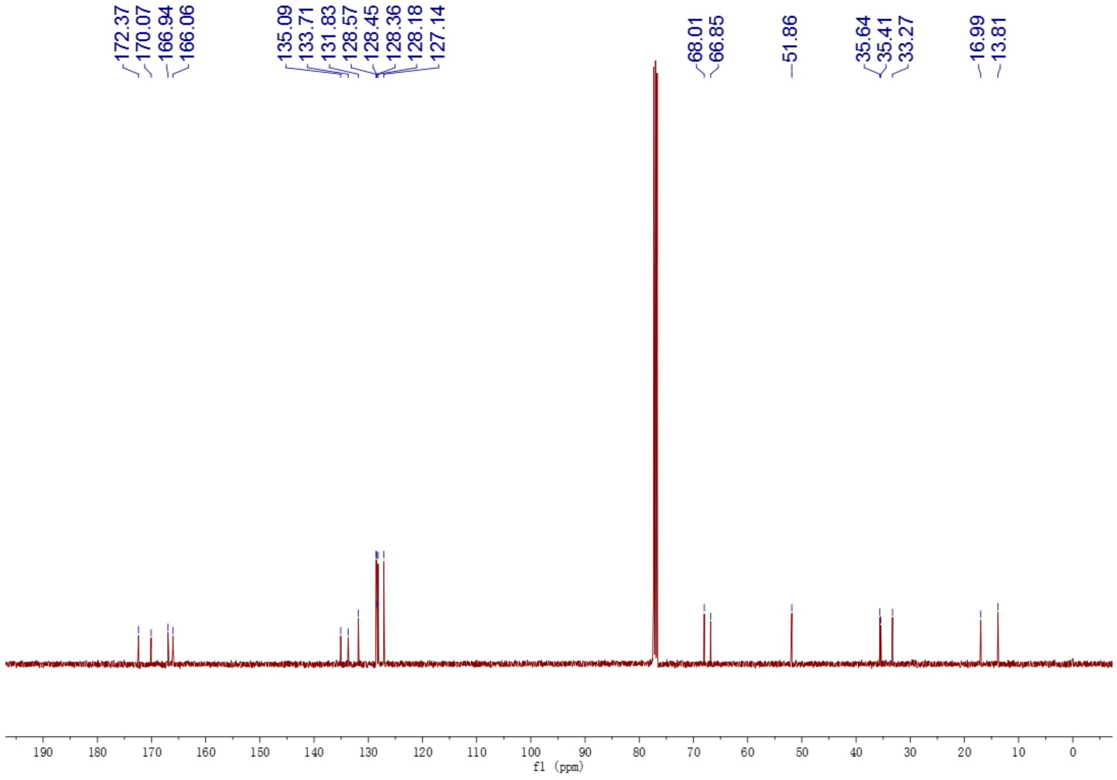
172.37 170.07 166.94 166.06
135.09 133.71 131.83 128.57 128.45 128.36 128.18 127.14
68.01 66.85
51.86
35.64 35.41 33.27
16.99 13.81
190 180 170 160 150 140 130 120 110 100 90 80 70 60 50 40 30 20 10 0
f1 (ppm)

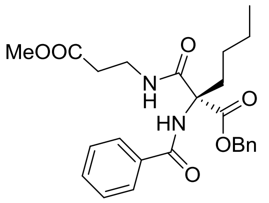
MeOOC
N
H
O
HN
O
OBn
O

2ab

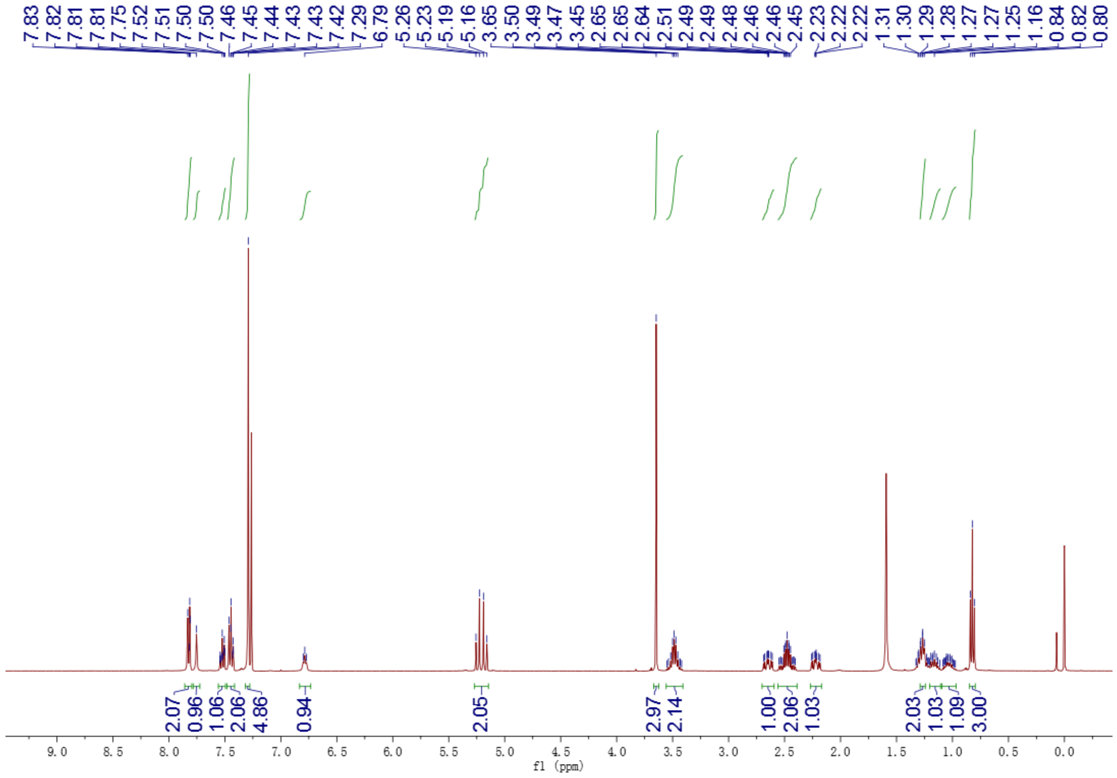
7.83 7.82 7.81 7.81 7.75 7.52 7.51 7.50 7.50 7.46 7.45 7.44 7.43 7.43 7.42 7.29 6.79 5.26 5.23 5.19 5.16 3.65 3.50 3.49 3.47 3.45 2.65 2.65 2.64 2.51 2.49 2.49 2.48 2.46 2.46 2.45 2.23 2.22 2.22 1.31 1.30 1.29 1.28 1.27 1.25 1.16 0.84 0.82 0.80
2.07 0.96 1.06 2.06 4.86 0.94 2.05 2.97 2.14 1.00 2.06 1.03 2.03 1.03 1.09 3.00
9.0 8.5 8.0 7.5 7.0 6.5 6.0 5.5 5.0 4.5 4.0 3.5 3.0 2.5 2.0 1.5 1.0 0.5 0.0
f1 (ppm)

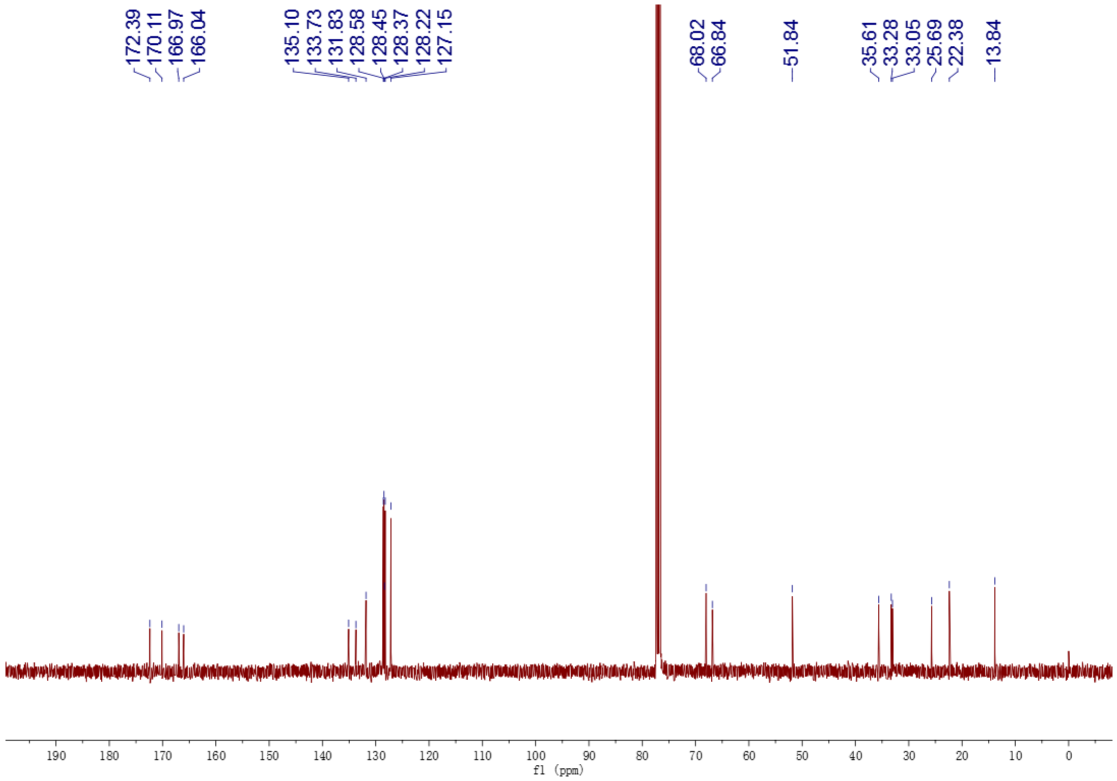
172.39 170.11 166.97 166.04
135.10 133.73 131.83 128.58 128.45 128.37 128.22 127.15
68.02 66.84
51.84
35.61 33.28 33.05 25.69 22.38
13.84
190 180 170 160 150 140 130 120 110 100 90 80 70 60 50 40 30 20 10 0
f1 (ppm)

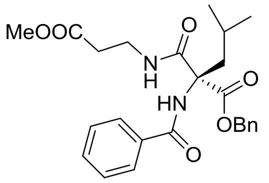
MeOOC
N
H
O
HN
O
OBn
O

2ac

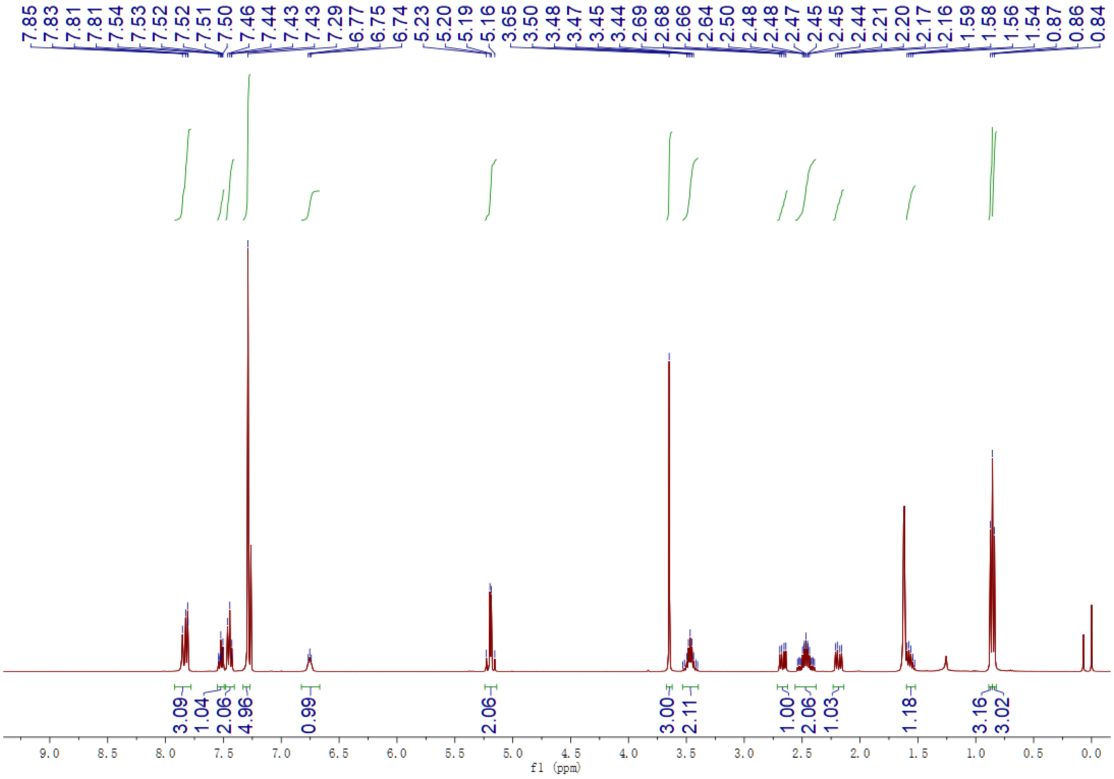
7.85 7.83 7.81 7.81 7.54 7.53 7.52 7.52 7.51 7.50 7.46 7.44 7.43 7.43 7.29 6.77 6.75 6.74 5.23 5.20 5.19 5.16 3.65 3.50 3.48 3.47 3.45 3.44 2.69 2.68 2.66 2.64 2.50 2.48 2.48 2.47 2.45 2.45 2.44 2.21 2.20 2.17 2.16 1.59 1.58 1.56 1.54 0.87 0.86 0.84
3.09 1.04 2.06 4.96 0.99 2.06 3.00 2.11 1.00 2.06 1.03 1.18 3.16 3.02
9.0 8.5 8.0 7.5 7.0 6.5 6.0 5.5 5.0 4.5 4.0 3.5 3.0 2.5 2.0 1.5 1.0 0.5 0.0
f1 (ppm)

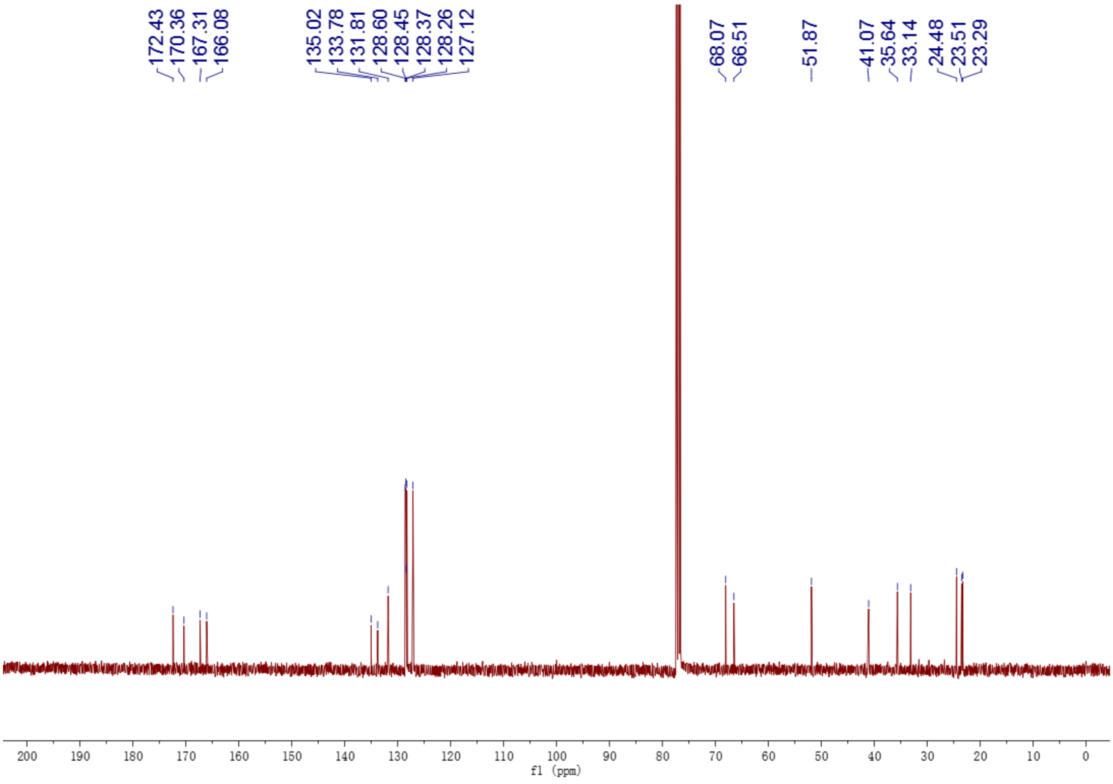
172.43 170.36 167.31 166.08 135.02 133.78 131.81 128.60 128.45 128.37 128.26 127.12 68.07 66.51 51.87 41.07 35.64 33.14 24.48 23.51 23.29
200 190 180 170 160 150 140 130 120 110 100 90 80 70 60 50 40 30 20 10 0
f1 (ppm)

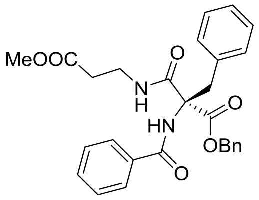
MeOOC
N
H
O
HN
O
OBn
O

2ad

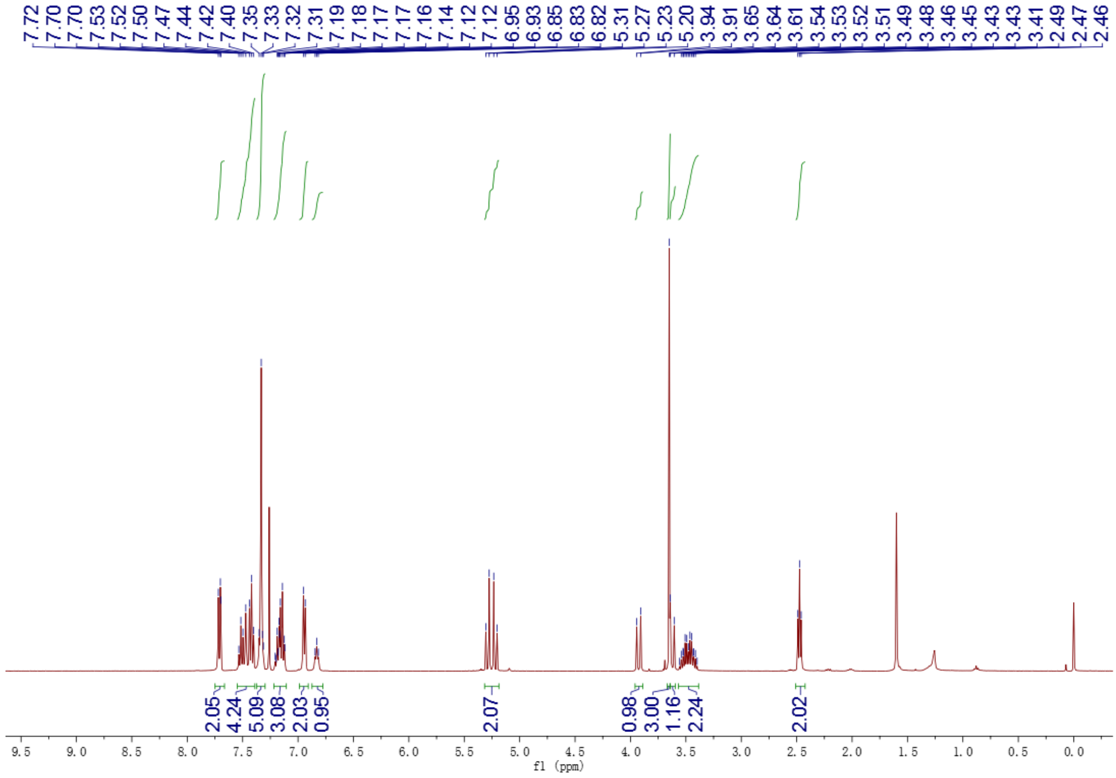
7.72 7.70 7.70 7.53 7.52 7.50 7.47 7.44 7.42 7.40 7.35 7.33 7.32 7.31 7.19 7.18 7.17 7.17 7.16 7.14 7.12 7.12 6.95 6.93 6.85 6.83 6.82 5.31 5.27 5.23 5.20 3.94 3.91 3.65 3.64 3.61 3.54 3.53 3.52 3.51 3.49 3.48 3.46 3.45 3.43 3.43 3.41 2.49 2.47 2.46
2.05 4.24 5.09 3.08 2.03 0.95 2.07 0.98 3.00 1.16 2.24 2.02
9.5 9.0 8.5 8.0 7.5 7.0 6.5 6.0 5.5 5.0 4.5 4.0 3.5 3.0 2.5 2.0 1.5 1.0 0.5 0.0
f1 (ppm)

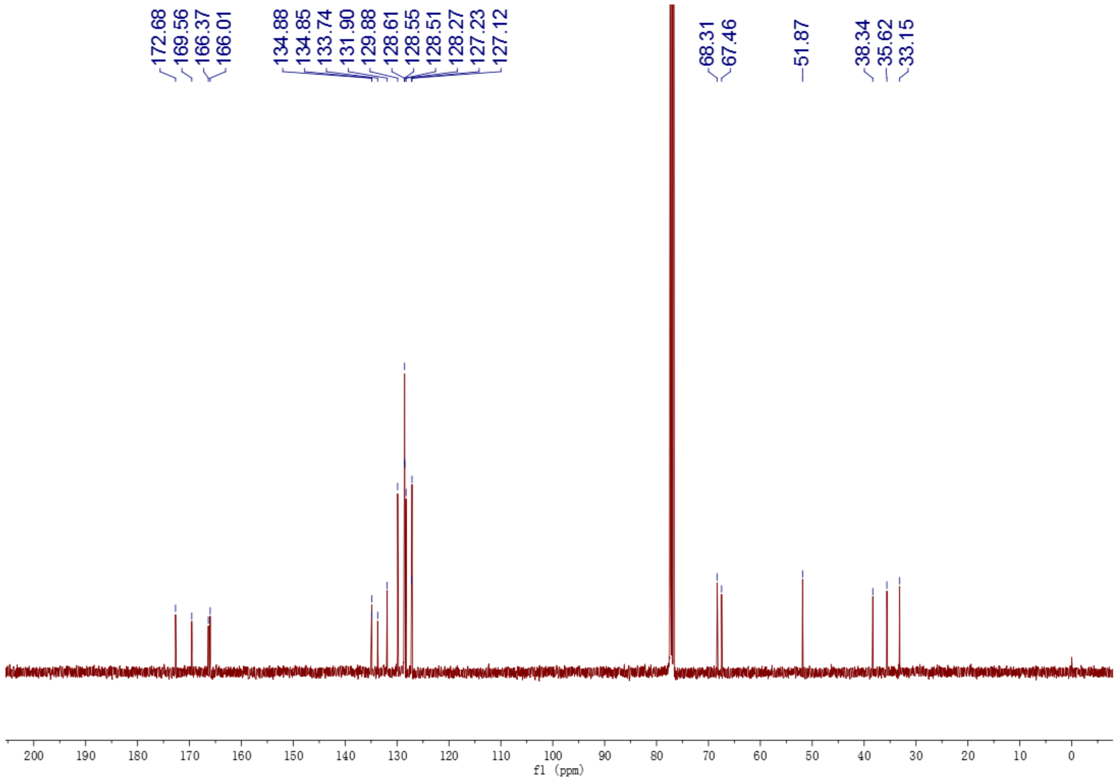
172.68 169.56 166.37 166.01
134.88 134.85 133.74 131.90 129.88 128.61 128.55 128.51 128.27 127.23 127.12
68.31 67.46
51.87
38.34 35.62 33.15
200 190 180 170 160 150 140 130 120 110 100 90 80 70 60 50 40 30 20 10 0
f1 (ppm)

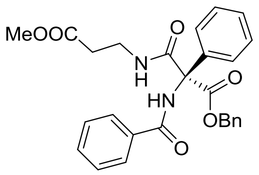
MeOOC
N
H
O
HN
O
OBn
O

2ae

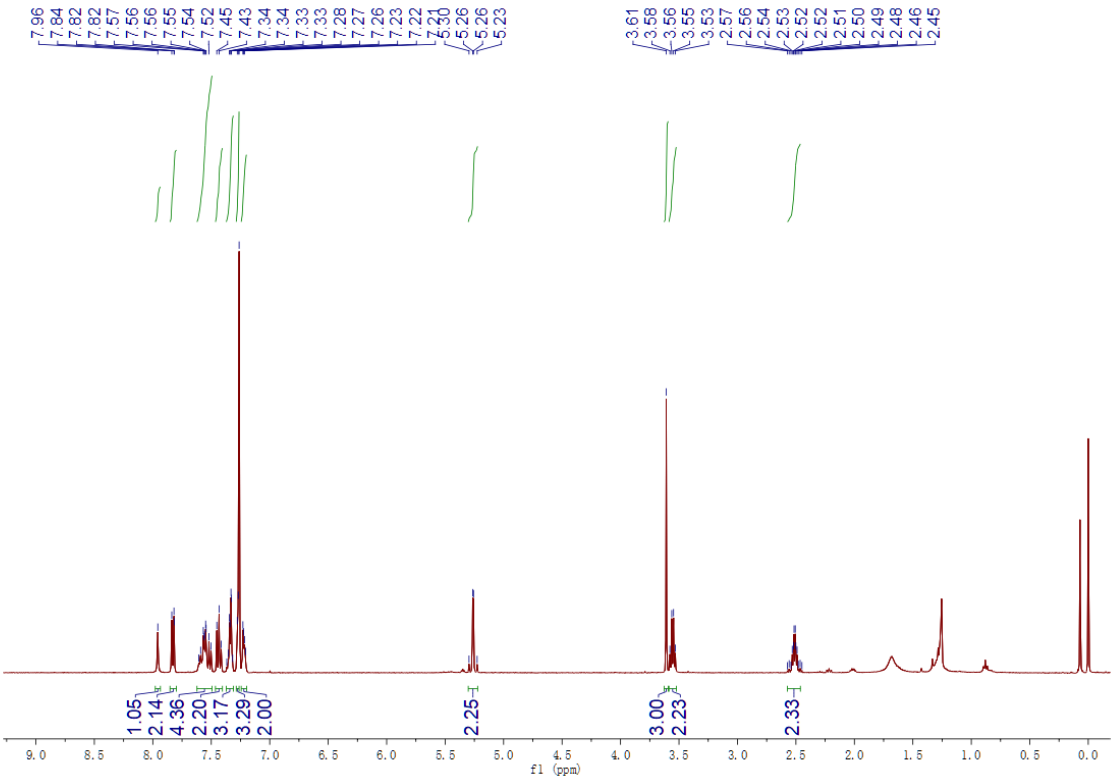
7.96
7.84
7.82
7.82
7.57
7.56
7.56
7.55
7.54
7.52
7.45
7.43
7.34
7.34
7.33
7.33
7.28
7.27
7.26
7.23
7.22
7.21
5.30
5.26
5.26
5.23
3.61
3.58
3.56
3.55
3.53
2.57
2.56
2.54
2.53
2.52
2.52
2.51
2.50
2.49
2.48
2.46
2.45
1.05
2.14
4.36
2.20
3.17
3.29
2.00
2.25
3.00
2.23
2.33
9.0 8.5 8.0 7.5 7.0 6.5 6.0 5.5 5.0 4.5 4.0 3.5 3.0 2.5 2.0 1.5 1.0 0.5 0.0
f1 (ppm)

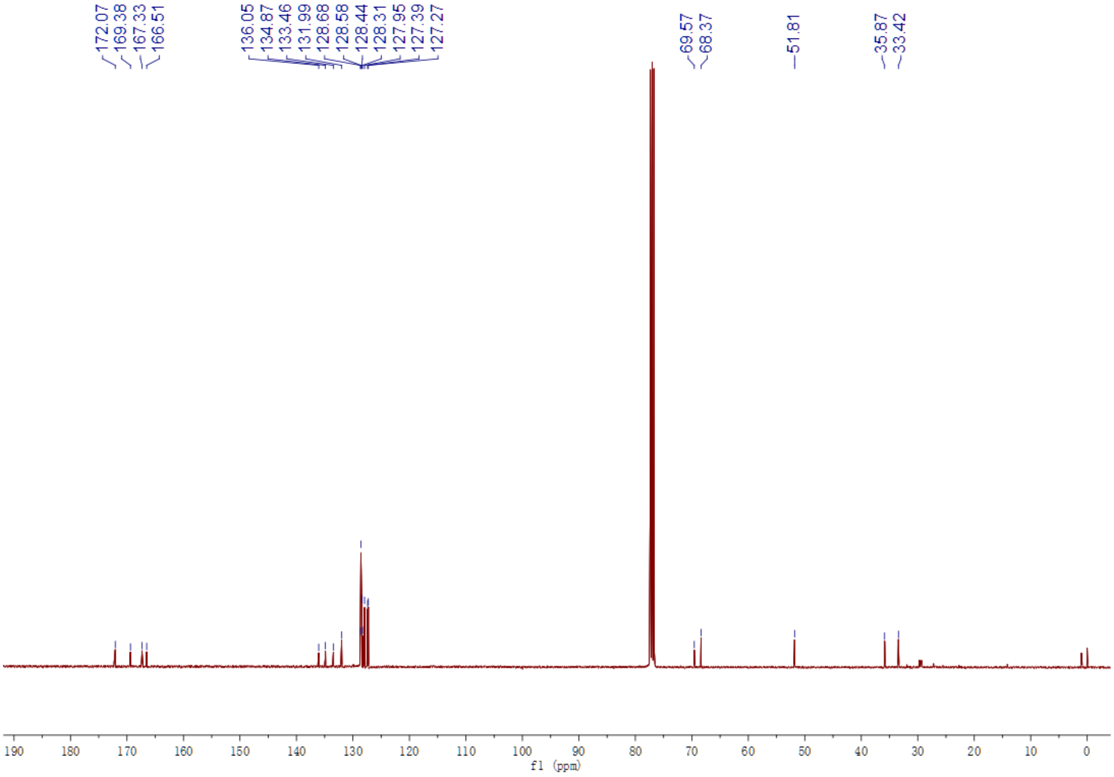
172.07
169.38
167.33
166.51
136.05
134.87
133.46
131.99
128.68
128.58
128.44
128.31
127.95
127.39
127.27
69.57
68.37
51.81
35.87
33.42
190 180 170 160 150 140 130 120 110 100 90 80 70 60 50 40 30 20 10 0
f1 (ppm)

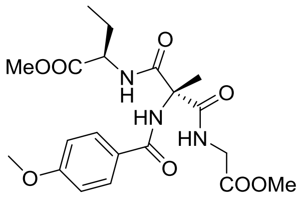
MeOOC
N
H
O
HN
O
HN
O
COOMe

7a

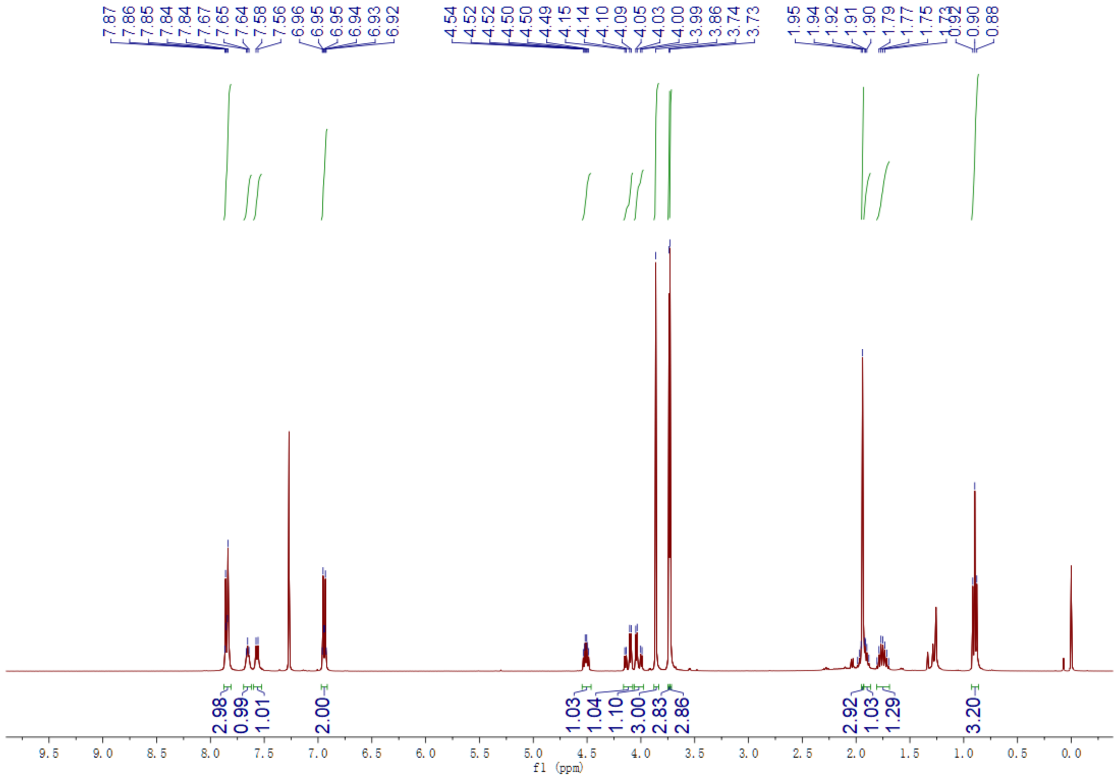
7.87
7.86
7.85
7.84
7.84
7.67
7.65
7.64
7.58
7.56
6.96
6.95
6.95
6.94
6.93
6.92
4.54
4.52
4.52
4.50
4.50
4.49
4.15
4.14
4.10
4.09
4.05
4.03
4.00
3.99
3.86
3.74
3.73
1.95
1.94
1.92
1.91
1.90
1.79
1.77
1.75
1.73
0.92
0.90
0.88
2.98
0.99
1.01
2.00
1.03
1.04
1.10
3.00
2.83
2.86
2.92
1.03
1.29
3.20
9.5 9.0 8.5 8.0 7.5 7.0 6.5 6.0 5.5 5.0 4.5 4.0 3.5 3.0 2.5 2.0 1.5 1.0 0.5 0.0
f1 (ppm)

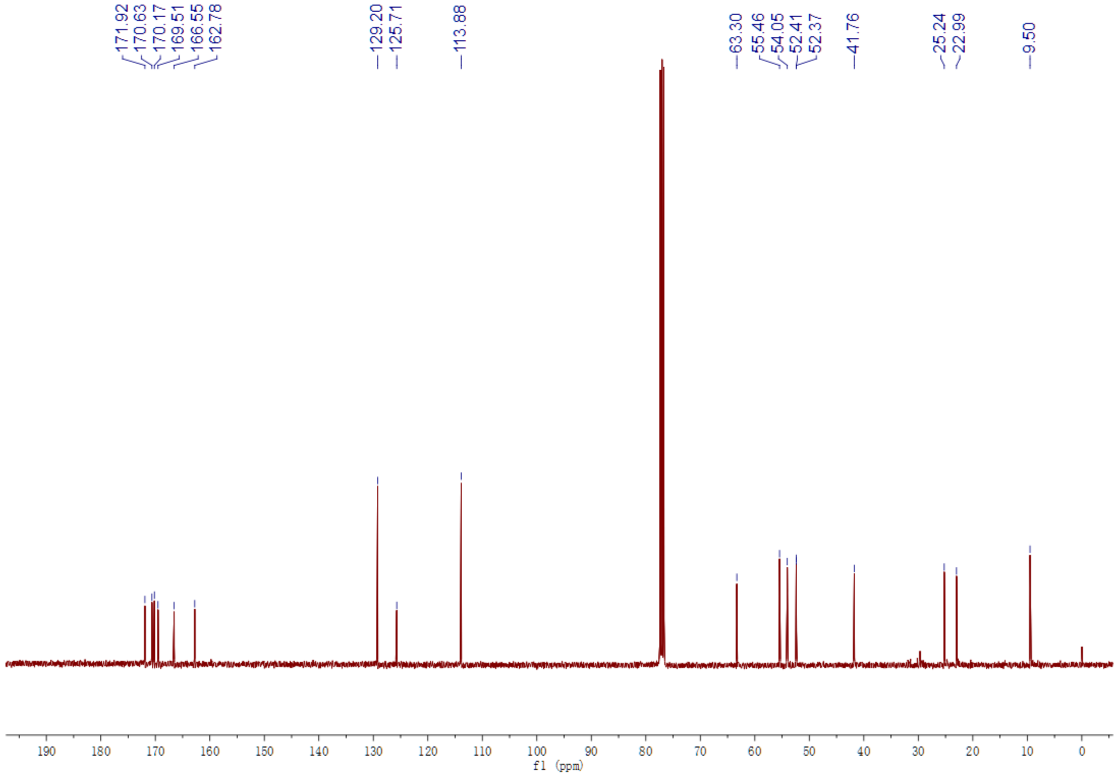
171.92
170.63
170.17
169.51
166.55
162.78
129.20
125.71
113.88
63.30
55.46
54.05
52.41
52.37
41.76
25.24
22.99
9.50
190 180 170 160 150 140 130 120 110 100 90 80 70 60 50 40 30 20 10 0
f1 (ppm)

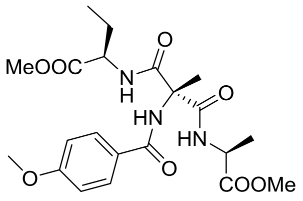
MeOOC
N
H
O
O
HN
HN
O
O
COOMe

7b

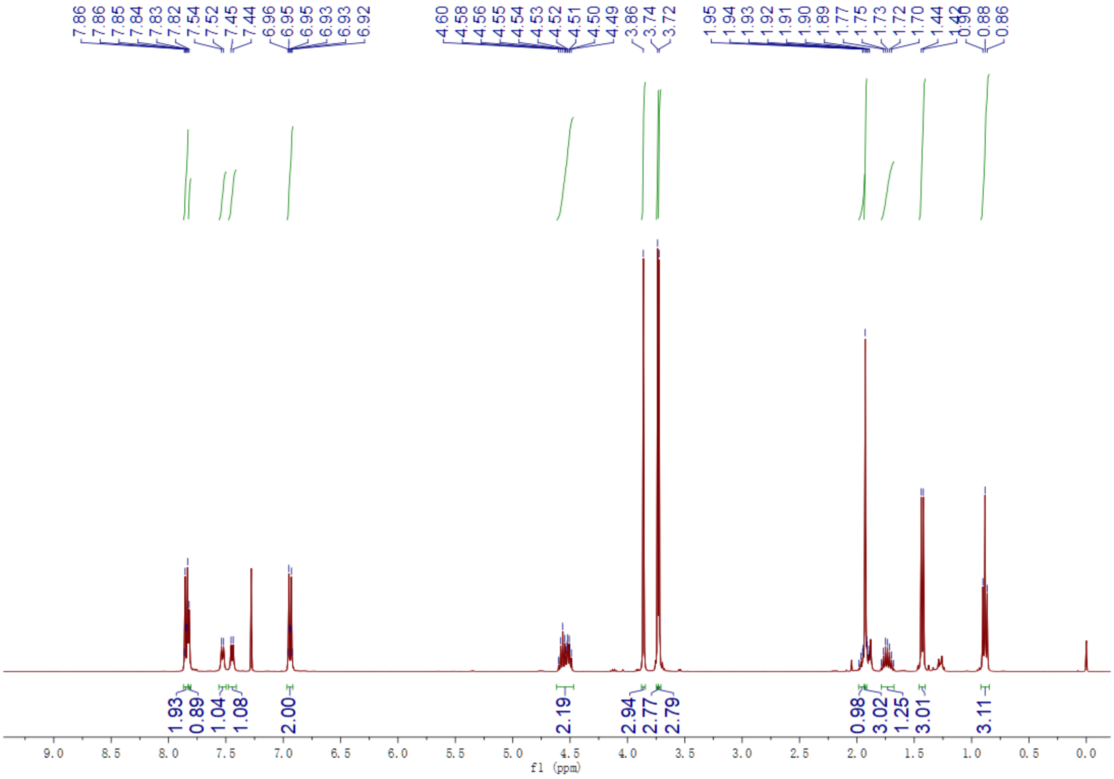
7.86
7.86
7.85
7.84
7.83
7.82
7.54
7.52
7.45
7.44
6.96
6.95
6.95
6.93
6.93
6.92
4.60
4.58
4.56
4.55
4.54
4.53
4.52
4.51
4.50
4.49
3.86
3.74
3.72
1.95
1.94
1.93
1.92
1.91
1.90
1.89
1.77
1.75
1.73
1.72
1.70
1.44
1.42
0.90
0.88
0.86
1.93
0.89
1.04
1.08
2.00
2.19
2.94
2.77
2.79
0.98
3.02
1.25
3.01
3.11
f1 (ppm)

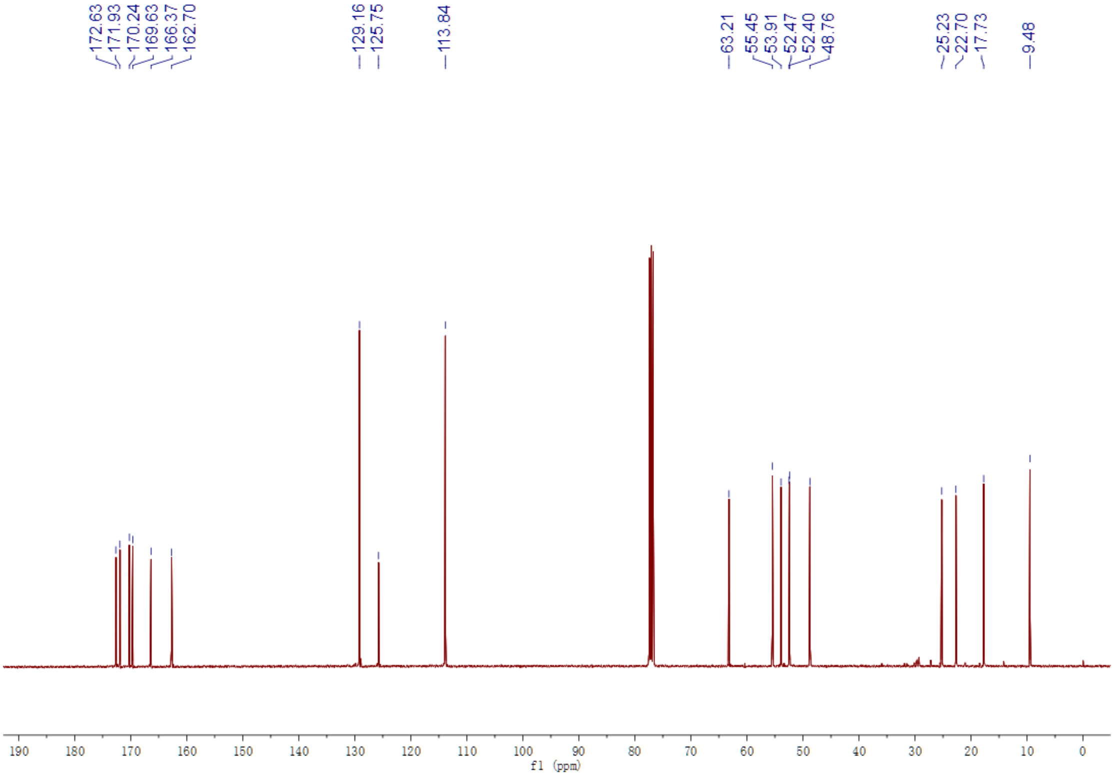
172.63
171.93
170.24
169.63
166.37
162.70
129.16
125.75
113.84
63.21
55.45
53.91
52.47
52.40
48.76
25.23
22.70
17.73
9.48
f1 (ppm)

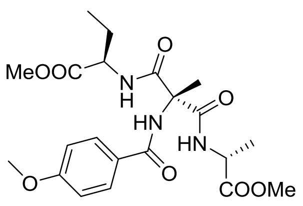
MeOOC
N
H
O
HN
O
HN
COOMe
O
O

7c

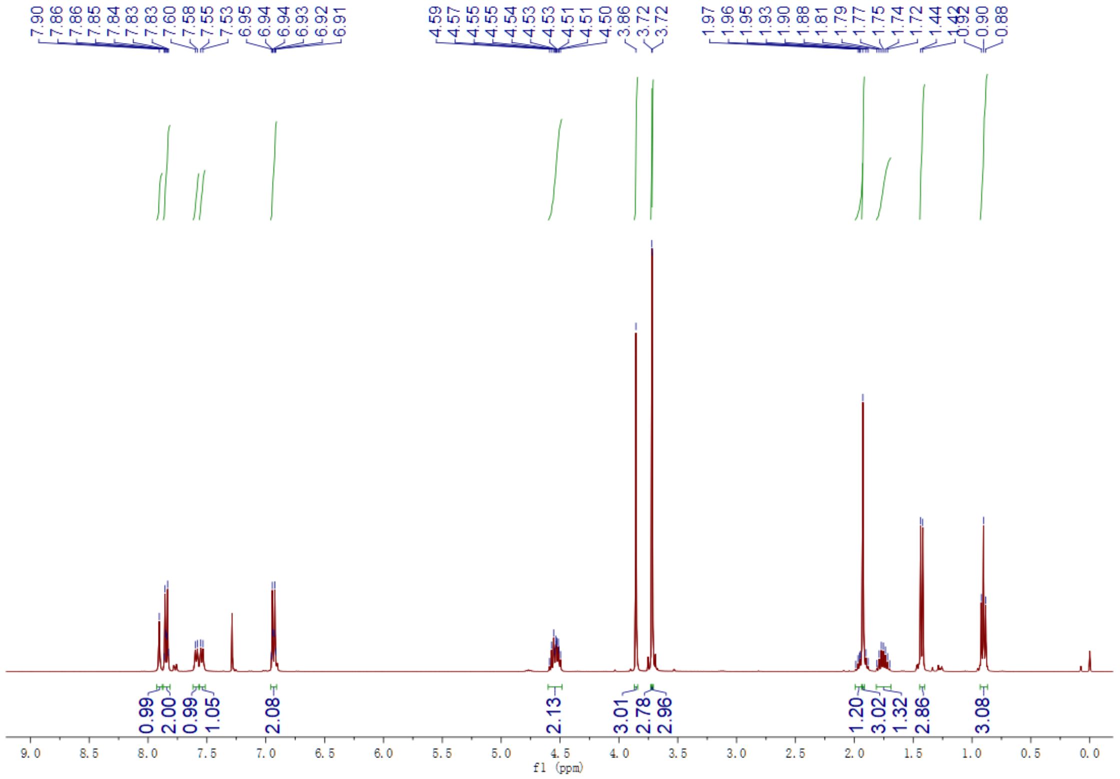
7.90
7.86
7.86
7.85
7.84
7.83
7.83
7.60
7.58
7.55
7.53
6.95
6.94
6.94
6.93
6.92
6.91
4.59
4.57
4.55
4.55
4.54
4.53
4.53
4.51
4.51
4.50
3.86
3.72
3.72
1.97
1.96
1.95
1.93
1.90
1.88
1.81
1.79
1.77
1.75
1.74
1.72
1.44
1.42
0.92
0.90
0.88
0.99
2.00
0.99
1.05
2.08
2.13
3.01
2.78
2.96
1.20
3.02
1.32
2.86
3.08
f1 (ppm)

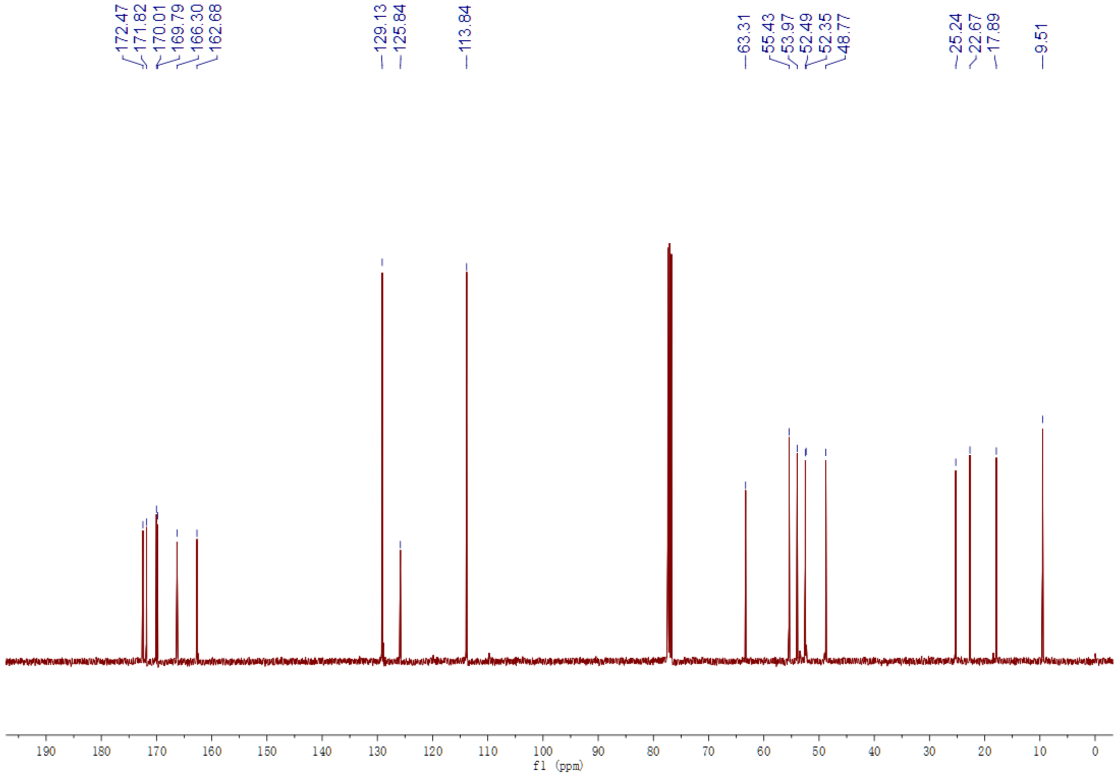
172.47
171.82
170.01
169.79
166.30
162.68
129.13
125.84
113.84
63.31
55.43
53.97
52.49
52.35
48.77
25.24
22.67
17.89
9.51
f1 (ppm)

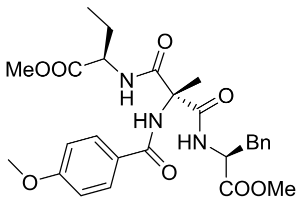

7d

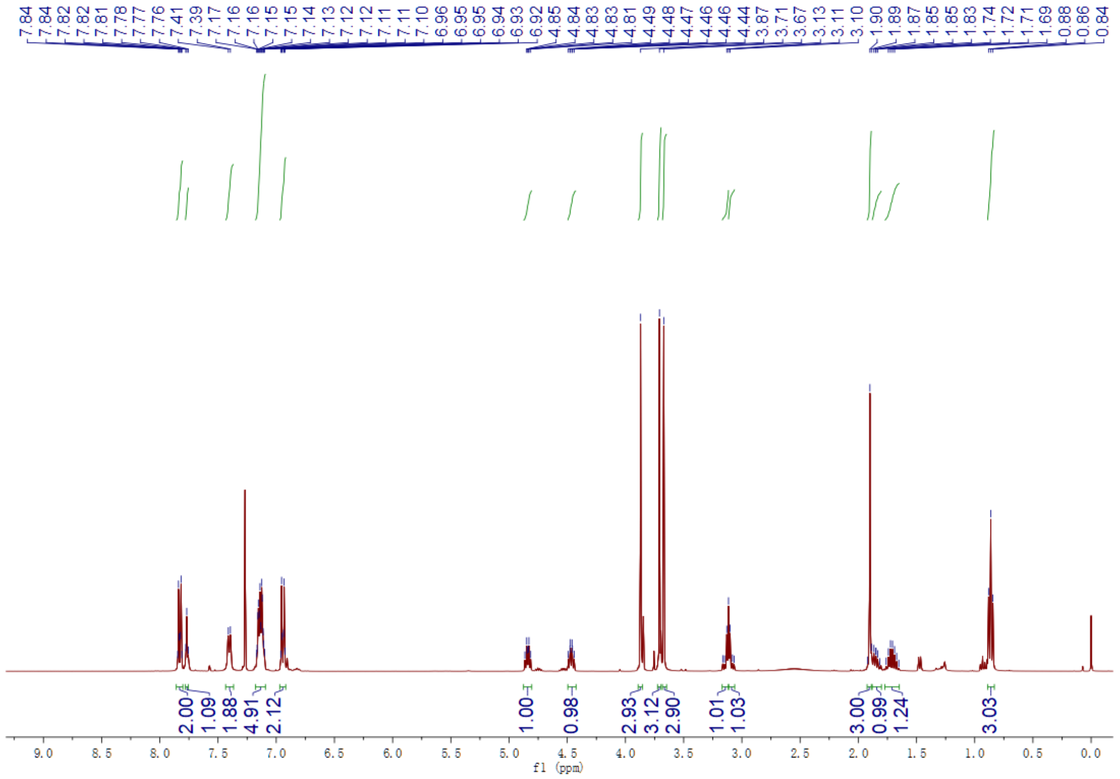

7.84 7.84 7.82 7.82 7.81 7.78 7.77 7.76 7.41 7.39 7.17 7.16 7.16 7.15 7.15 7.14 7.13 7.12 7.12 7.11 7.11 7.10 6.96 6.95 6.95 6.94 6.93 6.92 4.85 4.84 4.83 4.83 4.81 4.49 4.48 4.47 4.46 4.46 4.44 3.87 3.71 3.67 3.13 3.11 3.10 1.90 1.89 1.87 1.85 1.85 1.83 1.74 1.72 1.71 1.69 0.88 0.86 0.84
2.00 1.09 1.88 4.91 2.12 1.00 0.98 2.93 3.12 2.90 1.01 1.03 3.00 0.99 1.24 3.03
f1 (ppm)

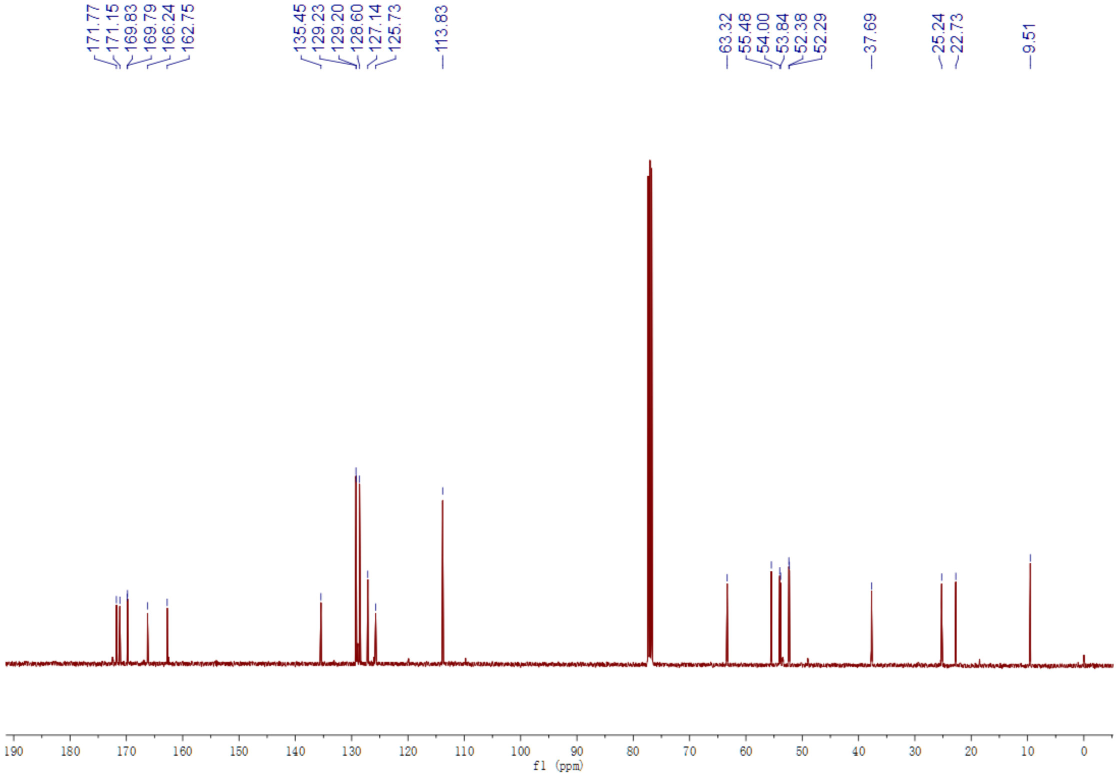

171.77 171.15 169.83 169.79 166.24 162.75 135.45 129.23 129.20 128.60 127.14 125.73 113.83 63.32 55.48 54.00 53.84 52.38 52.29 37.69 25.24 22.73 9.51
f1 (ppm)

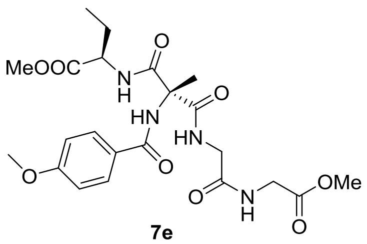
MeOOC
H
N
O
HN
HN
O
O
O
N
H
OMe
O

7e

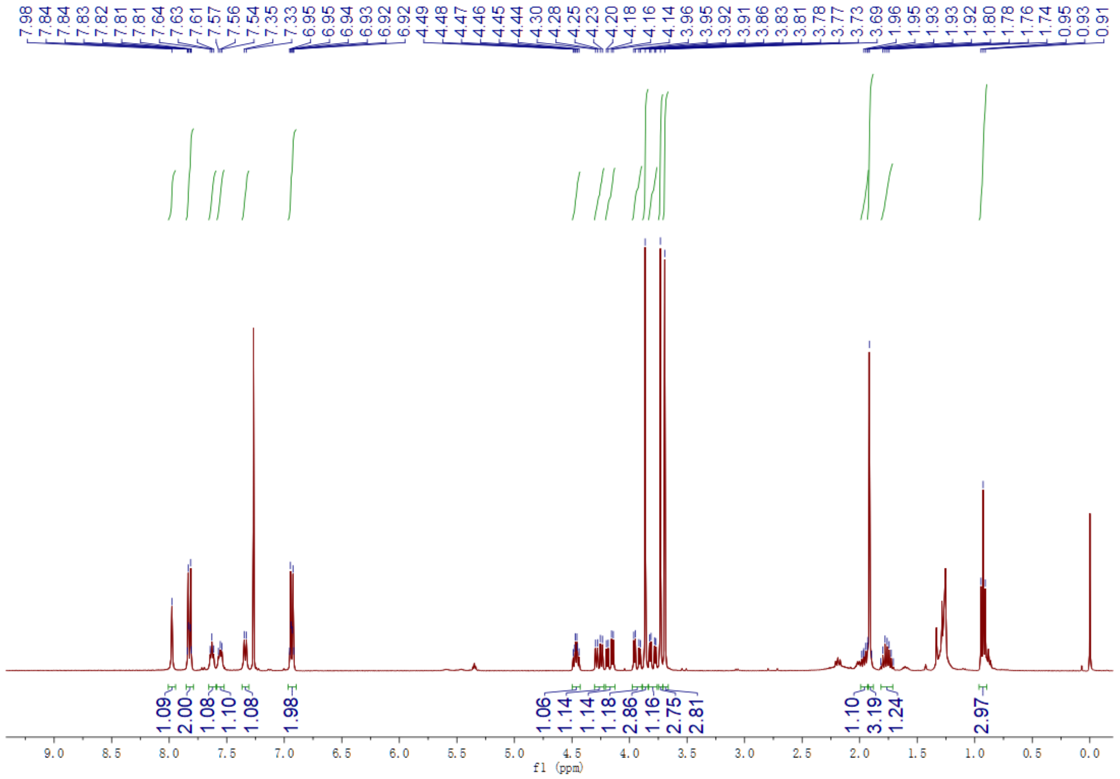
7.98 7.84 7.84 7.83 7.82 7.81 7.81 7.64 7.63 7.61 7.57 7.56 7.54 7.35 7.33 6.95 6.95 6.94 6.93 6.92 6.92 4.49 4.48 4.47 4.46 4.45 4.44 4.30 4.28 4.25 4.23 4.20 4.18 4.16 4.14 3.96 3.95 3.92 3.91 3.86 3.83 3.81 3.78 3.77 3.73 3.69 1.96 1.95 1.93 1.93 1.92 1.80 1.78 1.76 1.74 0.95 0.93 0.91
1.09 2.00 1.08 1.10 1.08 1.98 1.06 1.14 1.14 1.18 2.86 1.16 2.75 2.81 1.10 3.19 1.24 2.97
9.0 8.5 8.0 7.5 7.0 6.5 6.0 5.5 5.0 4.5 4.0 3.5 3.0 2.5 2.0 1.5 1.0 0.5 0.0
f1 (ppm)

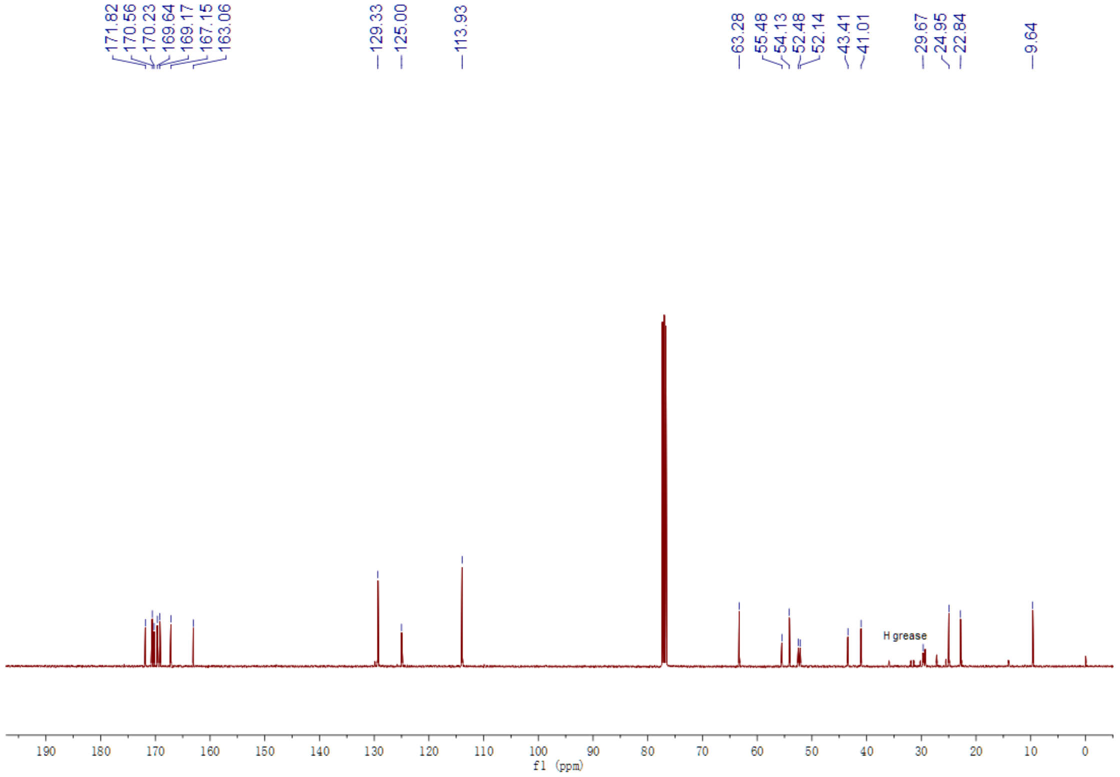
171.82 170.56 170.23 169.64 169.17 167.15 163.06 129.33 125.00 113.93 63.28 55.48 54.13 52.48 52.14 43.41 41.01 29.67 24.95 22.84 9.64
H grease
190 180 170 160 150 140 130 120 110 100 90 80 70 60 50 40 30 20 10 0
f1 (ppm)

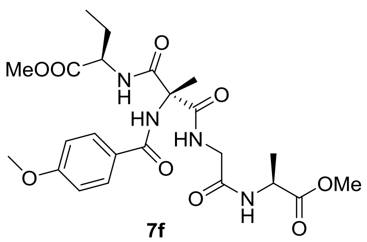
MeOOC
N
H
O
HN
O
HN
O
N
H
OMe
O
O
O

7f

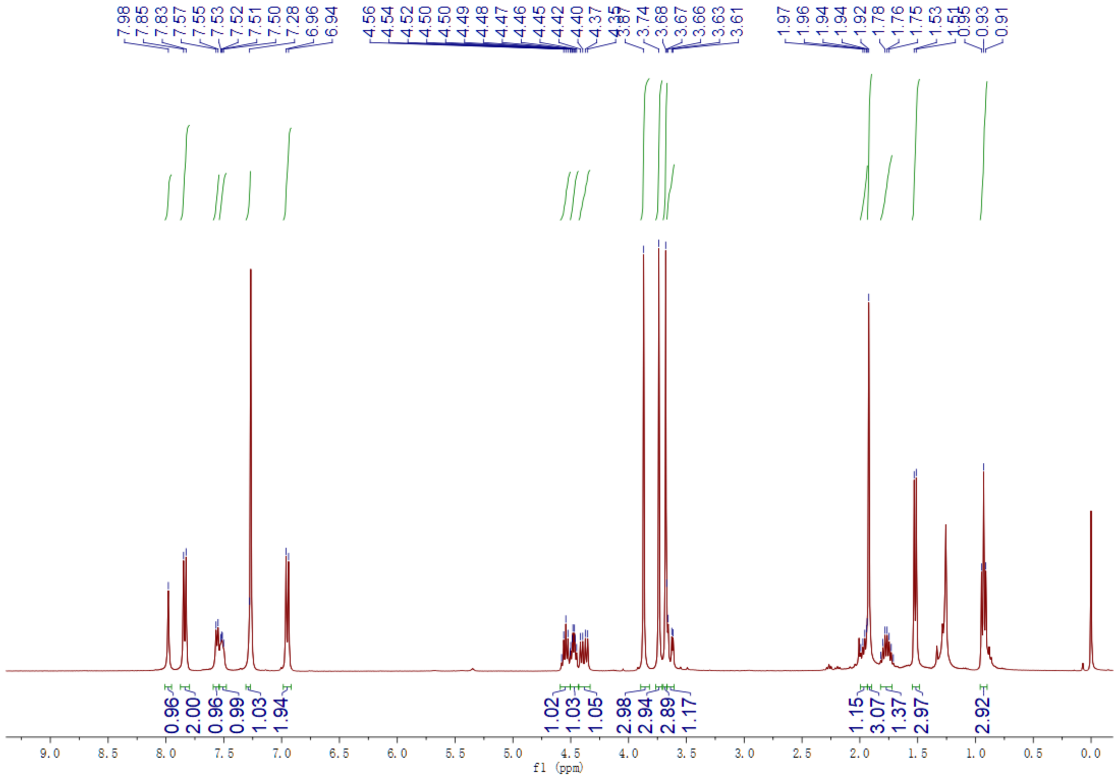
7.98
7.85
7.83
7.57
7.55
7.53
7.52
7.51
7.50
7.28
6.96
6.94
4.56
4.54
4.52
4.50
4.50
4.49
4.48
4.47
4.46
4.45
4.42
4.40
4.37
3.74
3.68
3.67
3.66
3.63
3.61
1.97
1.96
1.94
1.94
1.92
1.78
1.76
1.75
1.53
0.93
0.91
0.96
2.00
0.96
0.99
1.03
1.94
1.02
1.03
1.05
2.98
2.94
2.89
1.17
1.15
3.07
1.37
2.97
2.92
9.0
8.5
8.0
7.5
7.0
6.5
6.0
5.5
5.0
4.5
4.0
3.5
3.0
2.5
2.0
1.5
1.0
0.5
0.0
f1 (ppm)

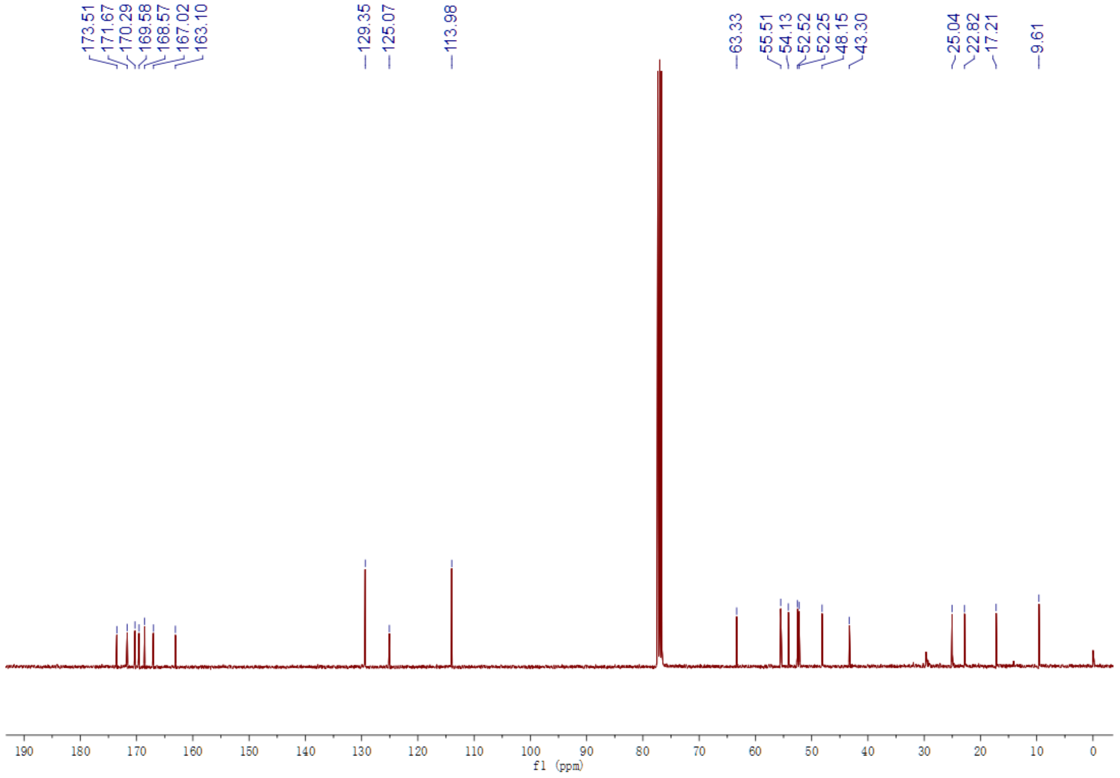
173.51
171.67
170.29
169.58
168.57
167.02
163.10
129.35
125.07
113.98
63.33
55.51
54.13
52.52
52.25
48.15
43.30
25.04
22.82
17.21
9.61
190
180
170
160
150
140
130
120
110
100
90
80
70
60
50
40
30
20
10
0
f1 (ppm)

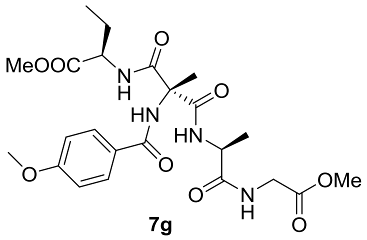
MeOOC
N
H
O
HN
O
HN
O
O
N
H
OMe
O
O

7g

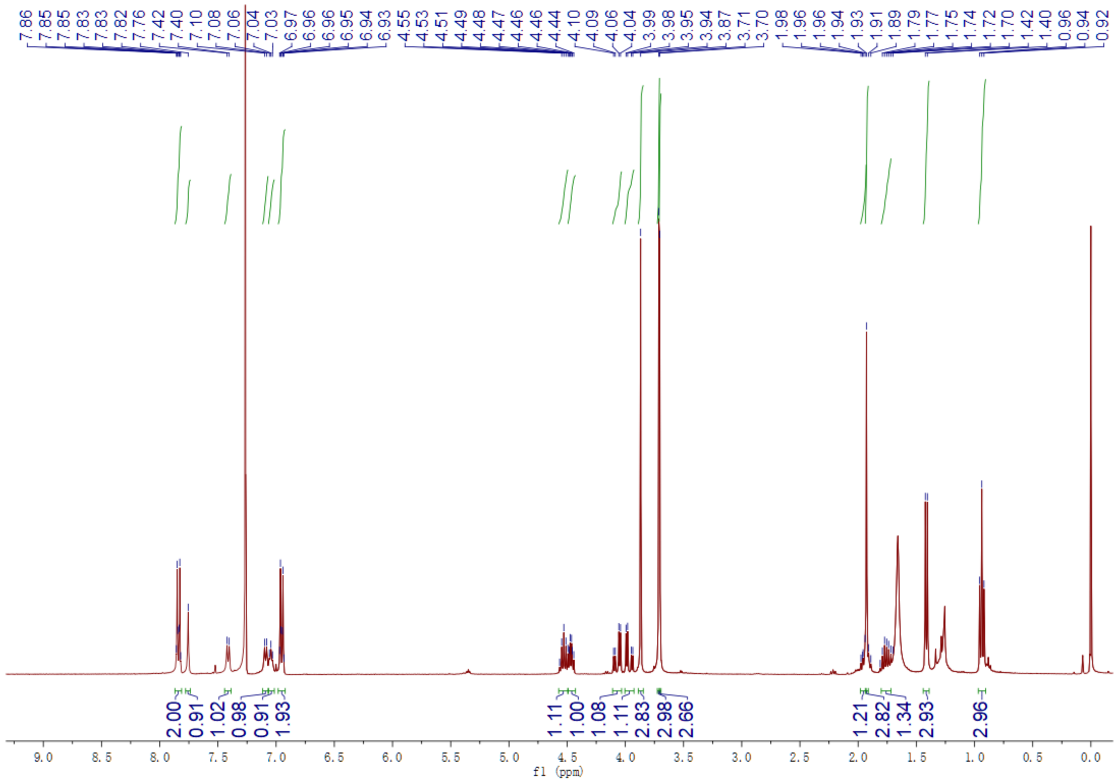
7.86
7.85
7.83
7.83
7.82
7.76
7.42
7.40
7.10
7.08
7.06
7.04
7.03
6.97
6.96
6.96
6.95
6.94
6.93
4.55
4.53
4.51
4.49
4.48
4.47
4.46
4.46
4.44
4.10
4.09
4.06
4.04
3.99
3.98
3.95
3.94
3.87
3.71
3.70
1.98
1.96
1.94
1.93
1.91
1.89
1.79
1.77
1.75
1.74
1.72
1.70
1.42
1.40
0.96
0.94
0.92
2.00
0.91
1.02
0.98
0.91
1.93
1.11
1.00
1.08
1.11
2.83
2.98
2.66
1.21
2.82
1.34
2.93
2.96
f1 (ppm)

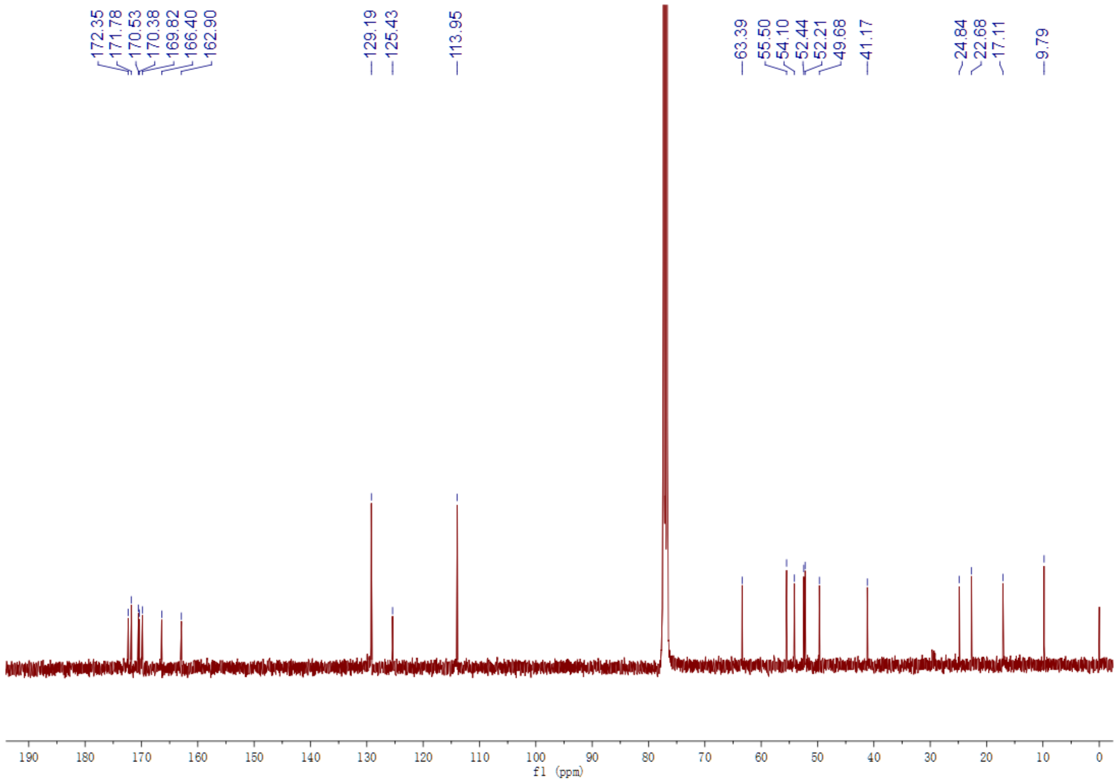
172.35
171.78
170.53
170.38
169.82
166.40
162.90
129.19
125.43
113.95
63.39
55.50
54.10
52.44
52.21
49.68
41.17
24.84
22.68
17.11
9.79
f1 (ppm)

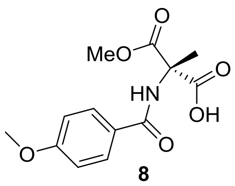

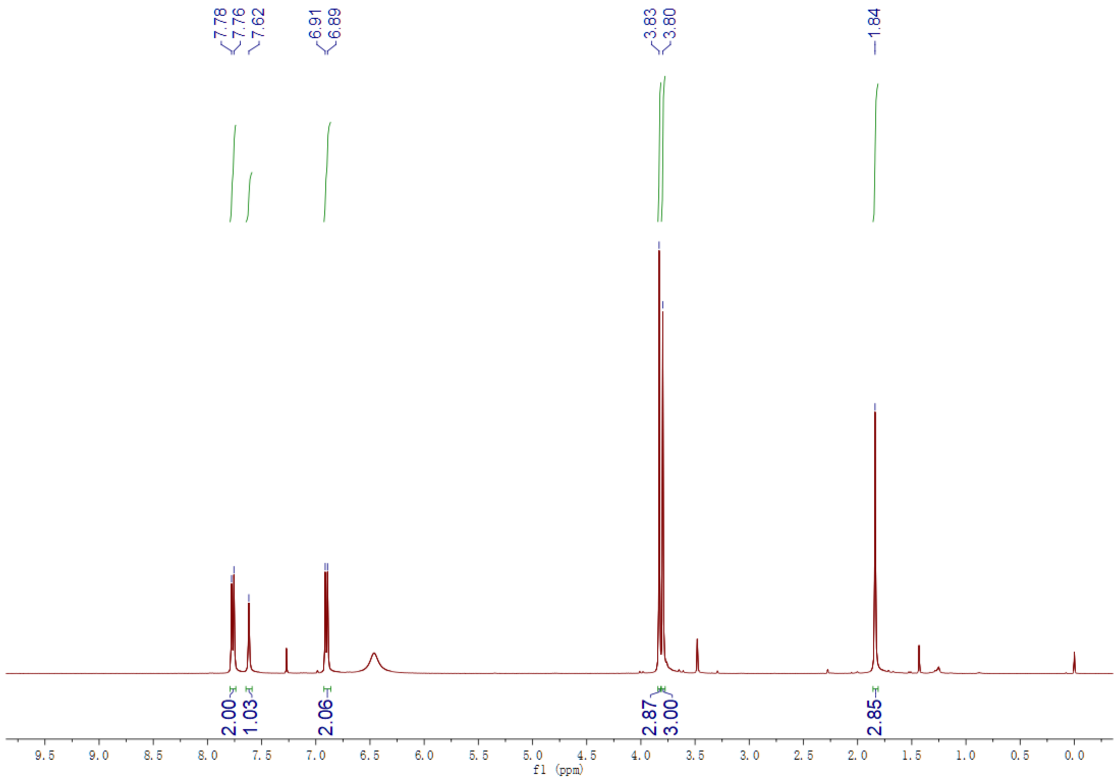
7.78
7.76
7.62
6.91
6.89
3.83
3.80
1.84
2.00
1.03
2.06
2.87
3.00
2.85
f1 (ppm)

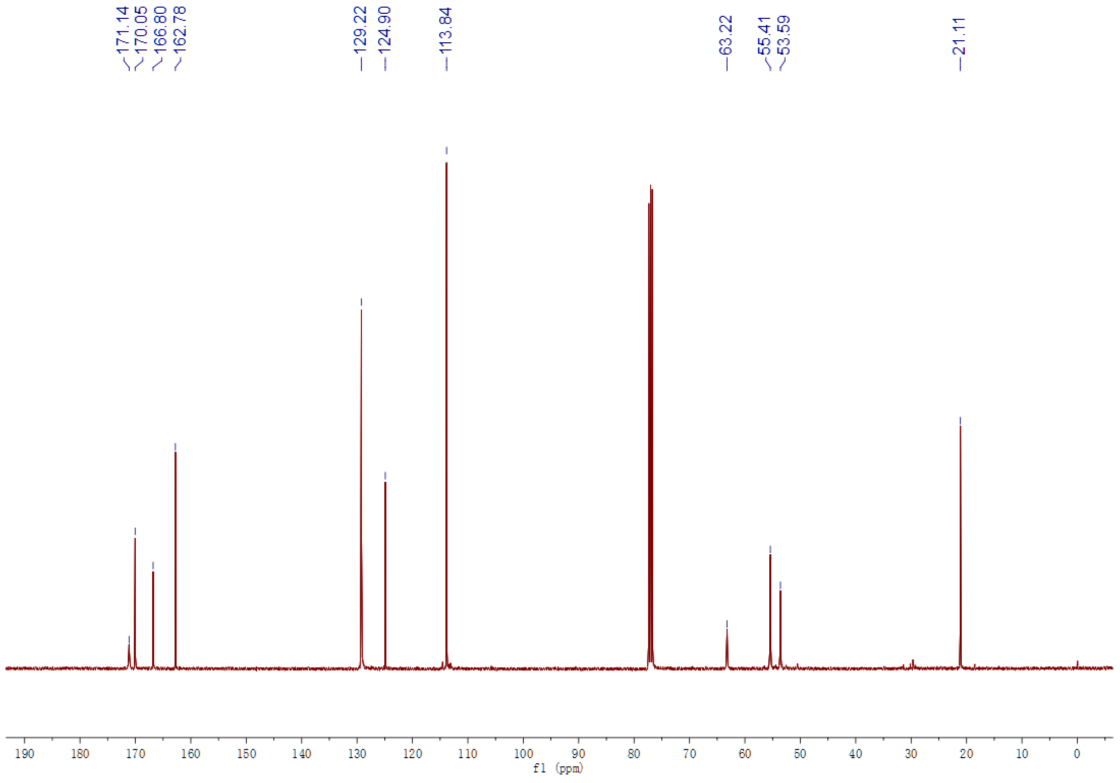
171.14
170.05
166.80
162.78
129.22
124.90
113.84
63.22
55.41
53.59
21.11
f1 (ppm)

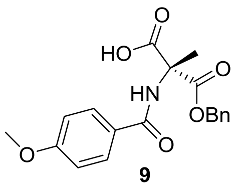
HO
O
HN
O
OBn
O
O

9

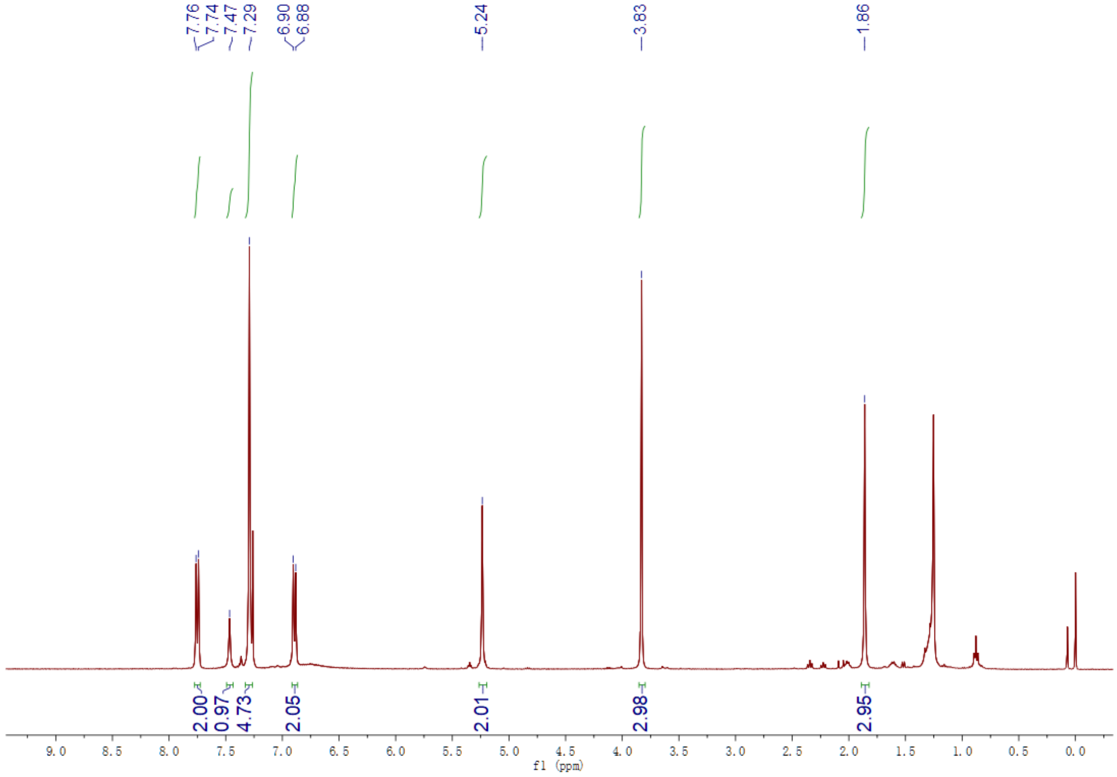
7.76
7.74
7.47
7.29
6.90
6.88
5.24
3.83
1.86
2.00
0.97
4.73
2.05
2.01
2.98
2.95
9.0
8.5
8.0
7.5
7.0
6.5
6.0
5.5
5.0
4.5
4.0
3.5
3.0
2.5
2.0
1.5
1.0
0.5
0.0
f1 (ppm)

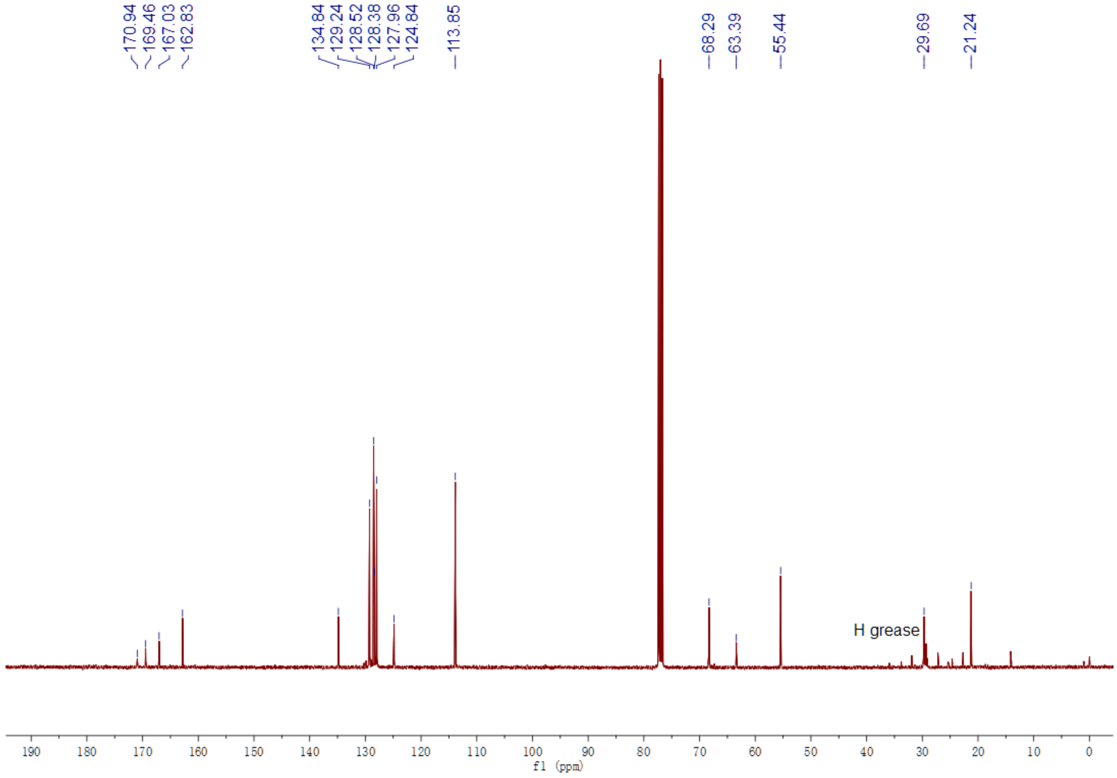
170.94
169.46
167.03
162.83
134.84
129.24
128.52
128.38
127.96
124.84
113.85
68.29
63.39
55.44
29.69
21.24
H grease
190
180
170
160
150
140
130
120
110
100
90
80
70
60
50
40
30
20
10
0
f1 (ppm)

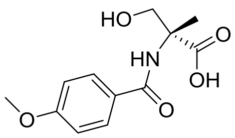
HO
HN
O
OH
O
O

(*R*)-10

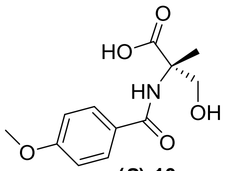
O
HO
HN
OH
O
O

(*S*)-10

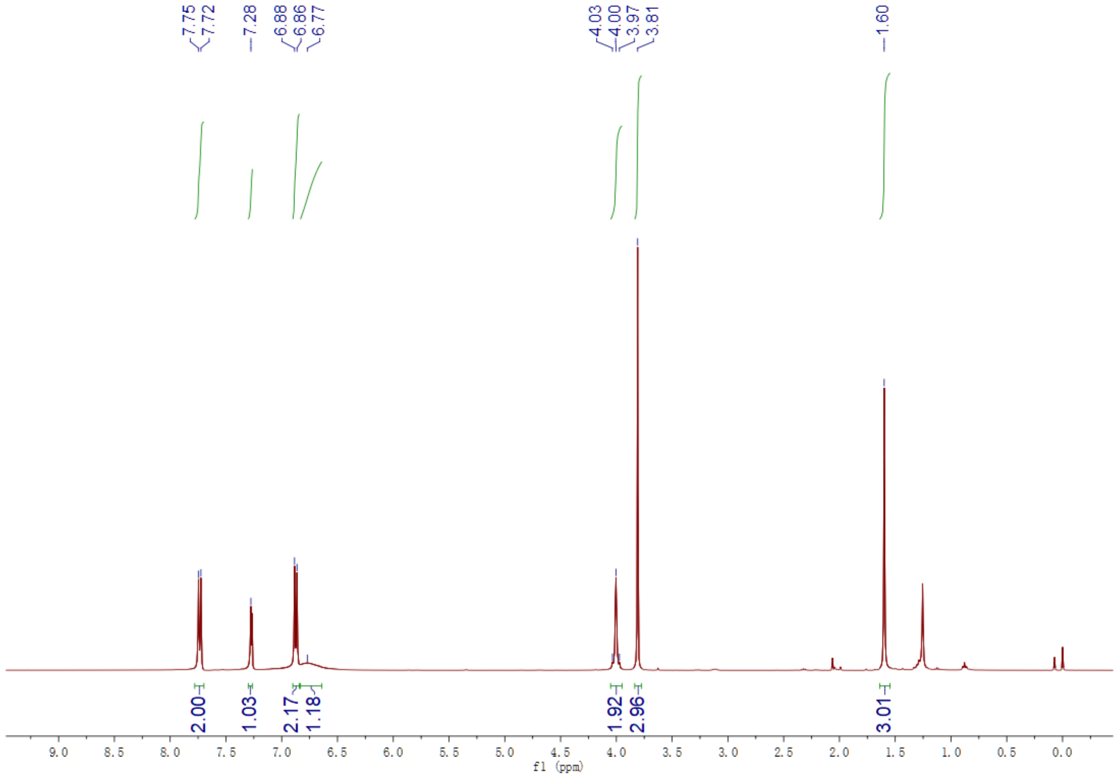
7.75
7.72
7.28
6.88
6.86
6.77
4.03
4.00
3.97
3.81
1.60
2.00
1.03
2.17
1.18
1.92
2.96
3.01
9.0 8.5 8.0 7.5 7.0 6.5 6.0 5.5 5.0 4.5 4.0 3.5 3.0 2.5 2.0 1.5 1.0 0.5 0.0
f1 (ppm)

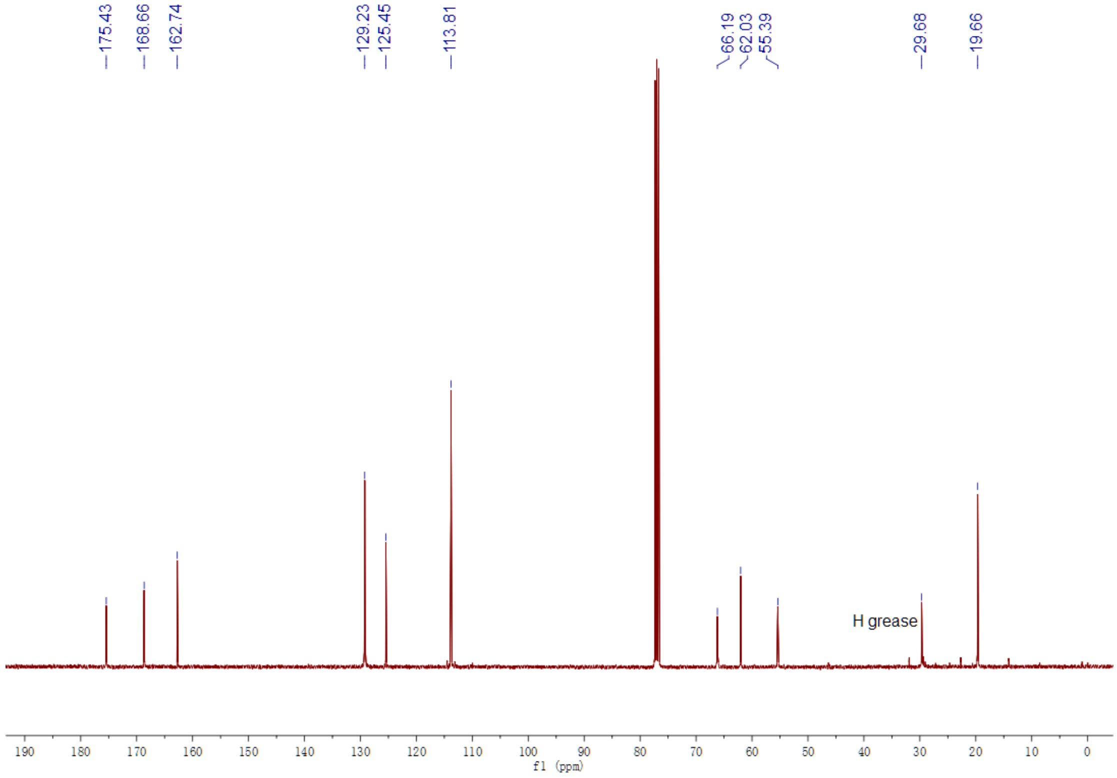
175.43
168.66
162.74
129.23
125.45
113.81
66.19
62.03
55.39
29.68
19.66
H grease
190 180 170 160 150 140 130 120 110 100 90 80 70 60 50 40 30 20 10 0
f1 (ppm)

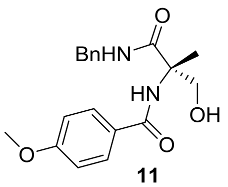
O
BnHN
HN
OH
O
O

11

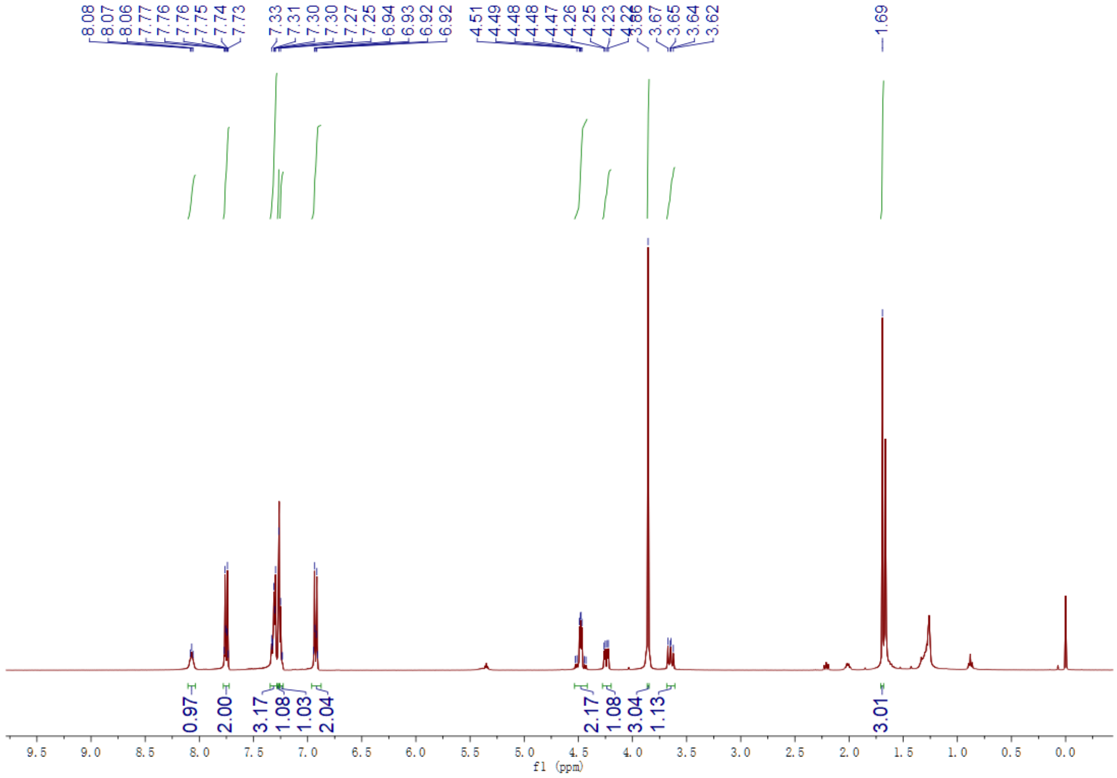
8.08
8.07
8.06
7.77
7.76
7.76
7.75
7.74
7.73
7.33
7.31
7.30
7.27
7.25
6.94
6.93
6.92
6.92
4.51
4.49
4.48
4.48
4.47
4.26
4.25
4.23
4.22
3.86
3.67
3.65
3.64
3.62
1.69
0.97
2.00
3.17
1.08
1.03
2.04
2.17
1.08
3.04
1.13
3.01
9.5
9.0
8.5
8.0
7.5
7.0
6.5
6.0
5.5
5.0
4.5
4.0
3.5
3.0
2.5
2.0
1.5
1.0
0.5
0.0
f1 (ppm)

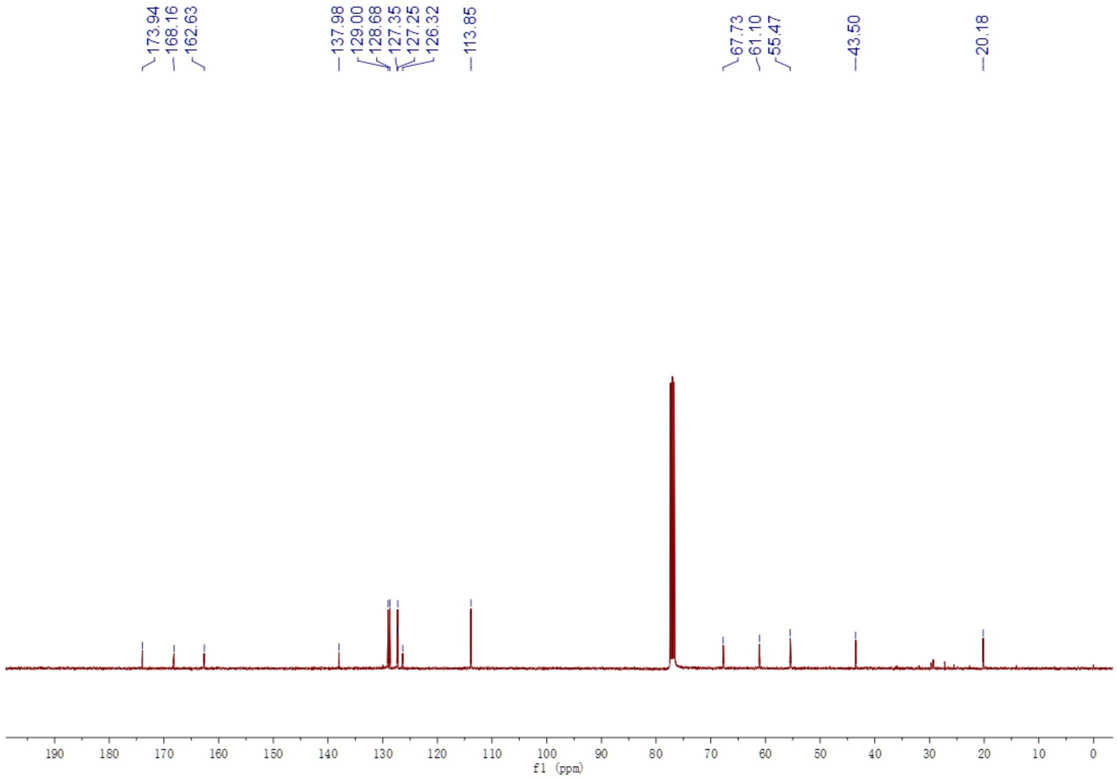
173.94
168.16
162.63
137.98
129.00
128.68
127.35
127.25
126.32
113.85
67.73
61.10
55.47
43.50
20.18
190
180
170
160
150
140
130
120
110
100
90
80
70
60
50
40
30
20
10
0
f1 (ppm)

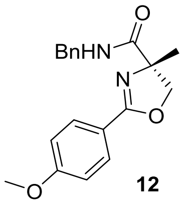
BnHN
O
N
O

12

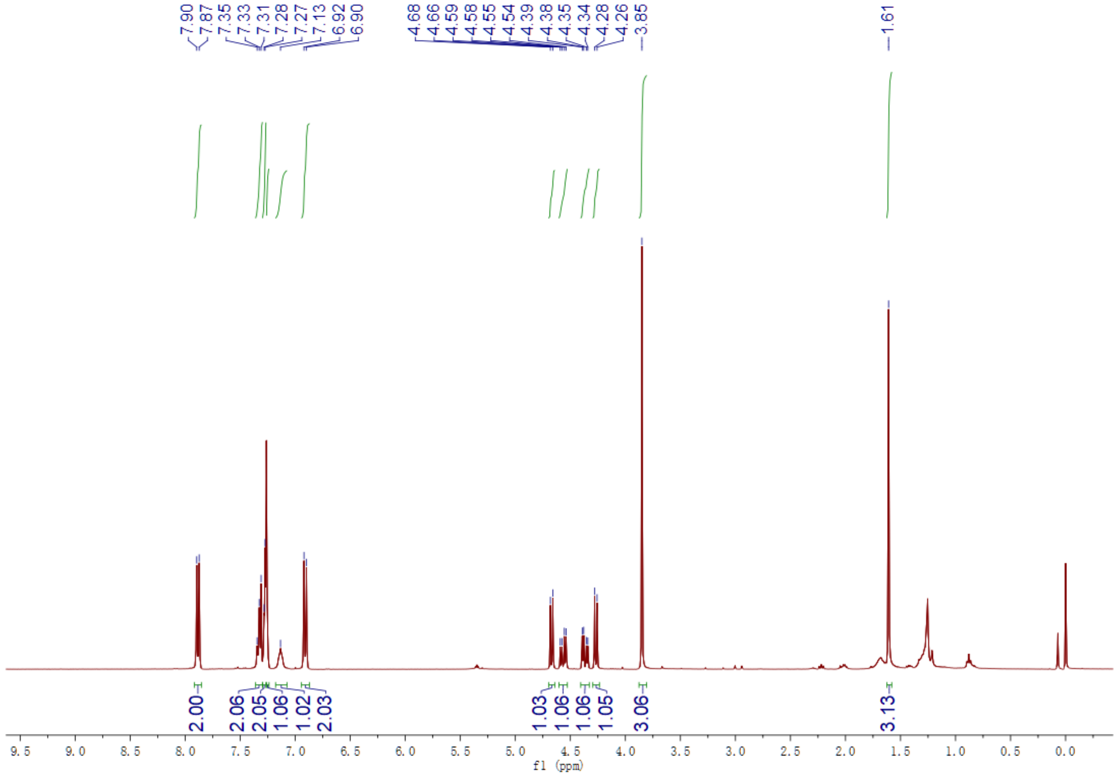
7.90
7.87
7.35
7.33
7.31
7.28
7.27
7.13
6.92
6.90
4.68
4.66
4.59
4.58
4.55
4.54
4.39
4.38
4.35
4.34
4.28
4.26
3.85
1.61
2.00
2.06
2.05
1.06
1.02
2.03
1.03
1.06
1.06
1.05
3.06
3.13
9.5 9.0 8.5 8.0 7.5 7.0 6.5 6.0 5.5 5.0 4.5 4.0 3.5 3.0 2.5 2.0 1.5 1.0 0.5 0.0
f1 (ppm)

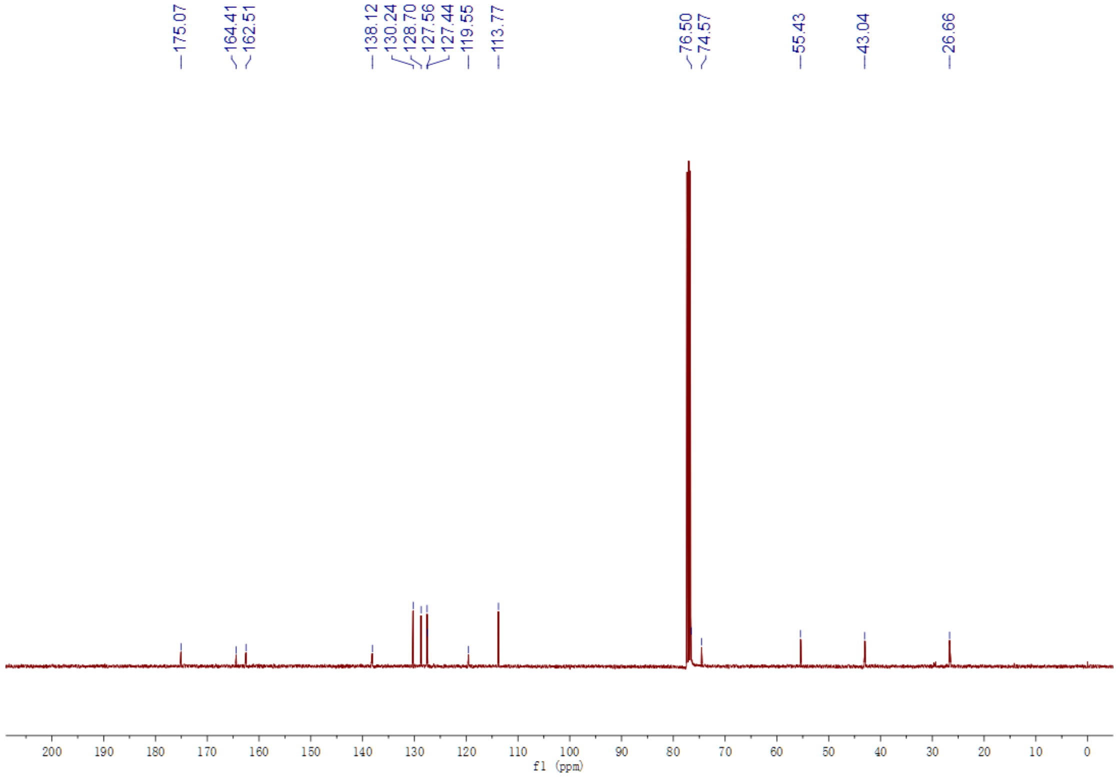
175.07
164.41
162.51
138.12
130.24
128.70
127.56
127.44
119.55
113.77
76.50
74.57
55.43
43.04
26.66
200 190 180 170 160 150 140 130 120 110 100 90 80 70 60 50 40 30 20 10 0
f1 (ppm)

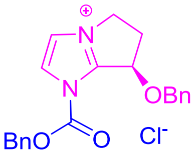
N
N
OBn
BnO
O
Cl-

A

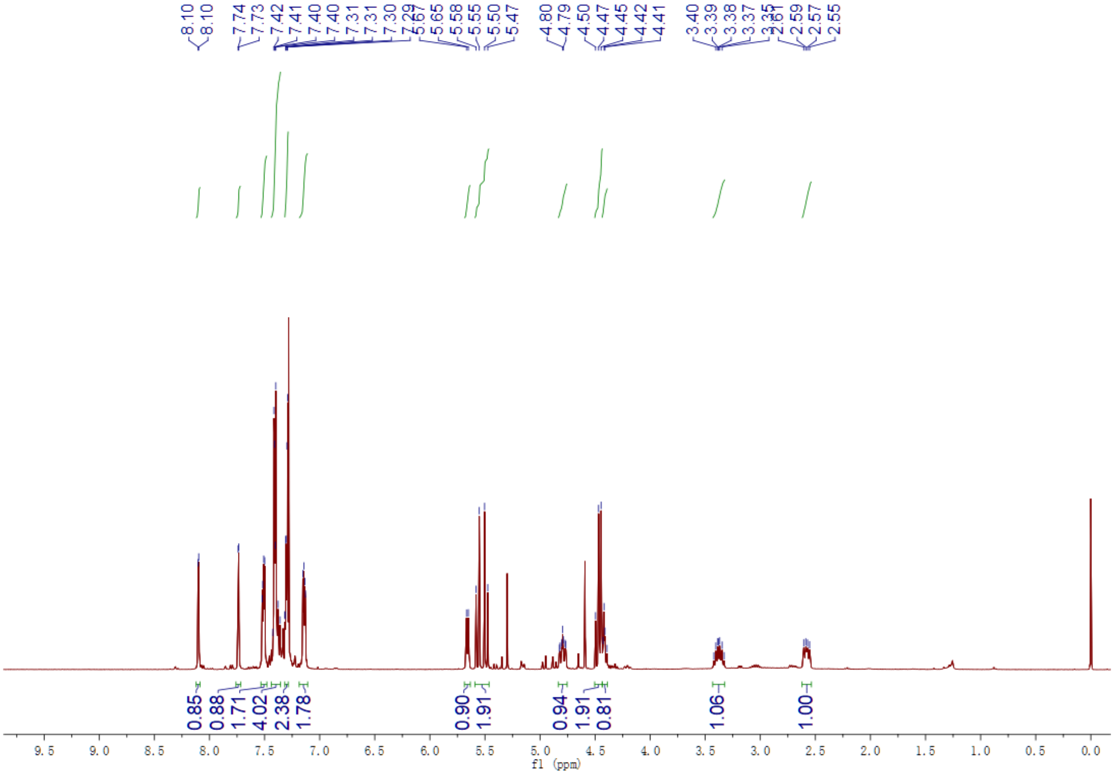
8.10
8.10
7.74
7.73
7.42
7.41
7.40
7.40
7.31
7.31
7.30
7.29
5.67
5.65
5.58
5.55
5.50
5.47
4.80
4.79
4.50
4.47
4.45
4.42
4.41
3.40
3.39
3.38
3.37
3.35
2.61
2.59
2.57
2.55
0.85
0.88
1.71
4.02
2.38
1.78
0.90
1.91
0.94
1.91
0.81
1.06
1.00
9.5 9.0 8.5 8.0 7.5 7.0 6.5 6.0 5.5 5.0 4.5 4.0 3.5 3.0 2.5 2.0 1.5 1.0 0.5 0.0
f1 (ppm)

10. HPLC Charts

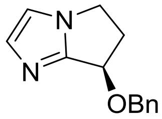

(*R*)-**OBn-DPI**

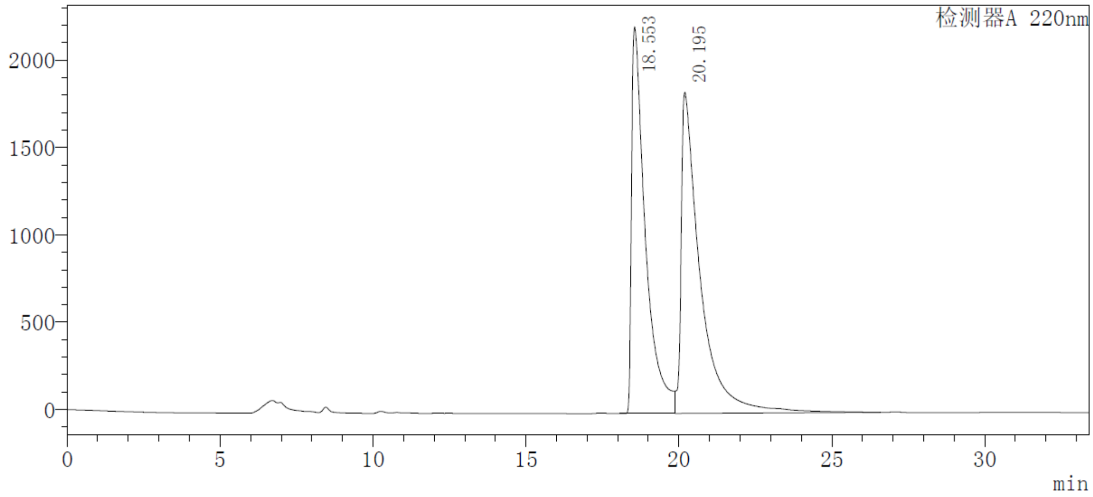

Peak	Ret. Time	Area %
1	18.553	46.721
2	20.195	53.279

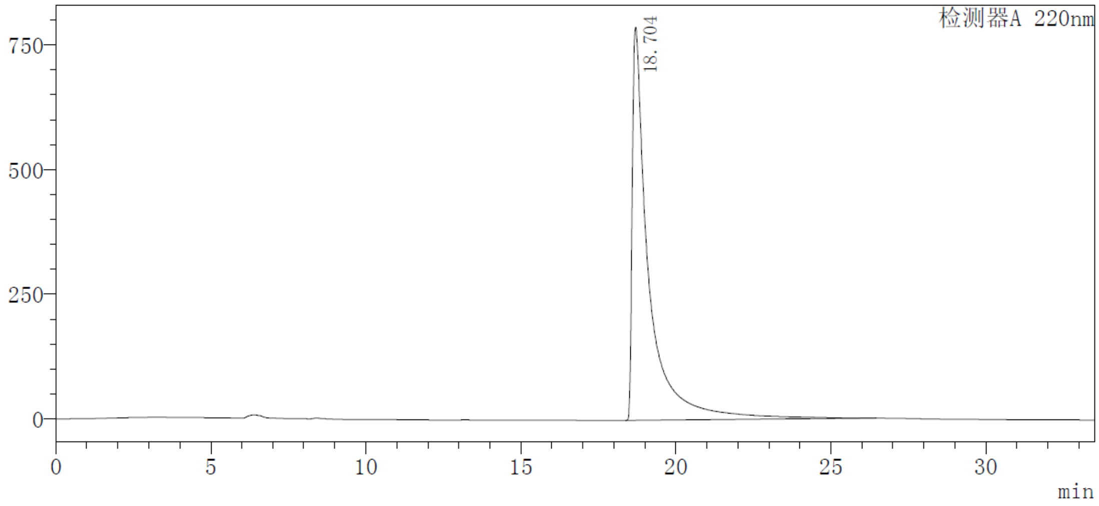

Peak	Ret. Time	Area %	Ee
1	18.704	100.000	100

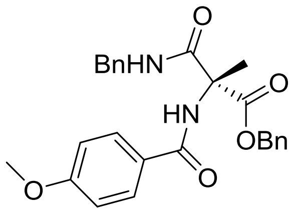

2a

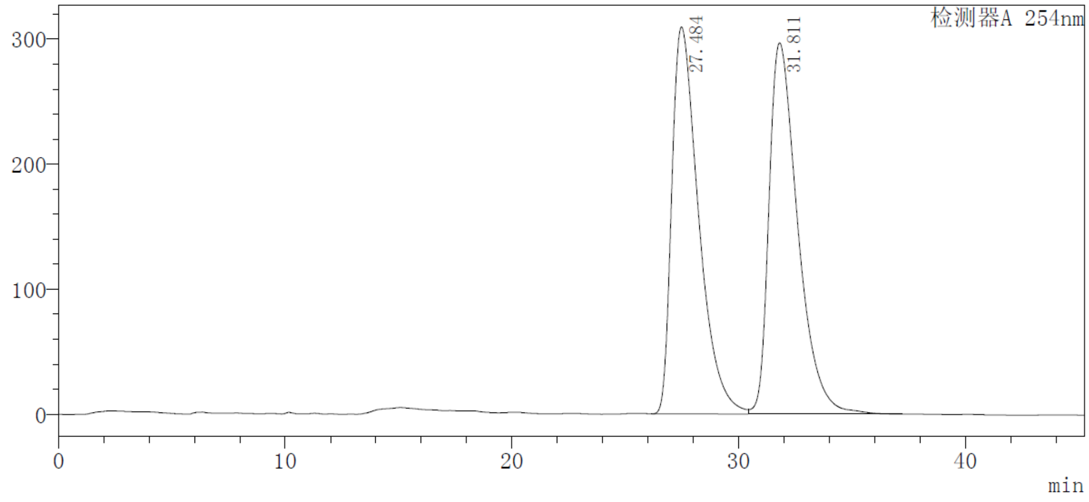

Peak	Ret. Time	Area %
1	27.484	49.318
2	31.811	50.682

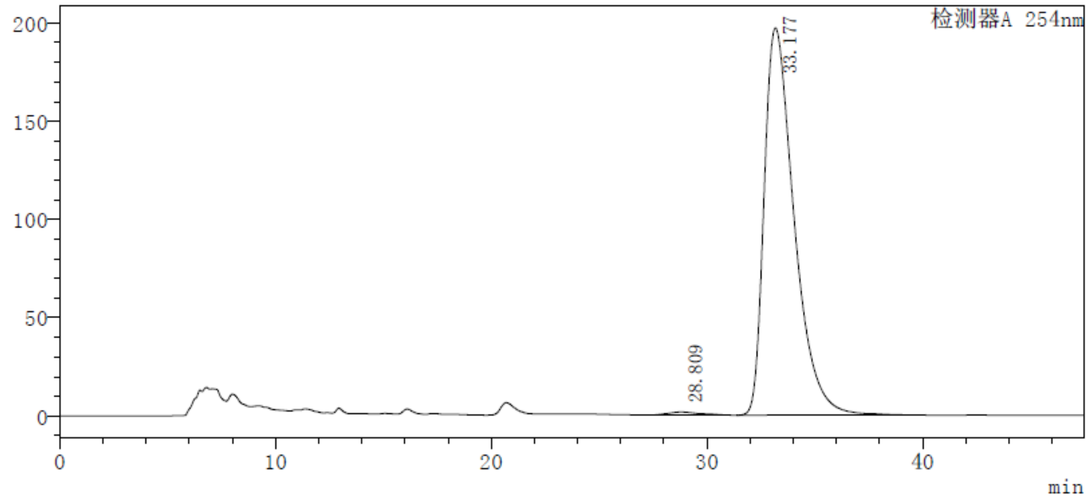

Peak	Ret. Time	Area %	Ee
1	28.809	0.719	
2	33.177	99.281	99

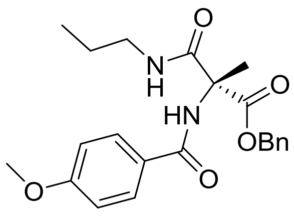

2b

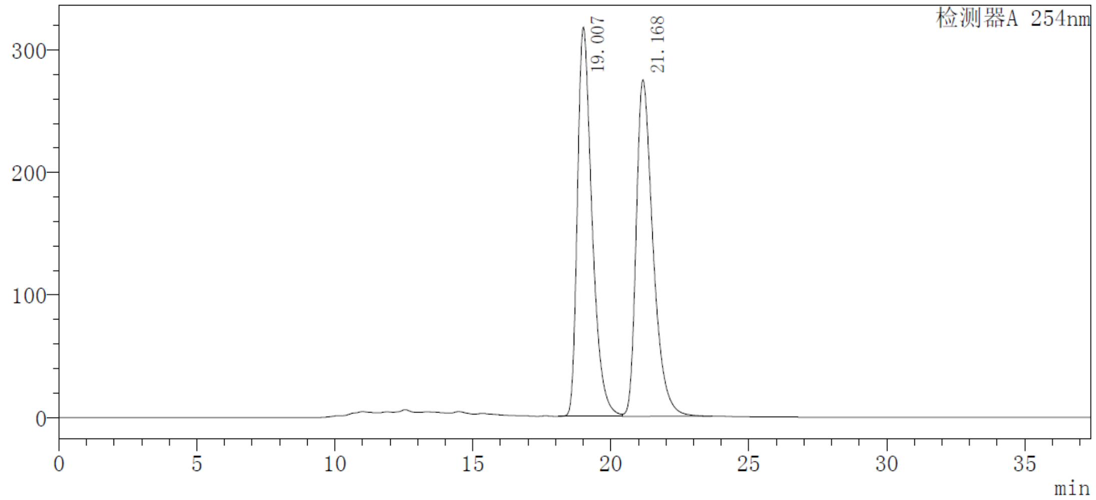

Peak	Ret. Time	Area %
1	19.007	50.033
2	21.168	49.967

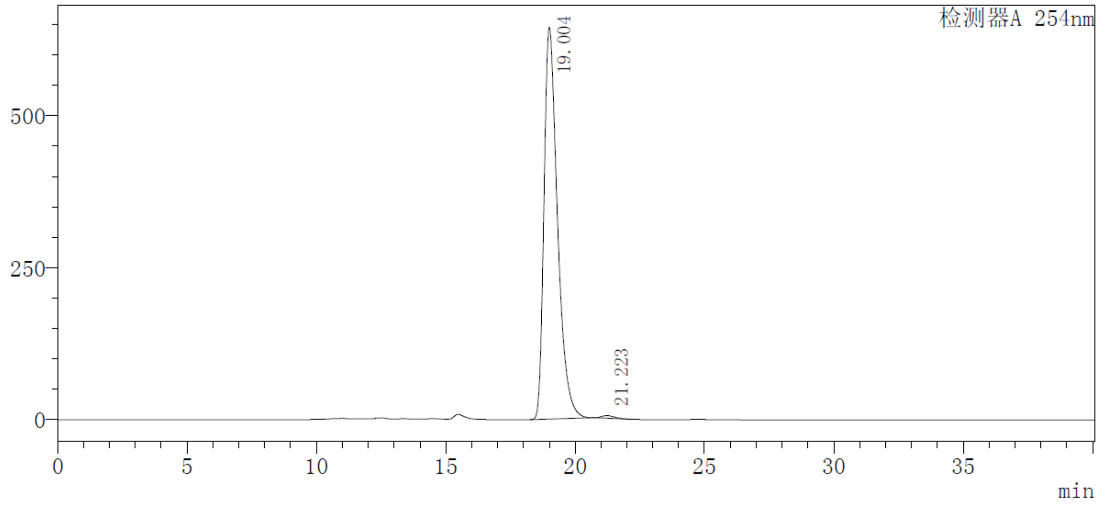

Peak	Ret. Time	Area %	Ee
1	19.004	99.334	99
2	21.223	0.666	

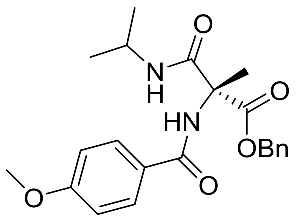

2c

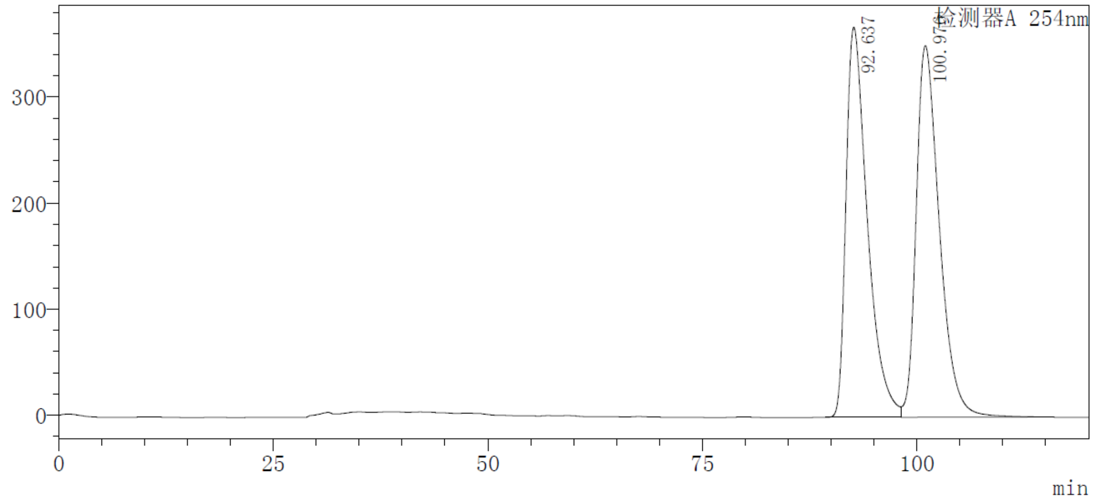

Peak	Ret. Time	Area %
1	92.637	49.171
2	100.976	50.829

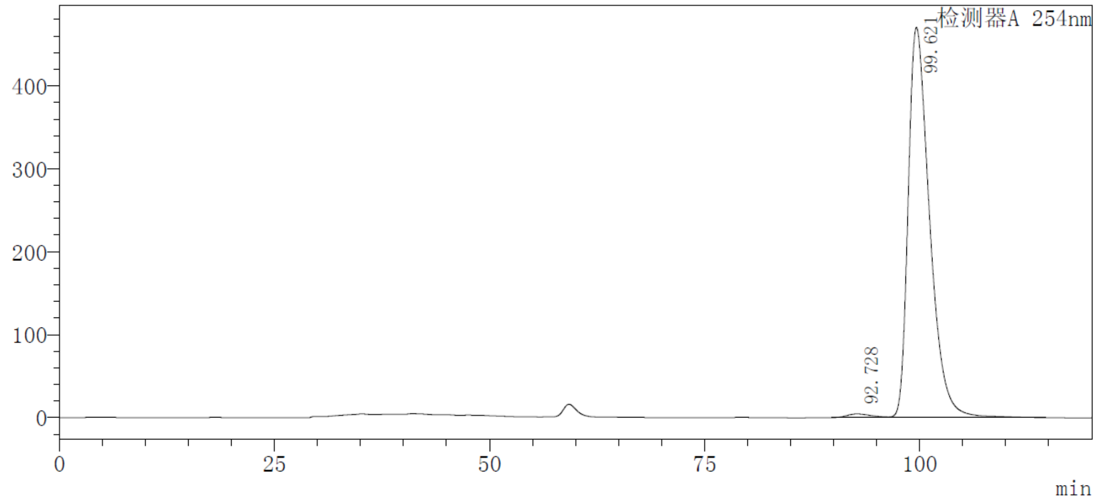

Peak	Ret. Time	Area %	Ee
1	92.728	1.078	
2	99.621	98.922	98

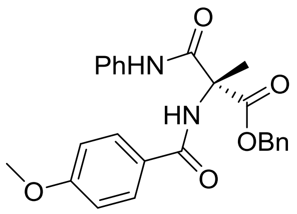

2d

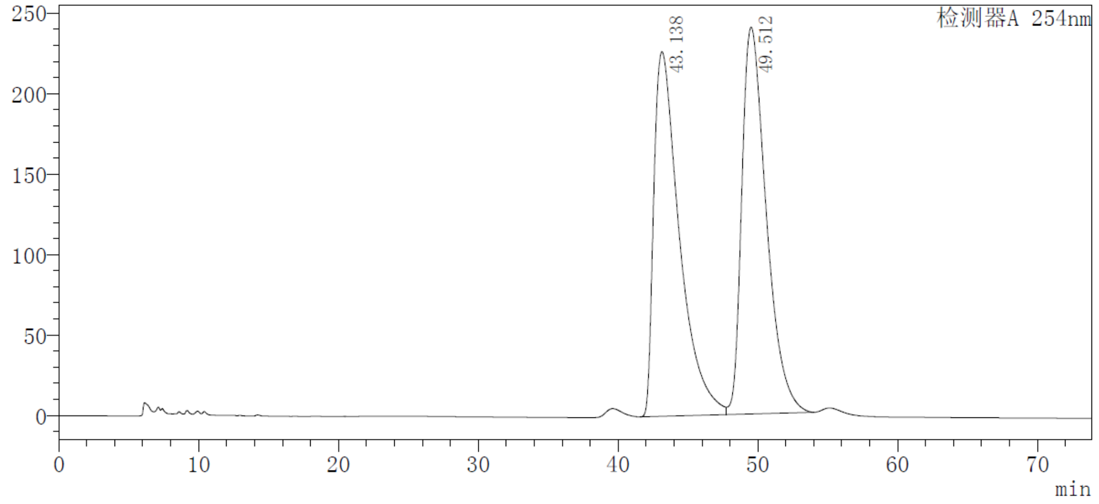

Peak	Ret. Time	Area %
1	43.138	49.685
2	49.512	50.315

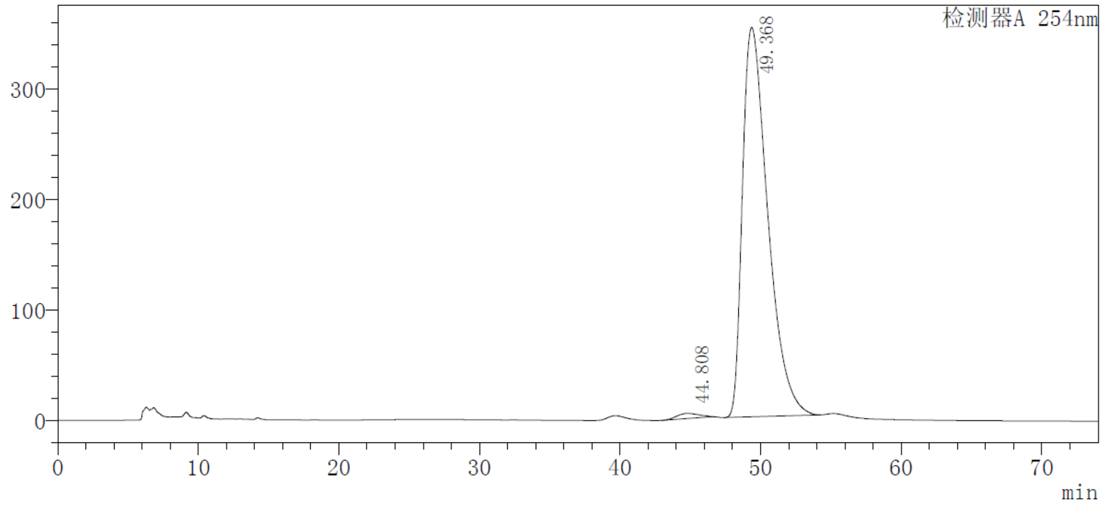

Peak	Ret. Time	Area %	Ee
1	44.808	1.163	
2	49.368	98.837	98

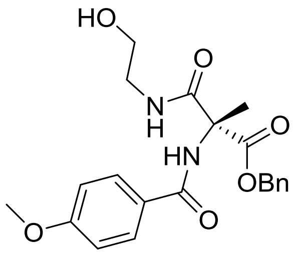

2e

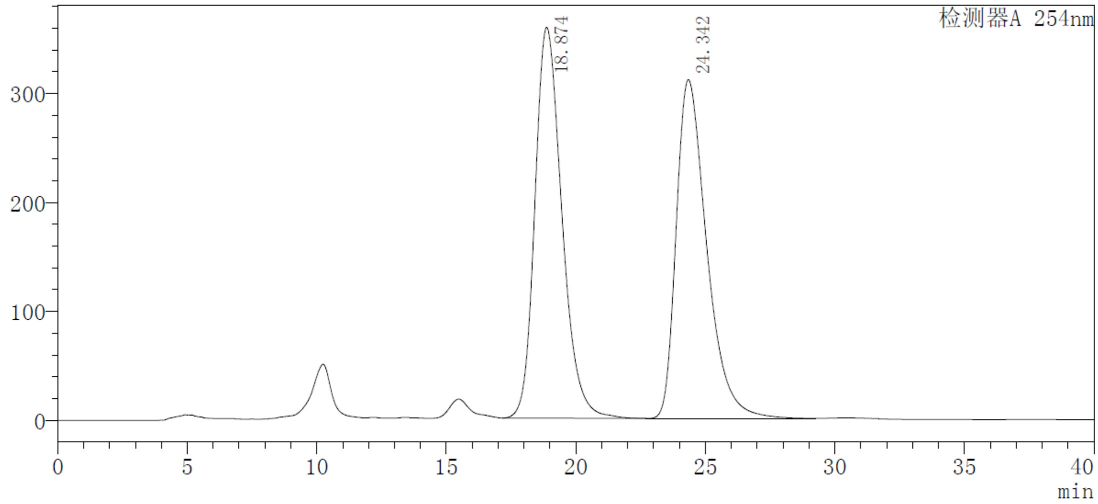

Peak	Ret. Time	Area %
1	18.874	50.587
2	24.342	49.413

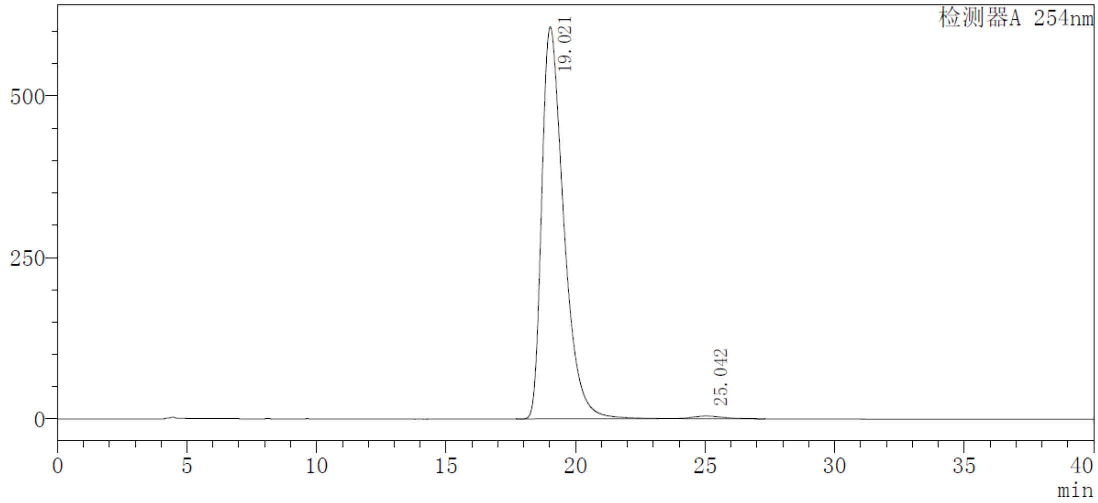

Peak	Ret. Time	Area %	Ee
1	19.021	99.127	98
2	25.042	0.873	

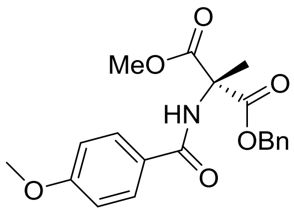

2f

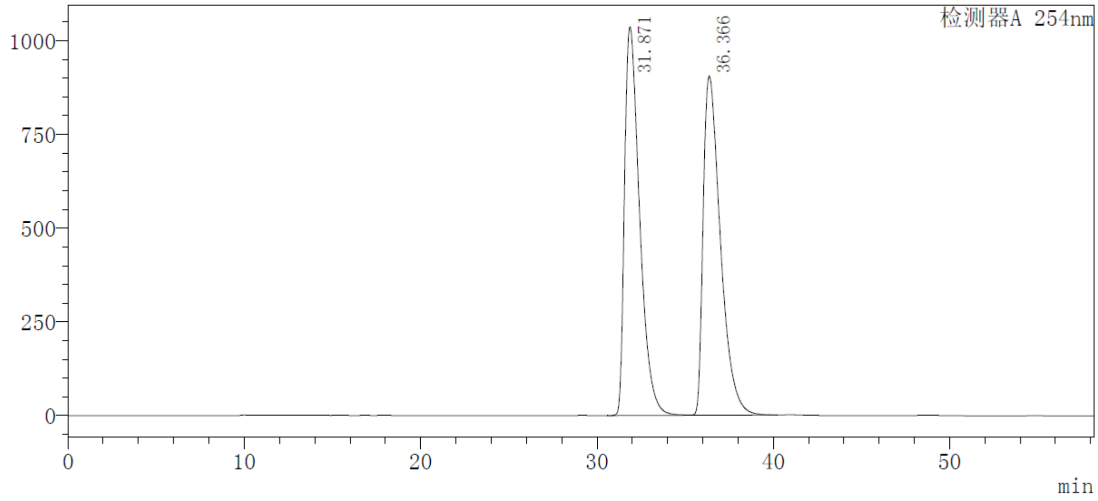

Peak	Ret. Time	Area %
1	31.871	50.030
2	36.366	49.970

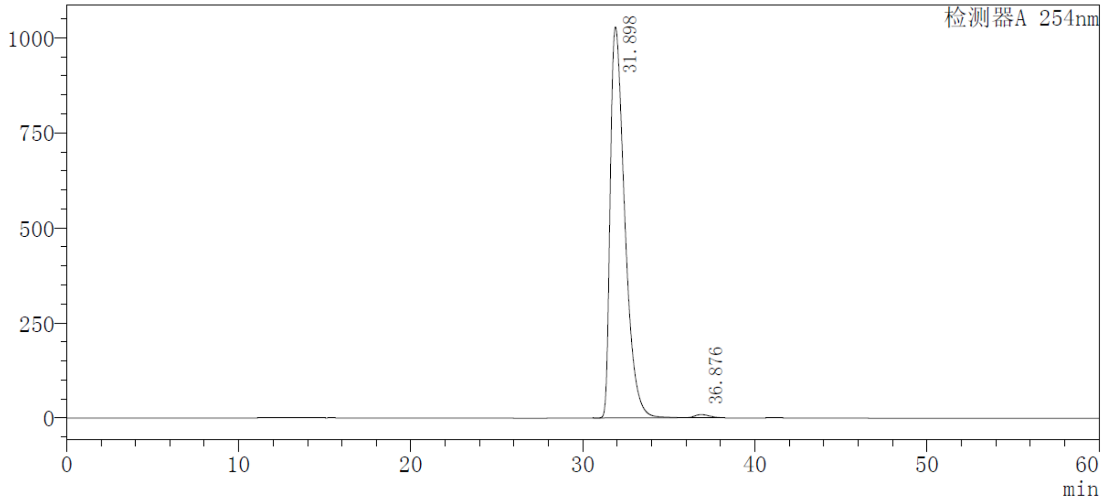

Peak	Ret. Time	Area %	Ee
1	31.898	99.230	98
2	36.876	0.770	

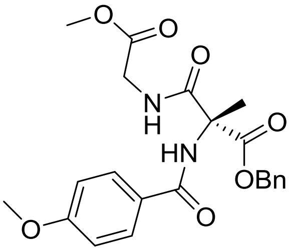

2g

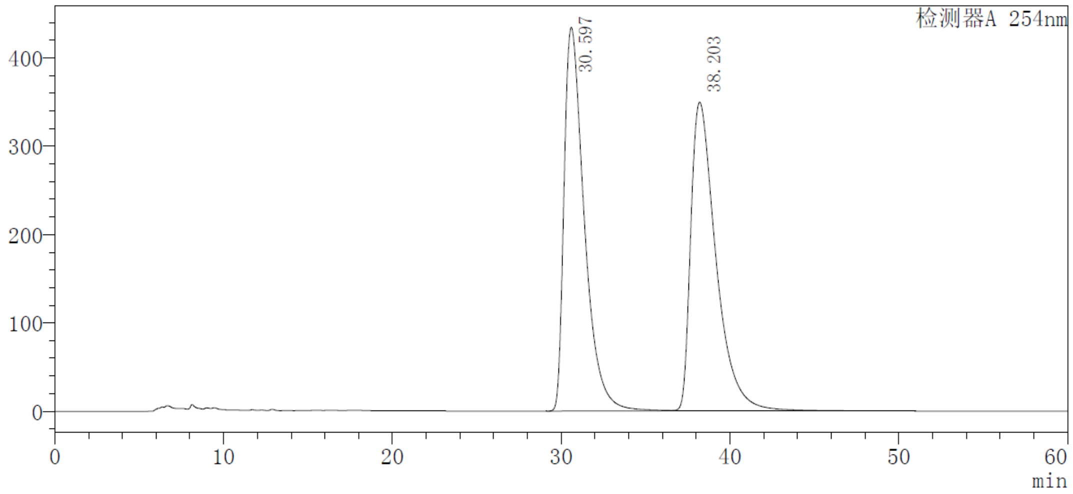

Peak	Ret. Time	Area %
1	30.597	49.979
2	38.203	50.021

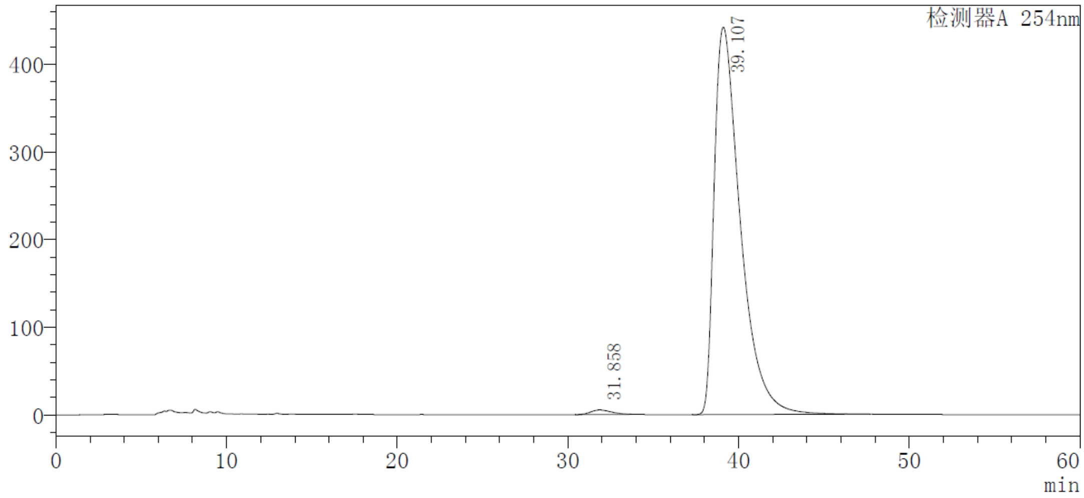

Peak	Ret. Time	Area %	Ee
1	31.858	0.923	
2	39.107	99.077	98

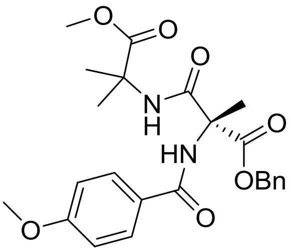

2h

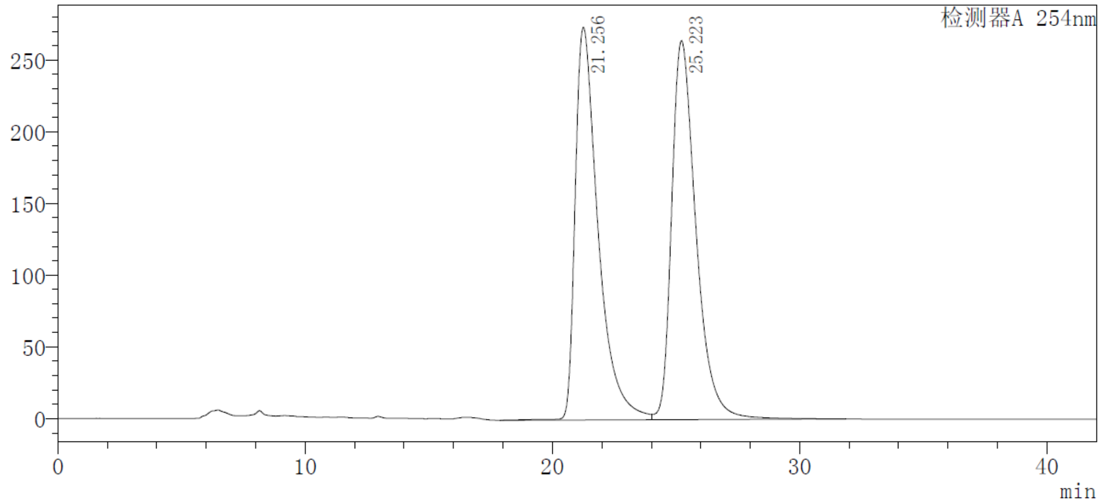

Peak	Ret. Time	Area %
1	21.256	49.374
2	25.223	50.626

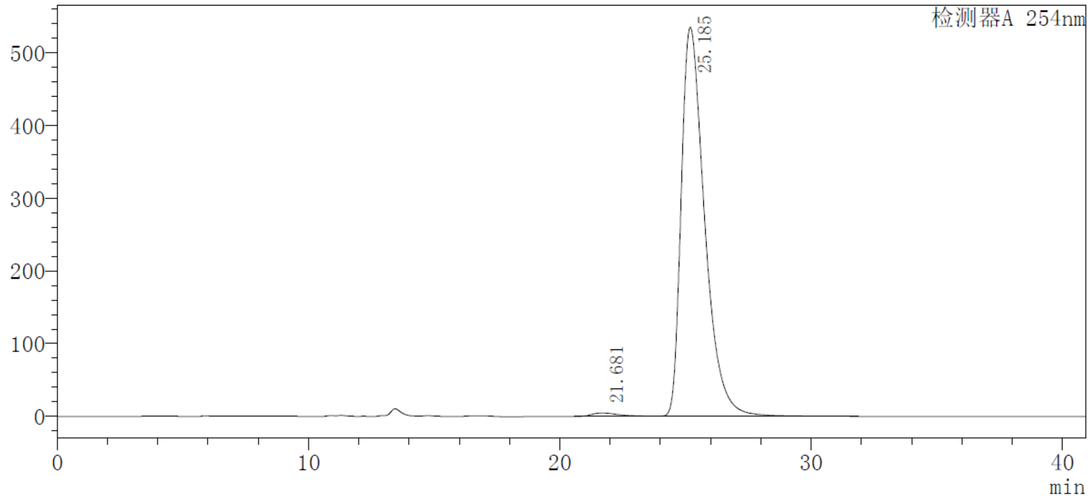

Peak	Ret. Time	Area %	Ee
1	21.681	1.030	
2	25.185	98.970	98

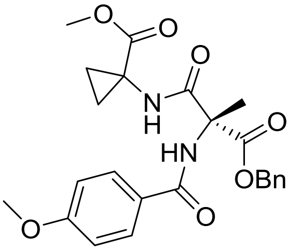

2i

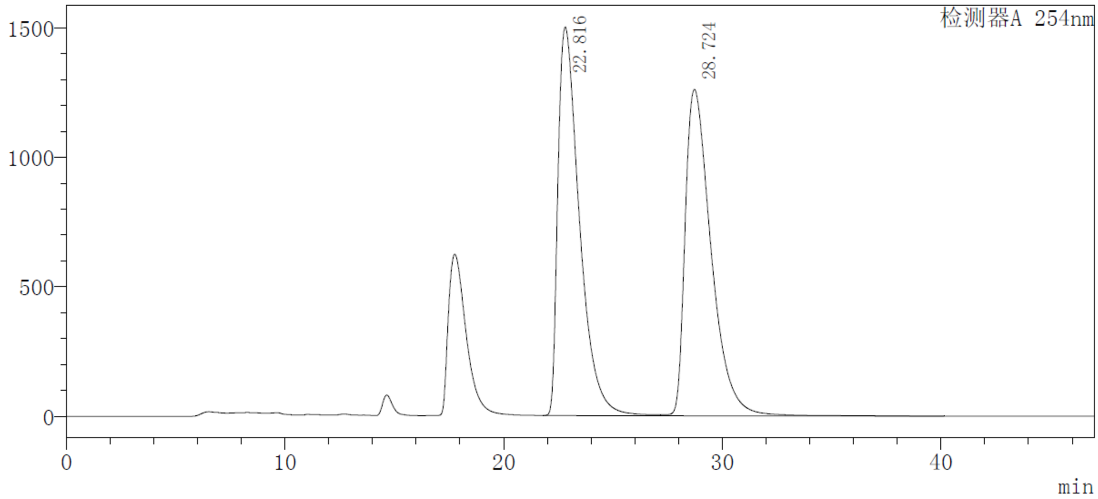

Peak	Ret. Time	Area %
1	22.816	49.643
2	28.724	50.357

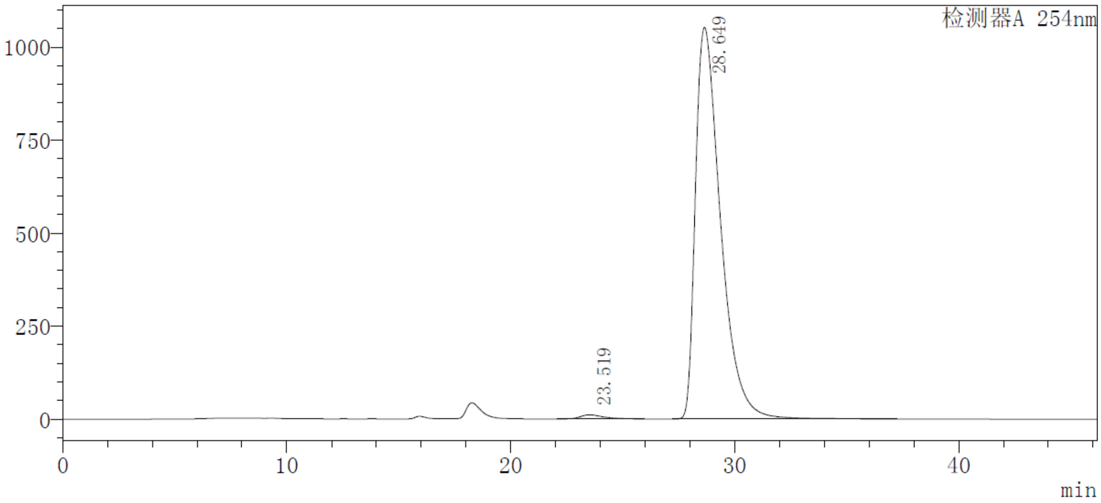

Peak	Ret. Time	Area %	Ee
1	23.519	0.959	
2	28.649	99.041	98

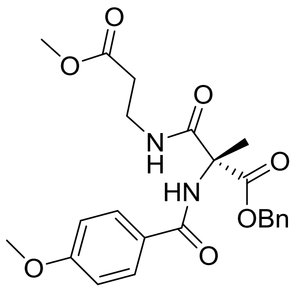

2j

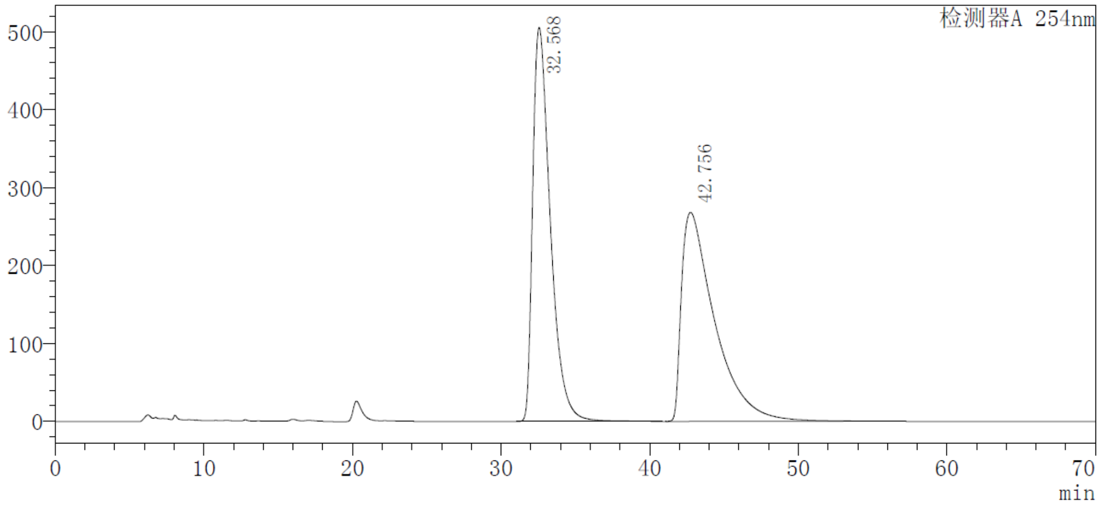

Peak	Ret. Time	Area %
1	32.568	50.129
2	42.756	49.871

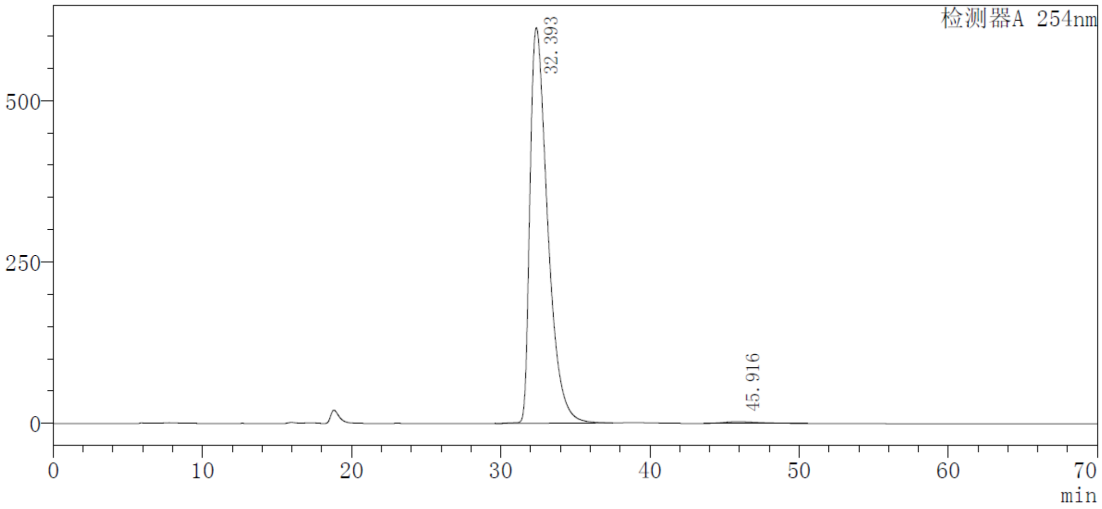

Peak	Ret. Time	Area %	Ee
1	32.393	99.110	98
2	45.916	0.890	

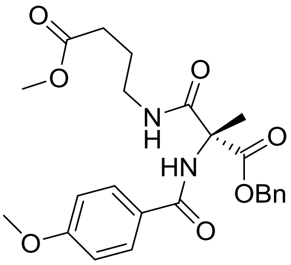

2k

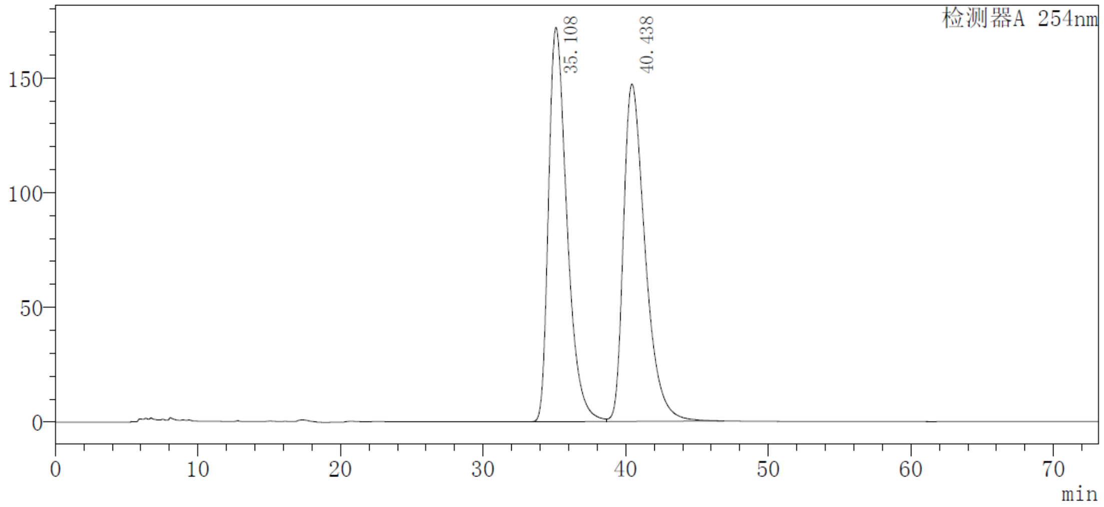

Peak	Ret. Time	Area %
1	35.108	49.799
2	40.438	50.201

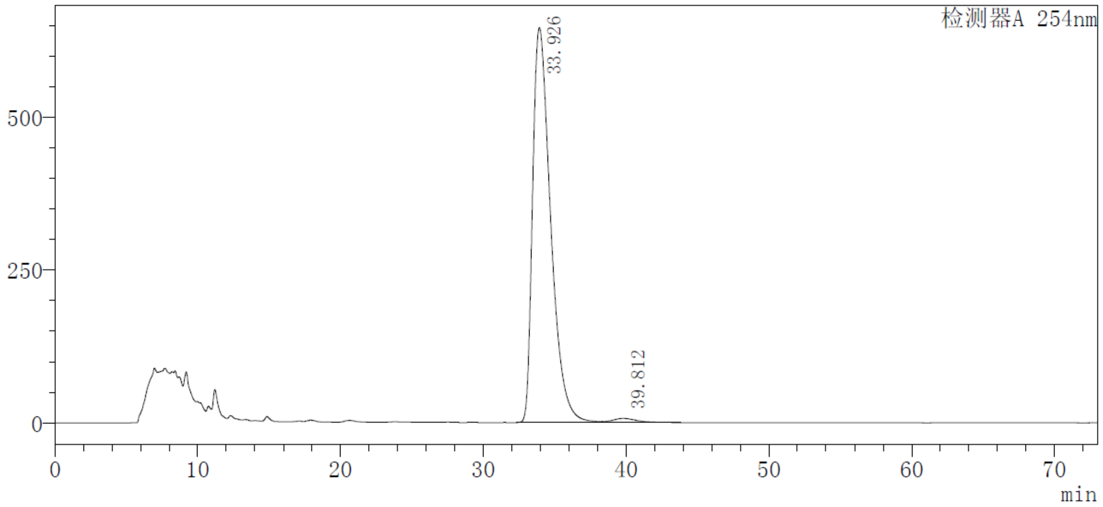

Peak	Ret. Time	Area %	Ee
1	33.926	98.596	97
2	39.812	1.404	

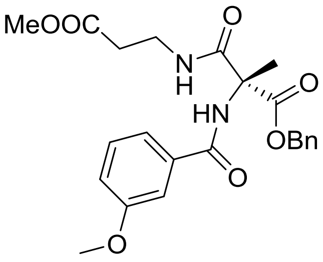

2s

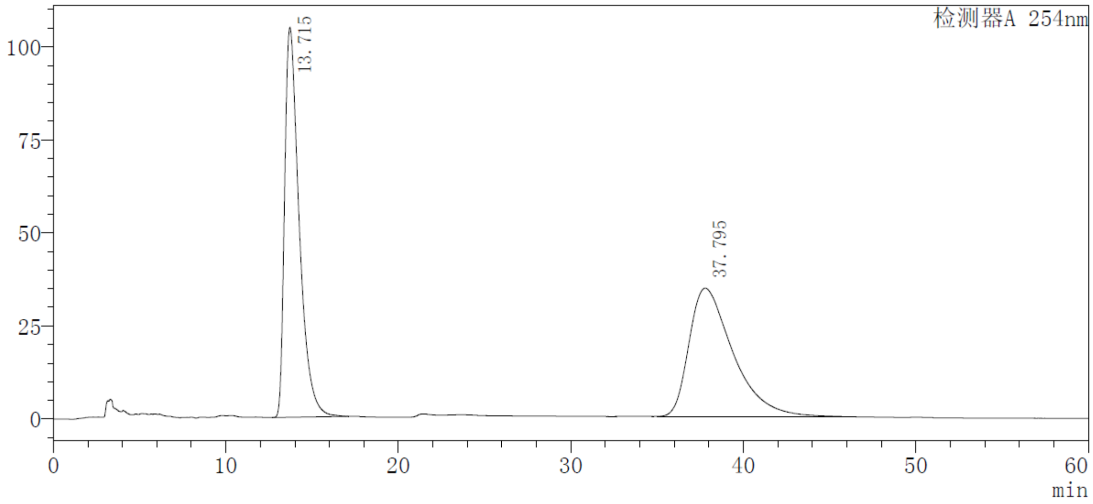

Peak	Ret. Time	Area %
1	13.715	50.187
2	37.795	49.813

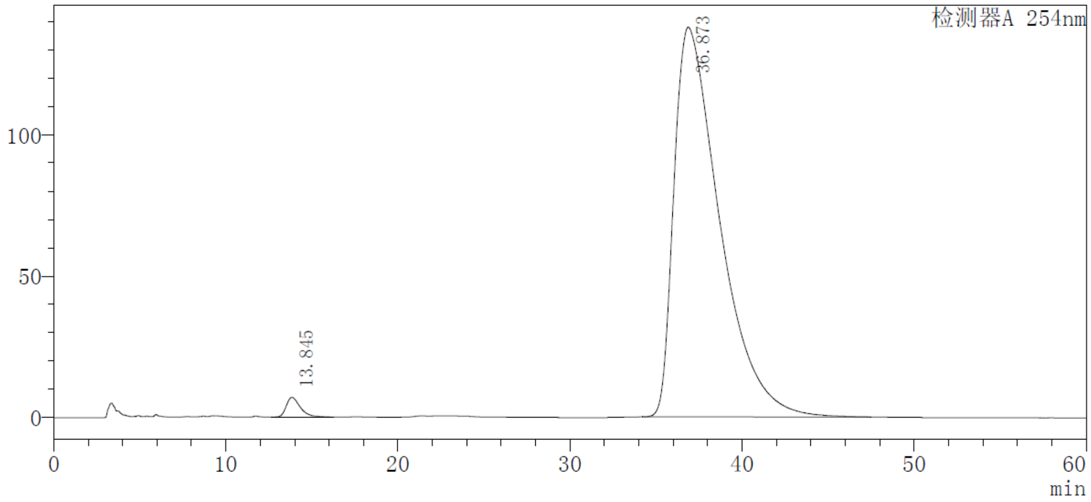

Peak	Ret. Time	Area %	Ee
1	13.845	1.545	
2	36.873	98.455	97

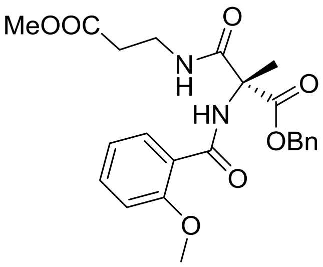

2t

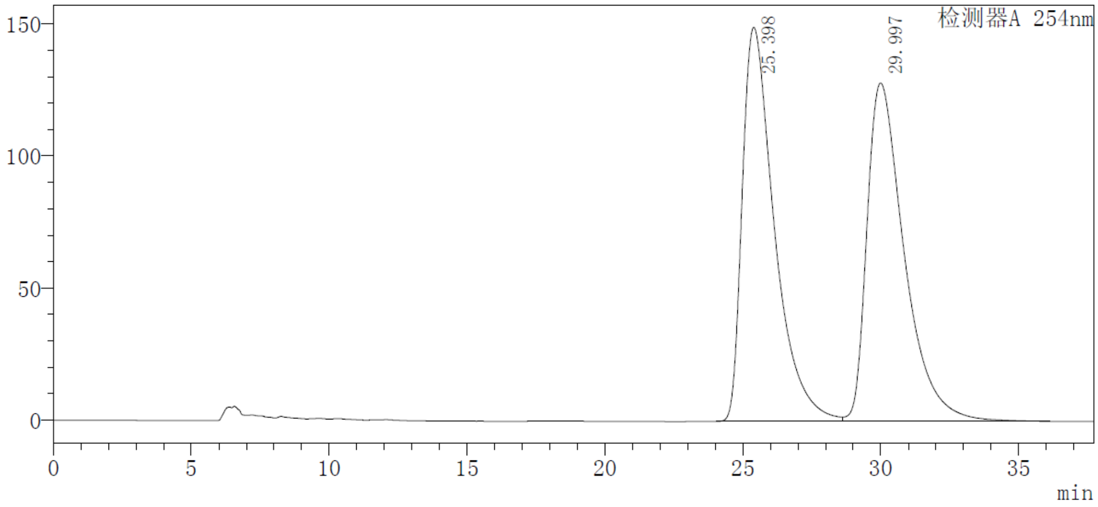

Peak	Ret. Time	Area %
1	25.398	50.002
2	29.997	49.998

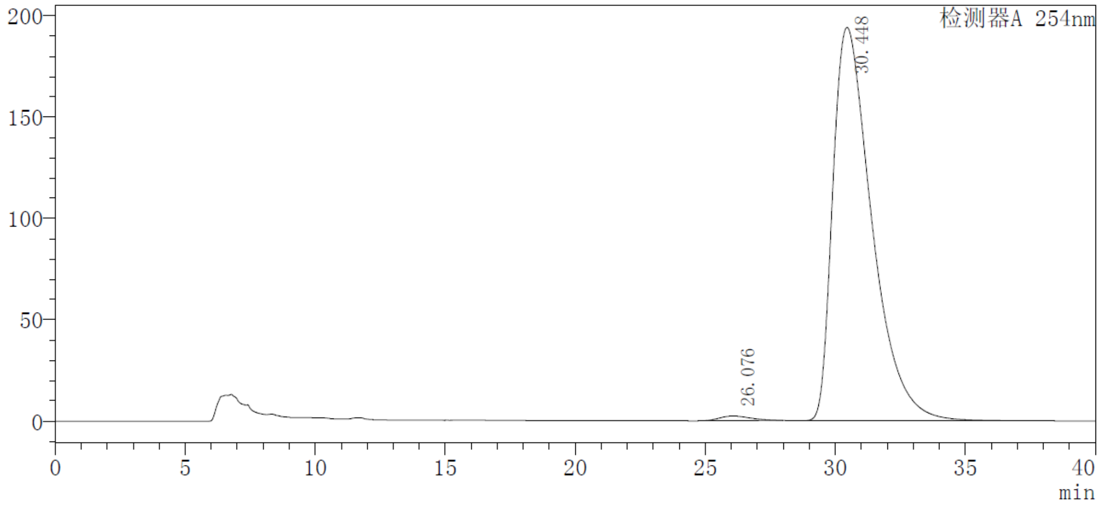

Peak	Ret. Time	Area %	Ee
1	26.076	0.938	
2	30.448	99.062	98

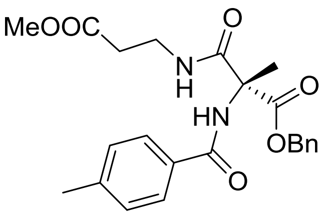

2u

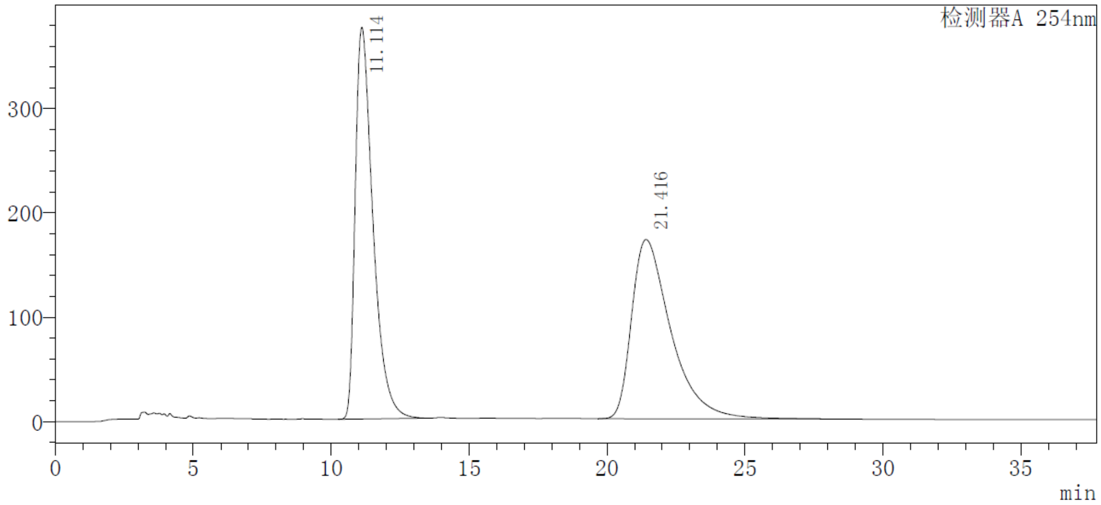

Peak	Ret. Time	Area %
1	11.114	49.621
2	21.416	50.379

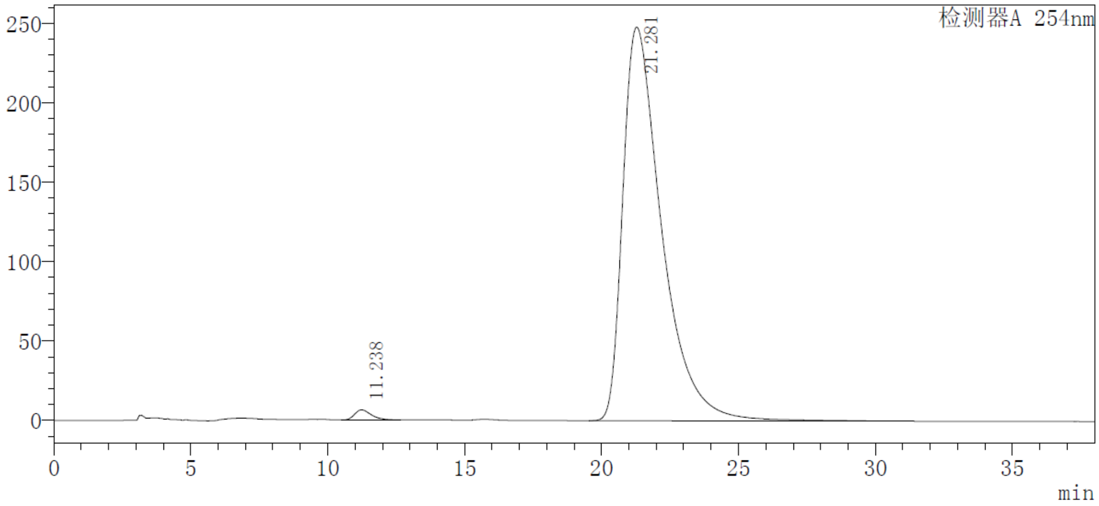

Peak	Ret. Time	Area %	Ee
1	11.238	1.088	
2	21.281	98.912	98

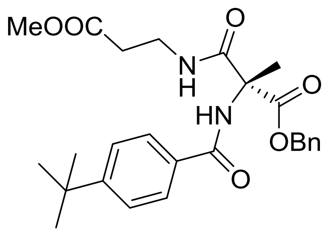

2v

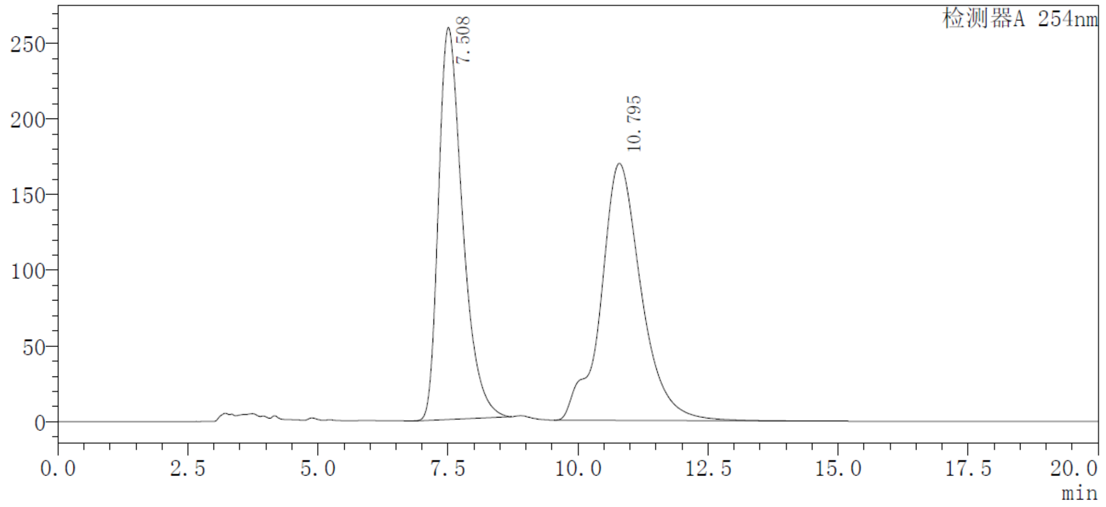

Peak	Ret. Time	Area %
1	7.508	47.488
2	10.795	52.512

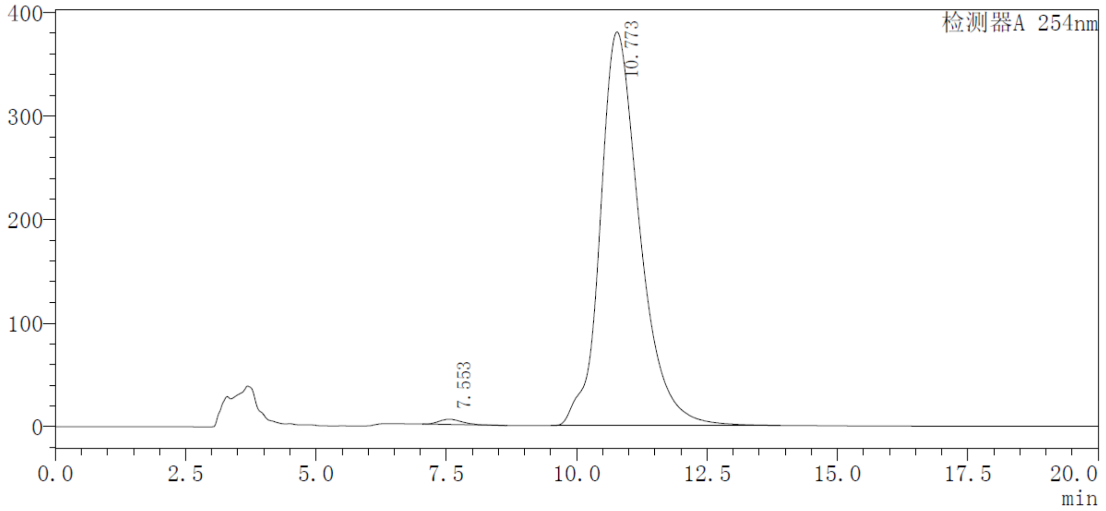

Peak	Ret. Time	Area %	Ee
1	7.553	0.804	
2	10.773	99.196	98

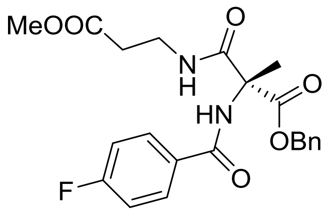

2w

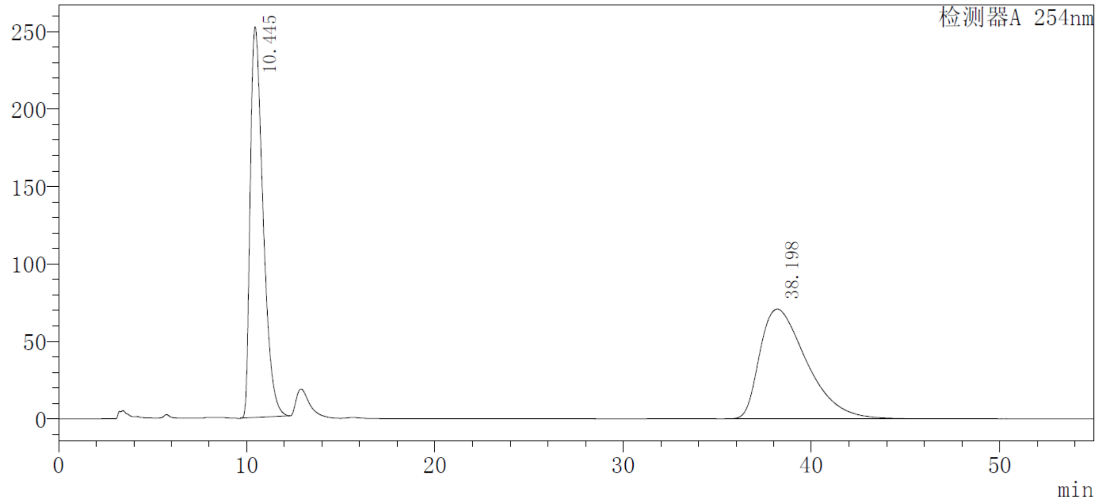

Peak	Ret. Time	Area %
1	10.445	49.398
2	38.198	50.602

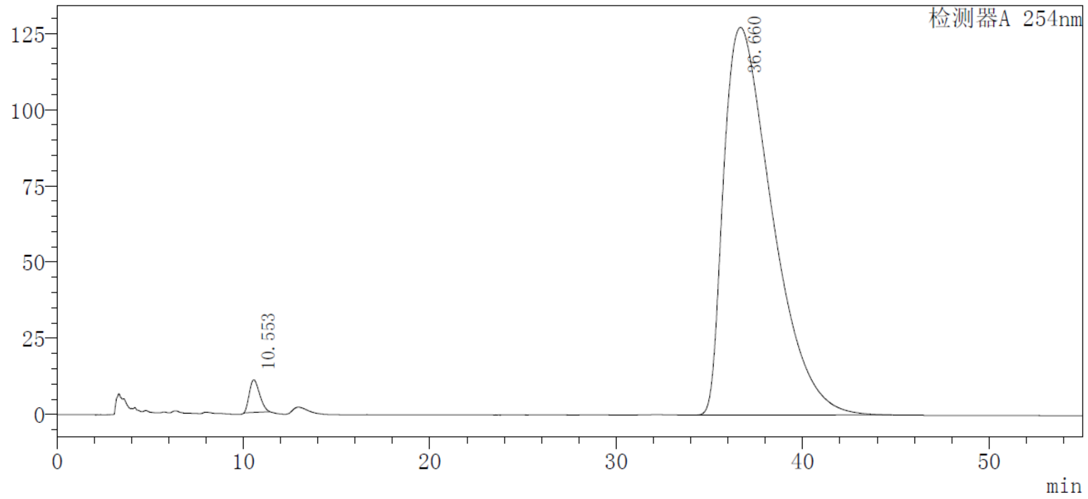

Peak	Ret. Time	Area %	Ee
1	10.553	1.746	
2	36.660	98.254	97

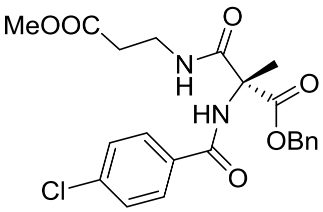

2x

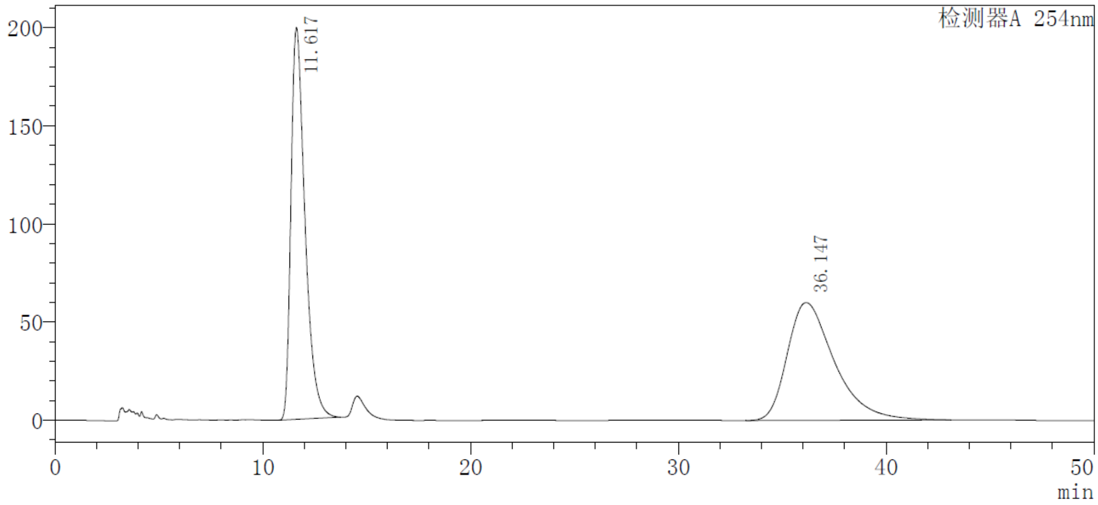

Peak	Ret. Time	Area %
1	11.617	49.723
2	36.147	50.277

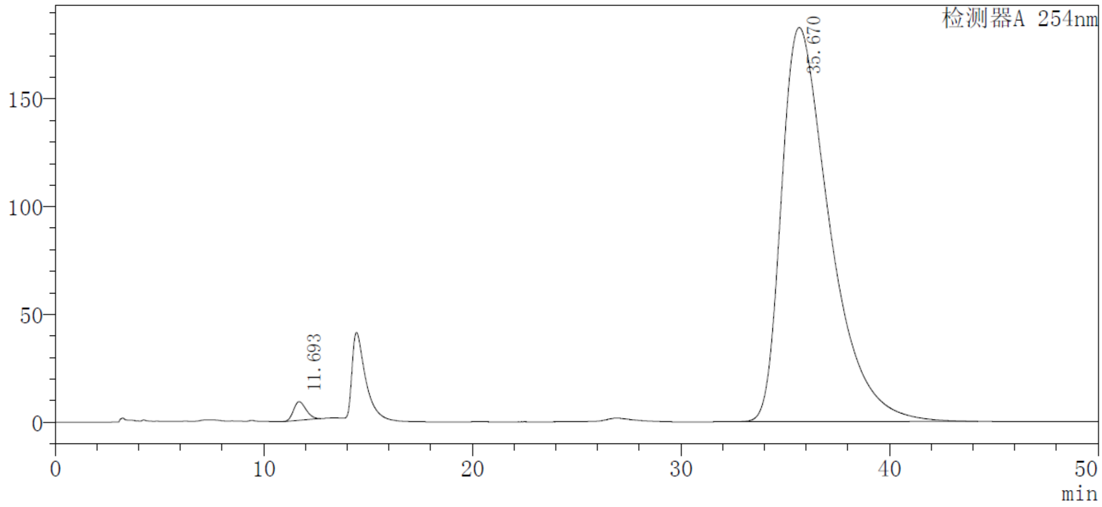

Peak	Ret. Time	Area %	Ee
1	11.693	1.205	
2	35.670	98.795	98

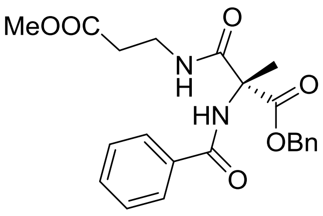

2y

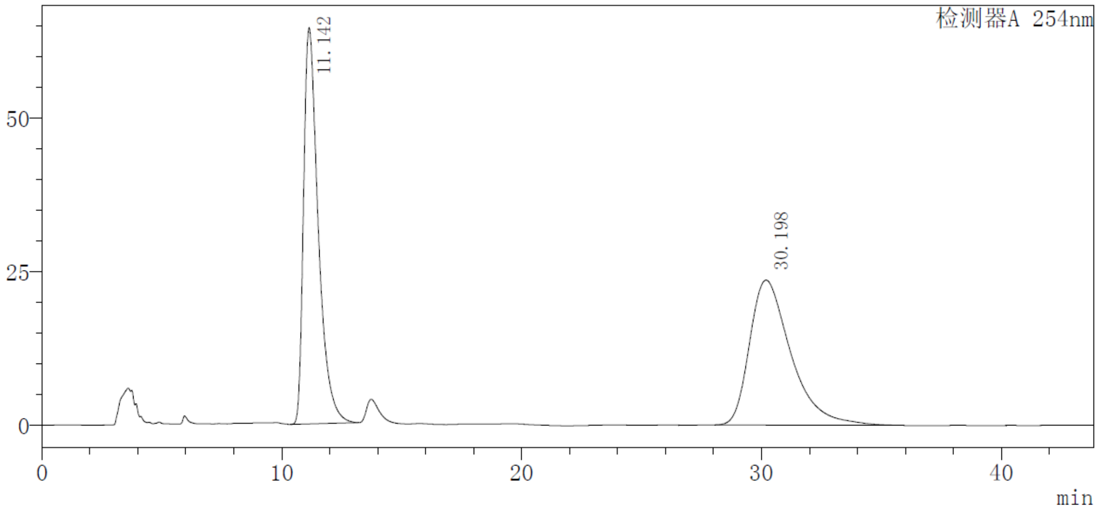

Peak	Ret. Time	Area %
1	11.142	49.296
2	30.198	50.704

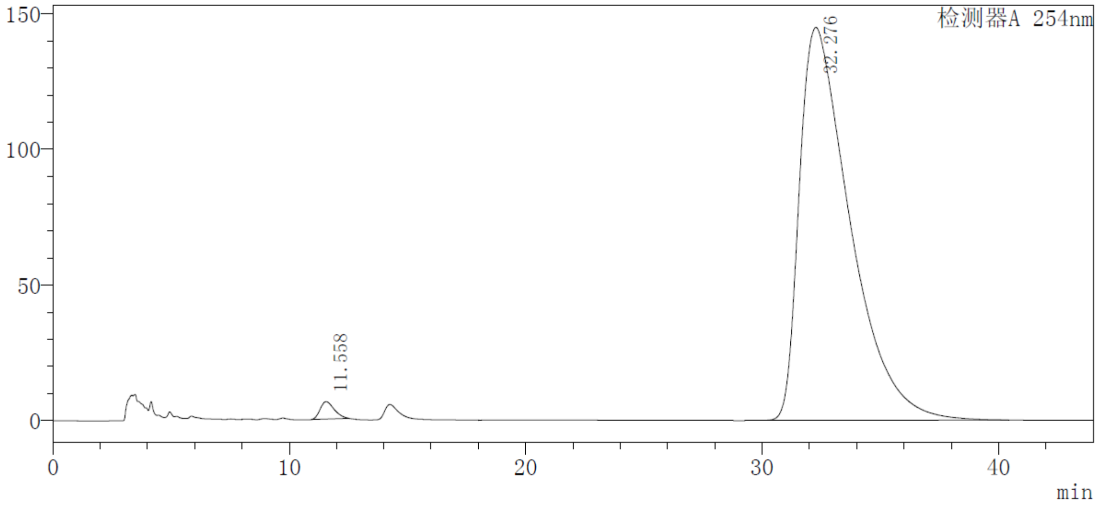

Peak	Ret. Time	Area %	Ee
1	11.558	1.221	
2	32.276	98.779	98

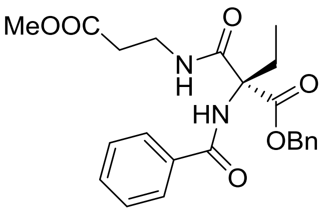

2z

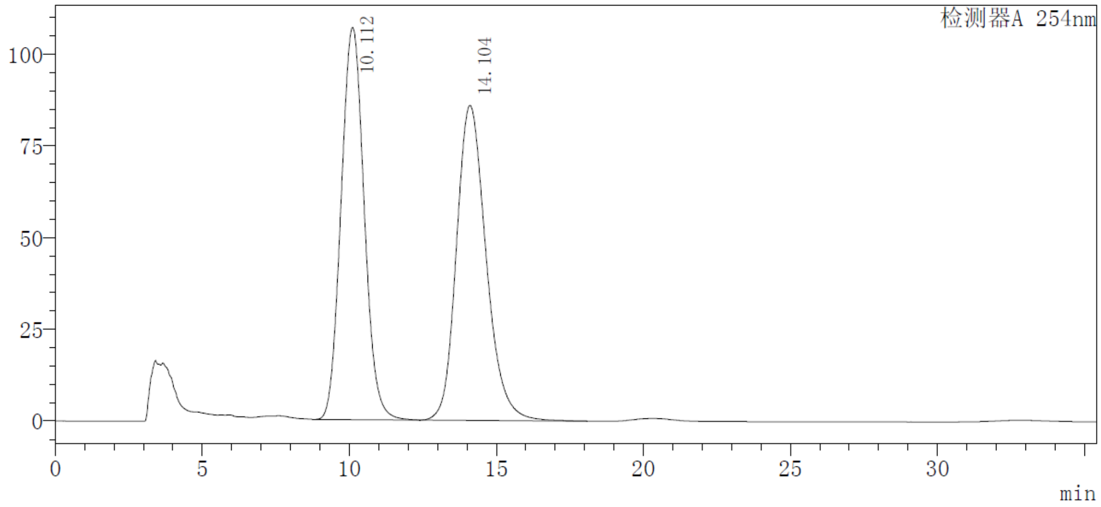

Peak	Ret. Time	Area %
1	10.112	49.198
2	14.104	50.802

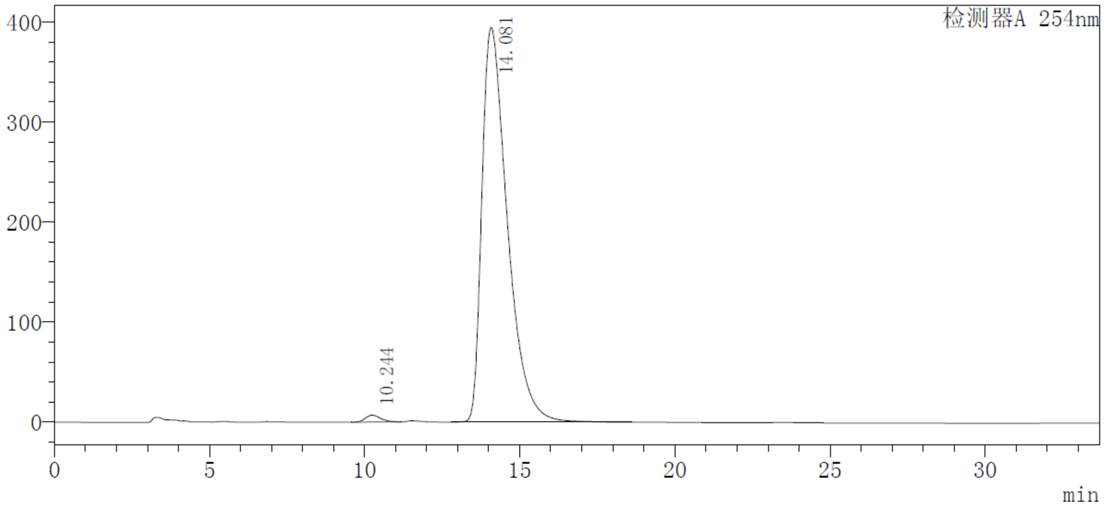

Peak	Ret. Time	Area %	Ee
1	10.244	1.021	
2	14.081	98.979	98

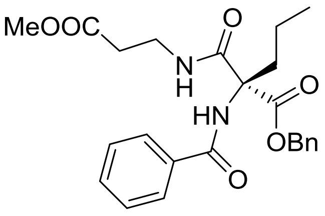

2aa

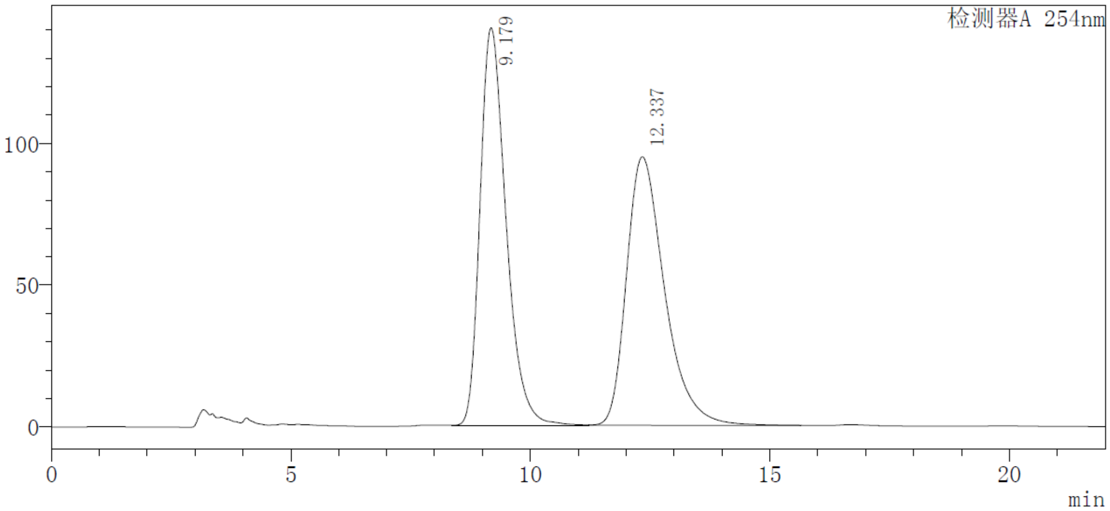

Peak	Ret. Time	Area %
1	9.179	50.262
2	12.337	49.738

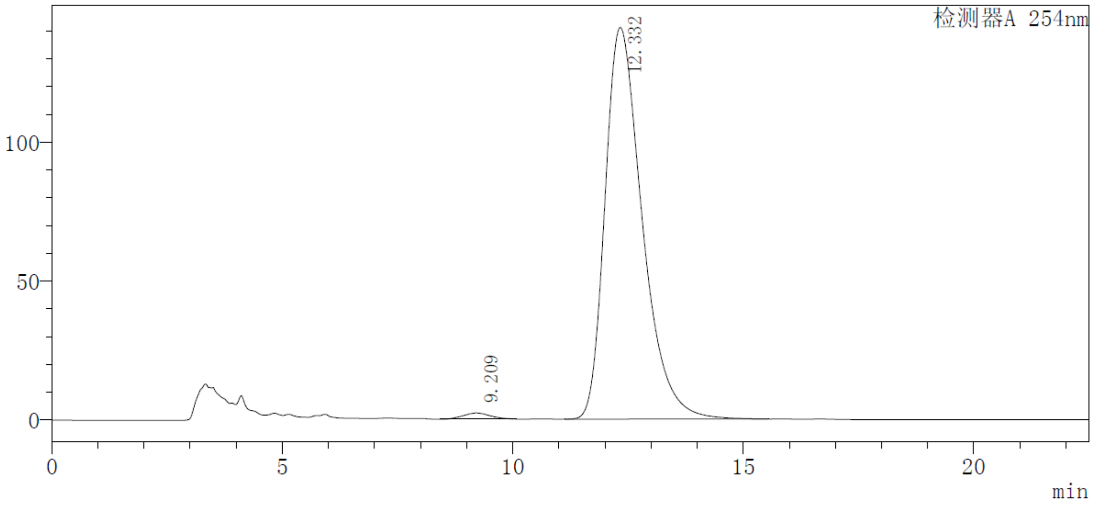

Peak	Ret. Time	Area %	Ee
1	9.209	1.160	
2	12.332	98.840	98

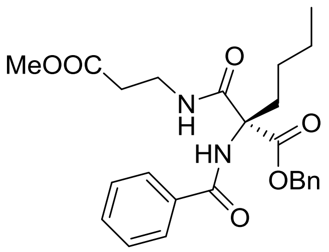

2ab

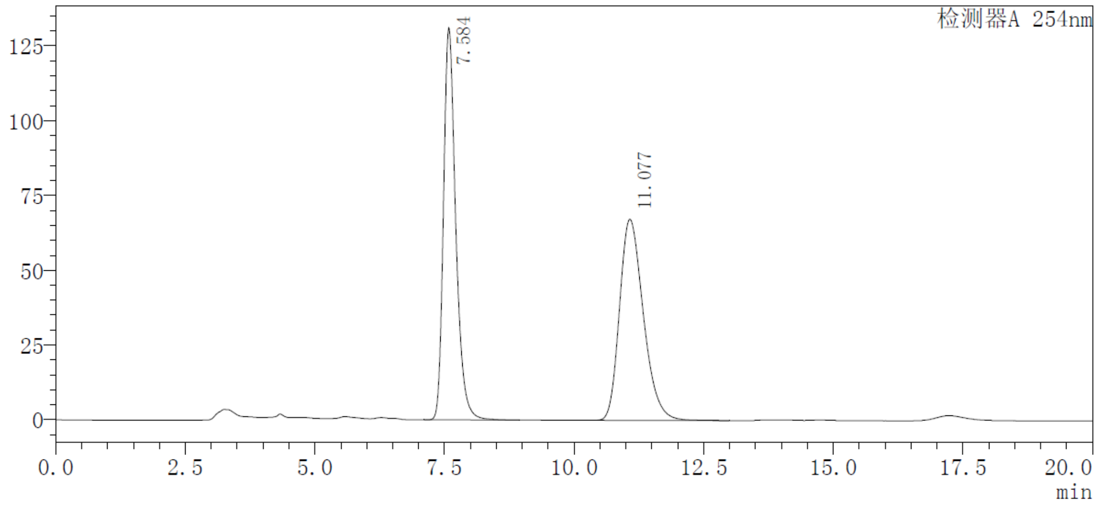

Peak	Ret. Time	Area %
1	7.584	50.033
2	11.077	49.967

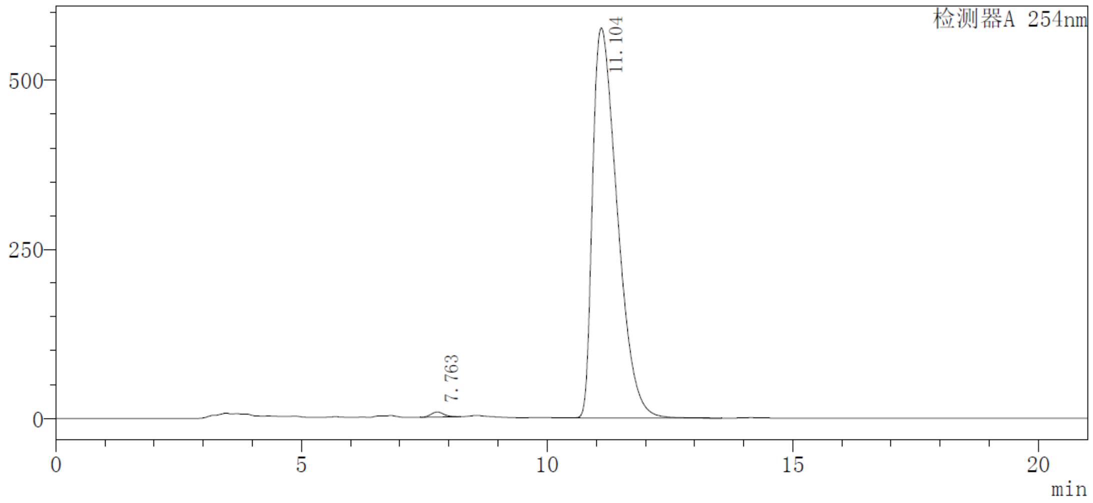

Peak	Ret. Time	Area %	Ee
1	7.763	0.669	
2	11.104	99.331	99

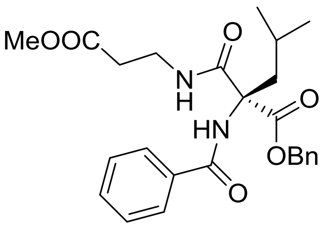

2ac

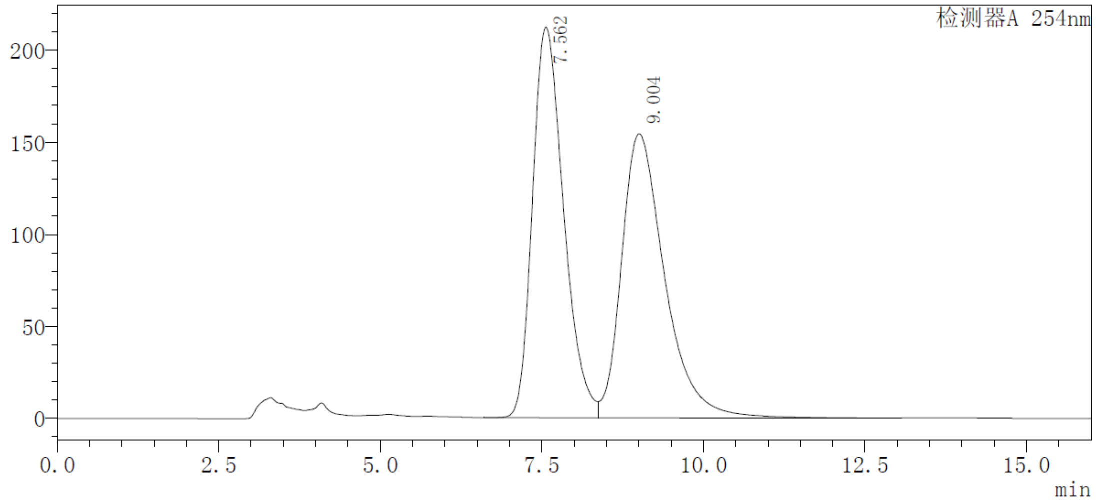

Peak	Ret. Time	Area %
1	7.562	49.817
2	9.004	50.183

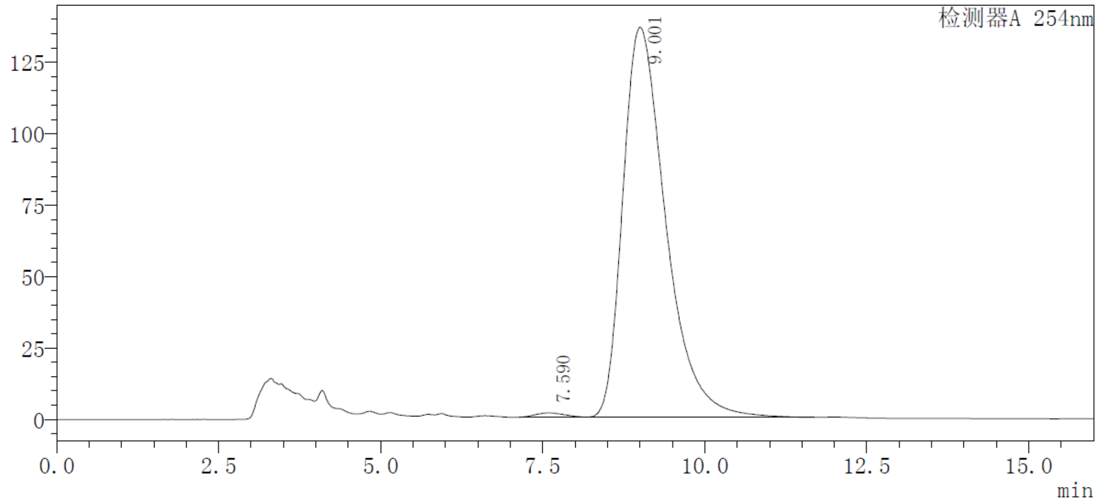

Peak	Ret. Time	Area %	Ee
1	7.590	0.726	
2	9.001	99.274	99

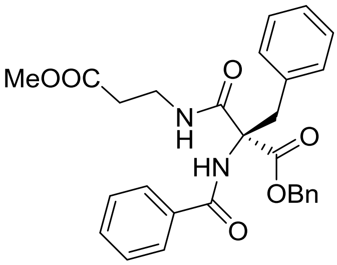

2ad

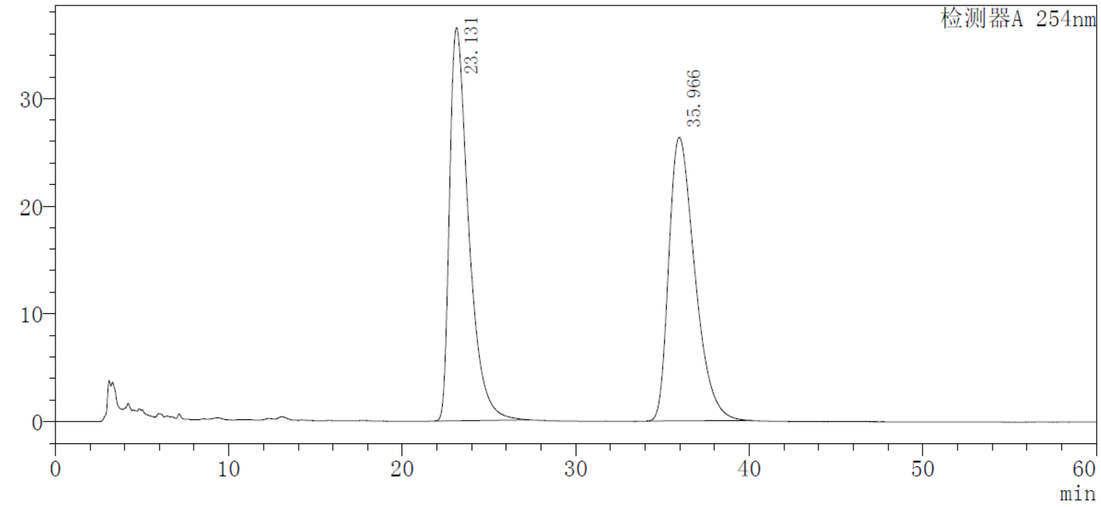

Peak	Ret. Time	Area %
1	23.131	50.455
2	35.966	49.545

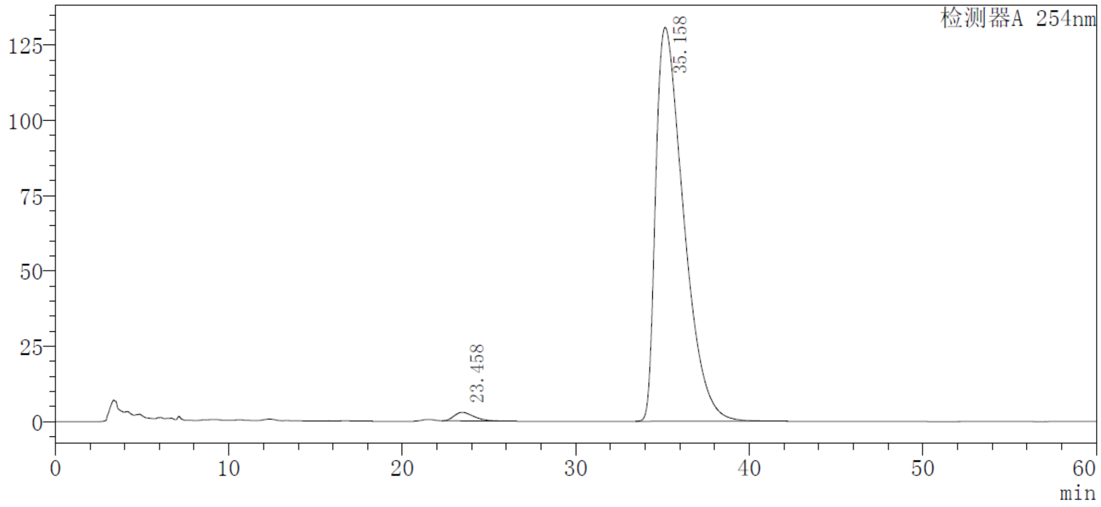

Peak	Ret. Time	Area %	Ee
1	23.458	1.506	
2	35.158	98.494	97

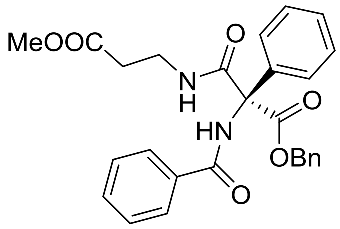

2ae

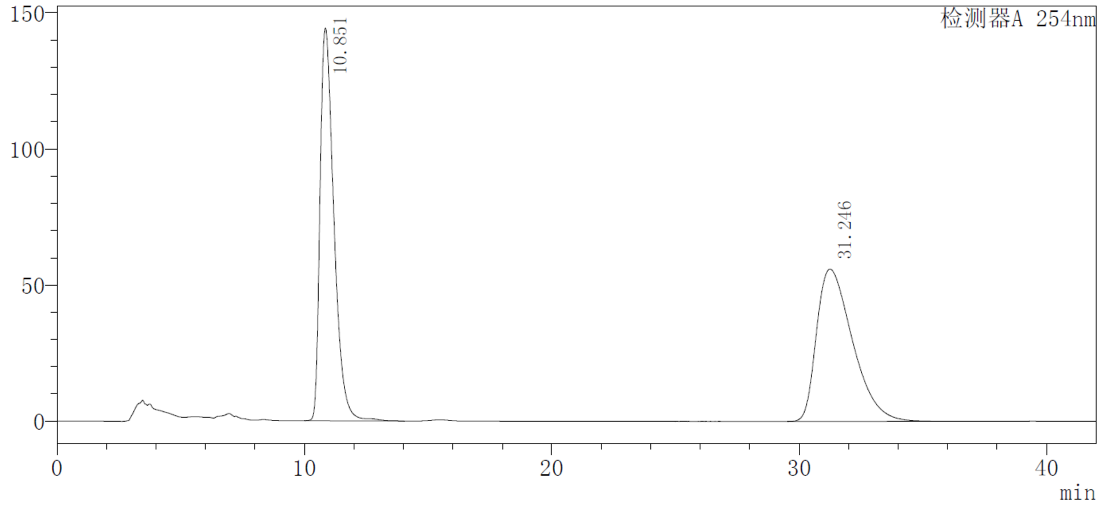

Peak	Ret. Time	Area %
1	10.851	49.854
2	31.246	50.146

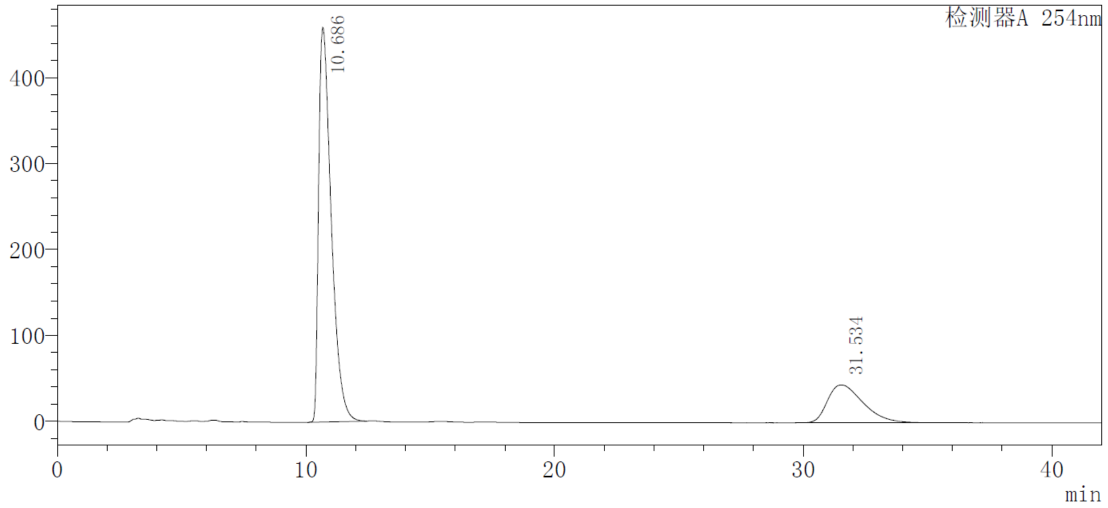

Peak	Ret. Time	Area %	Ee
1	10.686	78.617	57
2	31.534	21.383	

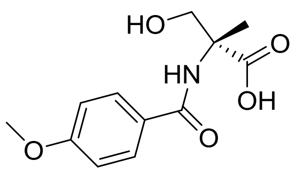

(*R*)-10

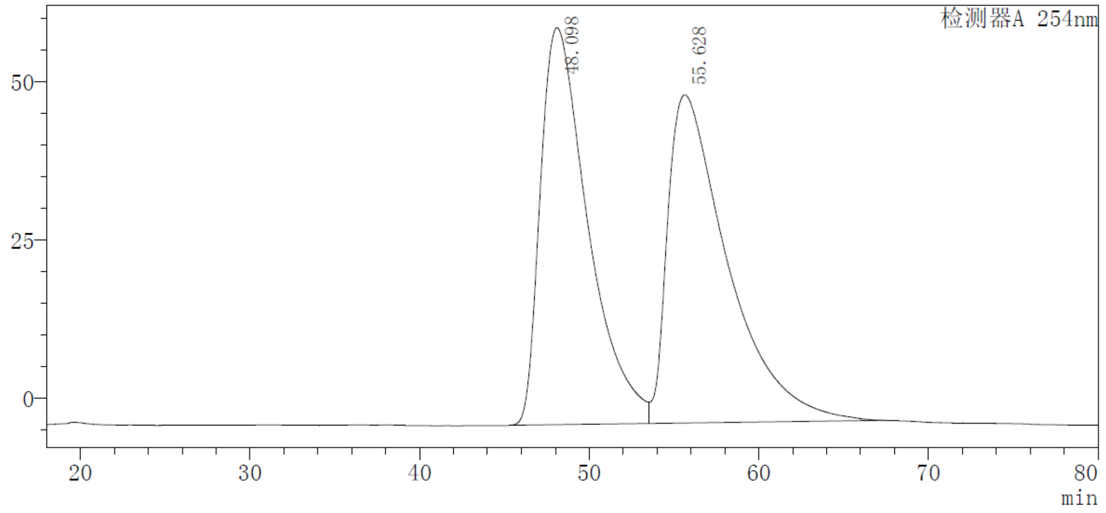

Peak	Ret. Time	Area %
1	48.098	48.860
2	55.628	51.140

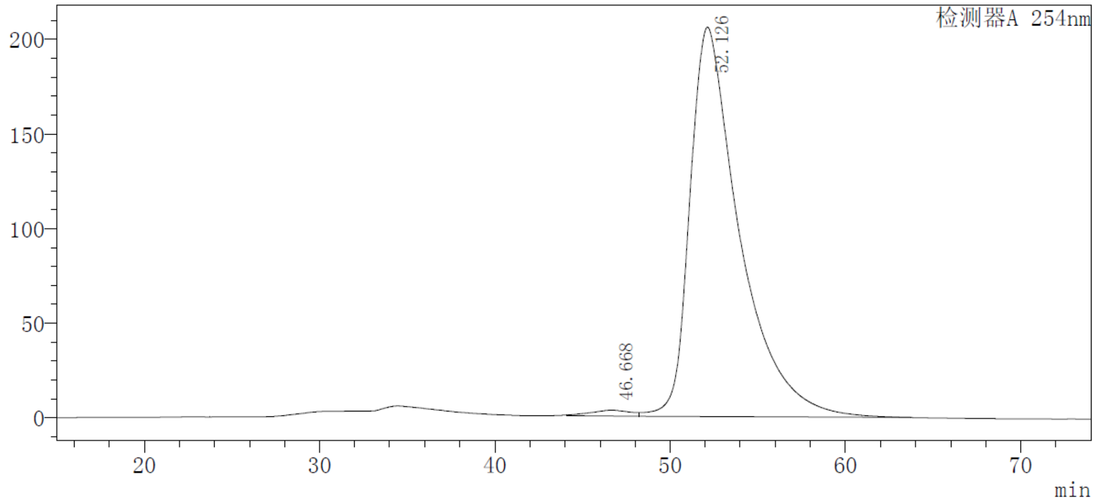

Peak	Ret. Time	Area %	Ee
1	46.668	1.182	
2	52.126	98.818	98

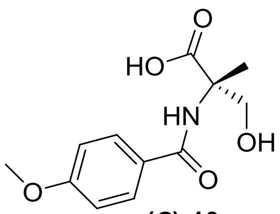

(*S*)-10

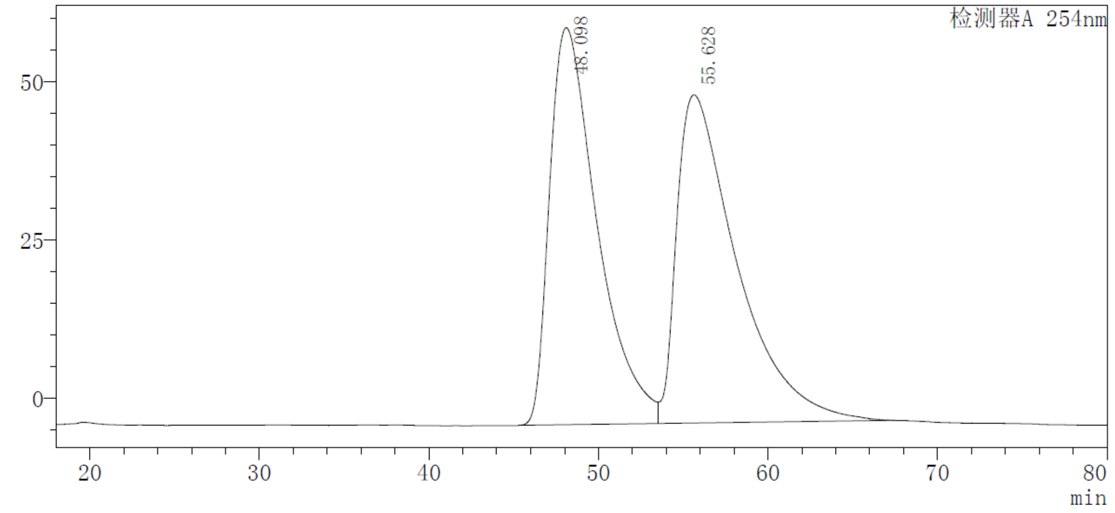

Peak	Ret. Time	Area %
1	48.098	48.860
2	55.628	51.140

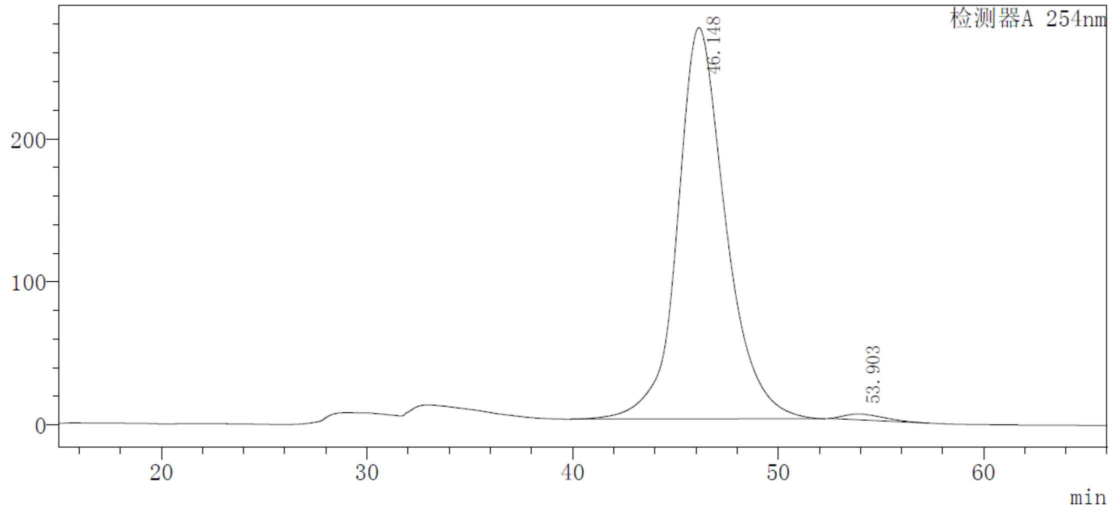

Peak	Ret. Time	Area %	Ee
1	46.148	98.774	98
2	53.903	1.226	

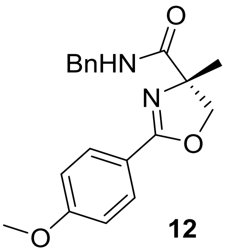

12

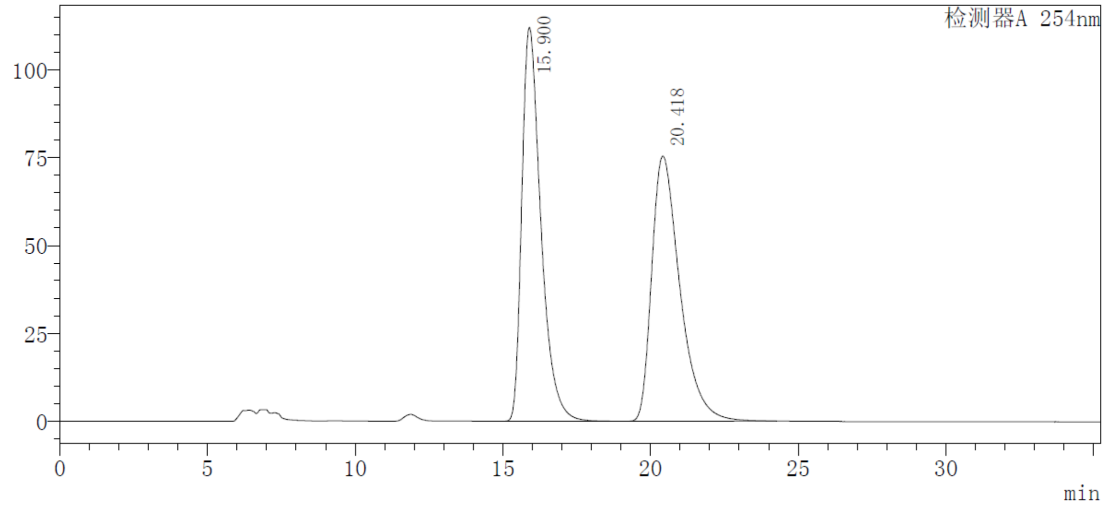

Peak	Ret. Time	Area %
1	15.900	50.147
2	20.418	49.853

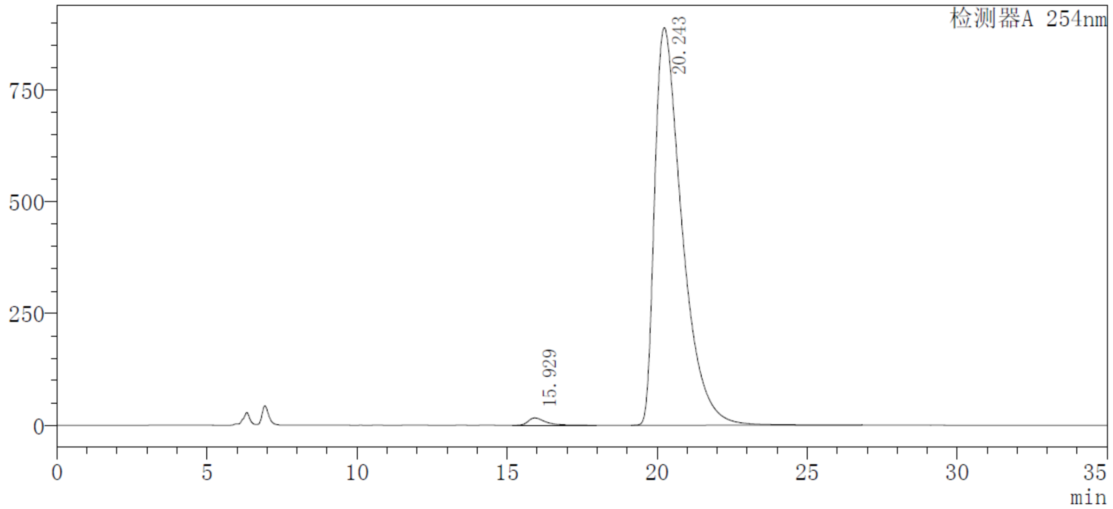

Peak	Ret. Time	Area %	Ee
1	15.929	1.118	
2	20.243	98.882	98
